# Supplementary material for: Sensitivity of outcome instruments in a priori selected patient groups after traumatic brain injury: Results from the CENTER-TBI study
Source: PLoS One. 2023 Apr 7;18(4):e0280796. doi: 10.1371/journal.pone.0280796 (PMC10081802; doi:10.1371/journal.pone.0280796)
Supplement: S1 Table — (PDF) [file pone.0280796.s001.pdf]

S1 Table. Overview of studies on protective and risk factors for the selected outcome areas after TBI.

Factor	Functional recovery (GOSE)	Generic HRQOL	TBI-specific HRQOL[1]	Anxiety	Depression	Post-traumatic stress disorder (PTSD)	Post-concussion symptoms
**Sex/Gender**	**SR: Worse outcomes in women than men (GOSE,** *post-concussion symptoms, quality of life, depression, PTSD***)** (1)<br><br>**CSS (CENTER-TBI): Poorer outcomes at six months after injury in women after** ***mild TBI*** compared with men **(GOSE,** *generic and disease-specific HRQOL, post-concussion symptoms, depression and anxiety*) (2)	**SR: Worse outcomes in women than men** (GOSE, post-concussion symptoms, **quality of life,** depression, PTSD) (1)<br><br>**LS:** Association between the *GOSE scores and all SF-36 domains and PQoL scores.* **Decreased HRQOL at 6 and 12 months** after TBI in **females,** *indviduals of advanced age*, and in those with co-morbidities and *high ISS*. (3)	**CSS: Decreased TBI-specific HRQOL** in ***older*** **females (54–76-years at TBI)** (4)	**SR: Higher risk for anxiety** and *depressive disorders* **after TBI in females**, those without employment, and *those with psychiatric history before TBI* (5)	**SR: Higher risk for** *anxiety* and **depressive disorders after TBI in females**, those without employment, and *those with psychiatric history before TBI* (5)	**SR: Worse outcomes in women than men** (*GOSE, post-concussion symptoms, quality of life, depression*, **PTSD**) (1)<br><br>**CSS: Higher PTSD risk in men** comapred to women after TBI (6)	**SR: Poorer outcome** in *older age* and **female gender** (7)<br><br>**CSS: Worse symptom burden in women than in men** (8)
**Age**	**SR/MA:** Worse outcomes in older patients after mild TBI (**GOSE**), but in long-term same amount of fully recovered as in younger adults; *less severe symptom burden* in terms of psychological health (*post-concussion symptoms, anxiety and depression*) (9)<br><br>**SR/MA:** Continuos association of **older**	**SR: Poorer** *post-injury functioning* and **HRQoL in older adults** compared with younger adults or preinjury levels (11)<br><br>**LS:** Association between the *GOSE scores and all SF-36 domains and PQoL scores*. **Decreased HRQOL at 6 and 12 months** after TBI in *females*, **indviduals of**	**CSS: Higher HRQOL values** in cognition and autonomy and daily life i**n younger patients** among elderly (i.e., > 50 years of age) (12)<br><br>**CSS: Decreased TBI-specific HRQOL in older** ***females*** **(54–76-years at TBI)** (4)	**CSS: Significant anxiety**, *depression*, and somatic preoccupation i**n patients aged 50 and above** despite their improvement on objective neuropsychological measures (13)<br><br>**LS (CENTER-TBI): Younger age** related to **more severe generalized anxiety disorder** (14)	**SR: Increased risk of new-onset depression** and *PTSD* **in older adults** (15)<br><br>**CSS: Significant** *anxiety*, **depression**, and somatic preoccupation **in patients aged 50** and above despite their improvement on objective neuropsychological measures (13)	**SR: Increased risk of new-onset** *depression* and **PTSD in older adults** (15)	**SR: Poorer outcome** in **older age** and *female gender* (7)

Factor	Functional recovery (GOSE)	Generic HRQOL	TBI-specific HRQOL[1]	Anxiety	Depression	Post-traumatic stress disorder (PTSD)	Post-concussion symptoms
	**age** with a **worsening outcome** after TBI (10)	**advanced age**, and in those with co-morbidities and *high ISS* (3)					
**Education**	**LS:** Significant association of the **longer education duration with a lower GOSE score** (16)<br><br>**CSS: Significant association of the poorer outcome on the GOSE** with **less education** (17)	**LS:** Association between **persistent low generic** and *disease-specific* **HRQOL** and **low education level** (18)	**CSS:** Association of *depression*, amount of help needed, *anxiety*, **education level** and *age* at injury with generic and TBI-specific HRQOL (19)<br><br>**LS:** Association between **persistent low** *generic* and **disease-specific HRQOL** and **low education level** (18)	**LS:** Association of **lower educational level** with greater *post-concussion symptoms*, *depression*, **anxiety**, *PTSD* severity (8)	**LS:** Association of **lower educational level** with greater *post-concussion symptoms*, **depression**, *anxiety*, *PTSD* severity (8)	**LS:** Association of **lower educational level** with greater *post-concussion symptoms*, *depression*, *anxiety*, **PTSD** severity (8)<br><br>**CSS (CENTER-TBI): Lower levels of education**, more frequently reported *history of psychiatric disorders* and more often being injured in road traffic accidents or by violence in participants with suspected PTSD (20)	**LS:** Association of **lower educational level** with greater **post-concussion symptoms,** *depression*, *anxiety*, *PTSD* severity (8)<br><br>**CSS (CENTER-TBI):** Association of *female gender*, **lower education**, being injured by assault compared with other causes of injury, *having higher ISS* and AISH scores **with higher RPQ total score** (21)
**Premorbid psychological problems**	**CSS: Worse functional recovery** at one year after TBI for participants **with pre-injury anxiety and depresssive disorder** (22)<br><br>**CSS: Worse functional recovery** at six months after TBI **for those suffering from pre-injury mental**	**SR: Pre-existing conditions** are associated **with poorer outcomes (including HRQOL)** (11)	**CSS (CENTER-TBI): Strong association** of type of employment, *level of education*,and **pre-injury mental health problems** with the QOLIBRI total score at 6 months after TBI (24)	**SR: Higher risk for anxiety** and *depressive disorder*s after TBI in *females*, those without employment, and those **with psychiatric history before TBI** (5)<br><br>**LS: Pre-injury mental health treatment** as a risk factor for developing higher and more	**LS: Higher risk for having** *PTSD* or **major depression disoreder (MDD)** postinjury for indiviudals **with an antecedent mental health problem prior to TBI** (26)<br><br>**SR: Higher risk for** *anxiety* and **depressive disorders after TBI** in *females*, those	**LS: Higher risk** for having **PTSD** or *major depression disoreder (MDD)* postinjury for indiviudals with **an antecedent mental health problem prior to TBI** (26)	**SR:** *Female gender*, *lower education*, **pre-injury mental health status** as a significant **predictor for developing of post-concussion symptoms** (27)<br>**LS: Higher risk for worse** *functional* and **post-concussive outcomes** at 3- and 6-months post mild TBI for individuals with **pre-injury**

Factor	Functional recovery (GOSE)	Generic HRQOL	TBI-specific HRQOL[1]	Anxiety	Depression	Post-traumatic stress disorder (PTSD)	Post-concussion symptoms
	**health probelms** (23)			persistent levels of anxiety during 10 years after TBI (25)	without employment, and **those with psychiatric history before TBI** (5)		**psychiatric symptoms** (28)
**Clinical care pathways**	**CSS (CENTER-TBI): Unfavourable recovery (GOSE)**, *decreased generic* and *disease-specific HRQOL* **also in patients being discharged home after seen in the emergency room** (29)	**CSS (CENTER-TBI):** *Unfavourable recovery (GOSE)*, **decreased generic** and *disease-specific* **HRQOL also in patients being discharged home after seen in the emergency room** (29)	**CSS (CENTER-TBI): Unfavourable recovery (GOSE)**, **decreased** *generic* and **disease-specific HRQOL also in patients being discharged home after seen in the emergency room** (29)	**LS (CENTER-TBI):** Higher risk for **more severe** *major depression (MD)* and **generalized anxiety disorder (GAD)** for those being more severely disabled, having experienced major extracranial injuries, **an intensive care unit stay**, and being *female* (14)	**LS (CENTER-TBI):** Higher risk for **more severe major depression (MD)** and *generalized anxiety disorder (GAD)* for those being more severely disabled, having experienced major extracranial injuries, **an intensive care unit stay**, and being *female* (14)	**CSS: Higher risk to devlop PTSD** for injury patients (including TBI) admitted to an **ICU** (30)	**CSS (CENTER-TBI): Higher probability of more intense post-concussion symptoms in those admitted to an ICU** compared to ER , having experienced *a complicated mTBI compared to severe TBI*, and *having premorbid psychological problems* prior to TBI (31)
**Injury severity score / extracranial injuries**	**SR/MA: Injury severity score (ISS)** as a significant predictor of **global disability and global outcomes (GOSE)** postinjury (32)	**LS:** Association between the GOSE scores and all SF-36 domains and PQoL scores. **Decreased HRQOL at 6 and 12 months** after TBI in *females*, *indviduals of advanced age*, and in those with co-morbidities and **high ISS** (3)	**CSS: Negative associations of the TBI-specific HRQOL** with **exteremities and head trauma score** one year after TBI (Abbreviated Injury Scale, AIS) (33)	**LS (CENTER-TBI):** Higher risk for *more severe major depression (MD)* and **generalized anxiety disorder (GAD)** for those being more severely disabled, **having experienced major extracranial injuries**, *an intensive care unit stay*, and being *female* (14)	**LS (CENTER-TBI):** Higher risk for **more severe major depression (MD)** and *generalized anxiety disorder (GAD)* for those being more severely disabled, **having experienced major extracranial injuries**, *an intensive care unit stay*, and being *female* (14)	**SR/MA: Presence of phyiscal injuries** increases the **risk of developing of PTSD** after TBI (34)	**CSS (CENTER-TBI):** Probability of occurence of post-concussion symptoms is assocated with employment type, **Injury Severity Score (ISS)**, *TBI severity*, and presence of *premorbid health problems* prior to TBI (31)

Factor	Functional recovery (GOSE)	Generic HRQOL	TBI-specific HRQOL[1]	Anxiety	Depression	Post-traumatic stress disorder (PTSD)	Post-concussion symptoms
**TBI severity (uncomplicated mild, complicated mild, moderate, severe)**	**SR/MA: Injury severity (GCS)** as a significant predictor **of global disability and global outcomes (GOSE)** and **quality of life (including SF-36)** postinjury (32)	**SR/MA: Injury severity (GCS)** as a significant predictor **of** *global disability and global outcomes (GOSE)* and **quality of life (including SF-36)** postinjury (32)	**CSS: Lower TBI-specific HRQOL** is associated **with more severe TBI** (i.e., lower GCS) one year after TBI (33)	**CSS:** Increased **anxiety** and *depression* in *females* and patients with more severe TBI (35)	**CSS:** Increased *anxiety* and **depression** in *females* and patients with more severe TBI (35)	**SR/MA:** No clear effect of TBI severity on development of the PTSD (36)	**LS: Long-term post-cocnussion-like symptoms after severe TBI** (37)<br><br>**CSS (CENTER-TBI): Probability of occurrence of post-concussion symptoms** is assocated with employment type, *Injury Severity Score (ISS)*, **TBI severity**, and *presence of premorbid health problems* prior to TBI (31)

[1] Low availability of reviews or meta-analyses due to limited number of instruments measuring TBI-specific HRQOL.

Note. SR = Systematic review, MA = meta-analysis, CSS = cross-sectional study, LS = longitudinal study, CENTER-TBI = studies based on CENTER-TBI data, **bold** entries = association between respective risk factor and the outcome, *italic* entries = association between respective risk factors and other outcomes. SR and MA were preferred over all other types of studies. For CSS and LS, non-CENTER-TBI studies were preferred over CENTER-TBI studies.

1. Gupte RP, Brooks WM, Vukas RR, Pierce JD, Harris JL. Sex Differences in Traumatic Brain Injury: What We Know and What We Should Know. Journal of Neurotrauma. 2019 Nov 15;36(22):3063–91.

2. Mikolić A, van Klaveren D, Oude Groeniger J, Wiegers EJA, Lingsma HF, Zeldovich M, et al. Differences between Men and Women in Treatment and Outcome after Traumatic Brain Injury. Journal of Neurotrauma. 2020 Oct 19;neu.2020.7228.

3. Scholten AC, Haagsma JA, Andriessen TMJC, Vos PE, Steyerberg EW, van Beeck EF, et al. Health-related quality of life after mild, moderate and severe traumatic brain injury: Patterns and predictors of suboptimal functioning during the first year after injury. Injury. 2015 Apr;46(4):616–24.

4. Rauen K, Späni CB, Tartaglia MC, Ferretti MT, Reichelt L, Probst P, et al. Quality of life after traumatic brain injury: a cross-sectional analysis uncovers age- and sex-related differences over the adult life span. GeroScience. 2021 Feb;43(1):263–78.

5. Scholten AC, Haagsma JA, Cnossen MC, Olff M, van Beeck EF, Polinder S. Prevalence of and Risk Factors for Anxiety and Depressive Disorders after Traumatic Brain Injury: A Systematic Review. Journal of Neurotrauma. 2016 Nov 15;33(22):1969–94.

6. Iverson KM, Hendricks AM, Kimerling R, Krengel M, Meterko M, Stolzmann KL, et al. Psychiatric Diagnoses and Neurobehavioral Symptom Severity among OEF/OIF VA Patients with Deployment-Related Traumatic Brain Injury: A Gender Comparison. Women’s Health Issues. 2011 Jul;21(4):S210–7.

7. King NS. A systematic review of age and gender factors in prolonged post-concussion symptoms after mild head injury. Brain Injury. 2014 Dec;28(13–14):1639–45.

8. Levin HS, Temkin NR, Barber J, Nelson LD, Robertson C, Brennan J, et al. Association of Sex and Age With Mild Traumatic Brain Injury–Related Symptoms: A TRACK-TBI Study. JAMA Netw Open. 2021 Apr 6;4(4):e213046.

9. Hume CH, Wright BJ, Kinsella GJ. Systematic Review and Meta-analysis of Outcome after Mild Traumatic Brain Injury in Older People. J Int Neuropsychol Soc. 2022 Aug;28(7):736–55.

10. Hukkelhoven CWPM, Steyerberg EW, Rampen AJJ, Farace E, Habbema JDF, Marshall LF, et al. Patient age and outcome following severe traumatic brain injury: an analysis of 5600 patients. Journal of Neurosurgery. 2003 Oct;99(4):666–73.

11. Brown K, Cameron ID, Keay L, Coxon K, Ivers R. Functioning and health-related quality of life following injury in older people: a systematic review. Inj Prev. 2017 Dec;23(6):403–11.

12. Lin YN, Hwang HF, Chen YJ, Cheng CH, Liang WM, Lin MR. Suitability of the Quality of Life after Brain Injury Instrument for Older People with Traumatic Brain Injury. Journal of Neurotrauma. 2016 Jul 15;33(14):1363–70.

13. Goldstein FC, Levin HS. Cognitive Outcome After Mild and Moderate Traumatic Brain Injury in Older Adults. Journal of Clinical and Experimental Neuropsychology. 2001 Dec;23(6):739–53.

14. Wang B, Zeldovich M, Rauen K, Wu YJ, Covic A, Muller I, et al. Longitudinal Analyses of the Reciprocity of Depression and Anxiety after Traumatic Brain Injury and Its Clinical Implications. JCM. 2021 Nov 28;10(23):5597.

15. Gardner RC, Dams-O'Connor K, Morrissey MR, Manley GT. Geriatric Traumatic Brain Injury: Epidemiology, Outcomes, Knowledge Gaps, and Future Directions. Journal of Neurotrauma. 2018 Apr;35(7):889–906.

16. Ruet A, Bayen E, Jourdan C, Ghout I, Meaude L, Lalanne A, et al. A Detailed Overview of Long-Term Outcomes in Severe Traumatic Brain Injury Eight Years Post-injury. Front Neurol. 2019 Feb 21;10:120.

17. Ponsford J, Draper K, Schönberger M. Functional outcome 10 years after traumatic brain injury: Its relationship with demographic, injury severity, and cognitive and emotional status. J Inter Neuropsych Soc [Internet]. 2008 Mar [cited 2022 Dec 2];14(02). Available from: http://www.journals.cambridge.org/abstract_S1355617708080272

18. Chiang CC, Guo SE, Huang KC, Lee BO, Fan JY. Trajectories and associated factors of quality of life, global outcome, and post-concussion symptoms in the first year following mild traumatic brain injury. Qual Life Res. 2016 Aug;25(8):2009–19.

19. Siponkoski S, Wilson L, Steinbüchel N, Sarajuuri J, Koskinen S. Quality of life after traumatic brain injury: Finnish experience of the QOLIBRI in residential rehabilitation. J Rehabil Med. 2013;45(8):835–42.

20. Van Praag DLG, Wouters K, Van Den Eede F, Wilson L, Maas AIR, Åkerlund C, et al. Neurocognitive correlates of probable posttraumatic stress disorder following traumatic brain injury. Brain and Spine. 2022;2:100854.

21. Voormolen DC, Polinder S, von Steinbuechel N, Vos PE, Cnossen MC, Haagsma JA. The association between post-concussion symptoms and health-related quality of life in patients with mild traumatic brain injury. Injury. 2019 May;50(5):1068–74.

22. Gould KR, Ponsford JL, Johnston L, Schönberger M. Relationship Between Psychiatric Disorders and 1-Year Psychosocial Outcome Following Traumatic Brain Injury. Journal of Head Trauma Rehabilitation. 2011 Jan;26(1):79–89.

23. van der Naalt J, Timmerman ME, de Koning ME, van der Horn HJ, Scheenen ME, Jacobs B, et al. Early predictors of outcome after mild traumatic brain injury (UPFRONT): an observational cohort study. The Lancet Neurology. 2017 Jul;16(7):532–40.

24. Helmrich IRAR, van Klaveren D, Dijkland SA, Lingsma HF, Polinder S, Wilson L, et al. Development of prognostic models for Health-Related Quality of Life following traumatic brain injury. Qual Life Res. 2022 Feb;31(2):451–71.

25. Neumann D, Juengst SB, Bombardier CH, Finn JA, Miles SR, Zhang Y, et al. Anxiety Trajectories the First 10 Years After a Traumatic Brain Injury (TBI): A TBI Model Systems Study. Archives of Physical Medicine and Rehabilitation. 2022 Nov 1;103(11):2105–13.

26. Stein MB, Jain S, Giacino JT, Levin H, Dikmen S, Nelson LD, et al. Risk of Posttraumatic Stress Disorder and Major Depression in Civilian Patients After Mild Traumatic Brain Injury: A TRACK-TBI Study. JAMA Psychiatry. 2019 Mar 1;76(3):249.

27. Polinder S, Cnossen MC, Real RGL, Covic A, Gorbunova A, Voormolen DC, et al. A Multidimensional Approach to Post-concussion Symptoms in Mild Traumatic Brain Injury. Front Neurol. 2018 Dec 19;9:1113.

28. Yue JK, Cnossen MC, Winkler EA, Deng H, Phelps RRL, Coss NA, et al. Pre-injury Comorbidities Are Associated With Functional Impairment and Post-concussive Symptoms at 3- and 6-Months After Mild Traumatic Brain Injury: A TRACK-TBI Study. Front Neurol. 2019 Apr 9;10:343.

29. Steyerberg EW, Wiegers E, Sewalt C, Buki A, Citerio G, De Keyser V, et al. Case-mix, care pathways, and outcomes in patients with traumatic brain injury in CENTER-TBI: a European prospective, multicentre, longitudinal, cohort study. The Lancet Neurology. 2019 Oct;18(10):923–34.

30. O'Donnell ML, Creamer M, Holmes ACN, Ellen S, McFarlane AC, Judson R, et al. Posttraumatic Stress Disorder After Injury: Does Admission to Intensive Care Unit Increase Risk? Journal of Trauma: Injury, Infection & Critical Care. 2010 Sep;69(3):627–32.

31. Zeldovich M, Wu YJ, Gorbunova A, Mikolic A, Polinder S, Plass A, et al. Influence of Sociodemographic, Premorbid, and Injury-Related Factors on Post-Concussion Symptoms after Traumatic Brain Injury. JCM. 2020 Jun 19;9(6):1931.

32. Cappa KA, Conger JC, Conger AJ. Injury severity and outcome: A meta-analysis of prospective studies on TBI outcome. Health Psychology. 2011;30(5):542–60.

33. Born K, Amsler F, Gross T. Prospective evaluation of the Quality of Life after Brain Injury (QOLIBRI) score: minor differences in patients with major versus no or mild traumatic brain injury at one-year follow up. Health Qual Life Outcomes. 2018 Dec;16(1):136.

34. Loignon A, Ouellet MC, Belleville G. A Systematic Review and Meta-analysis on PTSD Following TBI Among Military/Veteran and Civilian Populations. Journal of Head Trauma Rehabilitation. 2020 Jan;35(1):E21–35.

35. van der Horn HJ, Spikman JM, Jacobs B, van der Naalt J. Postconcussive Complaints, Anxiety, and Depression Related to Vocational Outcome in Minor to Severe Traumatic Brain Injury. Archives of Physical Medicine and Rehabilitation. 2013 May;94(5):867–74.

36. Van Praag DLG, Cnossen MC, Polinder S, Wilson L, Maas AIR. Post-Traumatic Stress Disorder after Civilian Traumatic Brain Injury: A Systematic Review and Meta-Analysis of Prevalence Rates. Journal of Neurotrauma. 2019 Dec 1;36(23):3220–32.

37. Sigurdardottir S, Andelic N, Roe C, Jerstad T, Schanke AK. Post-concussion symptoms after traumatic brain injury at 3 and 12 months post-injury: A prospective study. Brain Injury. 2009 Jan;23(6):489–97.
