# Supplementary material for: Sensitivity of outcome instruments in a priori selected patient groups after traumatic brain injury: Results from the CENTER-TBI study
Source: PLoS One. 2023 Apr 7;18(4):e0280796. doi: 10.1371/journal.pone.0280796 (PMC10081802; doi:10.1371/journal.pone.0280796)
Supplement: S2 Table — (PDF) [file pone.0280796.s002.pdf]

S2 Table. Sample characteristics per outcome instrument and time point

			**3 months**	**6 months**	**12 months**	**3, 6, and 12 months**
Instrument	Group	Level	*N* (%)	*N* (%)	*N* (%)	*N* (%)
GOSE/-Q	Sex	male	1880 (66.2%)	1828 (66.2%)	1346 (68.1%)	1107 (68.3%)
		female	962 (33.8%)	932 (33.8%)	631 (31.9%)	513 (31.7%)
	Age	< 65	2148 (75.6%)	2090 (75.7%)	1485 (75.1%)	1211 (74.8%)
		65 +	694 (24.4%)	670 (24.3%)	492 (24.9%)	409 (25.2%)
	Education	≤ primary	432 (15.2%)	430 (15.6%)	308 (15.6%)	271 (16.7%)
		secondary +	2043 (71.9%)	1958 (70.9%)	1367 (69.1%)	1128 (69.6%)
		missing	367 (12.9%)	372 (13.5%)	302 (15.3%)	221 (13.6%)
	Premorbid psych. problems	absent	1475 (51.9%)	1626 (58.9%)	1104 (55.8%)	1009 (62.3%)
		present	406 (14.3%)	449 (16.3%)	280 (14.2%)	252 (15.6%)
		missing	961 (33.8%)	685 (24.8%)	593 (30.0%)	359 (22.2%)
	Clinical care pathways	ER	548 (19.3%)	534 (19.3%)	0 (0%)[a]	0 (0%)[a]
		ward	1013 (35.6%)	983 (35.6%)	865 (43.8%)	692 (42.7%)
		ICU	1281 (45.1%)	1243 (45.0%)	1112 (56.2%)	928 (57.3%)
	Injury severity score (ISS)	<10	967 (34.0%)	953 (34.5%)	407 (20.6%)	325 (20.1%)
		10+	1854 (65.2%)	1784 (64.6%)	1545 (78.1%)	1284 (79.3%)
		missing	21 (0.7%)	23 (0.8%)	25 (1.3%)	11 (0.7%)
	TBI Severity	uncomplicated	870 (30.6%)	847 (30.7%)	408 (20.6%)	320 (19.8%)
		complicated	776 (27.3%)	781 (28.3%)	640 (32.4%)	548 (33.8%)
		moderate	183 (6.4%)	178 (6.4%)	165 (8.3%)	142 (8.8%)
		severe	426 (15.0%)	417 (15.1%)	372 (18.8%)	317 (19.6%)
		missing	587 (20.7%)	537 (19.5%)	392 (19.8%)	293 (18.1%)
		**Total**	**2842 (100%)**	**2760 (100%)**	**1977 (100%)**	**1620 (100%)**
SF-36v2	Sex	male	1330 (64.2%)	1429 (65.2%)	960 (66.8%)	683 (66.2%)
		female	743 (35.8%)	763 (34.8%)	477 (33.2%)	349 (33.8%)
	Age	< 65	1555 (75.0%)	1670 (76.2%)	1094 (76.1%)	773 (74.9%)
		65 +	518 (25.0%)	522 (23.8%)	343 (23.9%)	259 (25.1%)
	Education	≤ primary	305 (14.7%)	309 (14.1%)	195 (13.6%)	144 (14.0%)
		secondary +	1571 (75.8%)	1649 (75.2%)	1084 (75.4%)	789 (76.5%)
		missing	197 (9.5%)	234 (10.7%)	158 (11.0%)	99 (9.6%)
	Premorbid psych. problems	absent	1253 (60.4%)	1553 (70.8%)	944 (65.7%)	775 (75.1%)
		present	346 (16.7%)	426 (19.4%)	239 (16.6%)	191 (18.5%)
		missing	474 (22.9%)	213 (9.7%)	254 (17.7%)	66 (6.4%)
	Clinical care pathways	ER	470 (22.7%)	452 (20.6%)	0 (0%)[a]	0 (0%)[a]
		ward	802 (38.7%)	826 (37.7%)	686 (47.7%)	512 (49.6%)

			3 months	6 months	12 months	3, 6, and 12 months
		ICU	801 (38.6%)	914 (41.7%)	751 (52.3%)	520 (50.4%)
	Injury severity score (ISS)	<10	797 (38.4%)	807 (36.8%)	324 (22.5%)	246 (23.8%)
		10+	1260 (60.8%)	1360 (62.0%)	1090 (75.9%)	776 (75.2%)
		missing	16 (0.8%)	25 (1.1%)	23 (1.6%)	10 (1.0%)
	TBI Severity	uncomplicated	688 (33.2%)	684 (31.2%)	319 (22.2%)	223 (21.6%)
		complicated	604 (29.1%)	658 (30.0%)	485 (33.8%)	383 (37.1%)
		moderate	114 (5.5%)	132 (6.0%)	102 (7.1%)	69 (6.7%)
		severe	231 (11.1%)	282 (12.9%)	241 (16.8%)	159 (15.4%)
		missing	436 (21.0%)	436 (19.9%)	290 (20.2%)	198 (19.2%)
		**Total**	**2073 (100%)**	**2192 (100%)**	**1437 (100%)**	**1032 (100%)**
SF-12v2	Sex	male	1478 (64.5%)	1500 (65.3%)	1023 (66.6%)	762 (66.5%)
		female	815 (35.5%)	797 (34.7%)	514 (33.4%)	384 (33.5%)
	Age	< 65	1723 (75.1%)	1756 (76.4%)	1160 (75.5%)	861 (75.1%)
		65 +	570 (24.9%)	541 (23.6%)	377 (24.5%)	285 (24.9%)
	Education	≤ primary	332 (14.5%)	329 (14.3%)	210 (13.7%)	163 (14.2%)
		secondary +	1726 (75.3%)	1721 (74.9%)	1148 (74.7%)	868 (75.7%)
		missing	235 (10.2%)	247 (10.8%)	179 (11.6%)	115 (10.0%)
	Premorbid psych. problems	absent	1342 (58.5%)	1576 (68.6%)	993 (64.6%)	850 (74.2%)
		present	370 (16.1%)	434 (18.9%)	248 (16.1%)	205 (17.9%)
		missing	581 (25.3%)	287 (12.5%)	296 (19.3%)	91 (7.9%)
	Clinical care pathways	ER	501 (21.8%)	479 (20.9%)	0 (0%)[a]	0 (0%)[a]
		ward	869 (37.9%)	856 (37.3%)	717 (46.6%)	544 (47.5%)
		ICU	923 (40.3%)	962 (41.9%)	820 (53.4%)	602 (52.5%)
	Injury severity score (ISS)	<10	865 (37.7%)	842 (36.7%)	343 (22.3%)	262 (22.9%)
		10+	1409 (61.4%)	1430 (62.3%)	1171 (76.2%)	873 (76.2%)
		missing	19 (0.8%)	25 (1.1%)	23 (1.5%)	11 (1.0%)
	TBI Severity	uncomplicated	747 (32.6%)	723 (31.5%)	329 (21.4%)	239 (20.9%)
		complicated	657 (28.7%)	673 (29.3%)	513 (33.4%)	417 (36.4%)
		moderate	127 (5.5%)	142 (6.2%)	118 (7.7%)	81 (7.1%)
		severe	275 (12.0%)	301 (13.1%)	271 (17.6%)	193 (16.8%)
		missing	487 (21.2%)	458 (19.9%)	306 (19.9%)	216 (18.8%)
		**Total**	**2293 (100%)**	**2297 (100%)**	**1537 (100%)**	**1146 (100%)**
QOLIBRI	Sex	male	1367 (64.5%)	1429 (65.4%)	972 (66.6%)	702 (66.1%)
		female	751 (35.5%)	757 (34.6%)	487 (33.4%)	360 (33.9%)
	Age	< 65	1593 (75.2%)	1669 (76.3%)	1107 (75.9%)	796 (75.0%)
		65 +	525 (24.8%)	517 (23.7%)	352 (24.1%)	266 (25.0%)
	Education	≤ primary	302 (14.3%)	303 (13.9%)	196 (13.4%)	146 (13.7%)

			3 months	6 months	12 months	3, 6, and 12 months
		secondary +	1614 (76.2%)	1649 (75.4%)	1099 (75.3%)	809 (76.2%)
		missing	202 (9.5%)	234 (10.7%)	164 (11.2%)	107 (10.1%)
	Premorbid psych. problems	absent	1266 (59.8%)	1544 (70.6%)	953 (65.3%)	791 (74.5%)
		present	355 (16.8%)	424 (19.4%)	242 (16.6%)	199 (18.7%)
		missing	497 (23.5%)	218 (10.0%)	264 (18.1%)	72 (6.8%)
	Clinical care pathways	ER	474 (22.4%)	454 (20.8%)	0 (0%)[a]	0 (0%)[a]
		ward	817 (38.6%)	825 (37.7%)	694 (47.6%)	517 (48.7%)
		ICU	827 (39.0%)	907 (41.5%)	765 (52.4%)	545 (51.3%)
	Injury severity score (ISS)	<10	806 (38.1%)	803 (36.7%)	329 (22.5%)	249 (23.4%)
		10+	1297 (61.2%)	1363 (62.4%)	1106 (75.8%)	804 (75.7%)
		missing	15 (0.7%)	20 (0.9%)	24 (1.6%)	9 (0.8%)
	TBI Severity	uncomplicated	696 (32.9%)	690 (31.6%)	323 (22.1%)	222 (20.9%)
		complicated	611 (28.8%)	647 (29.6%)	491 (33.7%)	388 (36.5%)
		moderate	115 (5.4%)	130 (5.9%)	104 (7.1%)	75 (7.1%)
		severe	244 (11.5%)	284 (13.0%)	249 (17.1%)	168 (15.8%)
		missing	452 (21.3%)	435 (19.9%)	292 (20.0%)	209 (19.7%)
		**Total**	**2118 (100%)**	**2186 (100%)**	**1459 (100%)**	**1062 (100%)**
QOLIBRI-OS	Sex	male	1520 (65.0%)	1522 (65.6%)	1061 (67.0%)	796 (66.7%)
		female	820 (35.0%)	798 (34.4%)	523 (33.0%)	397 (33.3%)
	Age	< 65	1764 (75.4%)	1776 (76.6%)	1194 (75.4%)	898 (75.3%)
		65 +	576 (24.6%)	544 (23.4%)	390 (24.6%)	295 (24.7%)
	Education	≤ primary	355 (15.2%)	344 (14.8%)	233 (14.7%)	184 (15.4%)
		secondary +	1745 (74.6%)	1718 (74.1%)	1160 (73.2%)	883 (74.0%)
		missing	240 (10.3%)	258 (11.1%)	191 (12.1%)	126 (10.6%)
	Premorbid psych. problems	absent	1363 (58.2%)	1592 (68.6%)	1008 (63.6%)	877 (73.5%)
		present	371 (15.9%)	441 (19.0%)	251 (15.8%)	212 (17.8%)
		missing	606 (25.9%)	287 (12.4%)	325 (20.5%)	104 (8.7%)
	Clinical care pathways	ER	494 (21.1%)	473 (20.4%)	0 (0%)[a]	0 (0%)[a]
		ward	868 (37.1%)	865 (37.3%)	733 (46.3%)	557 (46.7%)
		ICU	978 (41.8%)	982 (42.3%)	851 (53.7%)	636 (53.3%)
	Injury severity score (ISS)	<10	855 (36.5%)	837 (36.1%)	350 (22.1%)	266 (22.3%)
		10+	1467 (62.7%)	1459 (62.9%)	1210 (76.4%)	916 (76.8%)
		missing	18 (0.8%)	24 (1.0%)	24 (1.5%)	11 (0.9%)
	TBI Severity	uncomplicated	745 (31.8%)	731 (31.5%)	342 (21.6%)	249 (20.9%)
		complicated	660 (28.2%)	680 (29.3%)	525 (33.1%)	424 (35.5%)
		moderate	133 (5.7%)	143 (6.2%)	120 (7.6%)	87 (7.3%)
		severe	308 (13.2%)	311 (13.4%)	288 (18.2%)	211 (17.7%)

			3 months	6 months	12 months	3, 6, and 12 months
		missing	494 (21.1%)	455 (19.6%)	309 (19.5%)	222 (18.6%)
		**Total**	**2340 (100%)**	**2320 (100%)**	**1584 (100%)**	**1193 (100%)**
GAD-7	Sex	male	1344 (64.4%)	1427 (65.4%)	978 (66.8%)	704 (66.6%)
		female	744 (35.6%)	754 (34.6%)	487 (33.2%)	353 (33.4%)
	Age	< 65	1564 (74.9%)	1666 (76.4%)	1108 (75.6%)	791 (74.8%)
		65 +	524 (25.1%)	515 (23.6%)	357 (24.4%)	266 (25.2%)
	Education	≤ primary	299 (14.3%)	305 (14.0%)	196 (13.4%)	145 (13.7%)
		secondary +	1590 (76.1%)	1645 (75.4%)	1108 (75.6%)	811 (76.7%)
		missing	199 (9.5%)	231 (10.6%)	161 (11.0%)	101 (9.6%)
	Premorbid psych. problems	absent	1259 (60.3%)	1550 (71.1%)	963 (65.7%)	794 (75.1%)
		present	347 (16.6%)	429 (19.7%)	241 (16.5%)	197 (18.6%)
		missing	482 (23.1%)	202 (9.3%)	261 (17.8%)	66 (6.2%)
	Clinical care pathways	ER	463 (22.2%)	445 (20.4%)	0 (0%)[a]	0 (0%)[a]
		ward	801 (38.4%)	820 (37.6%)	691 (47.2%)	513 (48.5%)
		ICU	824 (39.5%)	916 (42.0%)	774 (52.8%)	544 (51.5%)
	Injury severity score (ISS)	<10	790 (37.8%)	795 (36.5%)	328 (22.4%)	245 (23.2%)
		10+	1283 (61.4%)	1363 (62.5%)	1115 (76.1%)	804 (76.1%)
		missing	15 (0.7%)	23 (1.1%)	22 (1.5%)	8 (0.8%)
	TBI Severity	uncomplicated	682 (32.7%)	678 (31.1%)	317 (21.6%)	222 (21.0%)
		complicated	608 (29.1%)	649 (29.8%)	495 (33.8%)	389 (36.8%)
		moderate	114 (5.5%)	137 (6.3%)	105 (7.2%)	73 (6.9%)
		severe	241 (11.5%)	285 (13.1%)	252 (17.2%)	169 (16.0%)
		missing	443 (21.2%)	432 (19.8%)	296 (20.2%)	204 (19.3%)
		**Total**	**2088 (100%)**	**2181 (100%)**	**1465 (100%)**	**1057 (100%)**
PHQ-9	Sex	male	1349 (64.4%)	1426 (65.2%)	973 (66.9%)	703 (66.4%)
		female	746 (35.6%)	760 (34.8%)	482 (33.1%)	355 (33.6%)
	Age	< 65	1571 (75.0%)	1667 (76.3%)	1100 (75.6%)	794 (75.0%)
		65 +	524 (25.0%)	519 (23.7%)	355 (24.4%)	264 (25.0%)
	Education	≤ primary	303 (14.5%)	308 (14.1%)	194 (13.3%)	149 (14.1%)
		secondary +	1592 (76.0%)	1646 (75.3%)	1103 (75.8%)	811 (76.7%)
		missing	200 (9.5%)	232 (10.6%)	158 (10.9%)	98 (9.3%)
	Premorbid psych. problems	absent	1263 (60.3%)	1555 (71.1%)	961 (66.0%)	796 (75.2%)
		present	351 (16.8%)	426 (19.5%)	240 (16.5%)	195 (18.4%)
		missing	481 (23.0%)	205 (9.4%)	254 (17.5%)	67 (6.3%)
	Clinical care pathways	ER	467 (22.3%)	447 (20.4%)	0 (0%)[a]	0 (0%)[a]
		ward	805 (38.4%)	823 (37.6%)	688 (47.3%)	515 (48.7%)
		ICU	823 (39.3%)	916 (41.9%)	767 (52.7%)	543 (51.3%)

			3 months	6 months	12 months	3, 6, and 12 months
	Injury severity score (ISS)	<10	794 (37.9%)	797 (36.5%)	324 (22.3%)	246 (23.3%)
		10+	1286 (61.4%)	1365 (62.4%)	1109 (76.2%)	803 (75.9%)
		missing	15 (0.7%)	24 (1.1%)	22 (1.5%)	9 (0.9%)
	TBI Severity	uncomplicated	686 (32.7%)	684 (31.3%)	314 (21.6%)	224 (21.2%)
		complicated	609 (29.1%)	648 (29.6%)	491 (33.7%)	386 (36.5%)
		moderate	115 (5.5%)	137 (6.3%)	106 (7.3%)	77 (7.3%)
		severe	242 (11.6%)	285 (13.0%)	250 (17.2%)	167 (15.8%)
		missing	443 (21.1%)	432 (19.8%)	294 (20.2%)	204 (19.3%)
		**Total**	**2095 (100%)**	**2186 (100%)**	**1455 (100%)**	**1058 (100%)**
PCL-5	Sex	male	1341 (64.4%)	1412 (65.0%)	966 (66.9%)	701 (66.8%)
		female	741 (35.6%)	759 (35.0%)	478 (33.1%)	348 (33.2%)
	Age	< 65	1561 (75.0%)	1656 (76.3%)	1095 (75.8%)	784 (74.7%)
		65 +	521 (25.0%)	515 (23.7%)	349 (24.2%)	265 (25.3%)
	Education	≤ primary	295 (14.2%)	306 (14.1%)	195 (13.5%)	146 (13.9%)
		secondary +	1587 (76.2%)	1640 (75.5%)	1088 (75.3%)	800 (76.3%)
		missing	200 (9.6%)	225 (10.4%)	161 (11.1%)	103 (9.8%)
	Premorbid psych. problems	absent	1254 (60.2%)	1547 (71.3%)	955 (66.1%)	790 (75.3%)
		present	353 (17.0%)	424 (19.5%)	235 (16.3%)	193 (18.4%)
		missing	475 (22.8%)	200 (9.2%)	254 (17.6%)	66 (6.3%)
	Clinical care pathways	ER	472 (22.7%)	455 (21.0%)	0 (0%)[a]	0 (0%)[a]
		ward	802 (38.5%)	817 (37.6%)	685 (47.4%)	511 (48.7%)
		ICU	808 (38.8%)	899 (41.4%)	759 (52.6%)	538 (51.3%)
	Injury severity score (ISS)	<10	797 (38.3%)	802 (36.9%)	322 (22.3%)	240 (22.9%)
		10+	1270 (61.0%)	1347 (62.0%)	1099 (76.1%)	800 (76.3%)
		missing	15 (0.7%)	22 (1.0%)	23 (1.6%)	9 (0.9%)
	TBI Severity	uncomplicated	681 (32.7%)	684 (31.5%)	316 (21.9%)	222 (21.2%)
		complicated	606 (29.1%)	646 (29.8%)	489 (33.9%)	381 (36.3%)
		moderate	112 (5.4%)	131 (6.0%)	106 (7.3%)	78 (7.4%)
		severe	243 (11.7%)	276 (12.7%)	244 (16.9%)	165 (15.7%)
		missing	440 (21.1%)	434 (20.0%)	289 (20.0%)	203 (19.4%)
		**Total**	**2082 (100%)**	**2171 (100%)**	**1444 (100%)**	**1049 (100%)**
RPQ	Sex	male	1396 (64.7%)	1474 (65.3%)	1009 (66.9%)	742 (67.3%)
		female	763 (35.3%)	783 (34.7%)	499 (33.1%)	361 (32.7%)
	Age	< 65	1625 (75.3%)	1727 (76.5%)	1144 (75.9%)	837 (75.9%)
		65 +	534 (24.7%)	530 (23.5%)	364 (24.1%)	266 (24.1%)
	Education	≤ primary	323 (15.0%)	335 (14.8%)	211 (14.0%)	169 (15.3%)
		secondary +	1627 (75.4%)	1679 (74.4%)	1124 (74.5%)	827 (75.0%)

			**3 months**	**6 months**	**12 months**	**3, 6, and 12 months**
		missing	209 (9.7%)	243 (10.8%)	173 (11.5%)	107 (9.7%)
	Premorbid psych. problems	absent	1287 (59.6%)	1569 (69.5%)	976 (64.7%)	818 (74.2%)
		present	352 (16.3%)	433 (19.2%)	242 (16.0%)	197 (17.9%)
		missing	520 (24.1%)	255 (11.3%)	290 (19.2%)	88 (8.0%)
	Clinical care pathways	ER	480 (22.2%)	466 (20.6%)	0 (0%)[a]	0 (0%)[a]
		ward	834 (38.6%)	851 (37.7%)	713 (47.3%)	541 (49.0%)
		ICU	845 (39.1%)	940 (41.6%)	795 (52.7%)	562 (51.0%)
	Injury severity score (ISS)	<10	823 (38.1%)	826 (36.6%)	340 (22.5%)	257 (23.3%)
		10+	1321 (61.2%)	1407 (62.3%)	1146 (76.0%)	837 (75.9%)
		missing	15 (0.7%)	24 (1.1%)	22 (1.5%)	9 (0.8%)
	TBI Severity	uncomplicated	712 (33.0%)	719 (31.9%)	336 (22.3%)	243 (22.0%)
		complicated	626 (29.0%)	668 (29.6%)	507 (33.6%)	397 (36.0%)
		moderate	118 (5.5%)	140 (6.2%)	109 (7.2%)	78 (7.1%)
		severe	250 (11.6%)	291 (12.9%)	261 (17.3%)	178 (16.1%)
		missing	453 (21.0%)	439 (19.5%)	295 (19.6%)	207 (18.8%)
		**Total**	**2159 (100%)**	**2257 (100%)**	**1508 (100%)**	**1103 (100%)**

ER = emergency room; ICU = intensive care unit; TBI = traumatic brain injury; *N* = number of observations; % = percentage: GOSE/-Q = Combined information on recovery status using the Glasgow Outcome Scale – Extended and its questionnaire version; SF-36v2 = 36-item Short Form Health Survey – version 2; SF-12v2 = 12-Item Short Form Survey – version 2; PCS = Physical Component Summary Score, MCS = Mental Component Summary Score; QOLIBRI = Quality of Life after Traumatic Brain Injury; QOLIBRI-OS = Quality of Life after Traumatic Brain Injury – Overall Scale; GAD-7 = Generalized Anxiety Disorder-7; PHQ-9 = Patient Health Questionnaire-9; PCL-5 = Posttraumatic Stress Disorder Checklist for DSM-5; RPQ = Rivermead Post-Concussion Symptoms Questionnaire.

[a] Based on the study design, participants seen in the emergency room (ER) and then discharged were not included in the 12-month follow-up assessments.
