# Supplementary material for: Sensitivity of outcome instruments in a priori selected patient groups after traumatic brain injury: Results from the CENTER-TBI study
Source: PLoS One. 2023 Apr 7;18(4):e0280796. doi: 10.1371/journal.pone.0280796 (PMC10081802; doi:10.1371/journal.pone.0280796)
Supplement: S3 Table — (PDF) [file pone.0280796.s003.pdf]

S3 Table. Sensitivity of the outcome instruments to the pairwise group comparisons

	**Three months**	**Six months**	**Twelve months**	
Data as available	n = 42	n = 41	n = 41	Average
GOSE	**79%**	**81%**	**78%**	**79%**
SF-36v2 PCS	38%	24%	17%	26%
SF-12v2 PCS	**45%**	33%	34%	38%
SF-36v2 MCS	26%	21%	**41%**	30%
SF-12v2 MCS	29%	19%	34%	27%
QOLIBRI	36%	38%	**51%**	**42%**
QOLIBRI-OS	29%	**40%**	**41%**	37%
GAD-7	21%	21%	34%	26%
PHQ-9	33%	29%	**41%**	34%
PCL-5	33%	26%	37%	32%
RPQ	**50%**	**52%**	39%	**47%**
Completers' data	n = 41	n = 41	n = 41	Average
GOSE	**76%**	**78%**	**76%**	**76%**
SF-36v2 PCS	44%	34%	17%	32%
SF-12v2 PCS	39%	**37%**	34%	37%
SF-36v2 MCS	39%	**37%**	**44%**	40%
SF-12v2 MCS	39%	22%	34%	32%
QOLIBRI	**46%**	**37%**	**56%**	**46%**
QOLIBRI-OS	32%	**37%**	37%	35%
GAD-7	24%	34%	**44%**	34%
PHQ-9	**46%**	**37%**	**44%**	**42%**
PCL-5	24%	29%	39%	31%
RPQ	**46%**	**37%**	37%	40%

n = number of pairwise comparisons, % = percentage, average = average relative frequencies from 3 to 12 months. **Bold** values indicate top three instruments with the highest sensitivity (i.e., in most group comparisons). GOSE/-Q = Combined information on recovery status using the Glasgow Outcome Scale – Extended and its questionnaire version; SF-36v2 = 36-item Short Form Health Survey – version 2; SF-12v2 = 12-Item Short Form Survey – version 2; PCS = Physical Component Summary Score, MCS = Mental Component Summary Score; QOLIBRI = Quality of Life after Traumatic Brain Injury; QOLIBRI-OS = Quality of Life after Traumatic Brain Injury – Overall Scale; GAD-7 = Generalized Anxiety Disorder-7; PHQ-9 = Patient Health Questionnaire-9; PCL-5 = Posttraumatic Stress Disorder Checklist for DSM-5; RPQ = Rivermead Post-Concussion Symptoms Questionnaire.
