# Supplementary material for: Sensitivity of outcome instruments in a priori selected patient groups after traumatic brain injury: Results from the CENTER-TBI study
Source: PLoS One. 2023 Apr 7;18(4):e0280796. doi: 10.1371/journal.pone.0280796 (PMC10081802; doi:10.1371/journal.pone.0280796)
Supplement: S4 Table — (PDF) [file pone.0280796.s004.pdf]

**Mann-Whitney effect size**
**3 months after TBI (data as available)**

| No. | Variable | Groups | GOSE/-Q | SF-36v2 PCS | SF-12v2 PCS | SF-36v2 MCS | SF-12v2 MCS | QOLIBRI | QOLIBRI-OS | GAD-7 | PHQ-9 | PCL-5 | RPQ |
|---|---|---|---|---|---|---|---|---|---|---|---|---|---|
| 1 | | male \| TBI Sev 1 vs. female \| TBI Sev 1 | 0.46 | 0.43 | 0.42 | 0.43 | 0.43 | 0.45 | 0.45 | 0.46 | 0.42 | 0.45 | 0.40 |
| 2 | | male \| TBI Sev 2 vs. female \| TBI Sev 2 | 0.47 | 0.43 | 0.43 | 0.44 | 0.43 | 0.46 | 0.48 | 0.42 | 0.43 | 0.43 | 0.46 |
| 3 | | male \| TBI Sev 3 vs. female \| TBI Sev 3 | 0.53 | 0.48 | 0.50 | 0.52 | 0.46 | 0.47 | 0.52 | 0.42 | 0.48 | 0.44 | 0.41 |
| 4 | | male \| TBI Sev 4 vs. female \| TBI Sev 4 | 0.46 | 0.47 | 0.45 | 0.45 | 0.43 | 0.46 | 0.50 | 0.42 | 0.43 | 0.42 | 0.42 |
| 5 | Sex | male \| TBI Sev 1 vs. male \| TBI Sev 2 | 0.35 | 0.44 | 0.42 | 0.45 | 0.45 | 0.43 | 0.45 | 0.49 | 0.46 | 0.47 | 0.40 |
| 6 | | male \| TBI Sev 2 vs. male \| TBI Sev 3 | 0.33 | 0.46 | 0.43 | 0.47 | 0.48 | 0.49 | 0.47 | 0.49 | 0.48 | 0.50 | 0.48 |
| 7 | | male \| TBI Sev 3 vs. male \| TBI Sev 4 | 0.41 | 0.41 | 0.44 | 0.47 | 0.48 | 0.41 | 0.41 | 0.45 | 0.44 | 0.44 | 0.43 |
| 8 | | female \| TBI Sev 1 vs. female \| TBI Sev 2 | 0.37 | 0.45 | 0.44 | 0.47 | 0.45 | 0.45 | 0.48 | 0.45 | 0.47 | 0.46 | 0.46 |
| 9 | | female \| TBI Sev 2 vs. female \| TBI Sev 3 | 0.38 | 0.50 | 0.51 | 0.54 | 0.51 | 0.51 | 0.51 | 0.52 | 0.55 | 0.51 | 0.44 |
| 10 | | female \| TBI Sev 3 vs. female \| TBI Sev 4 | 0.33 | 0.40 | 0.40 | 0.39 | 0.45 | 0.40 | 0.39 | 0.45 | 0.38 | 0.43 | 0.43 |
| 11 | | <65 \| TBI Sev 2 vs. 65+ \| TBI Sev 2 | 0.49 | 0.41 | 0.41 | 0.59 | 0.55 | 0.51 | 0.50 | 0.58 | 0.56 | 0.59 | 0.59 |
| 12 | | <65 \| TBI Sev 1 vs. <65 \| TBI Sev 2 | 0.36 | 0.44 | 0.42 | 0.44 | 0.45 | 0.43 | 0.45 | 0.46 | 0.46 | 0.46 | 0.40 |
| 13 | Age | <65 \| TBI Sev 2 vs. <65 \| TBI Sev 3 | 0.34 | 0.46 | 0.46 | 0.53 | 0.52 | 0.51 | 0.51 | 0.54 | 0.53 | 0.53 | 0.49 |
| 14 | | <65 \| TBI Sev 3 vs. <65 \| TBI Sev 4 | 0.37 | 0.40 | 0.41 | 0.43 | 0.47 | 0.40 | 0.38 | 0.43 | 0.41 | 0.43 | 0.43 |
| 15 | | 65+ \| TBI Sev 1 vs. 65+ \| TBI Sev 2 | 0.35 | 0.53 | 0.51 | 0.48 | 0.44 | 0.46 | 0.49 | 0.49 | 0.47 | 0.46 | 0.45 |
| 16 | | primary and less \| TBI Sev 1 vs. secondary+ \| TBI Sev 1 | 0.54 | 0.61 | 0.60 | 0.55 | 0.53 | 0.61 | 0.58 | 0.56 | 0.54 | 0.50 | 0.53 |
| 17 | | primary and less \| TBI Sev 1 vs. primary and less \| TBI Sev 2 | 0.41 | 0.50 | 0.47 | 0.48 | 0.48 | 0.46 | 0.51 | 0.49 | 0.49 | 0.44 | 0.44 |
| 18 | Education | secondary+ \| TBI Sev 1 vs. secondary+ \| TBI Sev 2 | 0.36 | 0.45 | 0.44 | 0.46 | 0.46 | 0.45 | 0.47 | 0.48 | 0.48 | 0.48 | 0.43 |
| 19 | | secondary+ \| TBI Sev 2 vs. secondary+ \| TBI Sev 3 | 0.33 | 0.47 | 0.45 | 0.49 | 0.49 | 0.48 | 0.48 | 0.49 | 0.51 | 0.49 | 0.47 |
| 20 | | secondary+ \| TBI Sev 3 vs. secondary+ \| TBI Sev 4 | 0.39 | 0.38 | 0.40 | 0.44 | 0.48 | 0.39 | 0.38 | 0.46 | 0.39 | 0.46 | 0.41 |
| 21 | | absent \| TBI Sev 2 vs. present \| TBI Sev 2 | 0.46 | 0.46 | 0.47 | 0.31 | 0.31 | 0.33 | 0.37 | 0.30 | 0.30 | 0.35 | 0.39 |
| 22 | | absent \| TBI Sev 4 vs. present \| TBI Sev 4 | 0.46 | 0.49 | 0.48 | 0.36 | 0.39 | 0.43 | 0.39 | 0.34 | 0.38 | 0.41 | 0.40 |
| 23 | Premorbid psych. problems | absent \| TBI Sev 1 vs. absent \| TBI Sev 2 | 0.36 | 0.45 | 0.44 | 0.43 | 0.43 | 0.43 | 0.45 | 0.47 | 0.47 | 0.47 | 0.41 |
| 24 | | absent \| TBI Sev 2 vs. absent \| TBI Sev 3 | 0.34 | 0.46 | 0.44 | 0.48 | 0.49 | 0.50 | 0.48 | 0.47 | 0.48 | 0.49 | 0.47 |
| 25 | | absent \| TBI Sev 3 vs. absent \| TBI Sev 4 | 0.42 | 0.43 | 0.46 | 0.45 | 0.47 | 0.40 | 0.43 | 0.46 | 0.43 | 0.44 | 0.43 |
| 26 | | present \| TBI Sev 1 vs. present \| TBI Sev 2 | 0.37 | 0.45 | 0.44 | 0.43 | 0.43 | 0.40 | 0.45 | 0.47 | 0.47 | 0.46 | 0.44 |
| 27 | | ER \| TBI Sev 1 vs. admission \| TBI Sev 1 | 0.42 | 0.44 | 0.45 | 0.50 | 0.52 | 0.48 | 0.48 | 0.49 | 0.50 | 0.47 | 0.45 |
| 28 | | ER \| TBI Sev 1 vs. admission \| TBI Sev 1 | 0.31 | 0.29 | 0.28 | 0.52 | 0.51 | 0.43 | 0.43 | 0.43 | 0.40 | 0.38 | 0.36 |
| 29 | | ER \| TBI Sev 2 vs. admission \| TBI Sev 2 | 0.38 | 0.45 | 0.46 | 0.50 | 0.48 | 0.50 | 0.54 | 0.47 | 0.50 | 0.44 | 0.41 |
| 30 | | ER \| TBI Sev 2 vs. admission \| TBI Sev 2 | 0.26 | 0.41 | 0.43 | 0.44 | 0.42 | 0.45 | 0.50 | 0.43 | 0.47 | 0.37 | 0.36 |
| 31 | Clinical care pathways | admission \| TBI Sev 2 vs. ICU \| TBI Sev 2 | 0.36 | 0.46 | 0.47 | 0.44 | 0.44 | 0.46 | 0.46 | 0.47 | 0.47 | 0.44 | 0.46 |
| 32 | | ER \| TBI Sev 1 vs. ER \| TBI Sev 2 | 0.48 | 0.45 | 0.44 | 0.48 | 0.51 | 0.44 | 0.43 | 0.51 | 0.47 | 0.52 | 0.50 |
| 33 | | admission \| TBI Sev 1 vs. admission \| TBI Sev 2 | 0.43 | 0.47 | 0.45 | 0.48 | 0.47 | 0.46 | 0.48 | 0.49 | 0.47 | 0.50 | 0.45 |
| 34 | | ICU \| TBI Sev 1 vs. ICU \| TBI Sev 2 | 0.43 | 0.59 | 0.61 | 0.42 | 0.42 | 0.46 | 0.50 | 0.51 | 0.54 | 0.51 | 0.47 |
| 35 | | ICU \| TBI Sev 2 vs. ICU \| TBI Sev 3 | 0.38 | 0.46 | 0.45 | 0.50 | 0.49 | 0.48 | 0.48 | 0.49 | 0.51 | 0.51 | 0.46 |
| 36 | | ICU \| TBI Sev 3 vs. ICU \| TBI Sev 4 | 0.42 | 0.45 | 0.45 | 0.47 | 0.51 | 0.45 | 0.42 | 0.49 | 0.44 | 0.49 | 0.47 |
| 37 | | ISS<10 \| TBI Sev 1 vs. ISS10+ \| TBI Sev 1 | 0.39 | 0.41 | 0.40 | 0.52 | 0.52 | 0.47 | 0.46 | 0.48 | 0.49 | 0.44 | 0.44 |
| 38 | | ISS<10 \| TBI Sev 2 vs. ISS10+ \| TBI Sev 2 | 0.36 | 0.43 | 0.43 | 0.45 | 0.43 | 0.48 | 0.46 | 0.47 | 0.46 | 0.44 | 0.46 |
| 39 | Injury severity score | ISS<10 \| TBI Sev 1 vs. ISS<10 \| TBI Sev 2 | 0.41 | 0.46 | 0.45 | 0.50 | 0.51 | 0.44 | 0.47 | 0.49 | 0.49 | 0.49 | 0.43 |
| 40 | | ISS10+ \| TBI Sev 1 vs. ISS10+ \| TBI Sev 2 | 0.39 | 0.48 | 0.48 | 0.43 | 0.43 | 0.45 | 0.48 | 0.48 | 0.47 | 0.48 | 0.44 |
| 41 | | ISS10+ \| TBI Sev 2 vs. ISS10+ \| TBI Sev 3 | 0.36 | 0.48 | 0.47 | 0.49 | 0.49 | 0.49 | 0.48 | 0.49 | 0.51 | 0.50 | 0.46 |
| 42 | | ISS10+ \| TBI Sev 3 vs. ISS10+ \| TBI Sev 4 | 0.40 | 0.42 | 0.44 | 0.46 | 0.50 | 0.42 | 0.41 | 0.48 | 0.43 | 0.46 | 0.45 |

*Note*. No. = continuous numbering of comparisons; TBI Sev 1 = uncomplicated mild TBI, TBI Sev 2 = complicated mild TBI, TBI Sev 3 = moderate TBI, TBI Sev 4 = severe TBI; ER = emergency room, admission = admission to a hospital ward, ICU = intensive care unit; missing pairwise comparisons indicate insufficient (n ≤ 28) number of participants; dark red cells = more than a medium-sized effect (< 0.36 | > 0.64 ), light red cells = more than a small, but less than a medium effect (0.36 - 0.43 | 0.64 - 0.55), white cells = small effect (0.44 - 0.51 | 0.51 - 0.56). Values below 0.50 indicate for a better outcome in the patient group 1 compared to the group 2. Rounding errors may occur on the second decimal place.

**<u>Interpretation example:</u>** Three months after TBI, the instruments measuring disease-specific (QOLIBRI/-OS) and mental HRQOL (SF-36v2/-12v2) as well as psychological outcomes (GAD-7, PHQ-9, PCL-5, RPQ) are moderately to highly sensitive for detecting differences between individuals with and without premorbid mental health problems, especially within the groups after complicated mTBI and severe TBI. Since individuals with premorbid mental health problems have significantly worse outcomes in these areas, it is recommended to apply these instruments for identifying potential problems and finding appropriate treatment.

**6 months after TBI**

| No. | Variable | Groups | GOSE/-Q | SF-36v2 PCS | SF-12v2 PCS | SF-36v2 MCS | SF-12v2 MCS | QOLIBRI | QOLIBRI-OS | GAD-7 | PHQ-9 | PCL-5 | RPQ |
|---|---|---|---|---|---|---|---|---|---|---|---|---|---|
| 1 | Sex | male \| TBI Sev 1 vs. female \| TBI Sev 1 | 0.44 | 0.42 | 0.44 | 0.43 | 0.43 | 0.44 | 0.43 | 0.41 | 0.41 | 0.44 | 0.41 |
| 2 | | male \| TBI Sev 2 vs. female \| TBI Sev 2 | 0.43 | 0.41 | 0.40 | 0.45 | 0.45 | 0.48 | 0.46 | 0.44 | 0.43 | 0.48 | 0.44 |
| 3 | | male \| TBI Sev 3 vs. female \| TBI Sev 3 | 0.57 | 0.49 | 0.48 | 0.47 | 0.49 | 0.50 | 0.52 | 0.46 | 0.50 | 0.44 | 0.44 |
| 4 | | male \| TBI Sev 4 vs. female \| TBI Sev 4 | 0.45 | 0.47 | 0.47 | 0.42 | 0.42 | 0.44 | 0.48 | 0.43 | 0.44 | 0.41 | 0.41 |
| 5 | | male \| TBI Sev 1 vs. male \| TBI Sev 2 | 0.37 | 0.46 | 0.45 | 0.45 | 0.45 | 0.42 | 0.43 | 0.45 | 0.45 | 0.44 | 0.38 |
| 6 | | male \| TBI Sev 2 vs. male \| TBI Sev 3 | 0.33 | 0.45 | 0.46 | 0.47 | 0.49 | 0.48 | 0.52 | 0.45 | 0.46 | 0.48 | 0.47 |
| 7 | | male \| TBI Sev 3 vs. male \| TBI Sev 4 | 0.42 | 0.45 | 0.43 | 0.47 | 0.47 | 0.46 | 0.42 | 0.51 | 0.48 | 0.49 | 0.46 |
| 8 | | female \| TBI Sev 1 vs. female \| TBI Sev 2 | 0.35 | 0.44 | 0.43 | 0.47 | 0.47 | 0.46 | 0.47 | 0.48 | 0.47 | 0.48 | 0.43 |
| 9 | | female \| TBI Sev 2 vs. female \| TBI Sev 3 | 0.46 | 0.54 | 0.55 | 0.49 | 0.53 | 0.51 | 0.58 | 0.49 | 0.53 | 0.43 | 0.45 |
| 10 | | female \| TBI Sev 3 vs. female \| TBI Sev 4 | 0.31 | 0.42 | 0.41 | 0.42 | 0.40 | 0.40 | 0.38 | 0.48 | 0.43 | 0.46 | 0.46 |
| 11 | Age | <65 \| TBI Sev 2 vs. 65+ \| TBI Sev 2 | 0.48 | 0.36 | 0.34 | 0.55 | 0.54 | 0.48 | 0.46 | 0.56 | 0.55 | 0.61 | 0.57 |
| 12 | | <65 \| TBI Sev 1 vs. <65 \| TBI Sev 2 | 0.36 | 0.46 | 0.45 | 0.45 | 0.45 | 0.44 | 0.45 | 0.46 | 0.45 | 0.43 | 0.39 |
| 13 | | <65 \| TBI Sev 2 vs. <65 \| TBI Sev 3 | 0.36 | 0.47 | 0.47 | 0.49 | 0.51 | 0.49 | 0.53 | 0.47 | 0.50 | 0.48 | 0.48 |
| 14 | | <65 \| TBI Sev 3 vs. <65 \| TBI Sev 4 | 0.37 | 0.41 | 0.40 | 0.46 | 0.45 | 0.43 | 0.41 | 0.50 | 0.45 | 0.49 | 0.47 |
| 15 | | 65+ \| TBI Sev 1 vs. 65+ \| TBI Sev 2 | 0.38 | 0.49 | 0.49 | 0.46 | 0.45 | 0.45 | 0.46 | 0.45 | 0.46 | 0.47 | 0.42 |
| 16 | Education | primary and less \| TBI Sev 1 vs. secondary+ \| TBI Sev 1 | 0.52 | 0.59 | 0.62 | 0.56 | 0.55 | 0.60 | 0.55 | 0.58 | 0.56 | 0.52 | 0.51 |
| 17 | | primary and less \| TBI Sev 1 vs. primary and less \| TBI Sev 2 | 0.40 | 0.48 | 0.50 | 0.50 | 0.50 | 0.47 | 0.47 | 0.52 | 0.51 | 0.48 | 0.43 |
| 18 | | secondary+ \| TBI Sev 1 vs. secondary+ \| TBI Sev 2 | 0.37 | 0.46 | 0.45 | 0.47 | 0.46 | 0.44 | 0.46 | 0.46 | 0.47 | 0.45 | 0.39 |
| 19 | | secondary+ \| TBI Sev 2 vs. secondary+ \| TBI Sev 3 | 0.36 | 0.46 | 0.47 | 0.47 | 0.50 | 0.48 | 0.52 | 0.45 | 0.47 | 0.45 | 0.49 |
| 20 | | secondary+ \| TBI Sev 3 vs. secondary+ \| TBI Sev 4 | 0.40 | 0.45 | 0.43 | 0.47 | 0.46 | 0.44 | 0.41 | 0.52 | 0.47 | 0.52 | 0.45 |
| 21 | Premorbid psych. problems | absent \| TBI Sev 2 vs. present \| TBI Sev 2 | 0.47 | 0.46 | 0.48 | 0.35 | 0.34 | 0.36 | 0.39 | 0.34 | 0.35 | 0.35 | 0.41 |
| 22 | | absent \| TBI Sev 4 vs. present \| TBI Sev 4 | 0.48 | 0.41 | 0.44 | 0.41 | 0.39 | 0.37 | 0.38 | 0.32 | 0.29 | 0.32 | 0.32 |
| 23 | | absent \| TBI Sev 1 vs. absent \| TBI Sev 2 | 0.35 | 0.45 | 0.44 | 0.43 | 0.43 | 0.41 | 0.42 | 0.45 | 0.44 | 0.45 | 0.38 |
| 24 | | absent \| TBI Sev 2 vs. absent \| TBI Sev 3 | 0.39 | 0.50 | 0.49 | 0.48 | 0.49 | 0.51 | 0.53 | 0.44 | 0.48 | 0.46 | 0.46 |
| 25 | | absent \| TBI Sev 3 vs. absent \| TBI Sev 4 | 0.40 | 0.45 | 0.44 | 0.45 | 0.46 | 0.43 | 0.42 | 0.52 | 0.48 | 0.49 | 0.49 |
| 26 | | present \| TBI Sev 1 vs. present \| TBI Sev 2 | 0.38 | 0.44 | 0.43 | 0.50 | 0.47 | 0.46 | 0.48 | 0.49 | 0.49 | 0.43 | 0.46 |
| 27 | Clinical care pathways | ER \| TBI Sev 1 vs. admission \| TBI Sev 1 | 0.44 | 0.50 | 0.51 | 0.51 | 0.50 | 0.50 | 0.49 | 0.50 | 0.53 | 0.46 | 0.46 |
| 28 | | ER \| TBI Sev 1 vs. admission \| TBI Sev 1 | 0.34 | 0.36 | 0.38 | 0.47 | 0.44 | 0.45 | 0.48 | 0.49 | 0.49 | 0.41 | 0.43 |
| 29 | | ER \| TBI Sev 2 vs. admission \| TBI Sev 2 | 0.38 | 0.43 | 0.44 | 0.50 | 0.49 | 0.46 | 0.50 | 0.49 | 0.44 | 0.46 | 0.41 |
| 30 | | ER \| TBI Sev 2 vs. admission \| TBI Sev 2 | 0.28 | 0.39 | 0.41 | 0.40 | 0.41 | 0.39 | 0.44 | 0.42 | 0.37 | 0.36 | 0.32 |
| 31 | | admission \| TBI Sev 2 vs. ICU \| TBI Sev 2 | 0.39 | 0.46 | 0.46 | 0.41 | 0.43 | 0.43 | 0.44 | 0.42 | 0.43 | 0.41 | 0.41 |
| 32 | | ER \| TBI Sev 1 vs. ER \| TBI Sev 2 | 0.47 | 0.52 | 0.51 | 0.50 | 0.48 | 0.49 | 0.47 | 0.50 | 0.55 | 0.50 | 0.48 |
| 33 | | admission \| TBI Sev 1 vs. admission \| TBI Sev 2 | 0.42 | 0.45 | 0.44 | 0.49 | 0.48 | 0.46 | 0.47 | 0.50 | 0.46 | 0.49 | 0.44 |
| 34 | | ICU \| TBI Sev 1 vs. ICU \| TBI Sev 2 | 0.40 | 0.55 | 0.53 | 0.43 | 0.45 | 0.44 | 0.43 | 0.42 | 0.44 | 0.46 | 0.38 |
| 35 | | ICU \| TBI Sev 2 vs. ICU \| TBI Sev 3 | 0.41 | 0.48 | 0.50 | 0.53 | 0.54 | 0.51 | 0.56 | 0.50 | 0.52 | 0.50 | 0.51 |
| 36 | | ICU \| TBI Sev 3 vs. ICU \| TBI Sev 4 | 0.40 | 0.47 | 0.45 | 0.47 | 0.46 | 0.46 | 0.41 | 0.52 | 0.47 | 0.51 | 0.49 |
| 37 | Injury severity score | ISS<10 \| TBI Sev 1 vs. ISS10+ \| TBI Sev 1 | 0.41 | 0.46 | 0.47 | 0.50 | 0.49 | 0.50 | 0.50 | 0.50 | 0.51 | 0.47 | 0.46 |
| 38 | | ISS<10 \| TBI Sev 2 vs. ISS10+ \| TBI Sev 2 | 0.40 | 0.44 | 0.44 | 0.46 | 0.46 | 0.48 | 0.49 | 0.48 | 0.48 | 0.46 | 0.43 |
| 39 | | ISS<10 \| TBI Sev 1 vs. ISS<10 \| TBI Sev 2 | 0.39 | 0.48 | 0.47 | 0.49 | 0.48 | 0.45 | 0.46 | 0.48 | 0.48 | 0.47 | 0.43 |
| 40 | | ISS10+ \| TBI Sev 1 vs. ISS10+ \| TBI Sev 2 | 0.39 | 0.46 | 0.46 | 0.45 | 0.45 | 0.43 | 0.44 | 0.46 | 0.45 | 0.46 | 0.40 |
| 41 | | ISS10+ \| TBI Sev 2 vs. ISS10+ \| TBI Sev 3 | 0.38 | 0.48 | 0.49 | 0.49 | 0.51 | 0.49 | 0.54 | 0.46 | 0.48 | 0.47 | 0.48 |
| 42 | | ISS10+ \| TBI Sev 3 vs. ISS10+ \| TBI Sev 4 | 0.39 | 0.45 | 0.43 | 0.46 | 0.46 | 0.45 | 0.41 | 0.51 | 0.48 | 0.50 | 0.48 |

*Note*. No. = continuous numbering of comparisons; TBI Sev 1 = uncomplicated mild TBI, TBI Sev 2 = complicated mild TBI, TBI Sev 3 = moderate TBI, TBI Sev 4 = severe TBI; ER = emergency room, admission = admission to a hospital ward, ICU = intensive care unit; missing pairwise comparisons indicate insufficient ($n \leq 28$) number of participants; dark red cells = more than a medium-sized effect (< 0.36 | > 0.64 ), light red cells = more than a small, but less than a medium effect (0.36 - 0.43 | 0.64 - 0.55), white cells = small effect (0.44 - 0.51 | 0.51 - 0.56). Values below 0.50 indicate for a better outcome in the patient group 1 compared to the group 2. Rounding errors may occur on the second decimal place.

**Interpretation example:** Six months after TBI, the instruments measuring disease-specific (QOLIBRI/-OS) and mental HRQOL (SF-36v2/-12v2) as well as psychological outcomes (GAD-7, PHQ-9, PCL-5, RPQ) are moderately to highly sensitive for detecting differences between individuals with and without premorbid mental health problems, especially within the groups after complicated mTBI and severe TBI. Since individuals with premorbid mental health problems have significantly worse outcomes in these areas, it is recommended to apply these instruments for identifying potential problems and finding appropriate treatment.

## 12 months after TBI

| No. | Variable | Groups | GOSE/-Q | SF-36v2 PCS | SF-12v2 PCS | SF-36v2 MCS | SF-12v2 MCS | QOLIBRI | QOLIBRI-OS | GAD-7 | PHQ-9 | PCL-5 | RPQ |
|---|---|---|---|---|---|---|---|---|---|---|---|---|---|
| 1 | | male \| TBI Sev 1 vs. female \| TBI Sev 1 | 0.45 | 0.41 | 0.43 | 0.39 | 0.40 | 0.39 | 0.41 | 0.43 | 0.37 | 0.34 | 0.39 |
| 2 | | male \| TBI Sev 2 vs. female \| TBI Sev 2 | 0.43 | 0.42 | 0.44 | 0.42 | 0.43 | 0.47 | 0.47 | 0.41 | 0.41 | 0.43 | 0.44 |
| 3 | | male \| TBI Sev 3 vs. female \| TBI Sev 3 | 0.52 | 0.48 | 0.50 | 0.45 | 0.43 | 0.50 | 0.53 | 0.44 | 0.45 | 0.45 | 0.39 |
| 4 | | male \| TBI Sev 4 vs. female \| TBI Sev 4 | 0.49 | 0.47 | 0.47 | 0.47 | 0.48 | 0.48 | 0.50 | 0.47 | 0.43 | 0.45 | 0.46 |
| 5 | Sex | male \| TBI Sev 1 vs. male \| TBI Sev 2 | 0.41 | 0.45 | 0.43 | 0.42 | 0.44 | 0.40 | 0.43 | 0.44 | 0.44 | 0.41 | 0.40 |
| 6 | | male \| TBI Sev 2 vs. male \| TBI Sev 3 | 0.36 | 0.47 | 0.48 | 0.48 | 0.48 | 0.49 | 0.46 | 0.45 | 0.48 | 0.44 | 0.46 |
| 7 | | male \| TBI Sev 3 vs. male \| TBI Sev 4 | 0.41 | 0.48 | 0.45 | 0.43 | 0.46 | 0.43 | 0.44 | 0.47 | 0.44 | 0.48 | 0.45 |
| 8 | | female \| TBI Sev 1 vs. female \| TBI Sev 2 | 0.39 | 0.48 | 0.47 | 0.45 | 0.45 | 0.48 | 0.50 | 0.43 | 0.46 | 0.50 | 0.45 |
| 9 | | female \| TBI Sev 2 vs. female \| TBI Sev 3 | 0.43 | 0.51 | 0.55 | 0.49 | 0.48 | 0.51 | 0.52 | 0.49 | 0.51 | 0.45 | 0.41 |
| 10 | | female \| TBI Sev 3 vs. female \| TBI Sev 4 | 0.36 | 0.47 | 0.42 | 0.46 | 0.51 | 0.41 | 0.41 | 0.49 | 0.43 | 0.47 | 0.53 |
| 11 | | <65 \| TBI Sev 1 vs. 65+ \| TBI Sev 1 | 0.49 | 0.32 | 0.33 | 0.49 | 0.49 | 0.40 | 0.42 | 0.56 | 0.53 | 0.49 | 0.48 |
| 12 | | <65 \| TBI Sev 2 vs. 65+ \| TBI Sev 2 | 0.48 | 0.33 | 0.30 | 0.56 | 0.55 | 0.48 | 0.44 | 0.60 | 0.54 | 0.60 | 0.58 |
| 13 | Age | <65 \| TBI Sev 1 vs. <65 \| TBI Sev 2 | 0.40 | 0.46 | 0.46 | 0.40 | 0.42 | 0.41 | 0.46 | 0.42 | 0.44 | 0.41 | 0.39 |
| 14 | | <65 \| TBI Sev 2 vs. <65 \| TBI Sev 3 | 0.39 | 0.46 | 0.49 | 0.49 | 0.49 | 0.49 | 0.48 | 0.48 | 0.51 | 0.47 | 0.46 |
| 15 | | <65 \| TBI Sev 3 vs. <65 \| TBI Sev 4 | 0.37 | 0.45 | 0.40 | 0.44 | 0.47 | 0.41 | 0.41 | 0.48 | 0.42 | 0.48 | 0.48 |
| 16 | | 65+ \| TBI Sev 1 vs. 65+ \| TBI Sev 2 | 0.40 | 0.49 | 0.45 | 0.47 | 0.47 | 0.48 | 0.47 | 0.46 | 0.45 | 0.51 | 0.48 |
| 17 | | primary and less \| TBI Sev 1 vs. secondary+ \| TBI Sev 1 | 0.52 | 0.59 | 0.57 | 0.55 | 0.56 | 0.58 | 0.50 | 0.49 | 0.49 | 0.51 | 0.54 |
| 18 | | primary and less \| TBI Sev 2 vs. secondary+ \| TBI Sev 2 | 0.52 | 0.56 | 0.58 | 0.48 | 0.44 | 0.56 | 0.50 | 0.47 | 0.47 | 0.51 | 0.46 |
| 19 | Education | primary and less \| TBI Sev 1 vs. Primary and less\| TBI Sev 2 | 0.41 | 0.50 | 0.48 | 0.48 | 0.54 | 0.47 | 0.48 | 0.46 | 0.47 | 0.44 | 0.50 |
| 20 | | secondary+ \| TBI Sev 1 vs. secondary+ \| TBI Sev 2 | 0.41 | 0.45 | 0.45 | 0.43 | 0.43 | 0.44 | 0.46 | 0.44 | 0.45 | 0.44 | 0.41 |
| 21 | | secondary+ \| TBI Sev 2 vs. secondary+ \| TBI Sev 3 | 0.37 | 0.47 | 0.49 | 0.48 | 0.50 | 0.47 | 0.48 | 0.46 | 0.50 | 0.44 | 0.46 |
| 22 | | secondary+ \| TBI Sev 3 vs. secondary+ \| TBI Sev 4 | 0.41 | 0.50 | 0.46 | 0.45 | 0.46 | 0.43 | 0.42 | 0.48 | 0.44 | 0.49 | 0.47 |
| 23 | | absent \| TBI Sev 1 vs. present \| TBI Sev 1 | 0.41 | 0.47 | 0.47 | 0.36 | 0.36 | 0.38 | 0.37 | 0.38 | 0.38 | 0.37 | 0.43 |
| 24 | | absent \| TBI Sev 2 vs. present \| TBI Sev 2 | 0.40 | 0.45 | 0.47 | 0.31 | 0.31 | 0.30 | 0.38 | 0.31 | 0.31 | 0.33 | 0.33 |
| 25 | Premorbid | absent \| TBI Sev 4 vs. present \| TBI Sev 4 | 0.47 | 0.47 | 0.48 | 0.35 | 0.35 | 0.32 | 0.36 | 0.29 | 0.28 | 0.27 | 0.31 |
| 26 | psych. | absent \| TBI Sev 1 vs. absent \| TBI Sev 2 | 0.39 | 0.45 | 0.43 | 0.43 | 0.44 | 0.42 | 0.44 | 0.44 | 0.45 | 0.45 | 0.42 |
| 27 | problems | absent \| TBI Sev 2 vs. absent \| TBI Sev 3 | 0.39 | 0.50 | 0.50 | 0.46 | 0.48 | 0.52 | 0.50 | 0.44 | 0.48 | 0.44 | 0.45 |
| 28 | | absent \| TBI Sev 3 vs. absent \| TBI Sev 4 | 0.42 | 0.46 | 0.45 | 0.46 | 0.48 | 0.40 | 0.42 | 0.50 | 0.45 | 0.50 | 0.49 |
| 29 | | present \| TBI Sev 1 vs. present \| TBI Sev 2 | 0.39 | 0.44 | 0.44 | 0.39 | 0.38 | 0.34 | 0.45 | 0.38 | 0.39 | 0.41 | 0.34 |
| 30 | | admission \| TBI Sev 1 vs. ICU \| TBI Sev 1 | 0.42 | 0.39 | 0.39 | 0.50 | 0.51 | 0.49 | 0.52 | 0.51 | 0.45 | 0.45 | 0.45 |
| 31 | | admission \| TBI Sev 2 vs. ICU \| TBI Sev 2 | 0.41 | 0.45 | 0.46 | 0.43 | 0.44 | 0.42 | 0.41 | 0.41 | 0.41 | 0.38 | 0.40 |
| 32 | Clinical care | admission \| TBI Sev 1 vs. admission \| TBI Sev 2 | 0.43 | 0.45 | 0.44 | 0.46 | 0.46 | 0.46 | 0.50 | 0.48 | 0.48 | 0.48 | 0.45 |
| 33 | pathways | ICU \| TBI Sev 1 vs. ICU \| TBI Sev 2 | 0.43 | 0.51 | 0.51 | 0.39 | 0.40 | 0.38 | 0.38 | 0.38 | 0.42 | 0.41 | 0.39 |
| 34 | | ICU \| TBI Sev 2 vs. ICU \| TBI Sev 3 | 0.40 | 0.50 | 0.53 | 0.52 | 0.51 | 0.53 | 0.52 | 0.50 | 0.54 | 0.49 | 0.48 |
| 35 | | ICU \| TBI Sev 3 vs. ICU \| TBI Sev 4 | 0.41 | 0.49 | 0.44 | 0.45 | 0.49 | 0.43 | 0.44 | 0.49 | 0.44 | 0.50 | 0.49 |
| 36 | | ISS<10 \| TBI Sev 1 vs. ISS10+ \| TBI Sev 1 | 0.43 | 0.44 | 0.44 | 0.52 | 0.54 | 0.53 | 0.53 | 0.54 | 0.56 | 0.50 | 0.50 |
| 37 | | ISS<10 \| TBI Sev 2 vs. ISS10+ \| TBI Sev 2 | 0.47 | 0.45 | 0.47 | 0.51 | 0.48 | 0.50 | 0.48 | 0.49 | 0.50 | 0.49 | 0.48 |
| 38 | Injury severity | ISS<10 \| TBI Sev 1 vs. ISS<10 \| TBI Sev 2 | 0.38 | 0.46 | 0.43 | 0.43 | 0.47 | 0.44 | 0.48 | 0.46 | 0.48 | 0.45 | 0.43 |
| 39 | score | ISS10+ \| TBI Sev 1 vs. ISS10+ \| TBI Sev 2 | 0.42 | 0.46 | 0.45 | 0.42 | 0.41 | 0.42 | 0.44 | 0.42 | 0.42 | 0.43 | 0.41 |
| 40 | | ISS10+ \| TBI Sev 2 vs. ISS10+ \| TBI Sev 3 | 0.39 | 0.51 | 0.52 | 0.49 | 0.49 | 0.50 | 0.50 | 0.47 | 0.50 | 0.45 | 0.45 |
| 41 | | ISS10+ \| TBI Sev 3 vs. ISS10+ \| TBI Sev 4 | 0.39 | 0.46 | 0.42 | 0.44 | 0.47 | 0.41 | 0.42 | 0.48 | 0.43 | 0.48 | 0.47 |

*Note.* No. = continuous numbering of comparisons; TBI Sev 1 = uncomplicated mild TBI, TBI Sev 2 = complicated mild TBI, TBI Sev 3 = moderate TBI, TBI Sev 4 = severe TBI; ER = emergency room, admission = admission to a hospital ward, ICU = intensive care unit; missing pairwise comparisons indicate insufficient ($n \leq 28$) number of participants; dark red cells = more than a medium-sized effect (< 0.36 | > 0.64 ), light red cells = more than a small, but less than a medium effect (0.36 - 0.43 | 0.64 - 0.55), white cells = small effect (0.44 - 0.51 | 0.51 - 0.56). Values below 0.50 indicate for a better outcome in the patient group 1 compared to the group 2. Rounding errors may occur on the second decimal place.

**Interpretation example:** Twelve months after TBI, the instruments measuring disease-specific (QOLIBRI/-OS) and mental HRQOL (SF-36v2/-12v2) as well as psychological outcomes (GAD-7, PHQ-9, PCL-5, RPQ) are moderately to highly sensitive for detecting differences between individuals with and without premorbid mental health problems, especially within the groups after uncomplicated mTBI, complicated mTBI, and severe TBI as well as between individuals with present premorbid psychological problems after uncomplicated and complicated mTBI. Since individuals with premorbid mental health problems have significantly worse outcomes in these areas, it is recommended to apply these instruments for identifying potential problems and finding appropriate treatment, especially for those with higher TBI severity.
