# Supplementary material for: Sensitivity of outcome instruments in a priori selected patient groups after traumatic brain injury: Results from the CENTER-TBI study
Source: PLoS One. 2023 Apr 7;18(4):e0280796. doi: 10.1371/journal.pone.0280796 (PMC10081802; doi:10.1371/journal.pone.0280796)
Supplement: S5 Table — (PDF) [file pone.0280796.s005.pdf]

Mann-Whitney effect size
3 months after TBI

No.	Variable	Groups	GOSE/-Q	SF-36v2 PCS	SF-12v2 PCS	SF-36v2 MCS	SF-12v2 MCS	QOLIBRI	QOLIBRI-OS	GAD-7	PHQ-9	PCL-5	RPQ
1		male | TBI Sev 1 vs. female | TBI Sev 1	0.46	0.37	0.36	0.43	0.41	0.42	0.40	0.44	0.37	0.38	0.30
2		male | TBI Sev 2 vs. female | TBI Sev 2	0.44	0.40	0.39	0.45	0.43	0.45	0.46	0.42	0.42	0.44	0.44
3		male | TBI Sev 3 vs. female | TBI Sev 3	0.56	0.45	0.53	0.49	0.43	0.48	0.53	0.40	0.47	0.43	0.38
4		male | TBI Sev 4 vs. female | TBI Sev 4	0.46	0.50	0.45	0.42	0.40	0.43	0.46	0.41	0.40	0.42	0.43
5	Sex	male | TBI Sev 1 vs. male | TBI Sev 2	0.40	0.51	0.49	0.45	0.44	0.45	0.46	0.50	0.48	0.48	0.40
6		male | TBI Sev 2 vs. male | TBI Sev 3	0.31	0.44	0.41	0.50	0.52	0.49	0.46	0.50	0.49	0.51	0.49
7		male | TBI Sev 3 vs. male | TBI Sev 4	0.42	0.40	0.44	0.46	0.45	0.42	0.43	0.45	0.43	0.46	0.41
8		female | TBI Sev 1 vs. female | TBI Sev 2	0.39	0.54	0.54	0.46	0.45	0.47	0.51	0.47	0.52	0.53	0.53
9		female | TBI Sev 2 vs. female | TBI Sev 3	0.41	0.49	0.55	0.54	0.53	0.53	0.52	0.51	0.55	0.51	0.44
10		female | TBI Sev 3 vs. female | TBI Sev 4	0.30	0.45	0.36	0.37	0.42	0.36	0.36	0.45	0.35	0.44	0.47
11		<65 | TBI Sev 1 vs. 65+ | TBI Sev 1	0.52	0.36	0.38	0.53	0.58	0.42	0.50	0.53	0.55	0.56	0.47
12		<65 | TBI Sev 2 vs. 65+ | TBI Sev 2	0.50	0.42	0.41	0.59	0.56	0.52	0.52	0.60	0.58	0.63	0.60
13	Age	<65 | TBI Sev 1 vs. <65 | TBI Sev 2	0.39	0.51	0.50	0.43	0.44	0.43	0.47	0.46	0.48	0.47	0.41
14		<65 | TBI Sev 2 vs. <65 | TBI Sev 3	0.34	0.43	0.45	0.53	0.52	0.49	0.49	0.52	0.51	0.53	0.46
15		<65 | TBI Sev 3 vs. <65 | TBI Sev 4	0.37	0.40	0.41	0.43	0.48	0.42	0.40	0.46	0.43	0.47	0.47
16		65+ | TBI Sev 1 vs. 65+ | TBI Sev 2	0.40	0.58	0.56	0.50	0.42	0.52	0.49	0.54	0.51	0.54	0.53
17		primary and less | TBI Sev 1 vs. secondary+ | TBI Sev 1	0.51	0.66	0.65	0.48	0.42	0.53	0.45	0.50	0.46	0.50	0.49
18		primary and less | TBI Sev 2 vs. secondary+ | TBI Sev 2	0.49	0.56	0.56	0.48	0.48	0.56	0.49	0.52	0.47	0.52	0.47
19	Education	primary and less | TBI Sev 1 vs. primary and less | TBI Sev 2	0.43	0.62	0.62	0.46	0.39	0.45	0.44	0.48	0.49	0.48	0.46
20		secondary+ | TBI Sev 1 vs. secondary+ | TBI Sev 2	0.39	0.52	0.51	0.45	0.45	0.47	0.49	0.49	0.50	0.50	0.44
21		secondary+ | TBI Sev 2 vs. secondary+ | TBI Sev 3	0.33	0.43	0.43	0.53	0.54	0.48	0.47	0.49	0.52	0.51	0.49
22		secondary+ | TBI Sev 3 vs. secondary+ | TBI Sev 4	0.39	0.42	0.42	0.43	0.45	0.40	0.39	0.48	0.39	0.47	0.41
23		absent | TBI Sev 1 vs. present | TBI Sev 1	0.47	0.47	0.47	0.35	0.35	0.39	0.37	0.38	0.32	0.38	0.38
24		absent | TBI Sev 2 vs. present | TBI Sev 2	0.44	0.45	0.46	0.31	0.30	0.31	0.36	0.30	0.28	0.33	0.35
25	Premorbid psychological problems	absent | TBI Sev 4 vs. present | TBI Sev 4	0.44	0.49	0.46	0.37	0.41	0.42	0.39	0.35	0.36	0.43	0.42
26		absent | TBI Sev 1 vs. absent | TBI Sev 2	0.42	0.52	0.51	0.46	0.45	0.47	0.49	0.50	0.51	0.51	0.45
27		absent | TBI Sev 2 vs. absent | TBI Sev 3	0.35	0.46	0.44	0.48	0.50	0.49	0.47	0.45	0.47	0.50	0.47
28		absent | TBI Sev 3 vs. absent | TBI Sev 4	0.40	0.41	0.45	0.46	0.46	0.40	0.43	0.50	0.44	0.44	0.43
29		present | TBI Sev 1 vs. present | TBI Sev 2	0.38	0.51	0.50	0.38	0.34	0.36	0.46	0.41	0.42	0.46	0.43
30		admission | TBI Sev 1 vs. ICU | TBI Sev 1	0.36	0.36	0.31	0.49	0.51	0.49	0.41	0.45	0.41	0.43	0.43
31		admission | TBI Sev 2 vs. ICU | TBI Sev 2	0.35	0.45	0.47	0.43	0.43	0.41	0.42	0.44	0.43	0.40	0.39
32	Clinical care pathways	admission | TBI Sev 1 vs. admission | TBI Sev 2	0.43	0.52	0.48	0.48	0.48	0.50	0.50	0.51	0.51	0.53	0.48
33		ICU | TBI Sev 1 vs. ICU | TBI Sev 2	0.43	0.60	0.65	0.42	0.40	0.40	0.50	0.48	0.51	0.49	0.42
34		ICU | TBI Sev 2 vs. ICU | TBI Sev 3	0.39	0.46	0.47	0.55	0.54	0.54	0.51	0.53	0.55	0.55	0.52
35		ICU | TBI Sev 3 vs. ICU | TBI Sev 4	0.41	0.44	0.43	0.44	0.47	0.43	0.42	0.48	0.42	0.49	0.46
36		ISS<10 | TBI Sev 1 vs. ISS10+ | TBI Sev 1	0.42	0.44	0.43	0.58	0.56	0.54	0.49	0.56	0.59	0.51	0.52
37		ISS<10 | TBI Sev 2 vs. ISS10+ | TBI Sev 2	0.39	0.43	0.46	0.47	0.46	0.49	0.48	0.49	0.47	0.47	0.47
38	Injury severity score	ISS<10 | TBI Sev 1 vs. ISS<10 | TBI Sev 2	0.42	0.53	0.49	0.52	0.52	0.50	0.49	0.53	0.56	0.53	0.49
39		ISS10+ | TBI Sev 1 vs. ISS10+ | TBI Sev 2	0.39	0.52	0.52	0.42	0.41	0.43	0.47	0.46	0.46	0.48	0.43
40		ISS10+ | TBI Sev 2 vs. ISS10+ | TBI Sev 3	0.35	0.46	0.47	0.51	0.52	0.51	0.48	0.49	0.52	0.51	0.47
41		ISS10+ | TBI Sev 3 vs. ISS10+ | TBI Sev 4	0.39	0.42	0.42	0.44	0.47	0.41	0.41	0.48	0.42	0.47	0.45

Note. No. = continuous numbering of comparisons; TBI Sev 1 = uncomplicated mild TBI, TBI Sev 2 = complicated mild TBI, TBI Sev 3 = moderate TBI, TBI Sev 4 = severe TBI; ER = emergency room, admission = admission to a hospital ward, ICU = intensive care unit; missing pairwise comparisons indicate insufficient (n ≤ 28) number of participants; dark red cells = more than a medium-sized effect (< 0.36 | > 0.64), light red cells = more than a small, but less than a medium effect (0.36 - 0.43 | 0.64 - 0.55), white cells = small effect (0.44 - 0.51 | 0.51 - 0.56). Values below 0.50 indicate a better outcome in patient group 1 compared to group 2. Rounding errors may occur on the second decimal place.

Interpretation example: Three months after TBI, the instruments measuring disease-specific (QOLIBRI/-OS) and mental HRQOL (SF-36v2/-12v2) as well as psychological outcomes (GAD-7, PHQ-9, PCL-5, RPQ) are moderately to highly sensitive for detecting differences between individuals with and without premorbid mental health problems, especially within the groups after uncomplicated mTBI, complicated mTBI, and severe TBI. Since individuals with premorbid mental health problems have significantly worse outcomes in these areas, it is recommended to apply these instruments for identifying potential problems and finding appropriate treatment.

6 months after TBI

No.	Variable	Groups	GOSE/-Q	SF-36v2 PCS	SF-12v2 PCS	SF-36v2 MCS	SF-12v2 MCS	QOLIBRI	QOLIBRI-OS	GAD-7	PHQ-9	PCL-5	RPQ
1	Sex	male | TBI Sev 1 vs. female | TBI Sev 1	0.41	0.36	0.36	0.42	0.41	0.39	0.41	0.38	0.36	0.40	0.34
2		male | TBI Sev 2 vs. female | TBI Sev 2	0.41	0.39	0.37	0.43	0.42	0.46	0.44	0.43	0.42	0.47	0.45
3		male | TBI Sev 3 vs. female | TBI Sev 3	0.54	0.46	0.51	0.42	0.45	0.49	0.50	0.37	0.43	0.40	0.41
4		male | TBI Sev 4 vs. female | TBI Sev 4	0.51	0.52	0.50	0.43	0.42	0.45	0.47	0.41	0.42	0.44	0.45
5		male | TBI Sev 1 vs. male | TBI Sev 2	0.41	0.49	0.48	0.48	0.48	0.43	0.45	0.47	0.44	0.46	0.39
6		male | TBI Sev 2 vs. male | TBI Sev 3	0.33	0.47	0.44	0.49	0.49	0.49	0.52	0.49	0.50	0.50	0.49
7		male | TBI Sev 3 vs. male | TBI Sev 4	0.40	0.41	0.42	0.42	0.46	0.45	0.40	0.46	0.44	0.46	0.43
8		female | TBI Sev 1 vs. female | TBI Sev 2	0.40	0.54	0.52	0.49	0.49	0.50	0.50	0.50	0.50	0.53	0.52
9		female | TBI Sev 2 vs. female | TBI Sev 3	0.46	0.54	0.59	0.47	0.52	0.52	0.57	0.46	0.52	0.40	0.44
10		female | TBI Sev 3 vs. female | TBI Sev 4	0.37	0.46	0.40	0.42	0.43	0.41	0.37	0.48	0.42	0.50	0.48
11	Age	<65 | TBI Sev 1 vs. 65+ | TBI Sev 1	0.48	0.36	0.34	0.51	0.51	0.46	0.45	0.52	0.53	0.55	0.51
12		<65 | TBI Sev 2 vs. 65+ | TBI Sev 2	0.48	0.36	0.35	0.59	0.56	0.50	0.49	0.59	0.59	0.65	0.61
13		<65 | TBI Sev 1 vs. <65 | TBI Sev 2	0.40	0.51	0.50	0.46	0.47	0.44	0.46	0.46	0.44	0.45	0.40
14		<65 | TBI Sev 2 vs. <65 | TBI Sev 3	0.37	0.46	0.47	0.51	0.51	0.49	0.53	0.49	0.54	0.50	0.49
15		<65 | TBI Sev 3 vs. <65 | TBI Sev 4	0.37	0.41	0.39	0.41	0.45	0.43	0.39	0.46	0.41	0.48	0.45
16		65+ | TBI Sev 1 vs. 65+ | TBI Sev 2	0.42	0.54	0.53	0.53	0.51	0.49	0.50	0.52	0.49	0.55	0.49
17	Education	primary and less | TBI Sev 1 vs. secondary+ | TBI Sev 1	0.51	0.70	0.67	0.56	0.54	0.62	0.47	0.62	0.59	0.55	0.54
18		primary and less | TBI Sev 2 vs. secondary+ | TBI Sev 2	0.51	0.53	0.55	0.52	0.51	0.56	0.53	0.50	0.48	0.47	0.43
19		primary and less | TBI Sev 1 vs. primary and less | TBI Sev 2	0.42	0.68	0.63	0.54	0.52	0.54	0.44	0.60	0.55	0.56	0.52
20		secondary+ | TBI Sev 1 vs. secondary+ | TBI Sev 2	0.41	0.49	0.49	0.49	0.50	0.46	0.49	0.48	0.46	0.48	0.43
21		secondary+ | TBI Sev 2 vs. secondary+ | TBI Sev 3	0.36	0.49	0.47	0.46	0.48	0.48	0.51	0.46	0.50	0.45	0.50
22		secondary+ | TBI Sev 3 vs. secondary+ | TBI Sev 4	0.41	0.43	0.43	0.45	0.47	0.45	0.40	0.51	0.46	0.52	0.45
23	Premorbid psychological problems	absent | TBI Sev 1 vs. present | TBI Sev 1	0.42	0.47	0.49	0.33	0.33	0.33	0.35	0.32	0.34	0.40	0.36
24		absent | TBI Sev 2 vs. present | TBI Sev 2	0.45	0.48	0.48	0.31	0.33	0.33	0.38	0.30	0.31	0.30	0.38
25		absent | TBI Sev 4 vs. present | TBI Sev 4	0.50	0.41	0.47	0.46	0.41	0.41	0.41	0.37	0.34	0.36	0.39
26		absent | TBI Sev 1 vs. absent | TBI Sev 2	0.39	0.51	0.48	0.49	0.47	0.44	0.46	0.49	0.46	0.50	0.42
27		absent | TBI Sev 2 vs. absent | TBI Sev 3	0.41	0.50	0.49	0.47	0.49	0.52	0.55	0.44	0.49	0.45	0.47
28		absent | TBI Sev 3 vs. absent | TBI Sev 4	0.38	0.44	0.43	0.41	0.45	0.41	0.38	0.50	0.45	0.49	0.46
29		present | TBI Sev 1 vs. present | TBI Sev 2	0.42	0.52	0.50	0.44	0.44	0.45	0.49	0.43	0.44	0.41	0.48
30	Clinical care pathways	admission | TBI Sev 1 vs. ICU | TBI Sev 1	0.38	0.40	0.39	0.47	0.46	0.47	0.49	0.46	0.46	0.43	0.47
31		admission | TBI Sev 2 vs. ICU | TBI Sev 2	0.37	0.46	0.46	0.42	0.43	0.42	0.43	0.43	0.42	0.40	0.38
32		admission | TBI Sev 1 vs. admission | TBI Sev 2	0.44	0.50	0.48	0.52	0.51	0.48	0.50	0.51	0.48	0.51	0.47
33		ICU | TBI Sev 1 vs. ICU | TBI Sev 2	0.43	0.56	0.55	0.44	0.46	0.42	0.44	0.46	0.44	0.48	0.39
34		ICU | TBI Sev 2 vs. ICU | TBI Sev 3	0.42	0.49	0.51	0.52	0.53	0.53	0.56	0.49	0.54	0.48	0.52
35		ICU | TBI Sev 3 vs. ICU | TBI Sev 4	0.41	0.44	0.43	0.45	0.47	0.45	0.40	0.51	0.45	0.52	0.48
36	Injury severity score	ISS<10 | TBI Sev 1 vs. ISS10+ | TBI Sev 1	0.43	0.47	0.49	0.56	0.55	0.55	0.53	0.57	0.57	0.56	0.55
37		ISS<10 | TBI Sev 2 vs. ISS10+ | TBI Sev 2	0.43	0.46	0.48	0.51	0.51	0.52	0.52	0.51	0.49	0.50	0.44
38		ISS<10 | TBI Sev 1 vs. ISS<10 | TBI Sev 2	0.41	0.51	0.50	0.51	0.52	0.47	0.48	0.51	0.51	0.52	0.50
39		ISS10+ | TBI Sev 1 vs. ISS10+ | TBI Sev 2	0.41	0.51	0.49	0.46	0.47	0.44	0.46	0.46	0.44	0.47	0.40
40		ISS10+ | TBI Sev 2 vs. ISS10+ | TBI Sev 3	0.38	0.49	0.50	0.48	0.49	0.50	0.54	0.46	0.49	0.45	0.49
41		ISS10+ | TBI Sev 3 vs. ISS10+ | TBI Sev 4	0.39	0.42	0.41	0.44	0.47	0.43	0.39	0.50	0.45	0.50	0.46

Note. No. = continuous numbering of comparisons; TBI Sev 1 = uncomplicated mild TBI, TBI Sev 2 = complicated mild TBI, TBI Sev 3 = moderate TBI, TBI Sev 4 = severe TBI; ER = emergency room, admission = admission to a hospital ward, ICU = intensive care unit; missing pairwise comparisons indicate insufficient (n ≤ 28) number of participants; dark red cells = more than a medium-sized effect (< 0.36 | > 0.64), light red cells = more than a small, but less than a medium effect (0.36 - 0.43 | 0.64 - 0.55), white cells = small effect (0.44 - 0.51 | 0.51 - 0.56). Values below 0.50 indicate a better outcome in patient group 1 compared to group 2. Rounding errors may occur on the second decimal place.

<u>Interpretation example:</u> Six months after TBI, the instruments measuring disease-specific (QOLIBRI/-OS) and mental HRQOL (SF-36v2/-12v2) as well as psychological outcomes (GAD-7, PHQ-9, PCL-5, RPQ) are moderately to highly sensitive for detecting differences between individuals with and without premorbid mental health problems, especially within the groups after uncomplicated mTBI, complicated mTBI, and severe TBI. Since individuals with premorbid mental health problems have significantly worse outcomes in these areas, it is recommended to apply these instruments for identifying potential problems and finding appropriate treatment.

12 months after TBI

No.	Variable	Groups	GOSE/-Q	SF-36v2 PCS	SF-12v2 PCS	SF-36v2 MCS	SF-12v2 MCS	QOLIBRI	QOLIBRI-OS	GAD-7	PHQ-9	PCL-5	RPQ
1	Sex	male | TBI Sev 1 vs. female | TBI Sev 1	0.45	0.40	0.41	0.38	0.41	0.39	0.39	0.43	0.37	0.33	0.40
2		male | TBI Sev 2 vs. female | TBI Sev 2	0.42	0.42	0.42	0.42	0.41	0.46	0.47	0.43	0.41	0.42	0.44
3		male | TBI Sev 3 vs. female | TBI Sev 3	0.51	0.47	0.50	0.48	0.43	0.48	0.53	0.42	0.45	0.43	0.40
4		male | TBI Sev 4 vs. female | TBI Sev 4	0.50	0.48	0.50	0.44	0.45	0.46	0.50	0.43	0.41	0.41	0.48
5		male | TBI Sev 1 vs. male | TBI Sev 2	0.42	0.45	0.44	0.43	0.45	0.39	0.43	0.44	0.45	0.43	0.40
6		male | TBI Sev 2 vs. male | TBI Sev 3	0.38	0.50	0.50	0.45	0.49	0.52	0.49	0.46	0.49	0.45	0.47
7		male | TBI Sev 3 vs. male | TBI Sev 4	0.39	0.46	0.44	0.46	0.45	0.40	0.42	0.45	0.42	0.46	0.43
8		female | TBI Sev 1 vs. female | TBI Sev 2	0.39	0.48	0.49	0.48	0.45	0.46	0.51	0.44	0.47	0.52	0.44
9		female | TBI Sev 2 vs. female | TBI Sev 3	0.45	0.53	0.57	0.51	0.51	0.53	0.54	0.50	0.52	0.45	0.43
10		female | TBI Sev 3 vs. female | TBI Sev 4	0.36	0.49	0.42	0.42	0.47	0.39	0.39	0.43	0.39	0.44	0.53
11	Age	<65 | TBI Sev 1 vs. 65+ | TBI Sev 1	0.49	0.34	0.36	0.48	0.50	0.42	0.44	0.56	0.54	0.51	0.49
12		<65 | TBI Sev 2 vs. 65+ | TBI Sev 2	0.47	0.33	0.31	0.59	0.57	0.49	0.46	0.60	0.57	0.61	0.59
13		<65 | TBI Sev 1 vs. <65 | TBI Sev 2	0.41	0.47	0.48	0.41	0.43	0.40	0.46	0.42	0.45	0.43	0.39
14		<65 | TBI Sev 2 vs. <65 | TBI Sev 3	0.40	0.48	0.51	0.50	0.51	0.52	0.50	0.49	0.53	0.48	0.47
15		<65 | TBI Sev 3 vs. <65 | TBI Sev 4	0.35	0.45	0.40	0.43	0.45	0.38	0.40	0.44	0.38	0.45	0.47
16		65+ | TBI Sev 1 vs. 65+ | TBI Sev 2	0.41	0.48	0.45	0.51	0.49	0.46	0.47	0.46	0.47	0.52	0.49
17	Education	primary and less | TBI Sev 1 vs. secondary+ | TBI Sev 1	0.50	0.66	0.61	0.59	0.52	0.59	0.47	0.56	0.51	0.57	0.56
18		primary and less | TBI Sev 2 vs. secondary+ | TBI Sev 2	0.52	0.57	0.59	0.47	0.43	0.57	0.50	0.49	0.46	0.51	0.44
19		primary and less | TBI Sev 1 vs. primary and less | TBI Sev 2	0.41	0.57	0.52	0.56	0.53	0.47	0.46	0.51	0.50	0.53	0.52
20		secondary+ | TBI Sev 1 vs. secondary+ | TBI Sev 2	0.41	0.46	0.46	0.44	0.44	0.42	0.47	0.44	0.46	0.45	0.41
21		secondary+ | TBI Sev 2 vs. secondary+ | TBI Sev 3	0.39	0.48	0.50	0.47	0.52	0.50	0.51	0.47	0.51	0.44	0.48
22		secondary+ | TBI Sev 3 vs. secondary+ | TBI Sev 4	0.39	0.50	0.46	0.45	0.45	0.40	0.40	0.45	0.41	0.45	0.44
23	Premorbid psychological problems	absent | TBI Sev 1 vs. present | TBI Sev 1	0.42	0.46	0.48	0.35	0.36	0.40	0.38	0.40	0.37	0.39	0.46
24		absent | TBI Sev 2 vs. present | TBI Sev 2	0.39	0.45	0.46	0.29	0.29	0.30	0.36	0.28	0.30	0.32	0.32
25		absent | TBI Sev 4 vs. present | TBI Sev 4	0.49	0.47	0.52	0.36	0.38	0.36	0.39	0.34	0.32	0.29	0.35
26		absent | TBI Sev 1 vs. absent | TBI Sev 2	0.41	0.46	0.45	0.44	0.45	0.42	0.45	0.45	0.46	0.47	0.44
27		absent | TBI Sev 2 vs. absent | TBI Sev 3	0.40	0.50	0.50	0.44	0.48	0.53	0.52	0.44	0.49	0.44	0.45
28		absent | TBI Sev 3 vs. absent | TBI Sev 4	0.40	0.47	0.45	0.48	0.47	0.39	0.40	0.47	0.43	0.48	0.46
29		present | TBI Sev 1 vs. present | TBI Sev 2	0.38	0.46	0.46	0.38	0.37	0.32	0.43	0.34	0.38	0.40	0.31
30	Clinical care pathways	admission | TBI Sev 1 vs. ICU | TBI Sev 1	0.42	0.45	0.42	0.53	0.56	0.51	0.54	0.53	0.49	0.48	0.46
31		admission | TBI Sev 2 vs. ICU | TBI Sev 2	0.40	0.45	0.47	0.45	0.45	0.42	0.40	0.43	0.42	0.40	0.41
32		admission | TBI Sev 1 vs. admission | TBI Sev 2	0.44	0.47	0.45	0.47	0.48	0.45	0.51	0.48	0.49	0.50	0.45
33		ICU | TBI Sev 1 vs. ICU | TBI Sev 2	0.43	0.47	0.50	0.40	0.38	0.35	0.37	0.37	0.41	0.42	0.38
34		ICU | TBI Sev 2 vs. ICU | TBI Sev 3	0.42	0.51	0.53	0.49	0.51	0.55	0.55	0.49	0.53	0.48	0.48
35		ICU | TBI Sev 3 vs. ICU | TBI Sev 4	0.40	0.49	0.44	0.47	0.48	0.42	0.42	0.48	0.44	0.49	0.50
36	Injury severity score	ISS<10 | TBI Sev 1 vs. ISS10+ | TBI Sev 1	0.43	0.46	0.46	0.58	0.57	0.54	0.54	0.56	0.60	0.51	0.50
37		ISS<10 | TBI Sev 2 vs. ISS10+ | TBI Sev 2	0.47	0.47	0.50	0.54	0.52	0.52	0.51	0.52	0.52	0.53	0.50
38		ISS<10 | TBI Sev 1 vs. ISS<10 | TBI Sev 2	0.38	0.47	0.43	0.46	0.48	0.42	0.48	0.47	0.51	0.45	0.42
39		ISS10+ | TBI Sev 1 vs. ISS10+ | TBI Sev 2	0.42	0.46	0.47	0.43	0.43	0.40	0.44	0.42	0.43	0.46	0.42
40		ISS10+ | TBI Sev 2 vs. ISS10+ | TBI Sev 3	0.40	0.52	0.53	0.45	0.48	0.52	0.51	0.46	0.49	0.44	0.45
41		ISS10+ | TBI Sev 3 vs. ISS10+ | TBI Sev 4	0.38	0.46	0.42	0.46	0.48	0.40	0.40	0.47	0.42	0.47	0.47

Note. No. = continuous numbering of comparisons; TBI Sev 1 = uncomplicated mild TBI, TBI Sev 2 = complicated mild TBI, TBI Sev 3 = moderate TBI, TBI Sev 4 = severe TBI; ER = emergency room, admission = admission to a hospital ward, ICU = intensive care unit; missing pairwise comparisons indicate insufficient (n ≤ 28) number of participants; dark red cells = more than a medium-sized effect (< 0.36 | > 0.64), light red cells = more than a small, but less than a medium effect (0.36 - 0.43 | 0.64 - 0.55), white cells = small effect (0.44 - 0.51 | 0.51 - 0.56). Values below 0.50 indicate a better outcome in patient group 1 compared to group 2. Rounding errors may occur on the second decimal place.

Interpretation example: Twelve months after TBI, the instruments measuring disease-specific (QOLIBRI/-OS) and mental HRQOL (SF-36v2/-12v2) as well as psychological outcomes (GAD-7, PHQ-9, PCL-5, RPQ) are moderately to highly sensitive for detecting differences between individuals with and without premorbid mental health problems, especially within the groups after uncomplicated mTBI, complicated mTBI, and severe TBI as well as between individuals with present premorbid psychological problems after uncomplicated and complicated mTBI. Since individuals with premorbid mental health problems have significantly worse outcomes in these areas, it is recommended to apply these instruments for identifying potential problems and finding appropriate treatment, especially for those with higher TBI severity.
