# Supplementary material for: Sensitivity of outcome instruments in a priori selected patient groups after traumatic brain injury: Results from the CENTER-TBI study
Source: PLoS One. 2023 Apr 7;18(4):e0280796. doi: 10.1371/journal.pone.0280796 (PMC10081802; doi:10.1371/journal.pone.0280796)
Supplement: S6 Table — (PDF) [file pone.0280796.s006.pdf]

S6 Table. The overall sensitivity of the PROMs to pairwise group comparisons with respect to functional recovery status for three time points (completers' data)

Instrument	Three months	Six months	Twelve months	Average
	n = 41	n = 34	n = 29	
SF-36v2 PCS	**100%**	**100%**	**100%**	**100%**
SF-12v2 PCS	95%	**97%**	**100%**	**97%**
SF-36v2 MCS	56%	71%	83%	70%
SF-12v2 MCS	59%	71%	76%	68%
QOLIBRI	**98%**	**79%**	**100%**	**92%**
QOLIBRI-OS	**98%**	76%	**100%**	91%
GAD-7	59%	62%	69%	63%
PHQ-9	61%	74%	79%	71%
PCL-5	61%	62%	76%	66%
RPQ	73%	74%	76%	74%

n = number of pairwise comparisons, % = percentage, average = average relative frequencies from 3 to 12 months. **Bold** values indicate top three instruments with the highest sensitivity (i.e., in most group comparisons).
