# Supplementary material for: Sensitivity of outcome instruments in a priori selected patient groups after traumatic brain injury: Results from the CENTER-TBI study
Source: PLoS One. 2023 Apr 7;18(4):e0280796. doi: 10.1371/journal.pone.0280796 (PMC10081802; doi:10.1371/journal.pone.0280796)
Supplement: S7 Table — (PDF) [file pone.0280796.s007.pdf]

Mann-Whitney effect size
3 months after TBI (data as available)

No.	Variable	Groups	SF-36v2 PCS	SF-12v2 PCS	SF-36v2 MCS	SF-12v2 MCS	QOLIBRI	QOLIBRI-OS	GAD-7	PHQ-9	PCL-5	RPQ
1		male | GOSE/-Q 7-8 vs. male | GOSE/-Q 5-6	0.23	0.23	0.30	0.31	0.28	0.26	0.30	0.26	0.27	0.22
2		male | GOSE/-Q 7-8 vs. male | GOSE/-Q 3-4	0.11	0.11	0.28	0.26	0.17	0.14	0.29	0.23	0.28	0.19
3	Sex	male | GOSE/-Q 5-6 vs. male | GOSE/-Q 3-4	0.30	0.31	0.47	0.43	0.34	0.32	0.48	0.44	0.50	0.45
4		female | GOSE/-Q 7-8 vs. female | GOSE/-Q 5-6	0.30	0.32	0.35	0.35	0.32	0.32	0.37	0.33	0.31	0.25
5		female | GOSE/-Q 7-8 vs. female | GOSE/-Q 3-4	0.14	0.17	0.29	0.29	0.18	0.16	0.31	0.27	0.31	0.23
6		female | GOSE/-Q 5-6 vs. female | GOSE/-Q 3-4	0.24	0.29	0.42	0.42	0.32	0.29	0.44	0.44	0.49	0.48
7		<65 | GOSE/-Q 7-8 vs. <65 | GOSE/-Q 5-6	0.22	0.23	0.33	0.33	0.28	0.27	0.34	0.30	0.30	0.24
8		<65 | GOSE/-Q 7-8 vs. <65 | GOSE/-Q 3-4	0.11	0.11	0.27	0.28	0.16	0.14	0.28	0.23	0.27	0.19
9	Age	<65 | GOSE/-Q 5-6 vs. <65 | GOSE/-Q 3-4	0.30	0.31	0.43	0.42	0.32	0.31	0.44	0.42	0.47	0.43
10		65+ | GOSE/-Q 7-8 vs. 65+ | GOSE/-Q 5-6	0.34	0.32	0.30	0.32	0.29	0.29	0.29	0.26	0.27	0.27
11		65+ | GOSE/-Q 7-8 vs. 65+ | GOSE/-Q 3-4	0.14	0.19	0.31	0.26	0.21	0.18	0.32	0.25	0.32	0.22
12		65+ | GOSE/-Q 5-6 vs. 65+ | GOSE/-Q 3-4	0.26	0.33	0.47	0.43	0.36	0.32	0.51	0.50	0.53	0.46
13		primary and less | GOSE/-Q 7-8 vs. primary and less | GOSE/-Q 5-6	0.28	0.28	0.32	0.34	0.27	0.25	0.32	0.27	0.30	0.22
14		primary and less | GOSE/-Q 7-8 vs. primary and less | GOSE/-Q 3-4	0.14	0.16	0.26	0.25	0.17	0.15	0.29	0.23	0.28	0.17
15	Education	primary and less | GOSE/-Q 5-6 vs. primary and less | GOSE/-Q 3-4	0.29	0.32	0.42	0.39	0.36	0.34	0.45	0.44	0.46	0.42
16		at least secondary | GOSE/-Q 7-8 vs. at least secondary | GOSE/-Q 5-6	0.25	0.25	0.31	0.32	0.28	0.27	0.32	0.29	0.28	0.24
17		at least secondary | GOSE/-Q 7-8 vs. at least secondary | GOSE/-Q 3-4	0.12	0.12	0.29	0.29	0.17	0.14	0.29	0.25	0.27	0.20
18		at least secondary | GOSE/-Q 5-6 vs. at least secondary | GOSE/-Q 3-4	0.28	0.29	0.46	0.45	0.34	0.31	0.47	0.45	0.50	0.46
19		absent | GOSE/-Q 7-8 vs. absent | GOSE/-Q 5-6	0.26	0.27	0.31	0.32	0.29	0.27	0.31	0.28	0.29	0.23
20		absent | GOSE/-Q 7-8 vs. absent | GOSE/-Q 3-4	0.12	0.13	0.25	0.25	0.16	0.15	0.28	0.23	0.28	0.20
21	Premorbid psych. problems	absent | GOSE/-Q 5-6 vs. absent | GOSE/-Q 3-4	0.27	0.29	0.43	0.41	0.33	0.33	0.45	0.43	0.50	0.46
22		present | GOSE/-Q 7-8 vs. present | GOSE/-Q 5-6	0.28	0.25	0.40	0.41	0.31	0.29	0.40	0.35	0.35	0.27
23		present | GOSE/-Q 3-4 vs. present | GOSE/-Q 3-4	0.20	0.20	0.42	0.41	0.24	0.20	0.43	0.35	0.35	0.26
24		present | GOSE/-Q 5-6 vs. present | GOSE/-Q 3-4	0.36	0.39	0.50	0.49	0.39	0.36	0.53	0.50	0.50	0.49
25		ER | GOSE/-Q 7-8 vs. ER | GOSE/-Q 5-6	0.21	0.24	0.23	0.23	0.23	0.19	0.29	0.19	0.25	0.17
26		admission | GOSE/-Q 7-8 vs. admission | GOSE/-Q 5-6	0.28	0.27	0.29	0.29	0.27	0.25	0.29	0.25	0.27	0.24
27	Clinical care pathways	admission | GOSE/-Q 7-8 vs. admission | GOSE/-Q 3-4	0.11	0.13	0.23	0.24	0.15	0.12	0.29	0.25	0.26	0.21
28		admission | GOSE/-Q 5-6 vs. admission | GOSE/-Q 3-4	0.24	0.30	0.42	0.43	0.34	0.30	0.47	0.47	0.46	0.46
29		ICU | GOSE/-Q 7-8 vs. ICU | GOSE/-Q 5-6	0.27	0.28	0.37	0.38	0.32	0.29	0.35	0.33	0.33	0.28
30		ICU | GOSE/-Q 3-4 vs. ICU | GOSE/-Q 3-4	0.12	0.13	0.31	0.29	0.18	0.14	0.30	0.24	0.32	0.22
31		ICU | GOSE/-Q 5-6 vs. ICU | GOSE/-Q 3-4	0.28	0.29	0.43	0.39	0.31	0.29	0.44	0.39	0.48	0.42
32		TBI Sev 1 | GOSE/-Q 7-8 vs. TBI Sev 1 | GOSE/-Q 5-6	0.21	0.22	0.29	0.27	0.24	0.22	0.29	0.23	0.26	0.22
33		TBI Sev 1 | GOSE/-Q 7-8 vs. TBI Sev 1 | GOSE/-Q 3-4	0.10	0.13	0.26	0.25	0.23	0.12	0.26	0.25	0.24	0.21
34		TBI Sev 2 | GOSE/-Q 7-8 vs. TBI Sev 2 | GOSE/-Q 5-6	0.28	0.29	0.30	0.32	0.29	0.28	0.31	0.30	0.29	0.25
35		TBI Sev 2 | GOSE/-Q 7-8 vs. TBI Sev 2 | GOSE/-Q 3-4	0.10	0.14	0.25	0.26	0.14	0.12	0.29	0.21	0.27	0.22
36		TBI Sev 3 | GOSE/-Q 7-8 vs. TBI Sev 3 | GOSE/-Q 5-6	0.28	0.32	0.35	0.33	0.25	0.27	0.39	0.31	0.25	0.27
37	TBI severity	TBI Sev 3 | GOSE/-Q 7-8 vs. TBI Sev 3 | GOSE/-Q 3-4	0.15	0.14	0.22	0.26	0.13	0.11	0.36	0.22	0.25	0.19
38		TBI Sev 4 | GOSE/-Q 7-8 vs. TBI Sev 4 | GOSE/-Q 5-6	0.27	0.28	0.38	0.31	0.32	0.26	0.32	0.26	0.32	0.27
39		TBI Sev 4 | GOSE/-Q 7-8 vs. TBI Sev 4 | GOSE/-Q 3-4	0.13	0.12	0.33	0.27	0.17	0.14	0.26	0.22	0.31	0.20
40		TBI Sev 1 | GOSE/-Q 5-6 vs. TBI Sev 1 | GOSE/-Q 3-4	0.29	0.35	0.46	0.45	0.43	0.31	0.45	0.47	0.45	0.50
41		TBI Sev 2 | GOSE/-Q 5-6 vs. TBI Sev 2 | GOSE/-Q 3-4	0.23	0.30	0.43	0.42	0.30	0.29	0.46	0.41	0.46	0.45
42		TBI Sev 3 | GOSE/-Q 5-6 vs. TBI Sev 3 | GOSE/-Q 3-4	0.30	0.25	0.40	0.42	0.32	0.33	0.46	0.42	0.55	0.39
43		TBI Sev 4 | GOSE/-Q 5-6 vs. TBI Sev 4 | GOSE/-Q 3-4	0.27	0.24	0.44	0.42	0.29	0.30	0.41	0.43	0.49	0.41
44		ISS<10 | GOSE/-Q 7-8 vs. ISS<10 | GOSE/-Q 5-6	0.26	0.26	0.28	0.27	0.25	0.24	0.28	0.22	0.25	0.19
45		ISS<10 | GOSE/-Q 7-8 vs. ISS<10 | GOSE/-Q 3-4	0.12	0.14	0.31	0.29	0.17	0.14	0.31	0.27	0.34	0.24
46	Injury severity score	ISS<10 | GOSE/-Q 5-6 vs. ISS<10 | GOSE/-Q 3-4	0.26	0.30	0.48	0.48	0.38	0.34	0.52	0.53	0.55	0.59
47		ISS10+ | GOSE/-Q 7-8 vs. ISS10+ | GOSE/-Q 5-6	0.27	0.28	0.33	0.34	0.30	0.28	0.33	0.30	0.30	0.25
48		ISS10+ | GOSE/-Q 7-8 vs. ISS10+ | GOSE/-Q 3-4	0.13	0.14	0.27	0.26	0.16	0.14	0.29	0.23	0.29	0.20
49		ISS10+ | GOSE/-Q 5-6 vs. ISS10+ | GOSE/-Q 3-4	0.28	0.30	0.44	0.40	0.32	0.29	0.45	0.41	0.48	0.43

Note. No. = continuous numbering of comparisons; GOSE/-Q = combined information on recovery status (Glasgow Outcome Coma Scale - Extended and its questionnaire version GOSE-Q); ER = emergency room, admission = admission to a hospital ward, ICU = intensive care unit; TBI Sev 1 = uncomplicated mild TBI, TBI Sev 2 = complicated mild TBI, TBI Sev 3 = moderate TBI, TBI Sev 4 = severe TBI; missing pairwise comparisons indicate insufficient number of participants (n ≤ 28); dark red cells = very large effect (beyond 0.20 or 0.80), medium dark red cells with black numbers = more than a large (0.29 | 0.71), but less than a very large effect (0.20 | 0.80), light red cells with black numbers = more than a medium effect (0.36 | 0.64), but less than a large effect (0.29 | 0.71), white cells = small effect (≥ 0.36 | ≤ 0.64). Values below 0.50 indicate a better outcome in patient group 1 compared to group 2. Rounding errors may occur on the second decimal place.

Interpretation example: Three months after TBI, all instruments are moderately to highly sensitive for detecting differences between individuals with good and moderate and good and severe recovery within the TBI severity groups . Since individuals with worse recovery have significantly worse outcomes in all areas, it is recommended to apply these instruments for identifying potential problems and finding appropriate treatment. Given equal sensitivity and for the sake of economy, short versions of instruments (i.e., SF-12v2, QOLIBRI-OS) can be used instead of long versions for screening purposes.

6 months after TBI (data as available)

No.	Variable	Groups	SF-36v2 PCS	SF-12v2 PCS	SF-36v2 MCS	SF-12v2 MCS	QOLIBRI	QOLIBRI-OS	GAD-7	PHQ-9	PCL-5	RPQ
1	Sex	male | GOSE/-Q 7-8 vs. male | GOSE/-Q 5-6	0.32	0.31	0.33	0.33	0.30	0.32	0.36	0.31	0.30	0.28
2		male | GOSE/-Q 7-8 vs. male | GOSE/-Q 3-4	0.16	0.15	0.33	0.33	0.21	0.23	0.32	0.27	0.32	0.22
3		male | GOSE/-Q 5-6 vs. male | GOSE/-Q 3-4	0.29	0.29	0.49	0.48	0.37	0.37	0.48	0.47	0.53	0.44
4		female | GOSE/-Q 7-8 vs. female | GOSE/-Q 5-6	0.36	0.37	0.33	0.34	0.32	0.33	0.32	0.31	0.29	0.22
5		female | GOSE/-Q 7-8 vs. female | GOSE/-Q 3-4	0.22	0.22	0.34	0.36	0.24	0.27	0.35	0.32	0.33	0.26
6		female | GOSE/-Q 5-6 vs. female | GOSE/-Q 3-4	0.31	0.31	0.50	0.50	0.41	0.44	0.53	0.51	0.54	0.56
7	Age	<65 | GOSE/-Q 7-8 vs. <65 | GOSE/-Q 5-6	0.29	0.29	0.34	0.35	0.31	0.31	0.35	0.32	0.31	0.26
8		<65 | GOSE/-Q 7-8 vs. <65 | GOSE/-Q 3-4	0.15	0.15	0.35	0.36	0.22	0.24	0.33	0.29	0.32	0.22
9		<65 | GOSE/-Q 5-6 vs. <65 | GOSE/-Q 3-4	0.31	0.31	0.49	0.49	0.39	0.40	0.48	0.47	0.52	0.46
10		65+ | GOSE/-Q 7-8 vs. 65+ | GOSE/-Q 5-6	0.37	0.35	0.30	0.31	0.27	0.32	0.35	0.28	0.26	0.31
11		65+ | GOSE/-Q 7-8 vs. 65+ | GOSE/-Q 3-4	0.23	0.22	0.29	0.30	0.22	0.24	0.34	0.29	0.32	0.26
12		65+ | GOSE/-Q 5-6 vs. 65+ | GOSE/-Q 3-4	0.32	0.33	0.48	0.48	0.41	0.38	0.50	0.52	0.54	0.47
13	Education	primary and less | GOSE/-Q 7-8 vs. primary and less | GOSE/-Q 5-6	0.35	0.36	0.36	0.40	0.27	0.31	0.33	0.31	0.33	0.27
14		primary and less | GOSE/-Q 7-8 vs. primary and less | GOSE/-Q 3-4	0.20	0.21	0.34	0.37	0.24	0.24	0.35	0.27	0.32	0.20
15		primary and less | GOSE/-Q 5-6 vs. primary and less | GOSE/-Q 3-4	0.32	0.35	0.46	0.45	0.45	0.40	0.53	0.47	0.50	0.45
16		at least secondary | GOSE/-Q 7-8 vs. at least secondary | GOSE/-Q 5-6	0.31	0.31	0.31	0.32	0.29	0.31	0.34	0.29	0.29	0.25
17		at least secondary | GOSE/-Q 7-8 vs. at least secondary | GOSE/-Q 3-4	0.17	0.16	0.34	0.34	0.22	0.25	0.33	0.29	0.33	0.23
18		at least secondary | GOSE/-Q 5-6 vs. at least secondary | GOSE/-Q 3-4	0.30	0.30	0.51	0.50	0.39	0.41	0.50	0.51	0.55	0.50
19	Premorbid psych. Problems	absent | GOSE/-Q 7-8 vs. absent | GOSE/-Q 5-6	0.34	0.34	0.32	0.34	0.32	0.32	0.34	0.30	0.29	0.26
20		absent | GOSE/-Q 7-8 vs. absent | GOSE/-Q 3-4	0.17	0.17	0.32	0.34	0.22	0.23	0.32	0.28	0.32	0.22
21		absent | GOSE/-Q 5-6 vs. absent | GOSE/-Q 3-4	0.28	0.29	0.48	0.49	0.37	0.38	0.48	0.48	0.52	0.46
22		present | GOSE/-Q 7-8 vs. present | GOSE/-Q 5-6	0.29	0.30	0.36	0.38	0.31	0.32	0.39	0.34	0.30	0.26
23		present | GOSE/-Q 3-4 vs. present | GOSE/-Q 3-4	0.21	0.20	0.42	0.44	0.30	0.34	0.39	0.36	0.37	0.29
24		present | GOSE/-Q 5-6 vs. present | GOSE/-Q 3-4	0.38	0.36	0.55	0.55	0.47	0.49	0.51	0.52	0.58	0.57
25	Clinical care pathways	ER | GOSE/-Q 7-8 vs. ER | GOSE/-Q 5-6	0.31	0.32	0.30	0.32	0.26	0.27	0.30	0.26	0.29	0.19
26		admission | GOSE/-Q 7-8 vs. admission | GOSE/-Q 5-6	0.32	0.33	0.32	0.32	0.31	0.33	0.31	0.27	0.28	0.25
27		admission | GOSE/-Q 7-8 vs. admission | GOSE/-Q 3-4	0.18	0.16	0.31	0.33	0.21	0.21	0.29	0.25	0.25	0.25
28		admission | GOSE/-Q 5-6 vs. admission | GOSE/-Q 3-4	0.28	0.27	0.49	0.49	0.38	0.35	0.49	0.48	0.48	0.51
29		ICU | GOSE/-Q 7-8 vs. ICU | GOSE/-Q 5-6	0.33	0.33	0.33	0.34	0.31	0.31	0.38	0.34	0.33	0.32
30		ICU | GOSE/-Q 3-4 vs. ICU | GOSE/-Q 3-4	0.16	0.16	0.33	0.34	0.21	0.23	0.34	0.29	0.36	0.26
31		ICU | GOSE/-Q 5-6 vs. ICU | GOSE/-Q 3-4	0.29	0.29	0.48	0.48	0.37	0.39	0.47	0.46	0.53	0.45
32	TBI severity	TBI Sev 1 | GOSE/-Q 7-8 vs. TBI Sev 1 | GOSE/-Q 5-6	0.29	0.30	0.34	0.34	0.29	0.32	0.33	0.30	0.31	0.26
33		TBI Sev 2 | GOSE/-Q 7-8 vs. TBI Sev 2 | GOSE/-Q 5-6	0.35	0.35	0.32	0.34	0.32	0.32	0.36	0.31	0.28	0.28
34		TBI Sev 2 | GOSE/-Q 7-8 vs. TBI Sev 2 | GOSE/-Q 3-4	0.16	0.16	0.31	0.33	0.21	0.22	0.31	0.24	0.29	0.24
35		TBI Sev 3 | GOSE/-Q 7-8 vs. TBI Sev 3 | GOSE/-Q 5-6	0.32	0.32	0.31	0.32	0.25	0.28	0.32	0.28	0.25	0.31
36		TBI Sev 3 | GOSE/-Q 7-8 vs. TBI Sev 3 | GOSE/-Q 3-4	0.17	0.14	0.29	0.28	0.14	0.24	0.30	0.24	0.26	0.22
37		TBI Sev 4 | GOSE/-Q 7-8 vs. TBI Sev 4 | GOSE/-Q 5-6	0.36	0.32	0.30	0.32	0.30	0.28	0.37	0.28	0.34	0.28
38		TBI Sev 4 | GOSE/-Q 7-8 vs. TBI Sev 4 | GOSE/-Q 3-4	0.16	0.14	0.32	0.34	0.20	0.22	0.34	0.27	0.39	0.24
39		TBI Sev 2 | GOSE/-Q 5-6 vs. TBI Sev 2 | GOSE/-Q 3-4	0.24	0.24	0.46	0.47	0.35	0.35	0.45	0.42	0.51	0.44
40		TBI Sev 3 | GOSE/-Q 5-6 vs. TBI Sev 3 | GOSE/-Q 3-4	0.28	0.27	0.47	0.44	0.36	0.44	0.47	0.47	0.51	0.42
41		TBI Sev 4 | GOSE/-Q 5-6 vs. TBI Sev 4 | GOSE/-Q 3-4	0.28	0.28	0.50	0.50	0.39	0.40	0.47	0.49	0.53	0.45
42	Injury Severity Score	ISS<10 | GOSE/-Q 7-8 vs. ISS<10 | GOSE/-Q 5-6	0.32	0.31	0.32	0.34	0.28	0.30	0.30	0.30	0.28	0.23
43		ISS10+ | GOSE/-Q 7-8 vs. ISS10+ | GOSE/-Q 5-6	0.34	0.34	0.32	0.33	0.31	0.32	0.35	0.30	0.30	0.28
44		ISS10+ | GOSE/-Q 7-8 vs. ISS10+ | GOSE/-Q 3-4	0.17	0.17	0.32	0.34	0.22	0.24	0.32	0.28	0.32	0.24
45		ISS10+ | GOSE/-Q 5-6 vs. ISS10+ | GOSE/-Q 3-4	0.29	0.29	0.48	0.48	0.37	0.38	0.47	0.47	0.51	0.45

Note. No. = continuous numbering of comparisons; GOSE/-Q = combined information on recovery status (Glasgow Outcome Coma Scale - Extended and its questionnaire version GOSE-Q); ER = emergency room, admission = admission to a hospital ward, ICU = intensive care unit; TBI Sev 1 = uncomplicated mild TBI, TBI Sev 2 = complicated mild TBI, TBI Sev 3 = moderate TBI, TBI Sev 4 = severe TBI; missing pairwise comparisons indicate insufficient number of participants (n ≤ 28); dark red cells = very large effect (beyond 0.20 or 0.80), medium dark red cells with black numbers = more than a large (0.29 | 0.71), but less than a very large effect (0.20 | 0.80), light red cells with black numbers = more than a medium effect (0.36 | 0.64), but less than a large effect (0.29 | 0.71), white cells = small effect (≥ 0.36 | ≤ 0.64). Values below 0.50 indicate a better outcome in patient group 1 compared to group 2. Rounding errors may occur on the second decimal place.

<u>Interpretation example:</u> Six months after TBI, all instruments are moderately to highly sensitive for detecting differences between individuals with good and moderate and good and severe recovery within the TBI severity groups . Since individuals with worse recovery have significantly worse outcomes in all areas, it is recommended to apply these instruments for identifying potential problems and finding appropriate treatment. Given equal sensitivity and for the sake of economy, short versions of instruments (i.e., SF-12v2, QOLIBRI-OS) can be used instead of long versions for screening purposes.

12 months after TBI (data as available)

No.	Variable	Groups	SF-36v2 PCS	SF-12v2 PCS	SF-36v2 MCS	SF-12v2 MCS	QOLIBRI	QOLIBRI-OS	GAD-7	PHQ-9	PCL-5	RPQ
1		male | GOSE/-Q 7-8 vs. male | GOSE/-Q 5-6	0.33	0.35	0.35	0.34	0.32	0.32	0.36	0.32	0.31	0.27
2		male | GOSE/-Q 7-8 vs. male | GOSE/-Q 3-4	0.19	0.18	0.26	0.29	0.19	0.21	0.31	0.25	0.25	0.21
3	Sex	male | GOSE/-Q 5-6 vs. male | GOSE/-Q 3-4	0.32	0.31	0.41	0.44	0.33	0.36	0.45	0.43	0.44	0.42
4		female | GOSE/-Q 7-8 vs. female | GOSE/-Q 5-6	0.44	0.44	0.38	0.34	0.34	0.38	0.36	0.34	0.34	0.28
5		female | GOSE/-Q 7-8 vs. female | GOSE/-Q 3-4	0.24	0.22	0.33	0.33	0.21	0.23	0.35	0.27	0.31	0.28
6		female | GOSE/-Q 5-6 vs. female | GOSE/-Q 3-4	0.26	0.25	0.45	0.48	0.35	0.35	0.49	0.43	0.47	0.50
7		<65 | GOSE/-Q 7-8 vs. <65 | GOSE/-Q 5-6	0.32	0.33	0.36	0.34	0.32	0.32	0.37	0.33	0.32	0.27
8		<65 | GOSE/-Q 7-8 vs. <65 | GOSE/-Q 3-4	0.16	0.15	0.28	0.28	0.18	0.19	0.33	0.25	0.26	0.21
9	Age	<65 | GOSE/-Q 5-6 vs. <65 | GOSE/-Q 3-4	0.30	0.29	0.42	0.44	0.33	0.35	0.46	0.42	0.44	0.42
10		65+ | GOSE/-Q 7-8 vs. 65+ | GOSE/-Q 5-6	0.39	0.39	0.37	0.37	0.31	0.37	0.37	0.33	0.34	0.34
11		65+ | GOSE/-Q 7-8 vs. 65+ | GOSE/-Q 3-4	0.26	0.26	0.30	0.36	0.23	0.28	0.30	0.26	0.29	0.29
12		65+ | GOSE/-Q 5-6 vs. 65+ | GOSE/-Q 3-4	0.37	0.38	0.40	0.47	0.36	0.38	0.44	0.42	0.40	0.45
13		primary and less | GOSE/-Q 7-8 vs. primary and less | GOSE/-Q 5-6	0.31	0.30	0.43	0.44	0.31	0.35	0.42	0.34	0.40	0.29
14		primary and less | GOSE/-Q 7-8 vs. primary and less | GOSE/-Q 3-4	0.20	0.20	0.26	0.28	0.19	0.20	0.33	0.25	0.28	0.19
15	Education	primary and less | GOSE/-Q 5-6 vs. primary and less | GOSE/-Q 3-4	0.33	0.38	0.35	0.34	0.38	0.33	0.40	0.43	0.38	0.39
16		at least secondary | GOSE/-Q 7-8 vs. at least secondary | GOSE/-Q 5-6	0.37	0.39	0.34	0.33	0.32	0.33	0.34	0.32	0.30	0.26
17		at least secondary | GOSE/-Q 7-8 vs. at least secondary | GOSE/-Q 3-4	0.21	0.19	0.29	0.30	0.19	0.21	0.31	0.26	0.27	0.24
18		at least secondary | GOSE/-Q 5-6 vs. at least secondary | GOSE/-Q 3-4	0.30	0.28	0.45	0.47	0.34	0.36	0.47	0.44	0.46	0.46
19		absent | GOSE/-Q 7-8 vs. absent | GOSE/-Q 5-6	0.37	0.39	0.38	0.36	0.34	0.34	0.37	0.34	0.33	0.29
20		absent | GOSE/-Q 7-8 vs. absent | GOSE/-Q 3-4	0.19	0.19	0.27	0.27	0.19	0.21	0.30	0.25	0.28	0.22
21	Premorbid. psych. problems	absent | GOSE/-Q 5-6 vs. absent | GOSE/-Q 3-4	0.29	0.28	0.39	0.41	0.32	0.34	0.42	0.41	0.45	0.42
22		present | GOSE/-Q 7-8 vs. present | GOSE/-Q 5-6	0.38	0.38	0.33	0.33	0.31	0.31	0.35	0.32	0.31	0.26
23		present | GOSE/-Q 3-4 vs. present | GOSE/-Q 3-4	0.25	0.24	0.38	0.42	0.26	0.30	0.42	0.32	0.33	0.24
24		present | GOSE/-Q 5-6 vs. present | GOSE/-Q 3-4	0.35	0.35	0.55	0.57	0.42	0.46	0.57	0.50	0.53	0.51
25		admission | GOSE/-Q 7-8 vs. admission | GOSE/-Q 5-6	0.36	0.36	0.36	0.35	0.33	0.35	0.36	0.32	0.34	0.25
26		admission | GOSE/-Q 7-8 vs. admission | GOSE/-Q 3-4	0.18	0.20	0.25	0.26	0.16	0.17	0.28	0.21	0.19	0.22
27	Clinical care pathways	admission | GOSE/-Q 5-6 vs. admission | GOSE/-Q 3-4	0.28	0.32	0.38	0.40	0.29	0.28	0.41	0.39	0.35	0.45
28		ICU | GOSE/-Q 7-8 vs. ICU | GOSE/-Q 5-6	0.35	0.38	0.36	0.34	0.32	0.31	0.36	0.34	0.32	0.32
29		ICU | GOSE/-Q 3-4 vs. ICU | GOSE/-Q 3-4	0.18	0.17	0.29	0.31	0.19	0.21	0.33	0.27	0.31	0.26
30		ICU | GOSE/-Q 5-6 vs. ICU | GOSE/-Q 3-4	0.29	0.27	0.44	0.47	0.35	0.38	0.47	0.43	0.48	0.44
31		TBI Sev 1 | GOSE/-Q 7-8 vs. TBI Sev 1 | GOSE/-Q 5-6	0.29	0.31	0.39	0.41	0.33	0.33	0.40	0.33	0.35	0.27
32		TBI Sev 2 | GOSE/-Q 7-8 vs. TBI Sev 2 | GOSE/-Q 5-6	0.37	0.39	0.34	0.30	0.32	0.35	0.31	0.31	0.30	0.27
33		TBI Sev 2 | GOSE/-Q 7-8 vs. TBI Sev 2 | GOSE/-Q 3-4	0.14	0.15	0.27	0.26	0.15	0.16	0.29	0.23	0.26	0.20
34	TBI severity	TBI Sev 4 | GOSE/-Q 7-8 vs. TBI Sev 4 | GOSE/-Q 5-6	0.32	0.36	0.35	0.33	0.30	0.28	0.38	0.33	0.33	0.32
35		TBI Sev 4 | GOSE/-Q 7-8 vs. TBI Sev 4 | GOSE/-Q 3-4	0.17	0.17	0.31	0.31	0.18	0.18	0.36	0.29	0.35	0.25
36		TBI Sev 1 | GOSE/-Q 5-6 vs. TBI Sev 1 | GOSE/-Q 3-4	0.34	0.31	0.43	0.41	0.42	0.38	0.42	0.38	0.34	0.48
37		TBI Sev 2 | GOSE/-Q 5-6 vs. TBI Sev 2 | GOSE/-Q 3-4	0.23	0.24	0.43	0.45	0.31	0.29	0.49	0.42	0.47	0.43
38		TBI Sev 4 | GOSE/-Q 5-6 vs. TBI Sev 4 | GOSE/-Q 3-4	0.28	0.27	0.47	0.49	0.35	0.40	0.49	0.46	0.51	0.41
39		ISS<10 | GOSE/-Q 7-8 vs. ISS<10 | GOSE/-Q 5-6	0.36	0.37	0.33	0.32	0.30	0.34	0.35	0.35	0.29	0.22
40	Injury severity score	ISS10+ | GOSE/-Q 7-8 vs. ISS10+ | GOSE/-Q 5-6	0.37	0.38	0.36	0.35	0.33	0.33	0.36	0.32	0.32	0.29
41		ISS10+ | GOSE/-Q 7-8 vs. ISS10+ | GOSE/-Q 3-4	0.20	0.19	0.28	0.30	0.19	0.21	0.31	0.24	0.27	0.23
42		ISS10+ | GOSE/-Q 5-6 vs. ISS10+ | GOSE/-Q 3-4	0.30	0.29	0.42	0.45	0.34	0.36	0.45	0.43	0.44	0.43

Note. No. = continuous numbering of comparisons; GOSE/-Q = combined information on recovery status (Glasgow Outcome Coma Scale - Extended and its questionnaire version GOSE-Q); ER = emergency room, admission = admission to a hospital ward, ICU = intensive care unit; TBI Sev 1 = uncomplicated mild TBI, TBI Sev 2 = complicated mild TBI, TBI Sev 3 = moderate TBI, TBI Sev 4 = severe TBI; missing pairwise comparisons indicate insufficient number of participants (n ≤ 28); dark red cells = very large effect (beyond 0.20 or 0.80),medium dark red cells with black numbers = more than a large (0.29 | 0.71), but less than a very large effect (0.20 | 0.80), light red cells with black numbers = more than a medium effect (0.36 | 0.64), but less than a large effect (0.29 | 0.71), white cells = small effect (≥ 0.36 | ≤ 0.64). Values below 0.50 indicate a better outcome in patient group 1 compared to group 2. Rounding errors may occur on the second decimal place.

Interpretation example: Twelve months after TBI, all instruments are moderately to highly sensitive for detecting differences between individuals with good and moderate and good and severe recovery within the complicated mTBI group. Since individuals with worse recovery have significantly worse outcomes in all areas, it is recommended to apply these instruments for identifying potential problems and finding appropriate treatment. Given equal sensitivity and for the sake of economy, short versions of instruments (i.e., SF-12v2, QOLIBRI-OS) can be used instead of long versions for screening purposes.
