# Supplementary material for: Sensitivity of outcome instruments in a priori selected patient groups after traumatic brain injury: Results from the CENTER-TBI study
Source: PLoS One. 2023 Apr 7;18(4):e0280796. doi: 10.1371/journal.pone.0280796 (PMC10081802; doi:10.1371/journal.pone.0280796)
Supplement: S9 Table — (PDF) [file pone.0280796.s009.pdf]

S8 Table. Number of individuals with impaired outcomes with respect to instruments' cut-off values at three, six, and twelve months after TBI stratified by sociodemographic, premorbid, and injury-related factors.

		Three months after TBI										
Group	Values	GOSE/-Q	SF-36v2 PCS	SF-12v2 PCS	SF-36v2 MCS	SF-12v2 MCS	QOLIBRI	QOLIBRI-OS	GAD-7	PHQ-9	PCL-5	RPQ
Sex	male	878 (67.3%)	360 (57.4%)	490 (59.3%)	335 (58.2%)	357 (57.5%)	327 (60.1%)	341 (60.9%)	136 (55.5%)	237 (58.4%)	126 (57.5%)	497 (60.1%)
	female	426 (32.7%)	267 (42.6%)	337 (40.7%)	241 (41.8%)	264 (42.5%)	217 (39.9%)	219 (39.1%)	109 (44.5%)	169 (41.6%)	93 (42.5%)	330 (39.9%)
Age	< 65	1024 (78.5%)	428 (68.3%)	555 (67.1%)	454 (78.8%)	474 (76.3%)	408 (75.0%)	410 (73.2%)	197 (80.4%)	311 (76.6%)	186 (84.9%)	658 (79.6%)
	65 +	280 (21.5%)	199 (31.7%)	272 (32.9%)	122 (21.2%)	147 (23.7%)	136 (25.0%)	150 (26.8%)	48 (19.6%)	95 (23.4%)	33 (15.1%)	169 (20.4%)
Education	≤ primary	204 (15.6%)	129 (20.6%)	160 (19.3%)	105 (18.2%)	110 (17.7%)	110 (20.2%)	110 (19.6%)	51 (20.8%)	80 (19.7%)	48 (21.9%)	130 (15.7%)
	secondary +	898 (68.9%)	430 (68.6%)	572 (69.2%)	422 (73.3%)	447 (72.0%)	374 (68.8%)	389 (69.5%)	176 (71.8%)	278 (68.5%)	153 (69.9%)	613 (74.1%)
	missing	202 (15.5%)	68 (10.8%)	95 (11.5%)	49 (8.5%)	64 (10.3%)	60 (11.0%)	61 (10.9%)	18 (7.3%)	48 (11.8%)	18 (8.2%)	84 (10.2%)
Premorbid psych. problems	absent	623 (47.8%)	351 (56.0%)	471 (57.0%)	268 (46.5%)	274 (44.1%)	252 (46.3%)	254 (45.4%)	99 (40.4%)	182 (44.8%)	85 (38.8%)	438 (53.0%)
	present	194 (14.9%)	124 (19.8%)	147 (17.8%)	155 (26.9%)	162 (26.1%)	136 (25.0%)	122 (21.8%)	79 (32.2%)	118 (29.1%)	70 (32.0%)	182 (22.0%)
	missing	487 (37.3%)	152 (24.2%)	209 (25.3%)	153 (26.6%)	185 (29.8%)	156 (28.7%)	184 (32.9%)	67 (27.3%)	106 (26.1%)	64 (29.2%)	207 (25.0%)
Clinical care pathways	ER	89 (6.8%)	94 (15.0%)	123 (14.9%)	110 (19.1%)	116 (18.7%)	93 (17.1%)	89 (15.9%)	57 (23.3%)	83 (20.4%)	49 (22.4%)	127 (15.4%)
	ward	318 (24.4%)	207 (33.0%)	267 (32.3%)	195 (33.9%)	215 (34.6%)	186 (34.2%)	168 (30.0%)	86 (35.1%)	143 (35.2%)	85 (38.8%)	297 (35.9%)
	ICU	897 (68.8%)	326 (52.0%)	437 (52.8%)	271 (47.0%)	290 (46.7%)	265 (48.7%)	303 (54.1%)	102 (41.6%)	180 (44.3%)	85 (38.8%)	403 (48.7%)
Injury severity score (ISS)	<10	199 (15.3%)	173 (27.6%)	222 (26.8%)	183 (31.8%)	207 (33.3%)	172 (31.6%)	163 (29.1%)	96 (39.2%)	148 (36.5%)	81 (37.0%)	254 (30.7%)
	10+	1093 (83.8%)	447 (71.3%)	595 (71.9%)	388 (67.4%)	406 (65.4%)	367 (67.5%)	392 (70.0%)	145 (59.2%)	254 (62.6%)	135 (61.6%)	564 (68.2%)
	missing	12 (0.9%)	7 (1.1%)	10 (1.2%)	5 (0.9%)	8 (1.3%)	5 (0.9%)	5 (0.9%)	4 (1.6%)	4 (1.0%)	3 (1.4%)	9 (1.1%)
TBI Severity	uncomplicated	208 (16.0%)	168 (26.8%)	213 (25.8%)	160 (27.8%)	173 (27.9%)	137 (25.2%)	138 (24.6%)	71 (29.0%)	114 (28.1%)	73 (33.3%)	206 (24.9%)
	complicated	342 (26.2%)	173 (27.6%)	236 (28.5%)	179 (31.1%)	196 (31.6%)	160 (29.4%)	135 (24.1%)	73 (29.8%)	114 (28.1%)	63 (28.8%)	256 (31.0%)
	moderate	125 (9.6%)	40 (6.4%)	52 (6.3%)	36 (6.2%)	42 (6.8%)	32 (5.9%)	31 (5.5%)	10 (4.1%)	20 (4.9%)	10 (4.6%)	53 (6.4%)
	severe	345 (26.5%)	101 (16.1%)	141 (17.0%)	81 (14.1%)	78 (12.6%)	90 (16.5%)	113 (20.2%)	31 (12.7%)	62 (15.3%)	28 (12.8%)	131 (15.8%)
	missing	284 (21.8%)	145 (23.1%)	185 (22.4%)	120 (20.8%)	132 (21.3%)	125 (23.0%)	143 (25.5%)	60 (24.5%)	96 (23.6%)	45 (20.5%)	181 (21.9%)
	Total	1304 (45.9%)	627 (30.2%)	827 (36.1%)	576 (27.8%)	621 (27.1%)	544 (25.7%)	560 (23.9%)	245 (11.7%)	406 (19.4%)	219 (10.5%)	827 (38.3%)

		Six months after TBI										
Group	Values	GOSE/-Q	SF-36v2 PCS	SF-12v2 PCS	SF-36v2 MCS	SF-12v2 MCS	QOLIBRI	QOLIBRI-OS	GAD-7	PHQ-9	PCL-5	RPQ
Sex	male	732 (66.6%)	319 (58.9%)	383 (58.0%)	346 (59.0%)	316 (57.5%)	315 (59.3%)	293 (57.5%)	136 (57.1%)	222 (57.8%)	127 (63.5%)	503 (58.9%)
	female	367 (33.4%)	223 (41.1%)	277 (42.0%)	240 (41.0%)	234 (42.5%)	216 (40.7%)	217 (42.5%)	102 (42.9%)	162 (42.2%)	73 (36.5%)	351 (41.1%)
Age	< 65	863 (78.5%)	368 (67.9%)	442 (67.0%)	463 (79.0%)	433 (78.7%)	405 (76.3%)	389 (76.3%)	194 (81.5%)	310 (80.7%)	176 (88.0%)	689 (80.7%)
	65 +	236 (21.5%)	174 (32.1%)	218 (33.0%)	123 (21.0%)	117 (21.3%)	126 (23.7%)	121 (23.7%)	44 (18.5%)	74 (19.3%)	24 (12.0%)	165 (19.3%)
Education	≤ primary	174 (15.8%)	106 (19.6%)	131 (19.8%)	94 (16.0%)	91 (16.5%)	99 (18.6%)	94 (18.4%)	47 (19.7%)	69 (18.0%)	34 (17.0%)	133 (15.6%)
	secondary +	744 (67.7%)	373 (68.8%)	450 (68.2%)	426 (72.7%)	400 (72.7%)	367 (69.1%)	359 (70.4%)	169 (71.0%)	271 (70.6%)	148 (74.0%)	622 (72.8%)
	missing	181 (16.5%)	63 (11.6%)	79 (12.0%)	66 (11.3%)	59 (10.7%)	65 (12.2%)	57 (11.2%)	22 (9.2%)	44 (11.5%)	18 (9.0%)	99 (11.6%)
Premorbid psych. Problems	absent	571 (52.0%)	349 (64.4%)	426 (64.5%)	329 (56.1%)	288 (52.4%)	294 (55.4%)	284 (55.7%)	117 (49.2%)	192 (50.0%)	95 (47.5%)	517 (60.5%)
	present	195 (17.7%)	128 (23.6%)	140 (21.2%)	181 (30.9%)	172 (31.3%)	165 (31.1%)	143 (28.0%)	91 (38.2%)	139 (36.2%)	81 (40.5%)	223 (26.1%)
	missing	333 (30.3%)	65 (12.0%)	94 (14.2%)	76 (13.0%)	90 (16.4%)	72 (13.6%)	83 (16.3%)	30 (12.6%)	53 (13.8%)	24 (12.0%)	114 (13.3%)
Clinical care pathways	ER	71 (6.5%)	85 (15.7%)	112 (17.0%)	102 (17.4%)	101 (18.4%)	80 (15.1%)	91 (17.8%)	46 (19.3%)	73 (19.0%)	40 (20.0%)	120 (14.1%)
	ward	246 (22.4%)	170 (31.4%)	206 (31.2%)	190 (32.4%)	183 (33.3%)	163 (30.7%)	159 (31.2%)	72 (30.3%)	124 (32.3%)	76 (38.0%)	283 (33.1%)
	ICU	782 (71.2%)	287 (53.0%)	342 (51.8%)	294 (50.2%)	266 (48.4%)	288 (54.2%)	260 (51.0%)	120 (50.4%)	187 (48.7%)	84 (42.0%)	451 (52.8%)
Injury severity score (ISS)	<10	164 (14.9%)	160 (29.5%)	193 (29.2%)	198 (33.8%)	189 (34.4%)	169 (31.8%)	164 (32.2%)	83 (34.9%)	125 (32.6%)	76 (38.0%)	249 (29.2%)
	10+	925 (84.2%)	371 (68.5%)	455 (68.9%)	380 (64.8%)	355 (64.5%)	357 (67.2%)	339 (66.5%)	151 (63.4%)	256 (66.7%)	121 (60.5%)	593 (69.4%)
	missing	10 (0.9%)	11 (2.0%)	12 (1.8%)	8 (1.4%)	6 (1.1%)	5 (0.9%)	7 (1.4%)	4 (1.7%)	3 (0.8%)	3 (1.5%)	12 (1.4%)
TBI Severity	uncomplicated	159 (14.5%)	144 (26.6%)	179 (27.1%)	154 (26.3%)	154 (28.0%)	122 (23.0%)	139 (27.3%)	58 (24.4%)	110 (28.6%)	62 (31.0%)	210 (24.6%)
	complicated	303 (27.6%)	162 (29.9%)	193 (29.2%)	186 (31.7%)	177 (32.2%)	156 (29.4%)	142 (27.8%)	79 (33.2%)	106 (27.6%)	61 (30.5%)	281 (32.9%)
	moderate	111 (10.1%)	37 (6.8%)	43 (6.5%)	35 (6.0%)	28 (5.1%)	40 (7.5%)	29 (5.7%)	18 (7.6%)	30 (7.8%)	16 (8.0%)	63 (7.4%)
	severe	312 (28.4%)	94 (17.3%)	120 (18.2%)	92 (15.7%)	87 (15.8%)	97 (18.3%)	93 (18.2%)	40 (16.8%)	59 (15.4%)	23 (11.5%)	140 (16.4%)
	missing	214 (19.5%)	105 (19.4%)	125 (18.9%)	119 (20.3%)	104 (18.9%)	116 (21.8%)	107 (21.0%)	43 (18.1%)	79 (20.6%)	38 (19.0%)	160 (18.7%)
	Total	1099 (45.9%)	542 (24.7%)	660 (28.7%)	586 (26.7%)	550 (23.9%)	531 (24.3%)	510 (22.0%)	238 (10.9%)	384 (17.6%)	200 (9.21%)	854 (37.8%)

		Twelve months after TBI										
Group	Values	GOSE/-Q	SF-36v2 PCS	SF-12v2 PCS	SF-36v2 MCS	SF-12v2 MCS	QOLIBRI	QOLIBRI-OS	GAD-7	PHQ-9	PCL-5	RPQ
Sex	male	534 (66.7%)	192 (59.4%)	258 (62.0%)	232 (61.2%)	245 (62.5%)	218 (62.1%)	221 (64.4%)	83 (55.7%)	160 (61.3%)	99 (66.9%)	345 (61.3%)
	female	267 (33.3%)	131 (40.6%)	158 (38.0%)	147 (38.8%)	147 (37.5%)	133 (37.9%)	122 (35.6%)	66 (44.3%)	101 (38.7%)	49 (33.1%)	218 (38.7%)
Age	< 65	621 (77.5%)	206 (63.8%)	260 (62.5%)	308 (81.3%)	324 (82.7%)	278 (79.2%)	269 (78.4%)	126 (84.6%)	224 (85.8%)	132 (89.2%)	462 (82.1%)
	65 +	180 (22.5%)	117 (36.2%)	156 (37.5%)	71 (18.7%)	68 (17.3%)	73 (20.8%)	74 (21.6%)	23 (15.4%)	37 (14.2%)	16 (10.8%)	101 (17.9%)
Education	≤ primary	131 (16.4%)	62 (19.2%)	78 (18.8%)	61 (16.1%)	55 (14.0%)	67 (19.1%)	55 (16.0%)	24 (16.1%)	35 (13.4%)	21 (14.2%)	80 (14.2%)
	secondary +	522 (65.2%)	221 (68.4%)	279 (67.1%)	281 (74.1%)	289 (73.7%)	242 (68.9%)	245 (71.4%)	112 (75.2%)	195 (74.7%)	107 (72.3%)	414 (73.5%)
	missing	148 (18.5%)	40 (12.4%)	59 (14.2%)	37 (9.8%)	48 (12.2%)	42 (12.0%)	43 (12.5%)	13 (8.7%)	31 (11.9%)	20 (13.5%)	69 (12.3%)
Premorbid psych. Problems	absent	390 (48.7%)	185 (57.3%)	248 (59.6%)	208 (54.9%)	212 (54.1%)	181 (51.6%)	172 (50.1%)	63 (42.3%)	120 (46.0%)	65 (43.9%)	319 (56.7%)
	present	136 (17.0%)	58 (18.0%)	70 (16.8%)	96 (25.3%)	94 (24.0%)	91 (25.9%)	78 (22.7%)	51 (34.2%)	85 (32.6%)	45 (30.4%)	137 (24.3%)
	missing	275 (34.3%)	80 (24.8%)	98 (23.6%)	75 (19.8%)	86 (21.9%)	79 (22.5%)	93 (27.1%)	35 (23.5%)	56 (21.5%)	38 (25.7%)	107 (19.0%)
Clinical care pathways	ER	0 (0%)[a]	0 (0%)[1]	0 (0%)[a]	0 (0%)[a]	0 (0%)[a]	0 (0%)[a]	0 (0%)[a]	0 (0%)[a]	0 (0%)[a]	0 (0%)[a]	0 (0%)[a]
	ward	185 (23.1%)	135 (41.8%)	167 (40.1%)	143 (37.7%)	137 (34.9%)	128 (36.5%)	121 (35.3%)	57 (38.3%)	90 (34.5%)	46 (31.1%)	206 (36.6%)
	ICU	616 (76.9%)	188 (58.2%)	249 (59.9%)	236 (62.3%)	255 (65.1%)	223 (63.5%)	222 (64.7%)	92 (61.7%)	171 (65.5%)	102 (68.9%)	357 (63.4%)
Injury severity score (ISS)	<10	88 (11.0%)	60 (18.6%)	78 (18.8%)	81 (21.4%)	74 (18.9%)	64 (18.2%)	65 (19.0%)	27 (18.1%)	45 (17.2%)	27 (18.2%)	105 (18.7%)
	10+	699 (87.3%)	252 (78.0%)	327 (78.6%)	292 (77.0%)	312 (79.6%)	281 (80.1%)	272 (79.3%)	117 (78.5%)	209 (80.1%)	117 (79.1%)	448 (79.6%)
	missing	14 (1.7%)	11 (3.4%)	11 (2.6%)	6 (1.6%)	6 (1.5%)	6 (1.7%)	6 (1.7%)	5 (3.4%)	7 (2.7%)	4 (2.7%)	10 (1.8%)
TBI Severity	uncomplicated	82 (10.2%)	65 (20.1%)	76 (18.3%)	55 (14.5%)	58 (14.8%)	48 (13.7%)	54 (15.7%)	20 (13.4%)	37 (14.2%)	18 (12.2%)	89 (15.8%)
	complicated	215 (26.8%)	108 (33.4%)	139 (33.4%)	131 (34.6%)	145 (37.0%)	118 (33.6%)	100 (29.2%)	53 (35.6%)	92 (35.2%)	51 (34.5%)	192 (34.1%)
	moderate	90 (11.2%)	31 (9.6%)	32 (7.7%)	29 (7.7%)	31 (7.9%)	25 (7.1%)	30 (8.7%)	11 (7.4%)	18 (6.9%)	13 (8.8%)	52 (9.2%)
	severe	258 (32.2%)	61 (18.9%)	94 (22.6%)	92 (24.3%)	97 (24.7%)	87 (24.8%)	98 (28.6%)	36 (24.2%)	66 (25.3%)	41 (27.7%)	125 (22.2%)
	missing	156 (19.5%)	58 (18.0%)	75 (18.0%)	72 (19.0%)	61 (15.6%)	73 (20.8%)	61 (17.8%)	29 (19.5%)	48 (18.4%)	25 (16.9%)	105 (18.7%)
	Total	801 (40.5%)	323 (22.5%)	416 (27.1%)	379 (26.4%)	392 (25.5%)	351 (24.1%)	343 (21.7%)	149 (10.2%)	261 (17.9%)	148 (10.2%)	563 (37.3%)

Grey-shaded cells indicate groups with the highest number of impaired individuals. ER = emergency room; ICU = intensive care unit; TBI = traumatic brain injury; N = number of observations; % = percentage. The following cut-off points determine impairment: GOSE/-Q < 7; SF-36v2/-12v2 < 40 (long and short version); QOLIBRI < 60; QOLIBRI-OS < 54; GAD-7 ≥ 10; PHQ-9 ≥ 10; PCL-5 ≥ 33, RPQ ≥ 12.

[a] Based on the study design, participants seen in the emergency room (ER) and then discharged were not included in the twelve-months follow-up assessments.
