# Supplementary material for: Sensitivity of outcome instruments in a priori selected patient groups after traumatic brain injury: Results from the CENTER-TBI study
Source: PLoS One. 2023 Apr 7;18(4):e0280796. doi: 10.1371/journal.pone.0280796 (PMC10081802; doi:10.1371/journal.pone.0280796)
Supplement: S1 Text — (PDF) [file pone.0280796.s010.pdf]

Sensitivity of outcome instruments in a priori patient groups after traumatic brain injury: results from the CENTER-TBI study – Online Supplement

Abbreviations/notations

Abbreviation	Explanation
TBI	Traumatic brain injury
MW	Mann-Whitney effect size
CI	Confidence interval
ISS	Injury Severity score
ER	Emergency room
ADMISSION	Admission to hospital ward
GOSE/-Q	Glasgow Outcome Scale – Extended combined with its questionnaire version GOSE/-Q
SF36MCS	SF-36v2 Mental Component Score
SF12MCS	SF-12v2 Mental Component Score
SF36PCS	SF-36v2 Physical Component Score
SF12PCS	SF-12v2 Physical Component Score
QOLIBRI	QoLIBRI total score
QOLI-OS	QoLIBRI-OS total score
GAD7	GAD-7 total score
PHQ9	PHQ-9 total score
PCL5	PCL-5 total score
RPQ	RPQ total score

Example

Comparisons of QOLIBRI scores between well and moderately recovered males (GOSE/-Q 7-8 vs GOSE/-Q 5-6) at three months after TBI yielded MW = 0.2764 with $p < .001$, indicating significantly higher mean health-related quality of life in well-recovered men.

Table of Contents

Abbreviations/notations **1**

Example **1**

Sociodemographic factors **3**

Sex **3**
6 months after TBI (data as available) 5
12 months after TBI (data as available) 7
3 months after TBI (completers) 8
6 months after TBI (completers) 11
12 months after TBI (completers) 13

Age **15**
6 months after TBI (data as available) 17
12 months after TBI (data as available) 19
3 months after TBI (completers) 21
6 months after TBI (completers) 23
12 months after TBI (completers) 25

ISS **27**
6 months after TBI (data as available) 29
12 months after TBI (data as available) 30
3 months after TBI (completers) 32
6 months after TBI (completers) 33
12 months after TBI (completers) 35

Education **37**
6 months after TBI (data as available) 39
12 months after TBI (data as available) 41
3 months after TBI (completers) 43
6 months after TBI (completers) 45
12 months after TBI (completers) 46

Clinical care pathways **48**
6 months after TBI (data as available) 50
12 months after TBI (data as available) 52
3 months after TBI (completers) 54
6 months after TBI (completers) 56
12 months after TBI (completers) 58

Premorbid psychiatric disturbances **60**
6 months after TBI (data as available) 62
12 months after TBI (data as available) 64
3 months after TBI (completers) 66
6 months after TBI (completers) 68
12 months after TBI (completers) 69

TBI severity **71**
3 months after TBI (data as available) 71
6 months after TBI (data as available) 75
12 months after TBI (data as available) 78
3 months after TBI (completers) 80
6 months after TBI (completers) 82
12 months after TBI (completers) 83

Sociodemographic factors

Sex

3 months after TBI (data as available)

GOSE/-Q 7-8 vs. GOSE/-Q 5-6 – Male

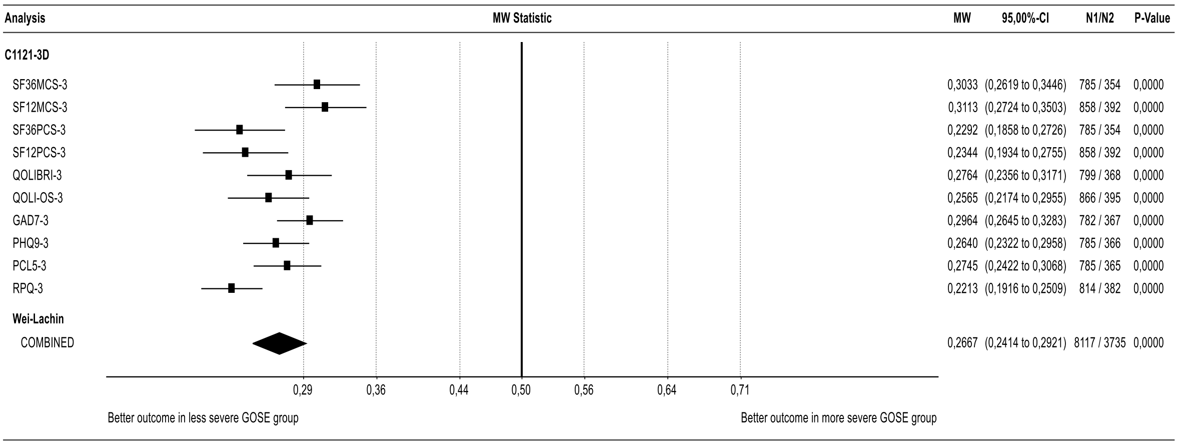

Analysis	MW	95,00%-CI	N1/N2	P-Value
**C1121-3D**				
SF36MCS-3	0,3033	(0,2619 to 0,3446)	785 / 354	0,0000
SF12MCS-3	0,3113	(0,2724 to 0,3503)	858 / 392	0,0000
SF36PCS-3	0,2292	(0,1858 to 0,2726)	785 / 354	0,0000
SF12PCS-3	0,2344	(0,1934 to 0,2755)	858 / 392	0,0000
QOLIBRI-3	0,2764	(0,2356 to 0,3171)	799 / 368	0,0000
QOLI-OS-3	0,2565	(0,2174 to 0,2955)	866 / 395	0,0000
GAD7-3	0,2964	(0,2645 to 0,3283)	782 / 367	0,0000
PHQ9-3	0,2640	(0,2322 to 0,2958)	785 / 366	0,0000
PCL5-3	0,2745	(0,2422 to 0,3068)	785 / 365	0,0000
RPQ-3	0,2213	(0,1916 to 0,2509)	814 / 382	0,0000
**Wei-Lachin**				
COMBINED	0,2667	(0,2414 to 0,2921)	8117 / 3735	0,0000

GOSE/-Q 5-6 vs. GOSE/-Q 3-4 - Male

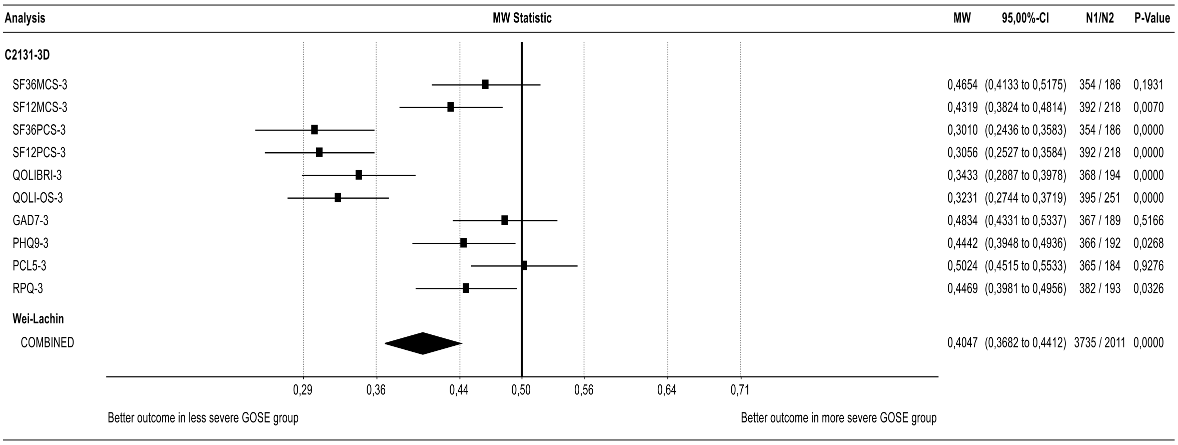

Analysis	MW	95,00%-CI	N1/N2	P-Value
**C2131-3D**				
SF36MCS-3	0,4654	(0,4133 to 0,5175)	354 / 186	0,1931
SF12MCS-3	0,4319	(0,3824 to 0,4814)	392 / 218	0,0070
SF36PCS-3	0,3010	(0,2436 to 0,3583)	354 / 186	0,0000
SF12PCS-3	0,3056	(0,2527 to 0,3584)	392 / 218	0,0000
QOLIBRI-3	0,3433	(0,2887 to 0,3978)	368 / 194	0,0000
QOLI-OS-3	0,3231	(0,2744 to 0,3719)	395 / 251	0,0000
GAD7-3	0,4834	(0,4331 to 0,5337)	367 / 189	0,5166
PHQ9-3	0,4442	(0,3948 to 0,4936)	366 / 192	0,0268
PCL5-3	0,5024	(0,4515 to 0,5533)	365 / 184	0,9276
RPQ-3	0,4469	(0,3981 to 0,4956)	382 / 193	0,0326
**Wei-Lachin**				
COMBINED	0,4047	(0,3682 to 0,4412)	3735 / 2011	0,0000

GOSE/-Q 7-8 vs. GOSE/-Q 3-4 - Male

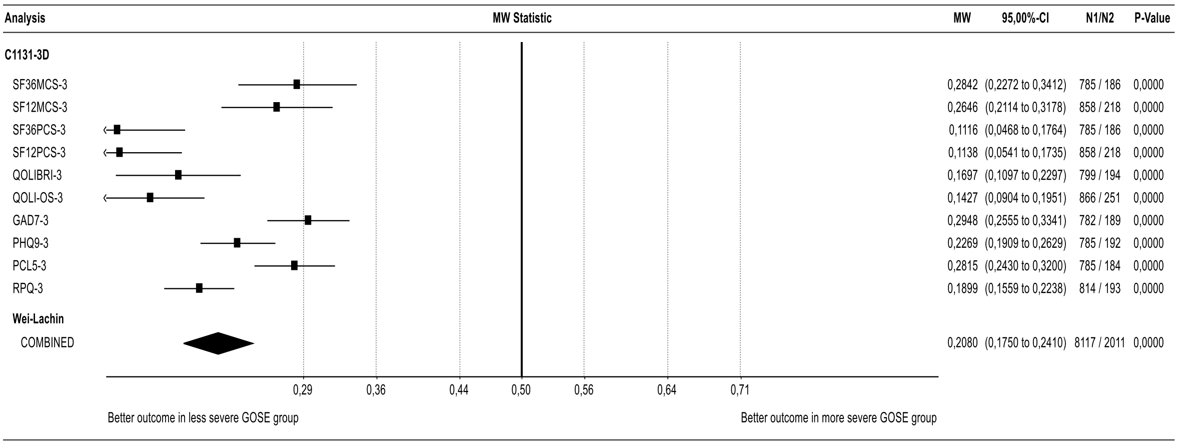

Analysis	MW	95,00%-CI	N1/N2	P-Value
**C1131-3D**				
SF36MCS-3	0,2842	(0,2272 to 0,3412)	785 / 186	0,0000
SF12MCS-3	0,2646	(0,2114 to 0,3178)	858 / 218	0,0000
SF36PCS-3	0,1116	(0,0468 to 0,1764)	785 / 186	0,0000
SF12PCS-3	0,1138	(0,0541 to 0,1735)	858 / 218	0,0000
QOLIBRI-3	0,1697	(0,1097 to 0,2297)	799 / 194	0,0000
QOLI-OS-3	0,1427	(0,0904 to 0,1951)	866 / 251	0,0000
GAD7-3	0,2948	(0,2555 to 0,3341)	782 / 189	0,0000
PHQ9-3	0,2269	(0,1909 to 0,2629)	785 / 192	0,0000
PCL5-3	0,2815	(0,2430 to 0,3200)	785 / 184	0,0000
RPQ-3	0,1899	(0,1559 to 0,2238)	814 / 193	0,0000
**Wei-Lachin**				
COMBINED	0,2080	(0,1750 to 0,2410)	8117 / 2011	0,0000

GOSE/-Q 7-8 vs. GOSE/-Q 5-6- Female

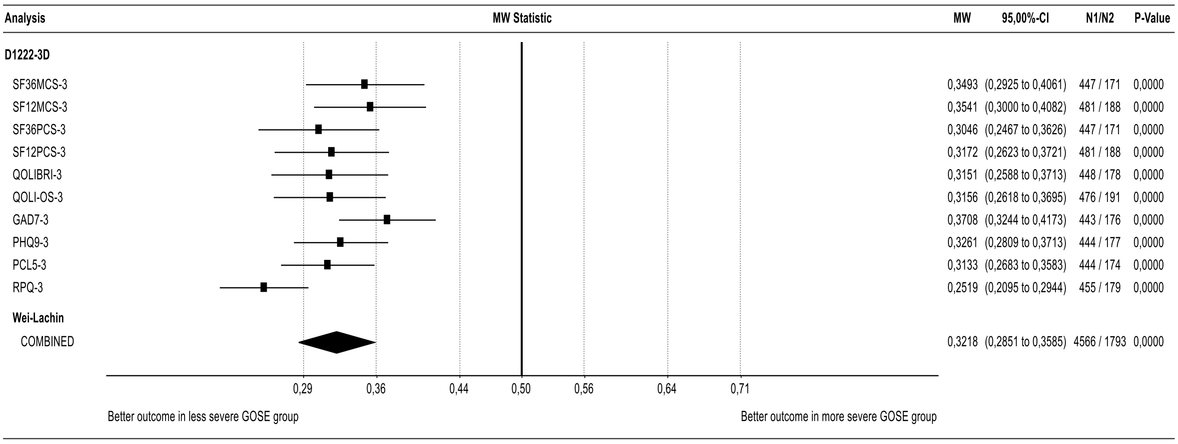

Analysis	MW	95,00%-CI	N1/N2	P-Value
**D1222-3D**				
SF36MCS-3	0,3493	(0,2925 to 0,4061)	447 / 171	0,0000
SF12MCS-3	0,3541	(0,3000 to 0,4082)	481 / 188	0,0000
SF36PCS-3	0,3046	(0,2467 to 0,3626)	447 / 171	0,0000
SF12PCS-3	0,3172	(0,2623 to 0,3721)	481 / 188	0,0000
QOLIBRI-3	0,3151	(0,2588 to 0,3713)	448 / 178	0,0000
QOLI-OS-3	0,3156	(0,2618 to 0,3695)	476 / 191	0,0000
GAD7-3	0,3708	(0,3244 to 0,4173)	443 / 176	0,0000
PHQ9-3	0,3261	(0,2809 to 0,3713)	444 / 177	0,0000
PCL5-3	0,3133	(0,2683 to 0,3583)	444 / 174	0,0000
RPQ-3	0,2519	(0,2095 to 0,2944)	455 / 179	0,0000
**Wei-Lachin**				
COMBINED	0,3218	(0,2851 to 0,3585)	4566 / 1793	0,0000

GOSE/-Q 5-6 vs. GOSE/-Q 3-4 - Female

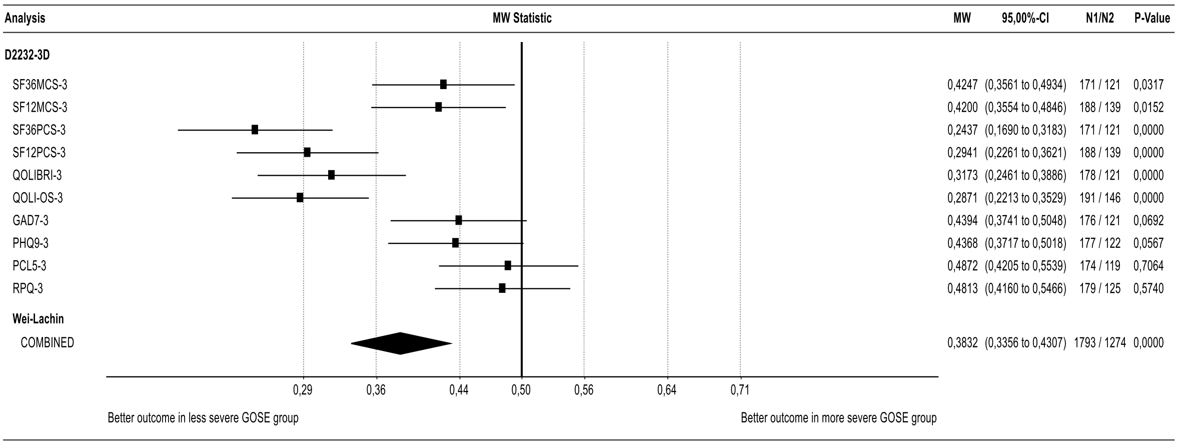

Analysis	MW	95,00%-CI	N1/N2	P-Value
**D2232-3D**				
SF36MCS-3	0,4247	(0,3561 to 0,4934)	171 / 121	0,0317
SF12MCS-3	0,4200	(0,3554 to 0,4846)	188 / 139	0,0152
SF36PCS-3	0,2437	(0,1690 to 0,3183)	171 / 121	0,0000
SF12PCS-3	0,2941	(0,2261 to 0,3621)	188 / 139	0,0000
QOLIBRI-3	0,3173	(0,2461 to 0,3886)	178 / 121	0,0000
QOLI-OS-3	0,2871	(0,2213 to 0,3529)	191 / 146	0,0000
GAD7-3	0,4394	(0,3741 to 0,5048)	176 / 121	0,0692
PHQ9-3	0,4368	(0,3717 to 0,5018)	177 / 122	0,0567
PCL5-3	0,4872	(0,4205 to 0,5539)	174 / 119	0,7064
RPQ-3	0,4813	(0,4160 to 0,5466)	179 / 125	0,5740
**Wei-Lachin**				
COMBINED	0,3832	(0,3356 to 0,4307)	1793 / 1274	0,0000

GOSE/-Q 7-8 vs. GOSE/-Q 3-4 - Female

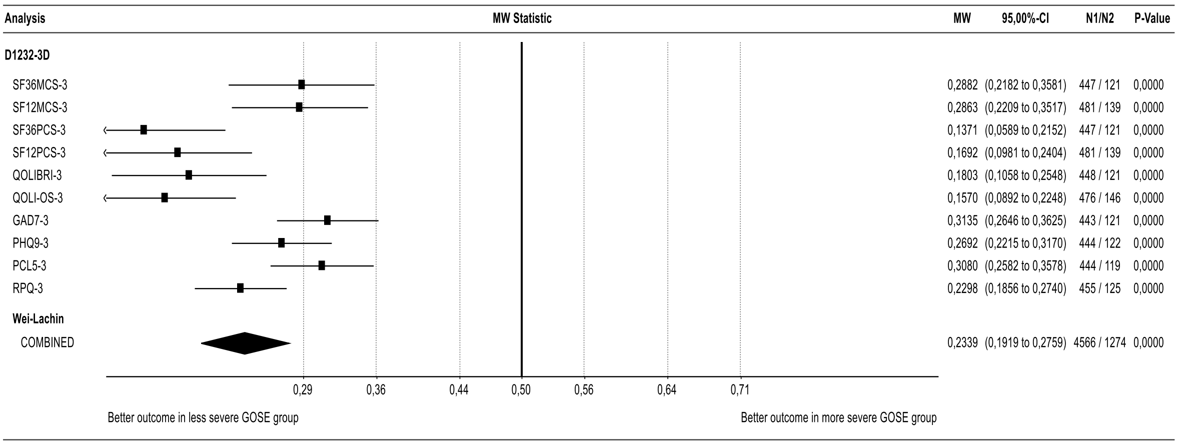

Analysis	MW	95,00%-CI	N1/N2	P-Value
**D1232-3D**				
SF36MCS-3	0,2882	(0,2182 to 0,3581)	447 / 121	0,0000
SF12MCS-3	0,2863	(0,2209 to 0,3517)	481 / 139	0,0000
SF36PCS-3	0,1371	(0,0589 to 0,2152)	447 / 121	0,0000
SF12PCS-3	0,1692	(0,0981 to 0,2404)	481 / 139	0,0000
QOLIBRI-3	0,1803	(0,1058 to 0,2548)	448 / 121	0,0000
QOLI-OS-3	0,1570	(0,0892 to 0,2248)	476 / 146	0,0000
GAD7-3	0,3135	(0,2646 to 0,3625)	443 / 121	0,0000
PHQ9-3	0,2692	(0,2215 to 0,3170)	444 / 122	0,0000
PCL5-3	0,3080	(0,2582 to 0,3578)	444 / 119	0,0000
RPQ-3	0,2298	(0,1856 to 0,2740)	455 / 125	0,0000
**Wei-Lachin**				
COMBINED	0,2339	(0,1919 to 0,2759)	4566 / 1274	0,0000

6 months after TBI (data as available)

GOSE/-Q 7-8 vs. GOSE/-Q 5-6 – Male

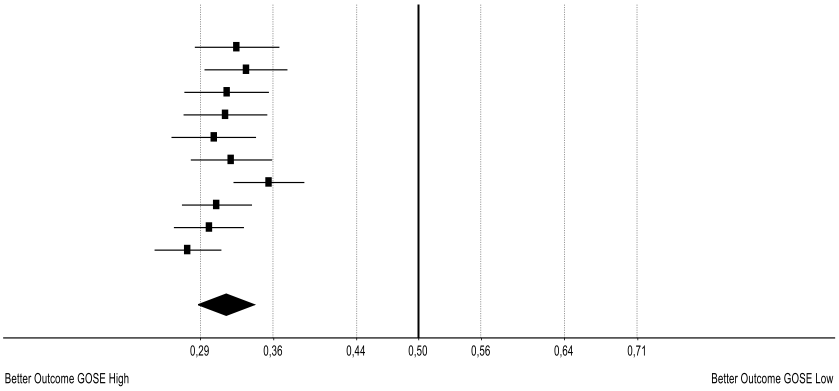

Analysis	MW	95,00%-CI	N1/N2	P-Value
**C1121-6D**				
SF36MCS-6	0,3252	(0,2846 to 0,3659)	726 / 357	0,0000
SF12MCS-6	0,3341	(0,2942 to 0,3740)	767 / 363	0,0000
SF36PCS-6	0,3154	(0,2744 to 0,3563)	726 / 357	0,0000
SF12PCS-6	0,3142	(0,2737 to 0,3547)	767 / 363	0,0000
QOLIBRI-6	0,3030	(0,2620 to 0,3439)	743 / 352	0,0000
QOLI-OS-6	0,3199	(0,2809 to 0,3588)	773 / 369	0,0000
GAD7-6	0,3560	(0,3221 to 0,3899)	729 / 356	0,0000
PHQ9-6	0,3058	(0,2722 to 0,3394)	728 / 356	0,0000
PCL5-6	0,2984	(0,2648 to 0,3320)	735 / 353	0,0000
RPQ-6	0,2781	(0,2458 to 0,3103)	756 / 365	0,0000
**Wei-Lachin**				
COMBINED	0,3150	(0,2876 to 0,3424)	7450 / 3591	0,0000

GOSE/-Q 5-6 vs. GOSE/-Q 3-4 – Male

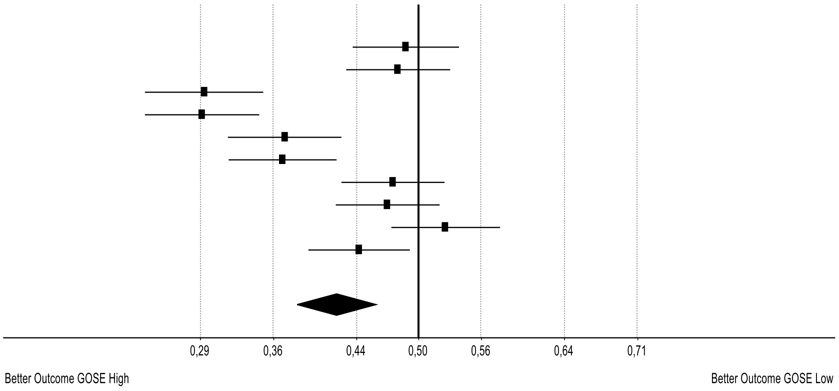

Analysis	MW	95,00%-CI	N1/N2	P-Value
**C2131-6D**				
SF36MCS-6	0,4879	(0,4367 to 0,5391)	357 / 191	0,6429
SF12MCS-6	0,4802	(0,4302 to 0,5303)	363 / 204	0,4389
SF36PCS-6	0,2939	(0,2369 to 0,3509)	357 / 191	0,0000
SF12PCS-6	0,2917	(0,2365 to 0,3469)	363 / 204	0,0000
QOLIBRI-6	0,3711	(0,3168 to 0,4255)	352 / 190	0,0000
QOLI-OS-6	0,3693	(0,3174 to 0,4213)	369 / 210	0,0000
GAD7-6	0,4751	(0,4255 to 0,5248)	356 / 189	0,3257
PHQ9-6	0,4701	(0,4203 to 0,5199)	356 / 189	0,2386
PCL5-6	0,5259	(0,4739 to 0,5779)	353 / 181	0,3288
RPQ-6	0,4425	(0,3936 to 0,4913)	365 / 192	0,0210
**Wei-Lachin**				
COMBINED	0,4208	(0,3828 to 0,4588)	3591 / 1941	0,0000

GOSE/-Q 7-8 vs. GOSE/-Q 3-4 - Male

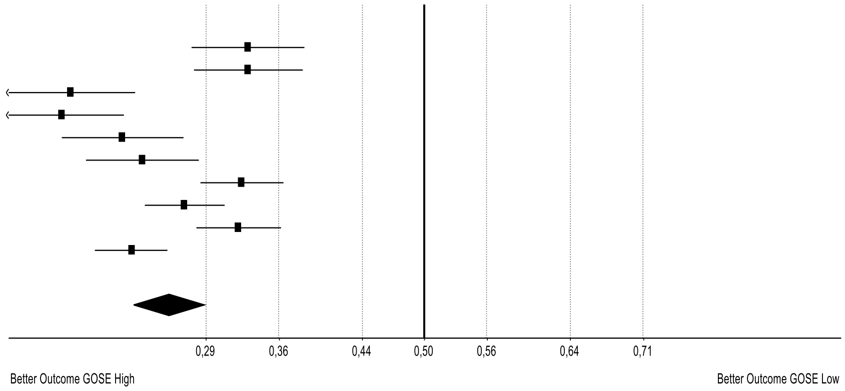

Analysis	MW	95,00%-CI	N1/N2	P-Value
**C1131-6D**				
SF36MCS-6	0,3304	(0,2762 to 0,3845)	726 / 191	0,0000
SF12MCS-6	0,3306	(0,2784 to 0,3827)	767 / 204	0,0000
SF36PCS-6	0,1600	(0,0983 to 0,2217)	726 / 191	0,0000
SF12PCS-6	0,1513	(0,0913 to 0,2114)	767 / 204	0,0000
QOLIBRI-6	0,2099	(0,1512 to 0,2687)	743 / 190	0,0000
QOLI-OS-6	0,2290	(0,1749 to 0,2831)	773 / 210	0,0000
GAD7-6	0,3244	(0,2845 to 0,3643)	729 / 189	0,0000
PHQ9-6	0,2696	(0,2315 to 0,3076)	728 / 189	0,0000
PCL5-6	0,3214	(0,2807 to 0,3622)	735 / 181	0,0000
RPQ-6	0,2185	(0,1836 to 0,2533)	756 / 192	0,0000
**Wei-Lachin**				
COMBINED	0,2545	(0,2202 to 0,2888)	7450 / 1941	0,0000

GOSE/-Q 7-8 vs. GOSE/-Q 5-6- Female

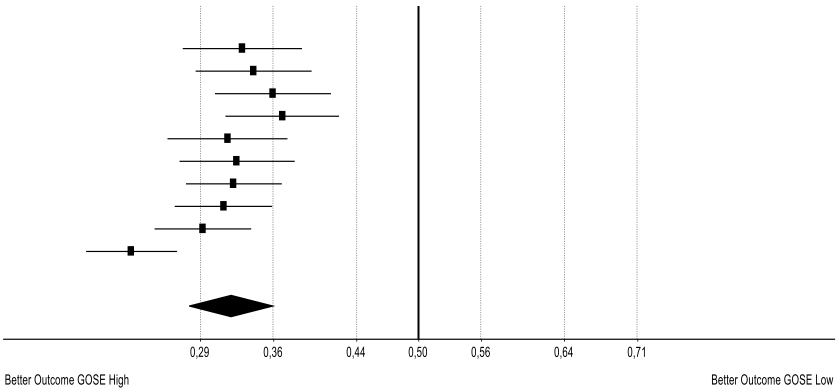

Analysis	MW	95,00%-CI	N1/N2	P-Value
**D1222-6D**				
SF36MCS-6	0,3305	(0,2733 to 0,3876)	381 / 175	0,0000
SF12MCS-6	0,3415	(0,2857 to 0,3973)	394 / 181	0,0000
SF36PCS-6	0,3599	(0,3039 to 0,4159)	379 / 175	0,0000
SF12PCS-6	0,3689	(0,3142 to 0,4235)	394 / 181	0,0000
QOLIBRI-6	0,3163	(0,2587 to 0,3739)	379 / 174	0,0000
QOLI-OS-6	0,3253	(0,2697 to 0,3808)	393 / 182	0,0000
GAD7-6	0,3222	(0,2762 to 0,3682)	380 / 173	0,0000
PHQ9-6	0,3122	(0,2654 to 0,3591)	381 / 176	0,0000
PCL5-6	0,2925	(0,2459 to 0,3390)	384 / 174	0,0000
RPQ-6	0,2238	(0,1802 to 0,2674)	390 / 180	0,0000
**Wei-Lachin**				
COMBINED	0,3193	(0,2790 to 0,3596)	3855 / 1771	0,0000

GOSE/-Q 5-6 vs. GOSE/-Q 3-4 - Female

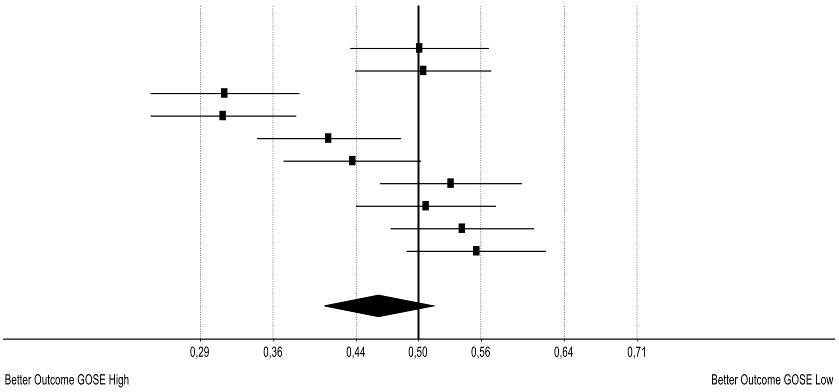

Analysis	MW	95,00%-CI	N1/N2	P-Value
**D2232-6D**				
SF36MCS-6	0,5008	(0,4341 to 0,5675)	175 / 121	0,9817
SF12MCS-6	0,5044	(0,4392 to 0,5696)	181 / 128	0,8937
SF36PCS-6	0,3136	(0,2418 to 0,3854)	175 / 121	0,0000
SF12PCS-6	0,3120	(0,2421 to 0,3819)	181 / 128	0,0000
QOLIBRI-6	0,4135	(0,3442 to 0,4829)	174 / 117	0,0145
QOLI-OS-6	0,4363	(0,3699 to 0,5027)	182 / 127	0,0601
GAD7-6	0,5309	(0,4625 to 0,5992)	173 / 114	0,3759
PHQ9-6	0,5071	(0,4396 to 0,5746)	176 / 115	0,8375
PCL5-6	0,5419	(0,4732 to 0,6105)	174 / 114	0,2321
RPQ-6	0,5554	(0,4884 to 0,6224)	180 / 121	0,1053
**Wei-Lachin**				
COMBINED	0,4616	(0,4094 to 0,5138)	1771 / 1206	0,1492

GOSE/-Q 7-8 vs. GOSE/-Q 3-4 - Female

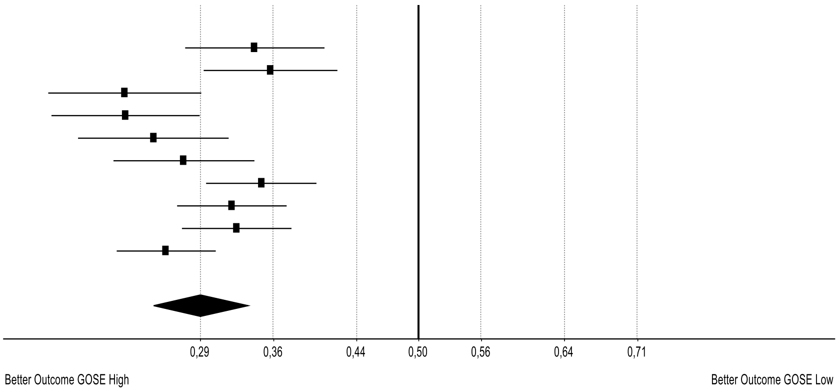

Analysis	MW	95,00%-CI	N1/N2	P-Value
**D1232-6D**				
SF36MCS-6	0,3421	(0,2751 to 0,4091)	381 / 121	0,0000
SF12MCS-6	0,3575	(0,2930 to 0,4220)	394 / 128	0,0000
SF36PCS-6	0,2174	(0,1439 to 0,2910)	379 / 121	0,0000
SF12PCS-6	0,2181	(0,1468 to 0,2893)	394 / 128	0,0000
QOLIBRI-6	0,2449	(0,1724 to 0,3174)	379 / 117	0,0000
QOLI-OS-6	0,2742	(0,2067 to 0,3418)	393 / 127	0,0000
GAD7-6	0,3488	(0,2957 to 0,4019)	380 / 114	0,0000
PHQ9-6	0,3200	(0,2673 to 0,3728)	381 / 115	0,0000
PCL5-6	0,3250	(0,2724 to 0,3776)	384 / 114	0,0000
RPQ-6	0,2572	(0,2097 to 0,3048)	390 / 121	0,0000
**Wei-Lachin**				
COMBINED	0,2905	(0,2450 to 0,3361)	3855 / 1206	0,0000

12 months after TBI (data as available)

GOSE/-Q 7-8 vs. GOSE/-Q 5-6 -Male

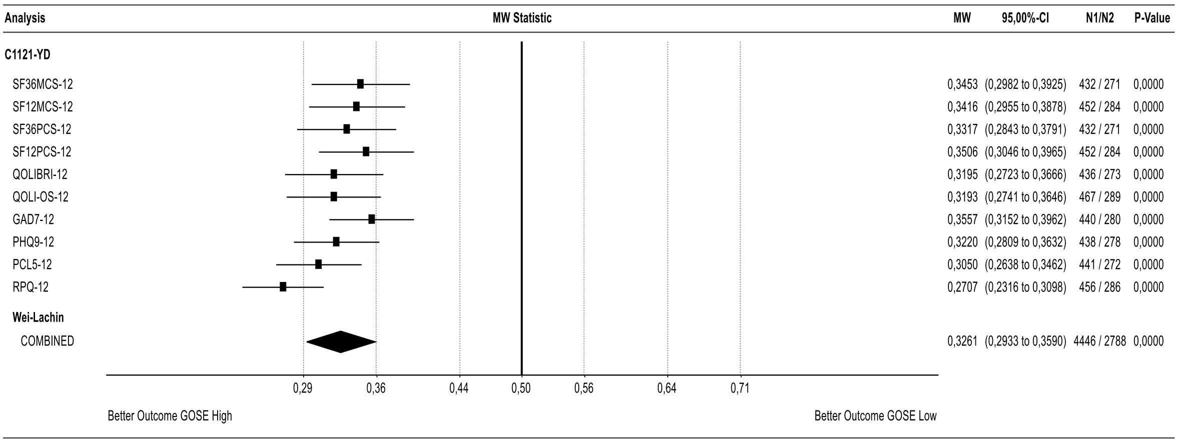

GOSE/-Q 5-6 vs. GOSE/-Q 3-4 - Male

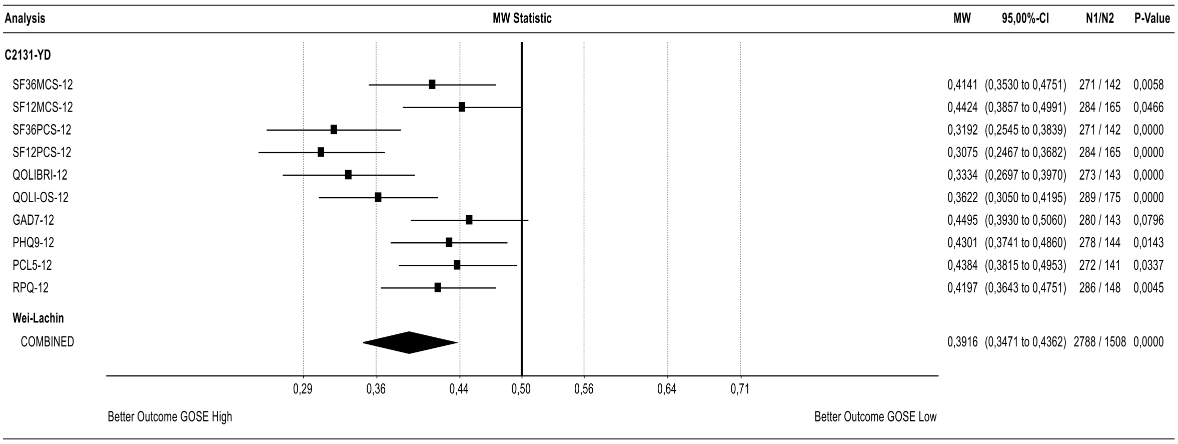

GOSE/-Q 7-8 vs. GOSE/-Q 3-4 - Male

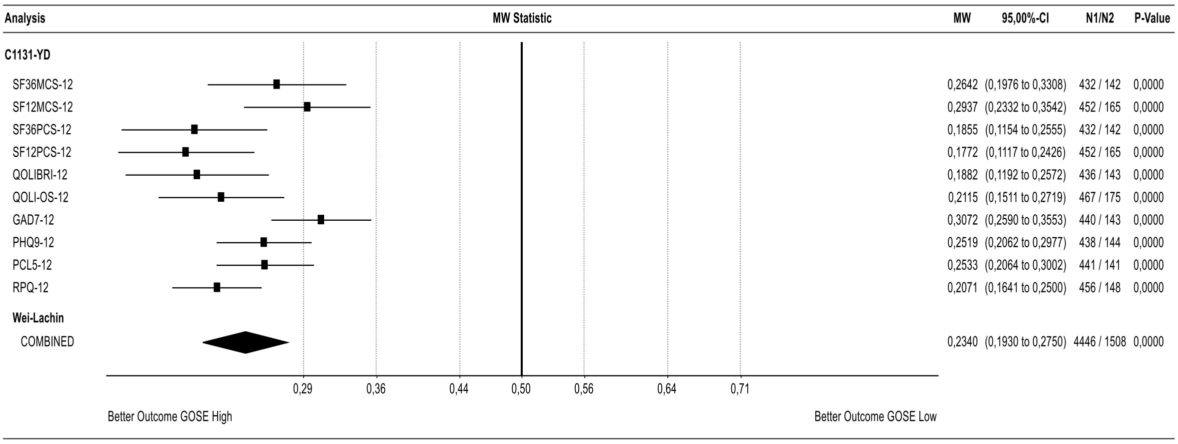

GOSE/-Q 7-8 vs. GOSE/-Q 5-6- Female

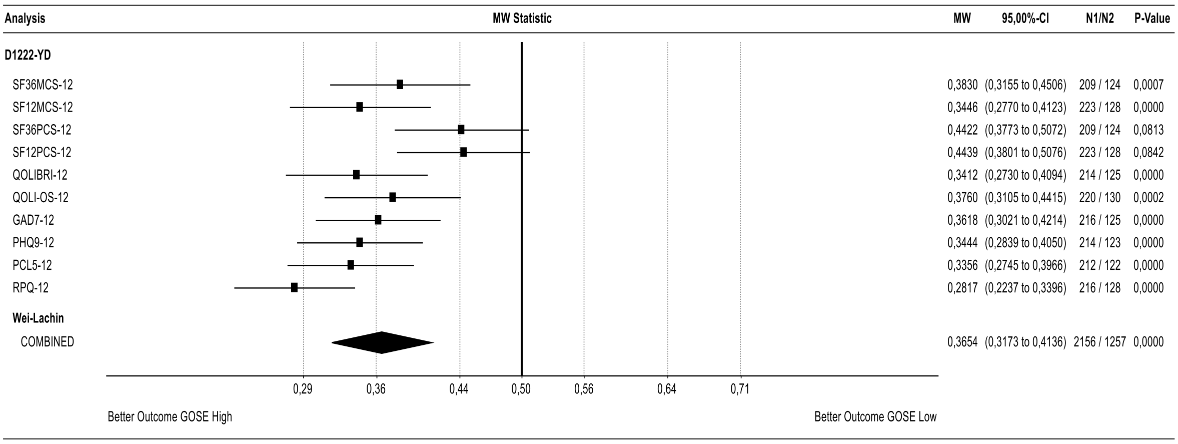

GOSE/-Q 5-6 vs. GOSE/-Q 3-4 - Female

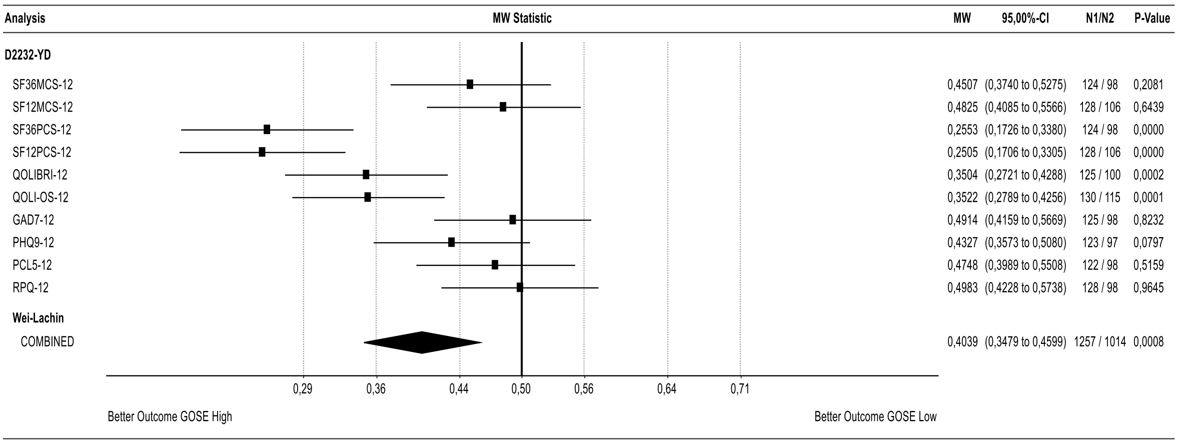

GOSE/-Q 7-8 vs. GOSE/-Q 3-4 - Female

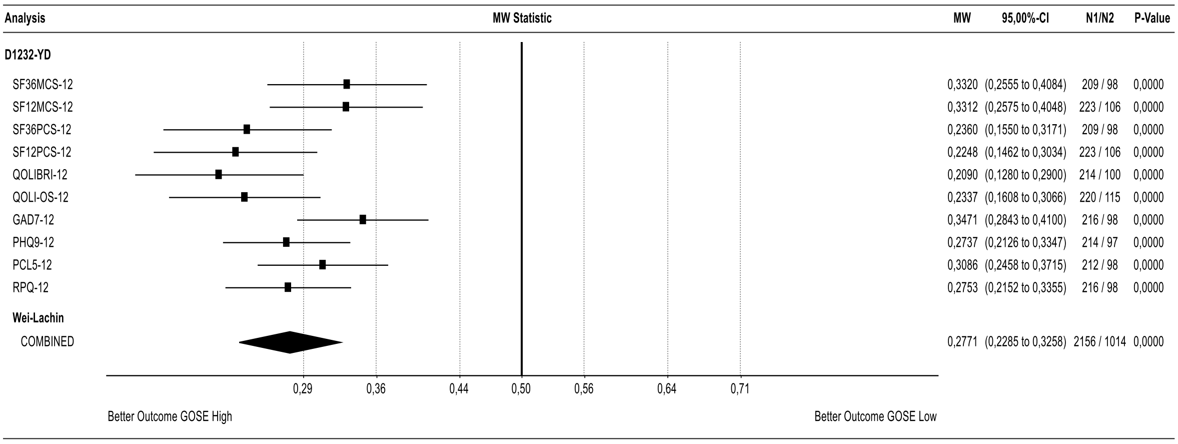

3 months after TBI (completers)

GOSE/-Q 7-8 vs. GOSE/-Q 5-6 – Male

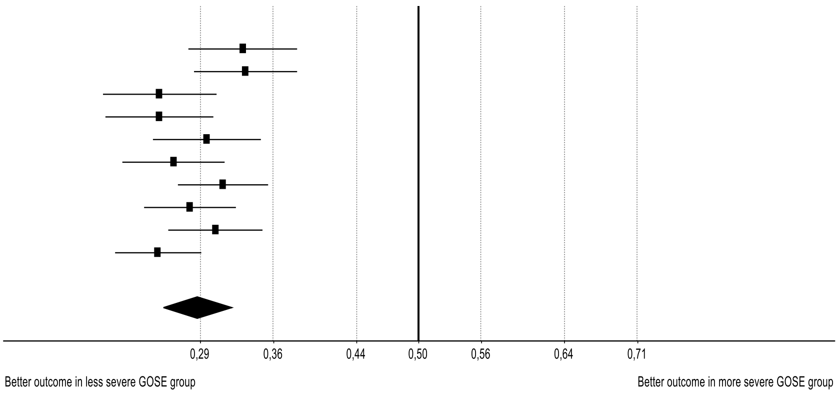

Analysis	MW	95,00%-CI	N1/N2	P-Value
**C1121-3X**				
SF36MCS-X	0,3308	(0,2784 to 0,3831)	359 / 222	0,0000
SF12MCS-X	0,3336	(0,2840 to 0,3832)	397 / 246	0,0000
SF36PCS-X	0,2510	(0,1965 to 0,3055)	359 / 222	0,0000
SF12PCS-X	0,2504	(0,1986 to 0,3023)	397 / 246	0,0000
QOLIBRI-X	0,2964	(0,2445 to 0,3484)	370 / 229	0,0000
QOLI-OS-X	0,2643	(0,2152 to 0,3135)	413 / 254	0,0000
GAD7-X3	0,3120	(0,2686 to 0,3554)	367 / 237	0,0000
PHQ9-X3	0,2800	(0,2358 to 0,3242)	365 / 236	0,0000
PCL5-X3	0,3046	(0,2595 to 0,3497)	372 / 230	0,0000
RPQ-X3	0,2494	(0,2077 to 0,2911)	392 / 247	0,0000
**Wei-Lachin**				
COMBINED	0,2873	(0,2544 to 0,3201)	3791 / 2369	0,0000

GOSE/-Q 5-6 vs. GOSE/-Q 3-4 - Male

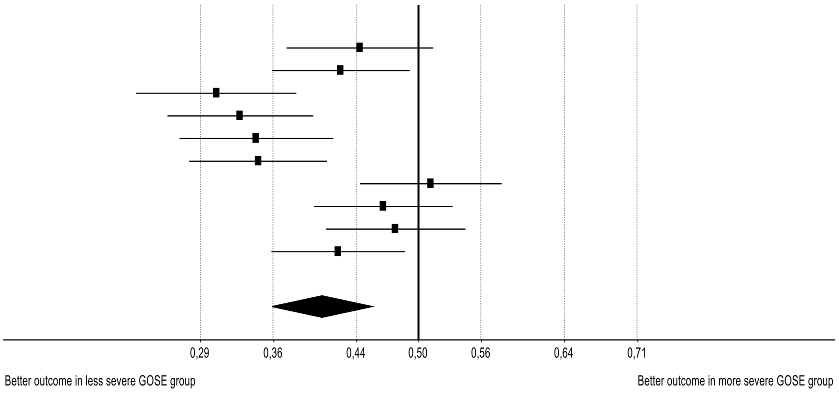

Analysis	MW	95,00%-CI	N1/N2	P-Value
**C2131-3X**				
SF36MCS-X	0,4437	(0,3731 to 0,5142)	222 / 101	0,1176
SF12MCS-X	0,4252	(0,3588 to 0,4915)	246 / 117	0,0271
SF36PCS-X	0,3053	(0,2284 to 0,3823)	222 / 101	0,0000
SF12PCS-X	0,3281	(0,2580 to 0,3983)	246 / 117	0,0000
QOLIBRI-X	0,3437	(0,2698 to 0,4176)	229 / 101	0,0000
QOLI-OS-X	0,3456	(0,2796 to 0,4117)	254 / 127	0,0000
GAD7-X3	0,5118	(0,4438 to 0,5798)	237 / 98	0,7333
PHQ9-X3	0,4662	(0,3997 to 0,5326)	236 / 100	0,3182
PCL5-X3	0,4779	(0,4111 to 0,5447)	230 / 97	0,5166
RPQ-X3	0,4227	(0,3586 to 0,4868)	247 / 101	0,0180
**Wei-Lachin**				
COMBINED	0,4070	(0,3590 to 0,4551)	2369 / 1060	0,0001

GOSE/-Q 7-8 vs. GOSE/-Q 3-4 - Male

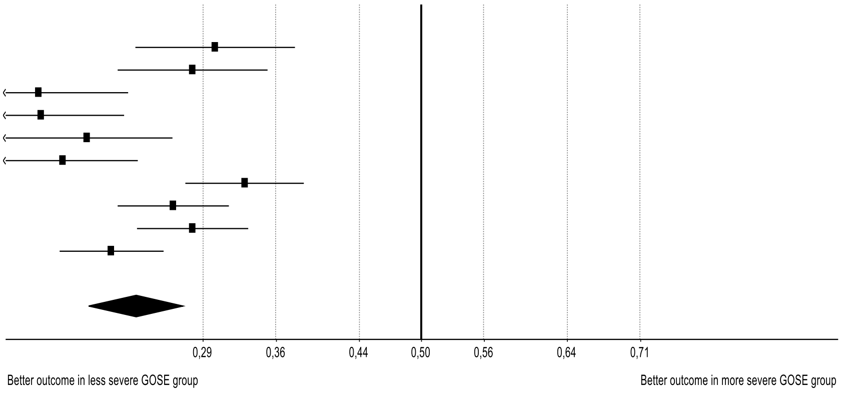

Analysis	MW	95,00%-CI	N1/N2	P-Value
**C1131-3X**				
SF36MCS-X	0,3017	(0,2248 to 0,3787)	359 / 101	0,0000
SF12MCS-X	0,2801	(0,2081 to 0,3521)	397 / 117	0,0000
SF36PCS-X	0,1321	(0,0459 to 0,2183)	359 / 101	0,0000
SF12PCS-X	0,1343	(0,0544 to 0,2142)	397 / 117	0,0000
QOLIBRI-X	0,1786	(0,0966 to 0,2605)	370 / 101	0,0000
QOLI-OS-X	0,1551	(0,0832 to 0,2271)	413 / 127	0,0000
GAD7-X3	0,3301	(0,2733 to 0,3868)	367 / 98	0,0000
PHQ9-X3	0,2614	(0,2079 to 0,3149)	365 / 100	0,0000
PCL5-X3	0,2800	(0,2265 to 0,3335)	372 / 97	0,0000
RPQ-X3	0,2022	(0,1525 to 0,2519)	392 / 101	0,0000
**Wei-Lachin**				
COMBINED	0,2256	(0,1800 to 0,2711)	3791 / 1060	0,0000

GOSE/-Q 7-8 vs. GOSE/-Q 5-6- Female

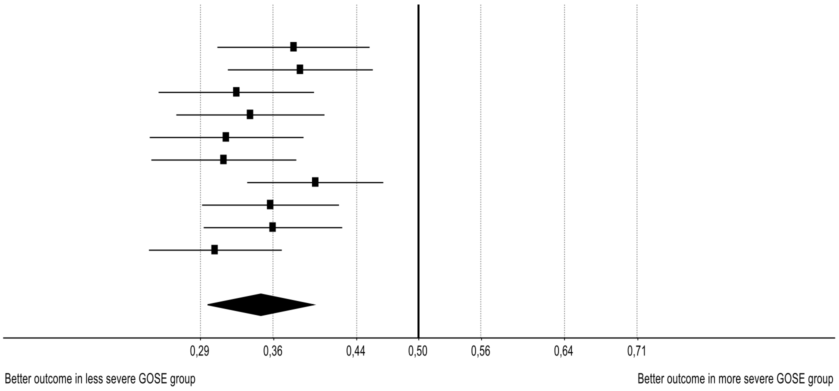

Analysis	MW	95,00%-CI	N1/N2	P-Value
**D1222-3X**				
SF36MCS-X	0,3796	(0,3065 to 0,4527)	171 / 107	0,0012
SF12MCS-X	0,3860	(0,3161 to 0,4560)	187 / 116	0,0014
SF36PCS-X	0,3248	(0,2500 to 0,3997)	171 / 107	0,0000
SF12PCS-X	0,3382	(0,2669 to 0,4095)	187 / 116	0,0000
QOLIBRI-X	0,3152	(0,2410 to 0,3893)	175 / 109	0,0000
QOLI-OS-X	0,3128	(0,2431 to 0,3825)	188 / 120	0,0000
GAD7-X3	0,4007	(0,3350 to 0,4663)	175 / 109	0,0030
PHQ9-X3	0,3577	(0,2920 to 0,4235)	174 / 111	0,0000
PCL5-X3	0,3599	(0,2932 to 0,4267)	172 / 107	0,0000
RPQ-X3	0,3043	(0,2405 to 0,3681)	176 / 115	0,0000
**Wei-Lachin**				
COMBINED	0,3479	(0,2974 to 0,3985)	1776 / 1117	0,0000

GOSE/-Q 5-6 vs. GOSE/-Q 3-4 - Female

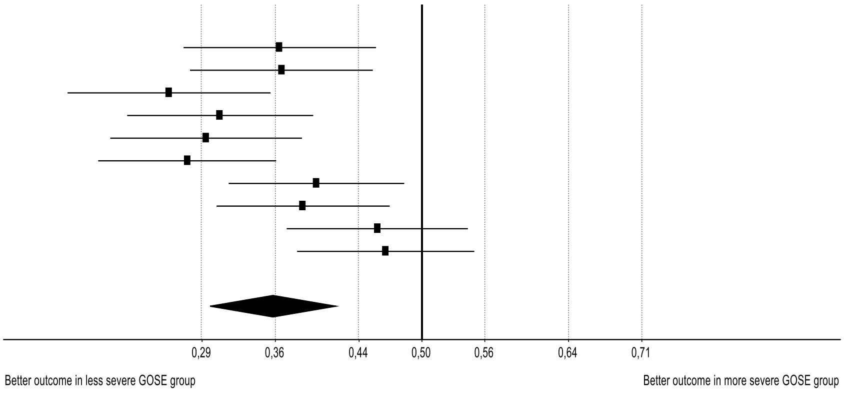

Analysis	MW	95,00%-CI	N1/N2	P-Value
**D2232-3X**				
SF36MCS-X	0,3644	(0,2725 to 0,4562)	107 / 69	0,0038
SF12MCS-X	0,3662	(0,2792 to 0,4531)	116 / 78	0,0025
SF36PCS-X	0,2590	(0,1623 to 0,3557)	107 / 69	0,0000
SF12PCS-X	0,3075	(0,2186 to 0,3964)	116 / 78	0,0000
QOLIBRI-X	0,2942	(0,2025 to 0,3859)	109 / 74	0,0000
QOLI-OS-X	0,2764	(0,1912 to 0,3615)	120 / 86	0,0000
GAD7-X3	0,3995	(0,3157 to 0,4832)	109 / 67	0,0187
PHQ9-X3	0,3869	(0,3039 to 0,4699)	111 / 68	0,0076
PCL5-X3	0,4577	(0,3714 to 0,5441)	107 / 67	0,3374
RPQ-X3	0,4657	(0,3809 to 0,5506)	115 / 68	0,4285
**Wei-Lachin**				
COMBINED	0,3577	(0,2977 to 0,4178)	1117 / 724	0,0000

GOSE/-Q 7-8 vs. GOSE/-Q 3-4 - Female

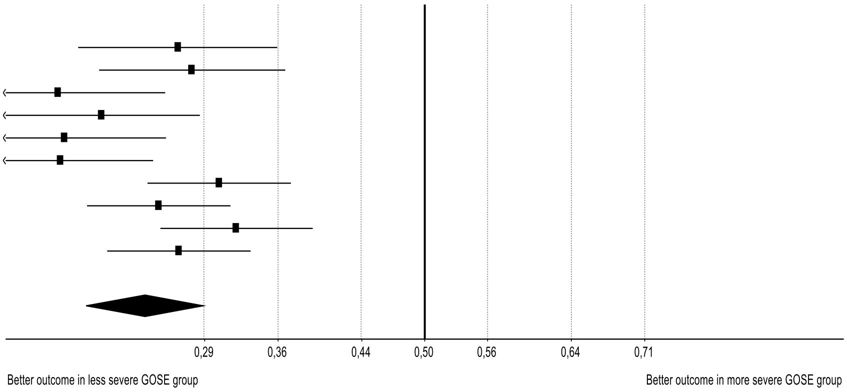

Analysis	MW	95,00%-CI	N1/N2	P-Value
**D1232-3X**				
SF36MCS-X	0,2648	(0,1699 to 0,3598)	171 / 69	0,0000
SF12MCS-X	0,2782	(0,1894 to 0,3671)	187 / 78	0,0000
SF36PCS-X	0,1504	(0,0481 to 0,2526)	171 / 69	0,0000
SF12PCS-X	0,1922	(0,0990 to 0,2855)	187 / 78	0,0000
QOLIBRI-X	0,1562	(0,0589 to 0,2535)	175 / 74	0,0000
QOLI-OS-X	0,1528	(0,0646 to 0,2410)	188 / 86	0,0000
GAD7-X3	0,3043	(0,2359 to 0,3727)	175 / 67	0,0000
PHQ9-X3	0,2463	(0,1778 to 0,3148)	174 / 68	0,0000
PCL5-X3	0,3207	(0,2478 to 0,3935)	172 / 67	0,0000
RPQ-X3	0,2655	(0,1972 to 0,3339)	176 / 68	0,0000
**Wei-Lachin**				
COMBINED	0,2331	(0,1773 to 0,2889)	1776 / 724	0,0000

6 months after TBI (completers)

GOSE/-Q 7-8 vs. GOSE/-Q 5-6 – Male

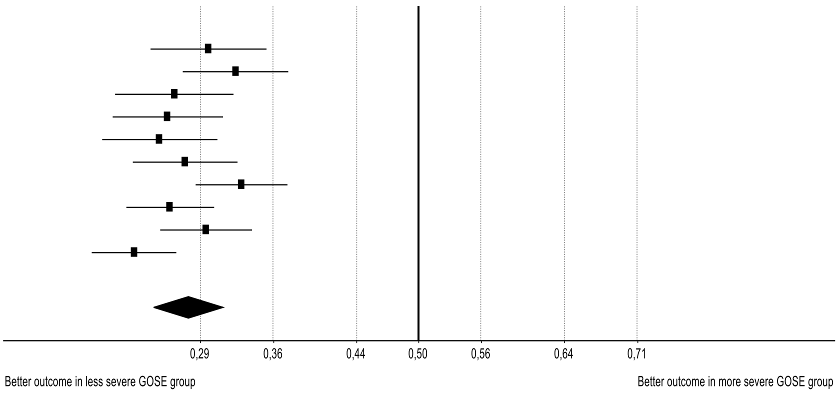

Analysis	MW	95,00%-CI	N1/N2	P-Value
**C1121-6X**				
SF36MCS-X	0,2979	(0,2422 to 0,3537)	423 / 195	0,0000
SF12MCS-X	0,3239	(0,2731 to 0,3748)	462 / 227	0,0000
SF36PCS-X	0,2653	(0,2084 to 0,3222)	423 / 195	0,0000
SF12PCS-X	0,2586	(0,2055 to 0,3117)	462 / 227	0,0000
QOLIBRI-X	0,2509	(0,1958 to 0,3061)	428 / 209	0,0000
QOLI-OS-X	0,2754	(0,2251 to 0,3257)	478 / 235	0,0000
GAD7-X6	0,3296	(0,2856 to 0,3736)	430 / 210	0,0000
PHQ9-X6	0,2609	(0,2186 to 0,3032)	427 / 212	0,0000
PCL5-X6	0,2956	(0,2516 to 0,3397)	429 / 209	0,0000
RPQ-X6	0,2266	(0,1859 to 0,2672)	455 / 221	0,0000
**Wei-Lachin**				
COMBINED	0,2785	(0,2454 to 0,3116)	4417 / 2140	0,0000

GOSE/-Q 5-6 vs. GOSE/-Q 3-4 – Male

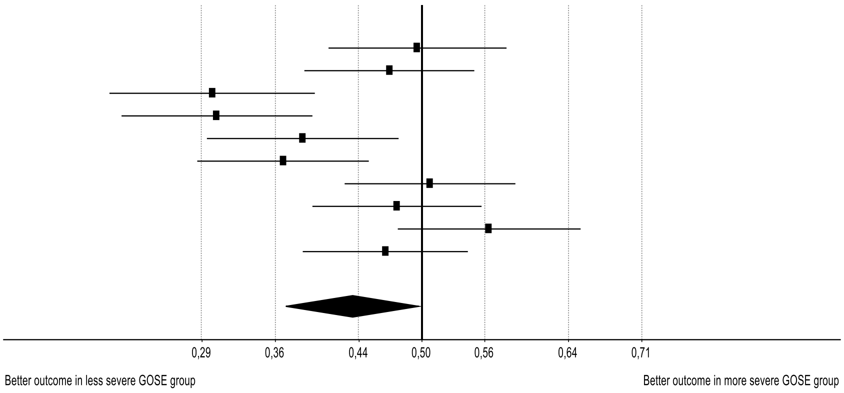

Analysis	MW	95,00%-CI	N1/N2	P-Value
**C2131-6X**				
SF36MCS-X	0,4960	(0,4109 to 0,5811)	195 / 60	0,9271
SF12MCS-X	0,4694	(0,3880 to 0,5507)	227 / 68	0,4601
SF36PCS-X	0,3000	(0,2019 to 0,3982)	195 / 60	0,0001
SF12PCS-X	0,3046	(0,2132 to 0,3960)	227 / 68	0,0000
QOLIBRI-X	0,3865	(0,2953 to 0,4777)	209 / 60	0,0147
QOLI-OS-X	0,3678	(0,2858 to 0,4497)	235 / 77	0,0016
GAD7-X6	0,5078	(0,4263 to 0,5894)	210 / 60	0,8509
PHQ9-X6	0,4763	(0,3957 to 0,5570)	212 / 61	0,5653
PCL5-X6	0,5646	(0,4775 to 0,6516)	209 / 59	0,1460
RPQ-X6	0,4656	(0,3868 to 0,5444)	221 / 62	0,3924
**Wei-Lachin**				
COMBINED	0,4339	(0,3704 to 0,4974)	2140 / 635	0,0412

GOSE/-Q 7-8 vs. GOSE/-Q 3-4 - Male

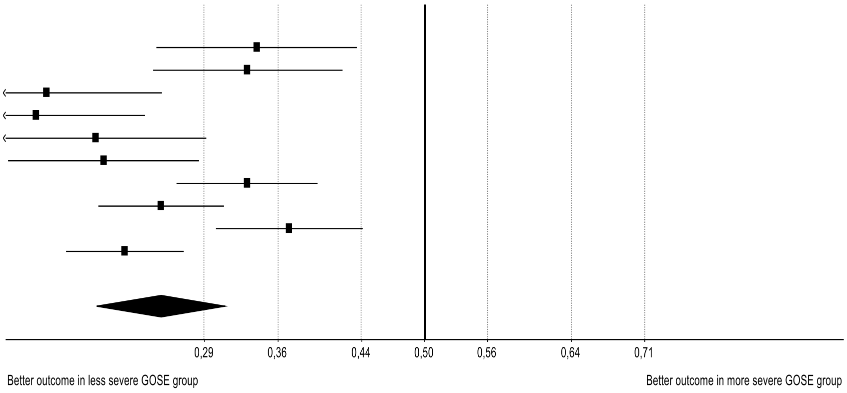

Analysis	MW	95,00%-CI	N1/N2	P-Value
**C1131-6X**				
SF36MCS-X	0,3402	(0,2445 to 0,4359)	423 / 60	0,0011
SF12MCS-X	0,3314	(0,2408 to 0,4219)	462 / 68	0,0003
SF36PCS-X	0,1397	(0,0298 to 0,2496)	423 / 60	0,0000
SF12PCS-X	0,1293	(0,0252 to 0,2333)	462 / 68	0,0000
QOLIBRI-X	0,1868	(0,0819 to 0,2917)	428 / 60	0,0000
QOLI-OS-X	0,1940	(0,1030 to 0,2850)	478 / 77	0,0000
GAD7-X6	0,3310	(0,2638 to 0,3982)	430 / 60	0,0000
PHQ9-X6	0,2489	(0,1892 to 0,3085)	427 / 61	0,0000
PCL5-X6	0,3710	(0,3012 to 0,4408)	429 / 59	0,0003
RPQ-X6	0,2142	(0,1579 to 0,2705)	455 / 62	0,0000
**Wei-Lachin**				
COMBINED	0,2486	(0,1874 to 0,3099)	4417 / 635	0,0000

GOSE/-Q 7-8 vs. GOSE/-Q 5-6- Female

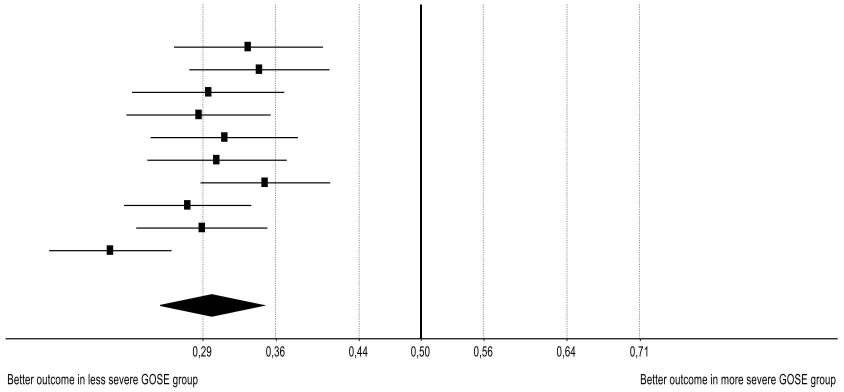

Analysis	MW	95,00%-CI	N1/N2	P-Value
**D1222-6X**				
SF36MCS-X	0,3337	(0,2621 to 0,4054)	190 / 116	0,0000
SF12MCS-X	0,3444	(0,2770 to 0,4117)	203 / 131	0,0000
SF36PCS-X	0,2952	(0,2222 to 0,3681)	190 / 116	0,0000
SF12PCS-X	0,2861	(0,2167 to 0,3555)	203 / 131	0,0000
QOLIBRI-X	0,3107	(0,2399 to 0,3815)	194 / 121	0,0000
QOLI-OS-X	0,3036	(0,2367 to 0,3705)	207 / 134	0,0000
GAD7-X6	0,3499	(0,2876 to 0,4122)	192 / 119	0,0000
PHQ9-X6	0,2753	(0,2141 to 0,3365)	194 / 119	0,0000
PCL5-X6	0,2890	(0,2257 to 0,3523)	191 / 115	0,0000
RPQ-X6	0,2008	(0,1419 to 0,2597)	197 / 121	0,0000
**Wei-Lachin**				
COMBINED	0,2989	(0,2493 to 0,3484)	1961 / 1223	0,0000

GOSE/-Q 5-6 vs. GOSE/-Q 3-4 - Female

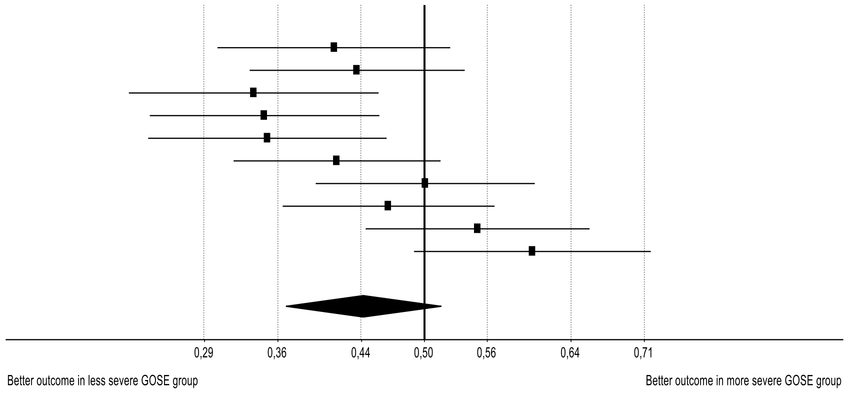

Analysis	MW	95,00%-CI	N1/N2	P-Value
**D2232-6X**				
SF36MCS-X	0,4139	(0,3028 to 0,5250)	116 / 38	0,1289
SF12MCS-X	0,4360	(0,3331 to 0,5388)	131 / 44	0,2225
SF36PCS-X	0,3371	(0,2181 to 0,4562)	116 / 38	0,0073
SF12PCS-X	0,3474	(0,2377 to 0,4571)	131 / 44	0,0064
QOLIBRI-X	0,3505	(0,2365 to 0,4645)	121 / 39	0,0102
QOLI-OS-X	0,4169	(0,3183 to 0,5156)	134 / 50	0,0988
GAD7-X6	0,5010	(0,3964 to 0,6057)	119 / 37	0,9847
PHQ9-X6	0,4659	(0,3647 to 0,5672)	119 / 38	0,5098
PCL5-X6	0,5508	(0,4439 to 0,6577)	115 / 38	0,3517
RPQ-X6	0,6034	(0,4903 to 0,7165)	121 / 38	0,0731
**Wei-Lachin**				
COMBINED	0,4423	(0,3683 to 0,5163)	1223 / 404	0,1262

GOSE/-Q 7-8 vs. GOSE/-Q 3-4 - Female

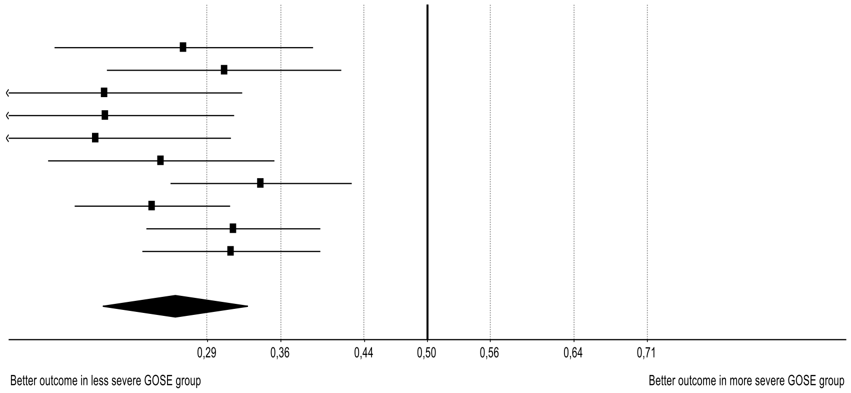

Analysis	MW	95,00%-CI	N1/N2	P-Value
**D1232-6X**				
SF36MCS-X	0,2675	(0,1442 to 0,3909)	190 / 38	0,0002
SF12MCS-X	0,3062	(0,1941 to 0,4183)	203 / 44	0,0007
SF36PCS-X	0,1916	(0,0597 to 0,3234)	190 / 38	0,0000
SF12PCS-X	0,1928	(0,0698 to 0,3158)	203 / 44	0,0000
QOLIBRI-X	0,1838	(0,0552 to 0,3125)	194 / 39	0,0000
QOLI-OS-X	0,2460	(0,1378 to 0,3542)	207 / 50	0,0000
GAD7-X6	0,3414	(0,2550 to 0,4279)	192 / 37	0,0003
PHQ9-X6	0,2375	(0,1631 to 0,3118)	194 / 38	0,0000
PCL5-X6	0,3153	(0,2323 to 0,3983)	191 / 38	0,0000
RPQ-X6	0,3129	(0,2279 to 0,3979)	197 / 38	0,0000
**Wei-Lachin**				
COMBINED	0,2595	(0,1900 to 0,3290)	1961 / 404	0,0000

12 months after TBI (completers)

GOSE/-Q 7-8 vs. GOSE/-Q 5-6 -Male

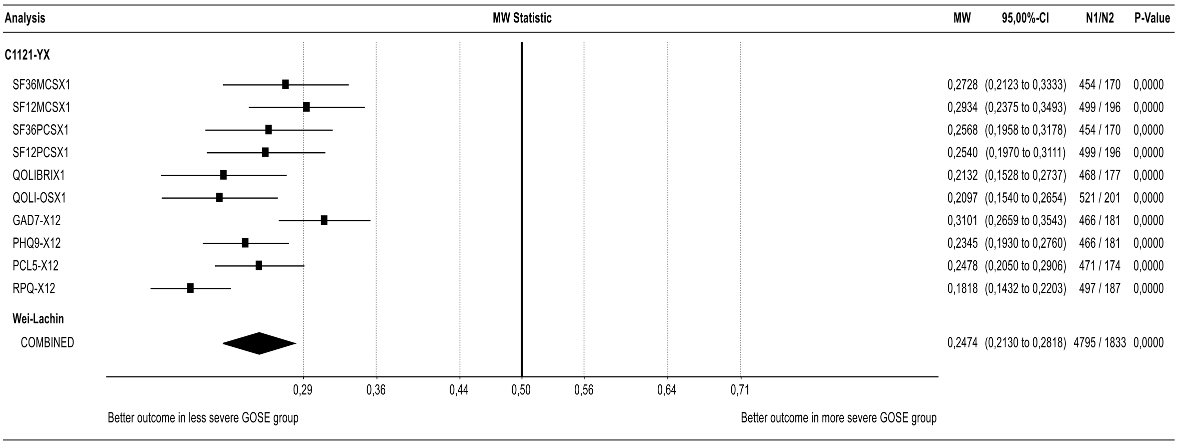

GOSE/-Q 5-6 vs. GOSE/-Q 3-4 - Male

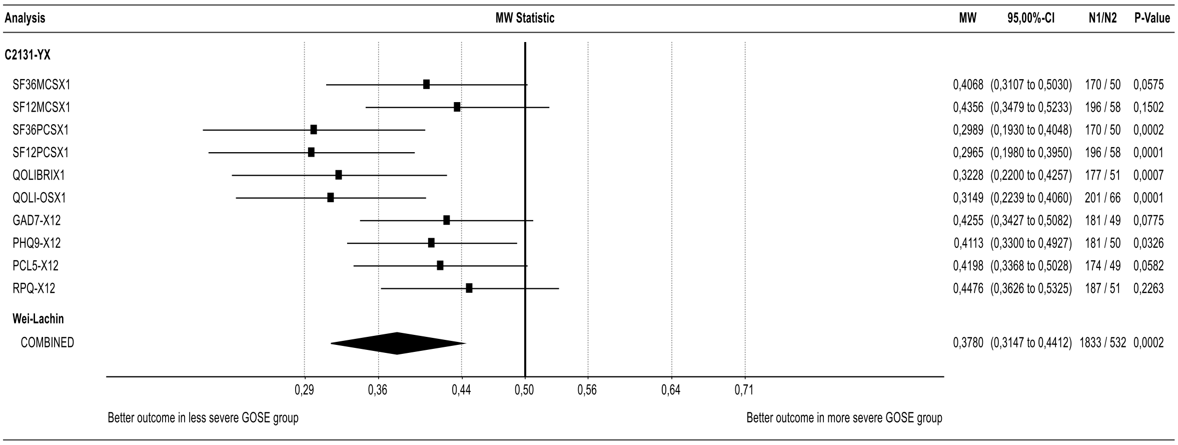

GOSE/-Q 7-8 vs. GOSE/-Q 3-4 – Male

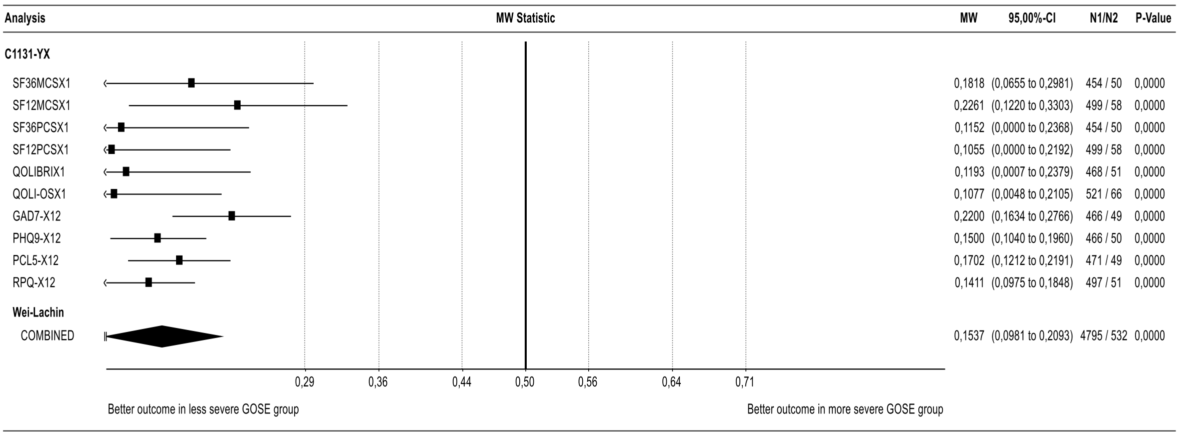

GOSE/-Q 7-8 vs. GOSE/-Q 5-6- Female

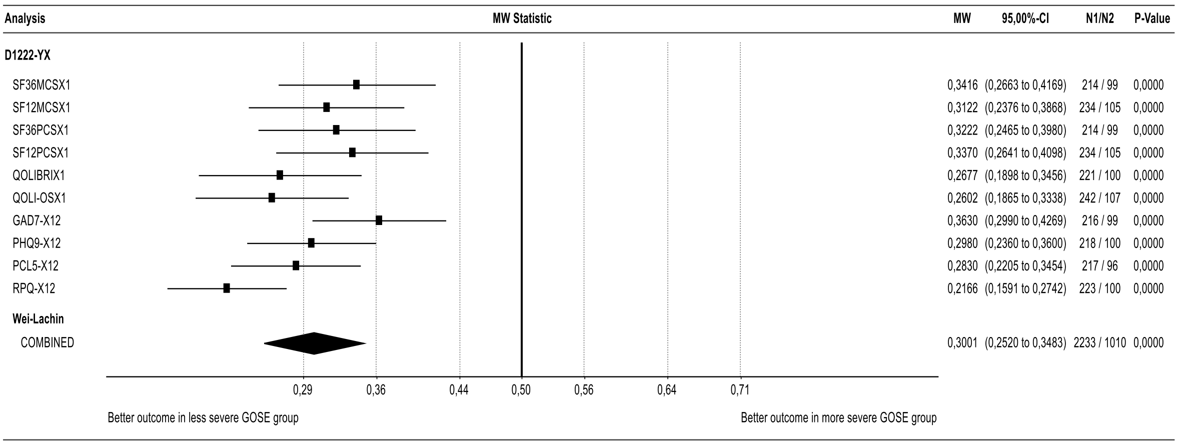

GOSE/-Q 5-6 vs. GOSE/-Q 3-4 - Female

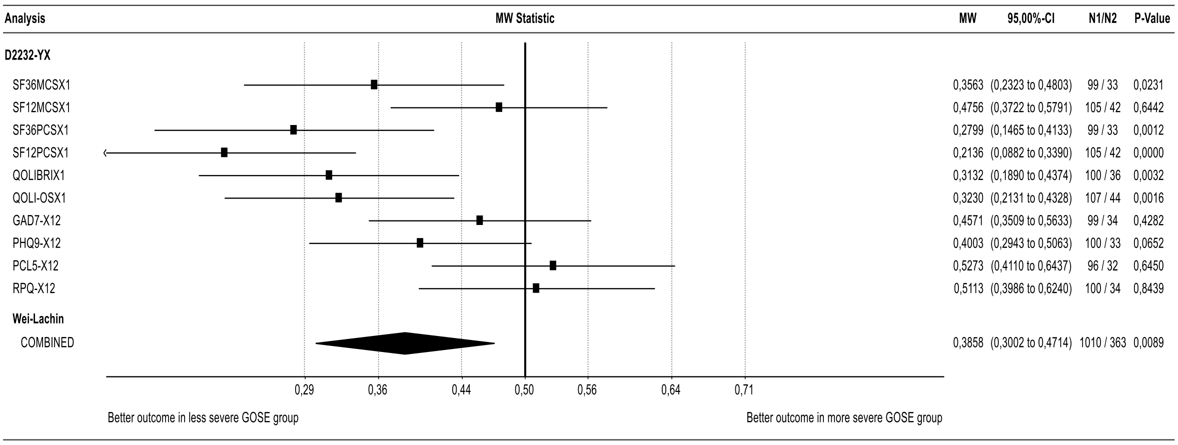

GOSE/-Q 7-8 vs. GOSE/-Q 3-4 - Female

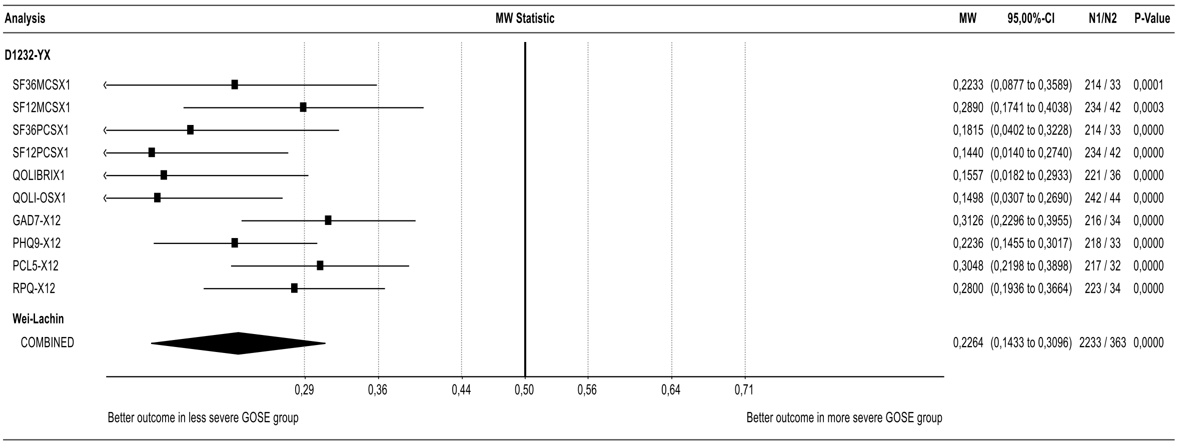

Age

3 months after TBI (data as available)

GOSE/-Q 7-8 vs. GOSE/-Q 5-6 -Age < 65

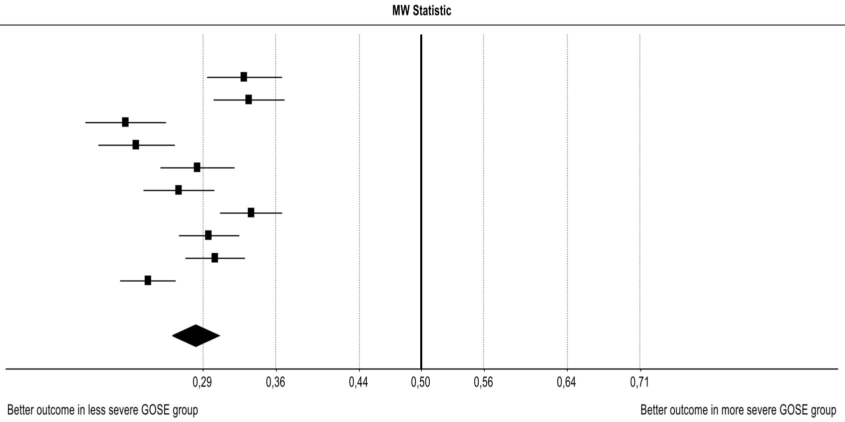

Analysis	MW	95,00%-CI	N1/N2	P-Value
**G1121-3D**				
SF36MCS-3	0,3299	(0,2939 to 0,3658)	878 / 463	0,0000
SF12MCS-3	0,3345	(0,3005 to 0,3685)	966 / 511	0,0000
SF36PCS-3	0,2160	(0,1774 to 0,2545)	878 / 463	0,0000
SF12PCS-3	0,2262	(0,1898 to 0,2627)	966 / 511	0,0000
QOLIBRI-3	0,2845	(0,2489 to 0,3202)	892 / 483	0,0000
QOLI-OS-3	0,2670	(0,2328 to 0,3011)	969 / 520	0,0000
GAD7-3	0,3365	(0,3067 to 0,3662)	872 / 480	0,0000
PHQ9-3	0,2958	(0,2669 to 0,3248)	877 / 480	0,0000
PCL5-3	0,3017	(0,2731 to 0,3302)	876 / 475	0,0000
RPQ-3	0,2371	(0,2104 to 0,2637)	912 / 496	0,0000
**Wei-Lachin**				
COMBINED	0,2829	(0,2604 to 0,3054)	9086 / 4882	0,0000

GOSE/-Q 5-6 vs. GOSE/-Q 3-4 -Age < 65

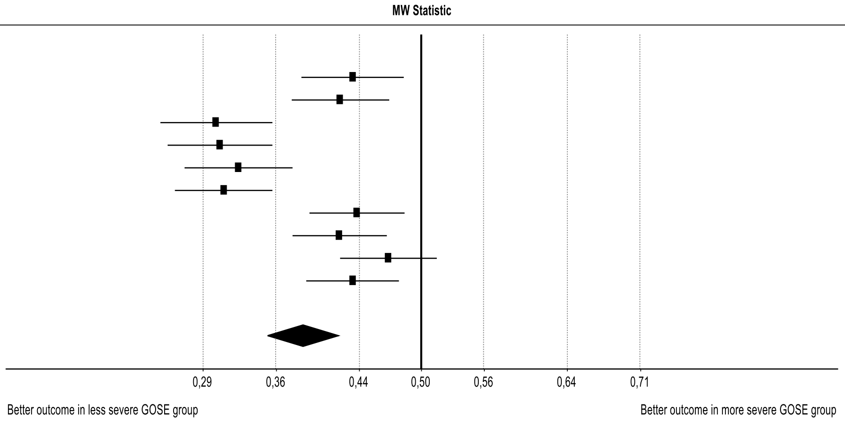

Analysis	MW	95,00%-CI	N1/N2	P-Value
**G2131-3D**				
SF36MCS-3	0,4338	(0,3846 to 0,4830)	463 / 207	0,0083
SF12MCS-3	0,4218	(0,3750 to 0,4687)	511 / 235	0,0011
SF36PCS-3	0,3026	(0,2487 to 0,3564)	463 / 207	0,0000
SF12PCS-3	0,3065	(0,2562 to 0,3567)	511 / 235	0,0000
QOLIBRI-3	0,3242	(0,2722 to 0,3762)	483 / 211	0,0000
QOLI-OS-3	0,3099	(0,2630 to 0,3568)	520 / 264	0,0000
GAD7-3	0,4383	(0,3926 to 0,4840)	480 / 205	0,0082
PHQ9-3	0,4212	(0,3759 to 0,4664)	480 / 207	0,0006
PCL5-3	0,4683	(0,4219 to 0,5147)	475 / 202	0,1811
RPQ-3	0,4338	(0,3890 to 0,4786)	496 / 209	0,0038
**Wei-Lachin**				
COMBINED	0,3860	(0,3521 to 0,4199)	4882 / 2182	0,0000

GOSE/-Q 7-8 vs. GOSE/-Q 3-4 -Age < 65

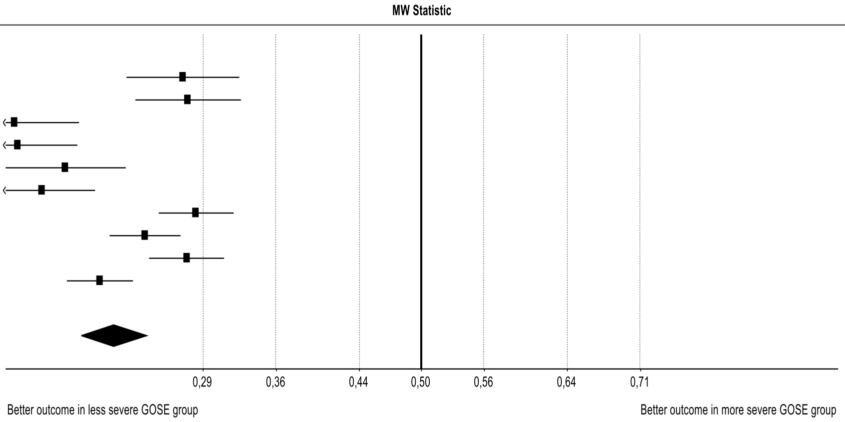

Analysis	MW	95,00%-CI	N1/N2	P-Value
**G1131-3D**				
SF36MCS-3	0,2709	(0,2166 to 0,3252)	878 / 207	0,0000
SF12MCS-3	0,2757	(0,2250 to 0,3264)	966 / 235	0,0000
SF36PCS-3	0,1091	(0,0476 to 0,1707)	878 / 207	0,0000
SF12PCS-3	0,1118	(0,0542 to 0,1695)	966 / 235	0,0000
QOLIBRI-3	0,1580	(0,1004 to 0,2156)	892 / 211	0,0000
QOLI-OS-3	0,1351	(0,0840 to 0,1861)	969 / 264	0,0000
GAD7-3	0,2835	(0,2472 to 0,3197)	872 / 205	0,0000
PHQ9-3	0,2344	(0,2001 to 0,2687)	877 / 207	0,0000
PCL5-3	0,2744	(0,2386 to 0,3103)	876 / 202	0,0000
RPQ-3	0,1911	(0,1596 to 0,2225)	912 / 209	0,0000
**Wei-Lachin**				
COMBINED	0,2044	(0,1731 to 0,2357)	9086 / 2182	0,0000

GOSE/-Q 7-8 vs. GOSE/-Q 5-6 –Age ≥ 65 Years

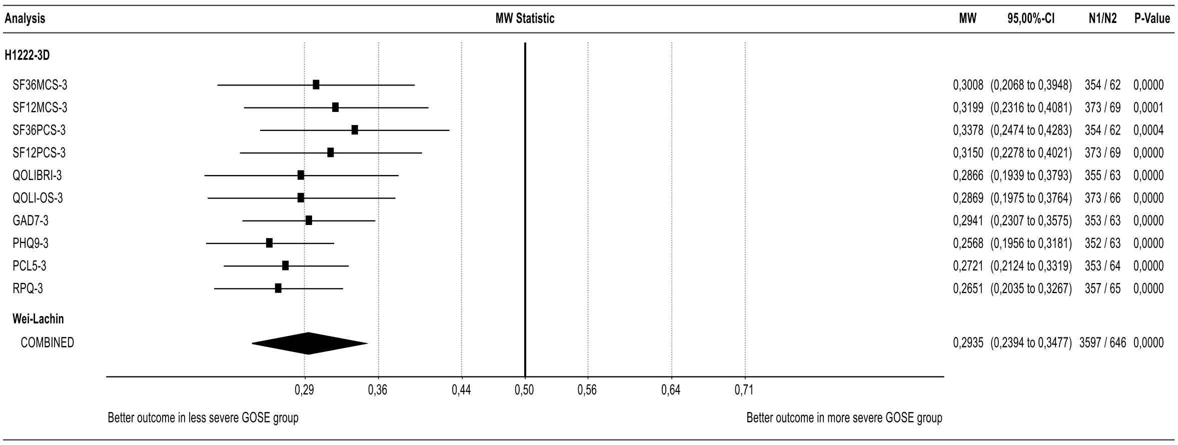

GOSE/-Q 5-6 vs. GOSE/-Q 3-4 - Age ≥ 65 Years

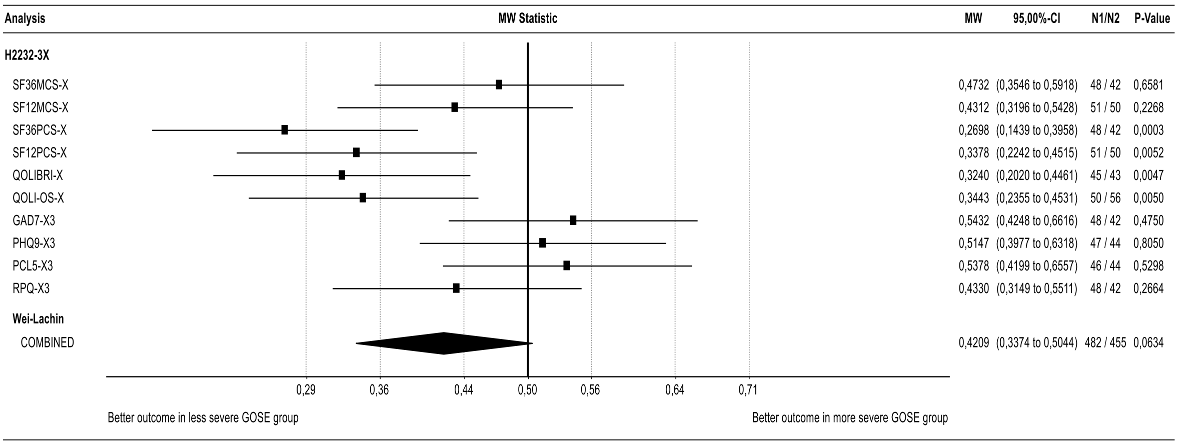

GOSE/-Q 7-8 vs. GOSE/-Q 3-4 - Age ≥ 65 Years

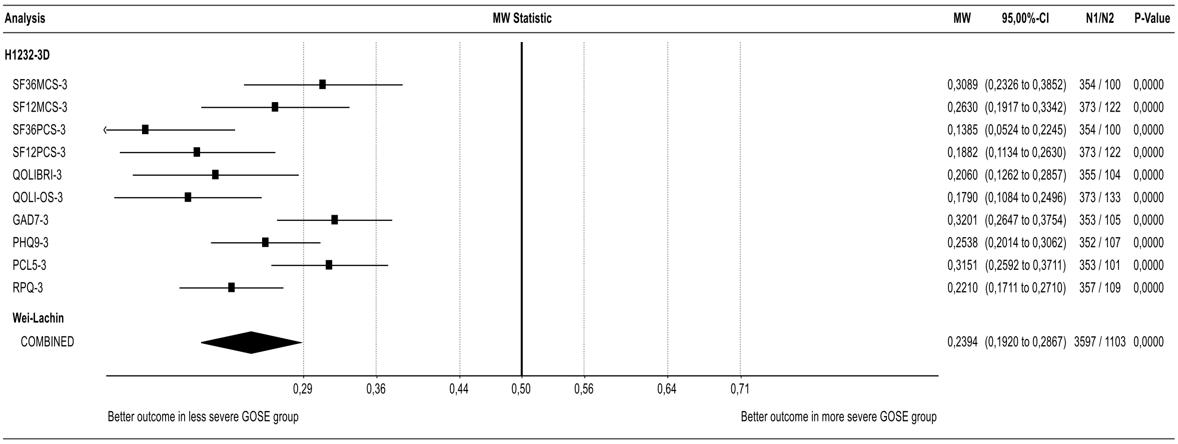

6 months after TBI (data as available)

GOSE/-Q 7-8 vs. GOSE/-Q 5-6 -Age < 65

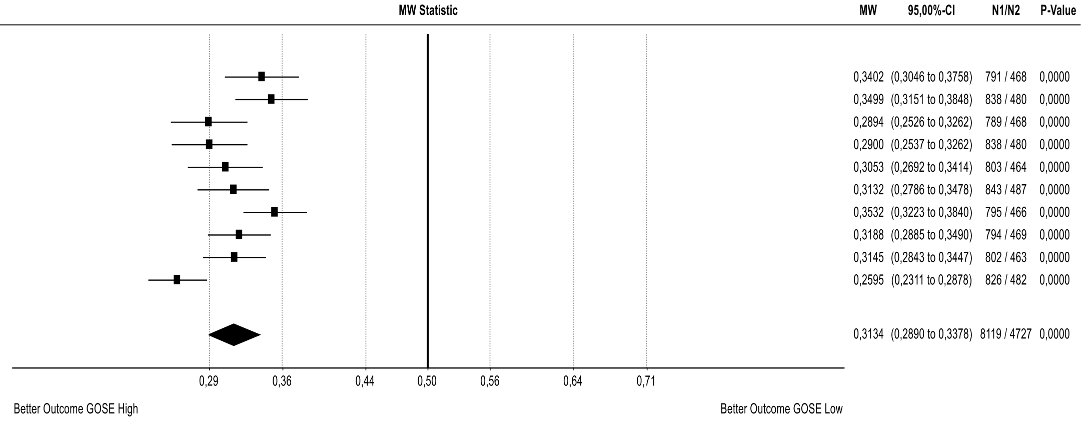

Analysis	MW	95,00%-CI	N1/N2	P-Value
**G1121-6D**				
SF36MCS-6	0,3402	(0,3046 to 0,3758)	791 / 468	0,0000
SF12MCS-6	0,3499	(0,3151 to 0,3848)	838 / 480	0,0000
SF36PCS-6	0,2894	(0,2526 to 0,3262)	789 / 468	0,0000
SF12PCS-6	0,2900	(0,2537 to 0,3262)	838 / 480	0,0000
QOLIBRI-6	0,3053	(0,2692 to 0,3414)	803 / 464	0,0000
QOLI-OS-6	0,3132	(0,2786 to 0,3478)	843 / 487	0,0000
GAD7-6	0,3532	(0,3223 to 0,3840)	795 / 466	0,0000
PHQ9-6	0,3188	(0,2885 to 0,3490)	794 / 469	0,0000
PCL5-6	0,3145	(0,2843 to 0,3447)	802 / 463	0,0000
RPQ-6	0,2595	(0,2311 to 0,2878)	826 / 482	0,0000
**Wei-Lachin**				
COMBINED	0,3134	(0,2890 to 0,3378)	8119 / 4727	0,0000

GOSE/-Q 5-6 vs. GOSE/-Q 3-4 -Age < 65

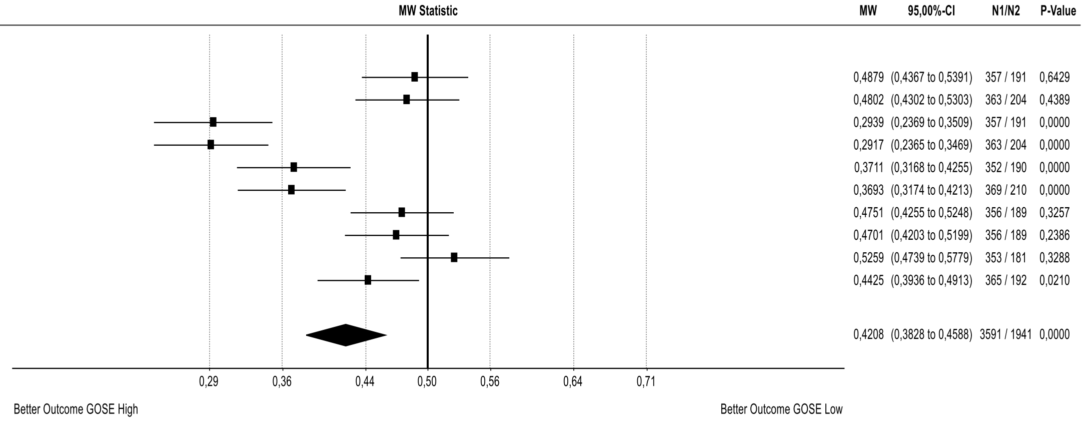

Analysis	MW	95,00%-CI	N1/N2	P-Value
**C2131-6D**				
SF36MCS-6	0,4879	(0,4367 to 0,5391)	357 / 191	0,6429
SF12MCS-6	0,4802	(0,4302 to 0,5303)	363 / 204	0,4389
SF36PCS-6	0,2939	(0,2369 to 0,3509)	357 / 191	0,0000
SF12PCS-6	0,2917	(0,2365 to 0,3469)	363 / 204	0,0000
QOLIBRI-6	0,3711	(0,3168 to 0,4255)	352 / 190	0,0000
QOLI-OS-6	0,3693	(0,3174 to 0,4213)	369 / 210	0,0000
GAD7-6	0,4751	(0,4255 to 0,5248)	356 / 189	0,3257
PHQ9-6	0,4701	(0,4203 to 0,5199)	356 / 189	0,2386
PCL5-6	0,5259	(0,4739 to 0,5779)	353 / 181	0,3288
RPQ-6	0,4425	(0,3936 to 0,4913)	365 / 192	0,0210
**Wei-Lachin**				
COMBINED	0,4208	(0,3828 to 0,4588)	3591 / 1941	0,0000

GOSE/-Q 7-8 vs. GOSE/-Q 3-4 -Age < 65

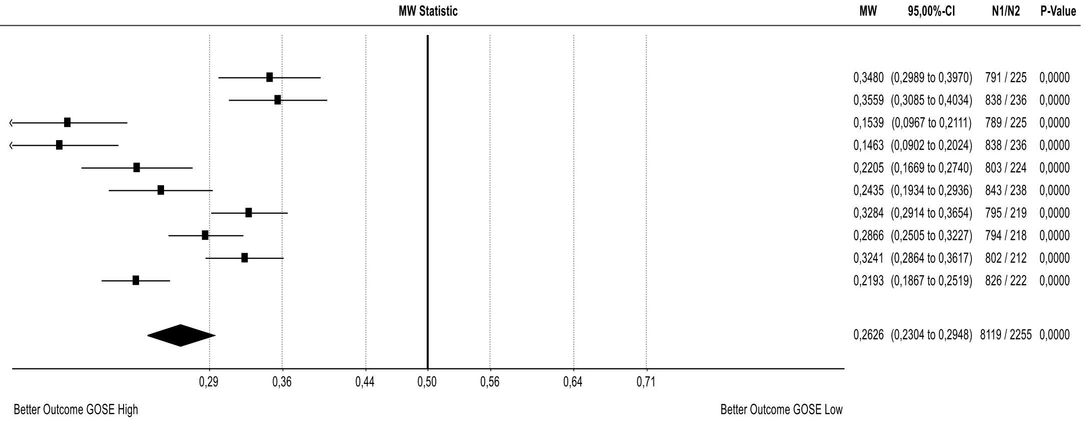

Analysis	MW	95,00%-CI	N1/N2	P-Value
**G1131-6D**				
SF36MCS-6	0,3480	(0,2989 to 0,3970)	791 / 225	0,0000
SF12MCS-6	0,3559	(0,3085 to 0,4034)	838 / 236	0,0000
SF36PCS-6	0,1539	(0,0967 to 0,2111)	789 / 225	0,0000
SF12PCS-6	0,1463	(0,0902 to 0,2024)	838 / 236	0,0000
QOLIBRI-6	0,2205	(0,1669 to 0,2740)	803 / 224	0,0000
QOLI-OS-6	0,2435	(0,1934 to 0,2936)	843 / 238	0,0000
GAD7-6	0,3284	(0,2914 to 0,3654)	795 / 219	0,0000
PHQ9-6	0,2866	(0,2505 to 0,3227)	794 / 218	0,0000
PCL5-6	0,3241	(0,2864 to 0,3617)	802 / 212	0,0000
RPQ-6	0,2193	(0,1867 to 0,2519)	826 / 222	0,0000
**Wei-Lachin**				
COMBINED	0,2626	(0,2304 to 0,2948)	8119 / 2255	0,0000

GOSE/-Q 7-8 vs. GOSE/-Q 5-6 - Age ≥ 65 Years

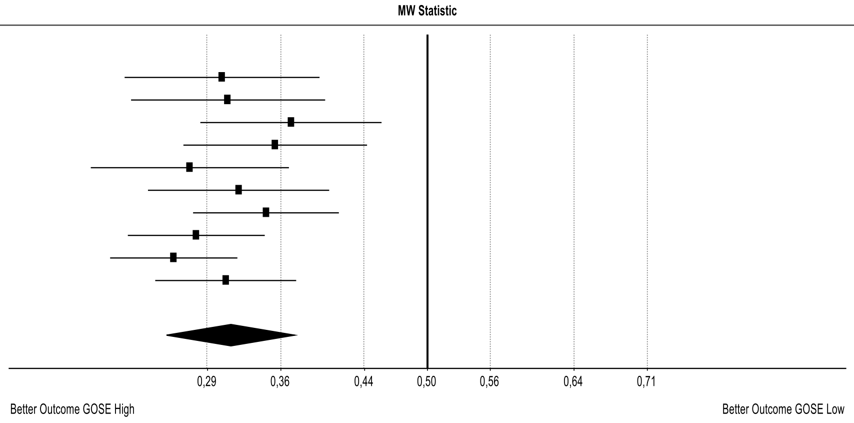

Analysis	MW	95,00%-CI	N1/N2	P-Value
**H1222-6D**				
SF36MCS-6	0,3042	(0,2110 to 0,3974)	316 / 64	0,0000
SF12MCS-6	0,3099	(0,2173 to 0,4026)	323 / 64	0,0001
SF36PCS-6	0,3701	(0,2836 to 0,4565)	316 / 64	0,0032
SF12PCS-6	0,3547	(0,2670 to 0,4425)	323 / 64	0,0012
QOLIBRI-6	0,2732	(0,1785 to 0,3680)	319 / 62	0,0000
QOLI-OS-6	0,3201	(0,2336 to 0,4066)	323 / 64	0,0000
GAD7-6	0,3462	(0,2765 to 0,4159)	314 / 63	0,0000
PHQ9-6	0,2798	(0,2145 to 0,3451)	315 / 63	0,0000
PCL5-6	0,2581	(0,1971 to 0,3191)	317 / 64	0,0000
RPQ-6	0,3078	(0,2406 to 0,3751)	320 / 63	0,0000
**Wei-Lachin**				
COMBINED	0,3124	(0,2513 to 0,3735)	3186 / 635	0,0000

GOSE/-Q 5-6 vs. GOSE/-Q 3-4 - Age ≥ 65 Years

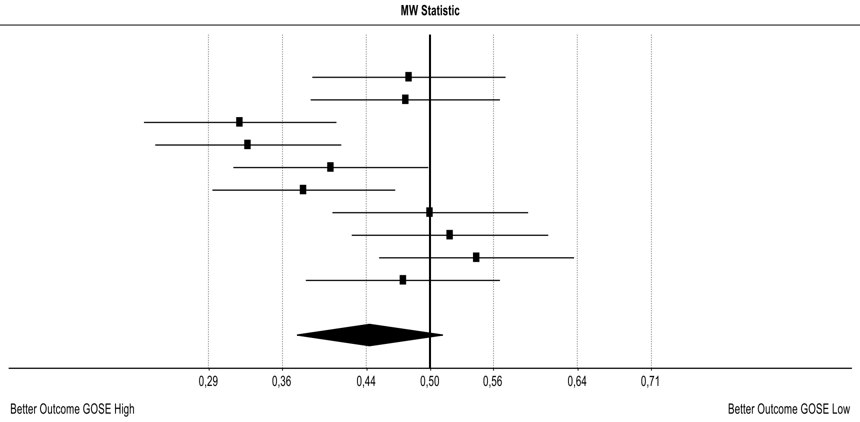

Analysis	MW	95,00%-CI	N1/N2	P-Value
**H2232-6D**				
SF36MCS-6	0,4799	(0,3882 to 0,5716)	64 / 87	0,6672
SF12MCS-6	0,4767	(0,3871 to 0,5664)	64 / 96	0,6109
SF36PCS-6	0,3200	(0,2288 to 0,4112)	64 / 87	0,0001
SF12PCS-6	0,3276	(0,2395 to 0,4158)	64 / 96	0,0001
QOLIBRI-6	0,4060	(0,3134 to 0,4986)	62 / 83	0,0467
QOLI-OS-6	0,3804	(0,2938 to 0,4669)	64 / 99	0,0067
GAD7-6	0,5003	(0,4076 to 0,5929)	63 / 84	0,9952
PHQ9-6	0,5192	(0,4262 to 0,6122)	63 / 86	0,6859
PCL5-6	0,5441	(0,4518 to 0,6365)	64 / 83	0,3486
RPQ-6	0,4746	(0,3826 to 0,5666)	63 / 91	0,5887
**Wei-Lachin**				
COMBINED	0,4429	(0,3739 to 0,5119)	635 / 892	0,1049

GOSE/-Q 7-8 vs. GOSE/-Q 3-4 - Age ≥ 65 Years

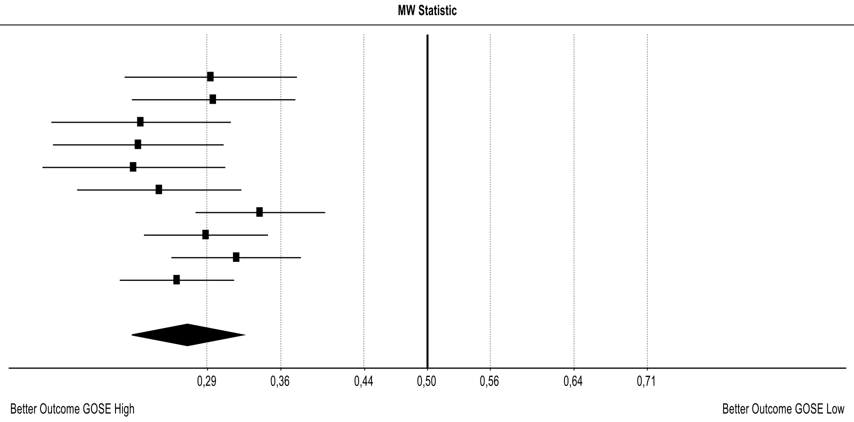

Analysis	MW	95,00%-CI	N1/N2	P-Value
**H1232-6D**				
SF36MCS-6	0,2938	(0,2115 to 0,3761)	316 / 87	0,0000
SF12MCS-6	0,2960	(0,2179 to 0,3742)	323 / 96	0,0000
SF36PCS-6	0,2267	(0,1410 to 0,3124)	316 / 87	0,0000
SF12PCS-6	0,2243	(0,1427 to 0,3060)	323 / 96	0,0000
QOLIBRI-6	0,2199	(0,1326 to 0,3072)	319 / 83	0,0000
QOLI-OS-6	0,2442	(0,1657 to 0,3227)	323 / 99	0,0000
GAD7-6	0,3406	(0,2788 to 0,4024)	314 / 84	0,0000
PHQ9-6	0,2890	(0,2296 to 0,3484)	315 / 86	0,0000
PCL5-6	0,3177	(0,2560 to 0,3794)	317 / 83	0,0000
RPQ-6	0,2613	(0,2067 to 0,3160)	320 / 91	0,0000
**Wei-Lachin**				
COMBINED	0,2714	(0,2184 to 0,3244)	3186 / 892	0,0000

12 months after TBI (data as available)

GOSE/-Q 7-8 vs. GOSE/-Q 5-6 -Age < 65

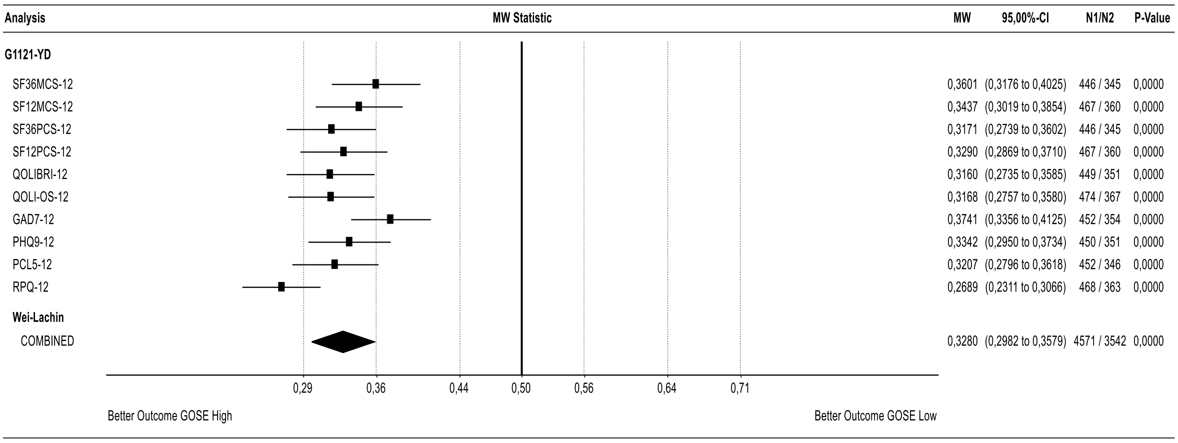

GOSE/-Q 5-6 vs. GOSE/-Q 3-4 -Age < 65

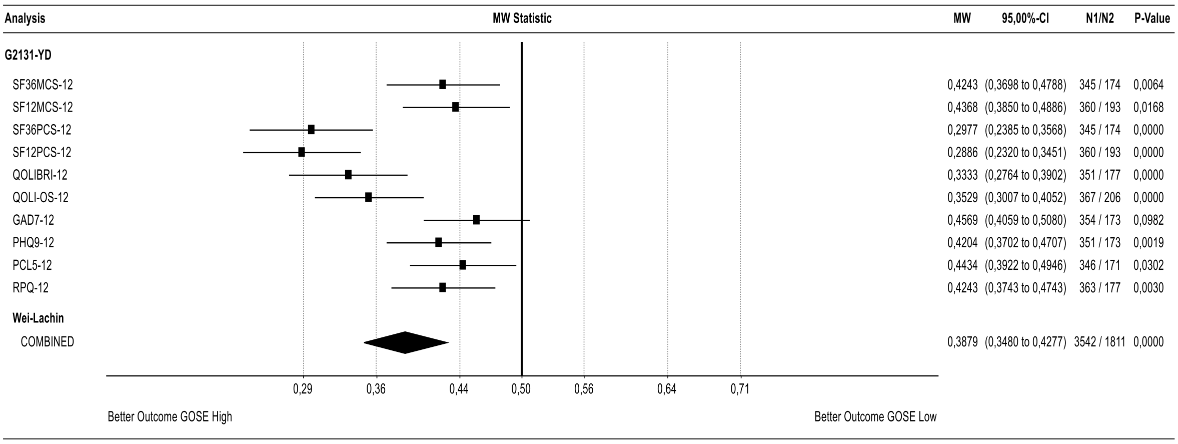

GOSE/-Q 7-8 vs. GOSE/-Q 3-4 -Age < 65

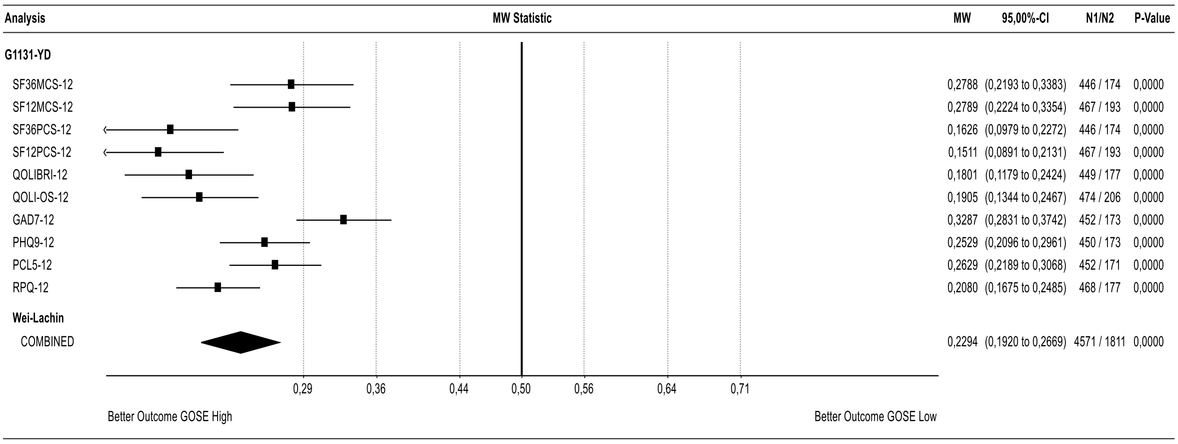

GOSE/-Q 7-8 vs. GOSE/-Q 5-6 - Age ≥ 65 Years

Analysis	MW	95,00%-CI	N1/N2	P-Value
**H1222-YD**				
SF36MCS-12	0,3745	(0,2760 to 0,4729)	195 / 50	0,0124
SF12MCS-12	0,3697	(0,2729 to 0,4666)	208 / 52	0,0084
SF36PCS-12	0,3876	(0,2900 to 0,4852)	195 / 50	0,0239
SF12PCS-12	0,3854	(0,2892 to 0,4817)	208 / 52	0,0196
QOLIBRI-12	0,3132	(0,2073 to 0,4190)	201 / 47	0,0005
QOLI-OS-12	0,3668	(0,2724 to 0,4612)	213 / 52	0,0057
GAD7-12	0,3719	(0,2920 to 0,4519)	204 / 51	0,0017
PHQ9-12	0,3319	(0,2525 to 0,4114)	202 / 50	0,0000
PCL5-12	0,3444	(0,2652 to 0,4236)	201 / 48	0,0001
RPQ-12	0,3373	(0,2578 to 0,4168)	204 / 51	0,0001
**Wei-Lachin**				
COMBINED	0,3583	(0,2913 to 0,4253)	2031 / 503	0,0000

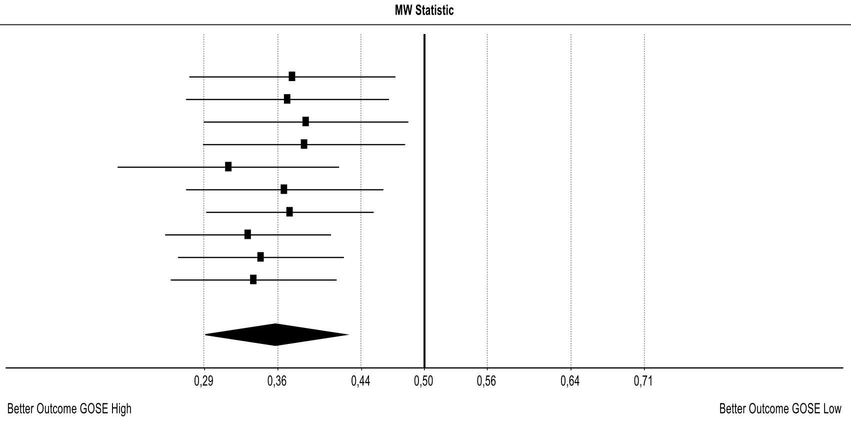

GOSE/-Q 5-6 vs. GOSE/-Q 3-4 - Age ≥ 65 Years

Analysis	MW	95,00%-CI	N1/N2	P-Value
**H2232-YD**				
SF36MCS-12	0,3970	(0,2939 to 0,5000)	50 / 66	0,0501
SF12MCS-12	0,4657	(0,3673 to 0,5642)	52 / 78	0,4950
SF36PCS-12	0,3680	(0,2643 to 0,4718)	50 / 66	0,0126
SF12PCS-12	0,3792	(0,2811 to 0,4773)	52 / 78	0,0158
QOLIBRI-12	0,3622	(0,2579 to 0,4665)	47 / 66	0,0096
QOLI-OS-12	0,3761	(0,2808 to 0,4715)	52 / 84	0,0109
GAD7-12	0,4394	(0,3379 to 0,5410)	51 / 68	0,2427
PHQ9-12	0,4235	(0,3182 to 0,5288)	50 / 68	0,1546
PCL5-12	0,4017	(0,2936 to 0,5097)	48 / 68	0,0745
RPQ-12	0,4534	(0,3502 to 0,5566)	51 / 69	0,3763
**Wei-Lachin**				
COMBINED	0,4066	(0,3323 to 0,4809)	503 / 711	0,0138

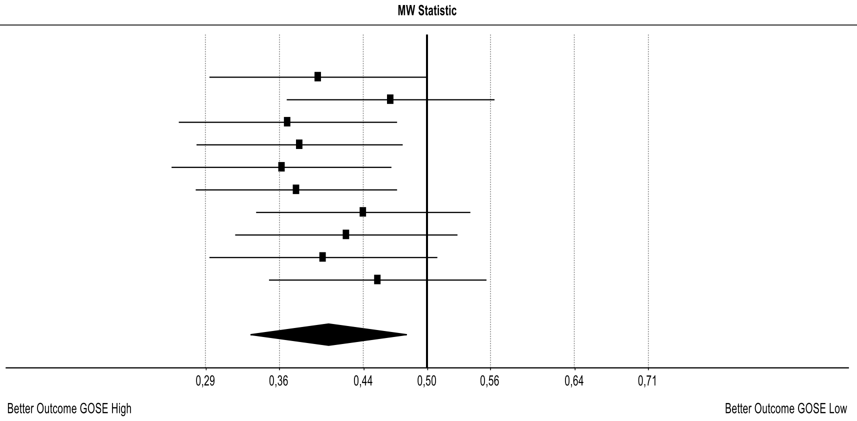

GOSE/-Q 7-8 vs. GOSE/-Q 3-4 - Age ≥ 65 Years

Analysis	MW	95,00%-CI	N1/N2	P-Value
**H1232-YD**				
SF36MCS-12	0,3038	(0,2101 to 0,3976)	195 / 66	0,0000
SF12MCS-12	0,3643	(0,2811 to 0,4474)	208 / 78	0,0014
SF36PCS-12	0,2594	(0,1636 to 0,3553)	195 / 66	0,0000
SF12PCS-12	0,2620	(0,1739 to 0,3500)	208 / 78	0,0000
QOLIBRI-12	0,2280	(0,1296 to 0,3263)	201 / 66	0,0000
QOLI-OS-12	0,2799	(0,1966 to 0,3631)	213 / 84	0,0000
GAD7-12	0,2970	(0,2277 to 0,3664)	204 / 68	0,0000
PHQ9-12	0,2591	(0,1918 to 0,3264)	202 / 68	0,0000
PCL5-12	0,2869	(0,2164 to 0,3573)	201 / 68	0,0000
RPQ-12	0,2889	(0,2205 to 0,3573)	204 / 69	0,0000
**Wei-Lachin**				
COMBINED	0,2829	(0,2247 to 0,3412)	2031 / 711	0,0000

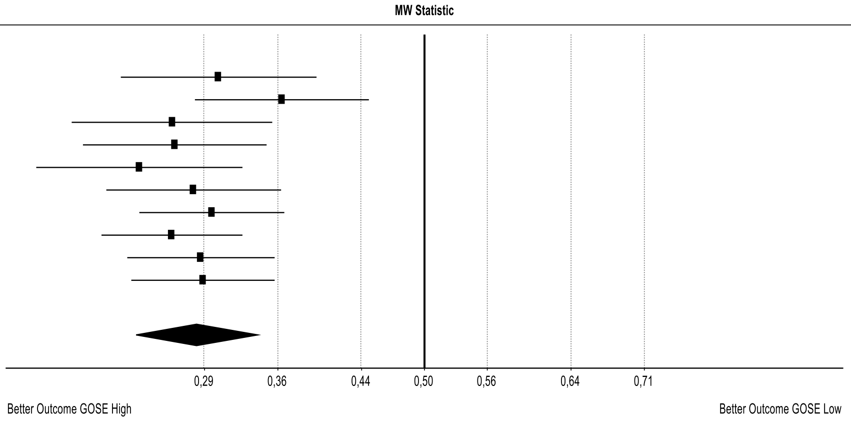

3 months after TBI (completers)

GOSE/-Q 7-8 vs. GOSE/-Q 5-6 - Age < 65

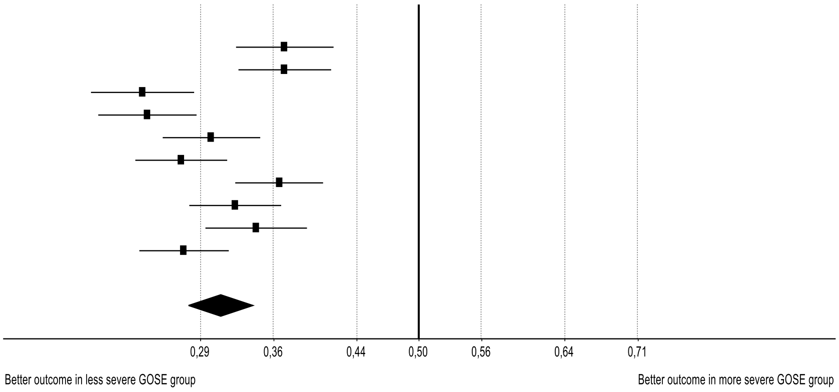

Analysis	MW	95,00%-CI	N1/N2	P-Value
**G1121-3X**				
SF36MCS-X	0,3708	(0,3241 to 0,4176)	361 / 281	0,0000
SF12MCS-X	0,3709	(0,3266 to 0,4153)	401 / 311	0,0000
SF36PCS-X	0,2346	(0,1849 to 0,2842)	361 / 281	0,0000
SF12PCS-X	0,2387	(0,1916 to 0,2859)	401 / 311	0,0000
QOLIBRI-X	0,3003	(0,2535 to 0,3471)	367 / 293	0,0000
QOLI-OS-X	0,2716	(0,2274 to 0,3157)	412 / 324	0,0000
GAD7-X3	0,3658	(0,3233 to 0,4082)	366 / 298	0,0000
PHQ9-X3	0,3234	(0,2793 to 0,3675)	366 / 300	0,0000
PCL5-X3	0,3434	(0,2947 to 0,3921)	369 / 291	0,0000
RPQ-X3	0,2742	(0,2311 to 0,3173)	392 / 314	0,0000
**Wei-Lachin**				
COMBINED	0,3094	(0,2786 to 0,3402)	3796 / 3004	0,0000

GOSE/-Q 5-6 vs. GOSE/-Q 3-4 - Age < 65

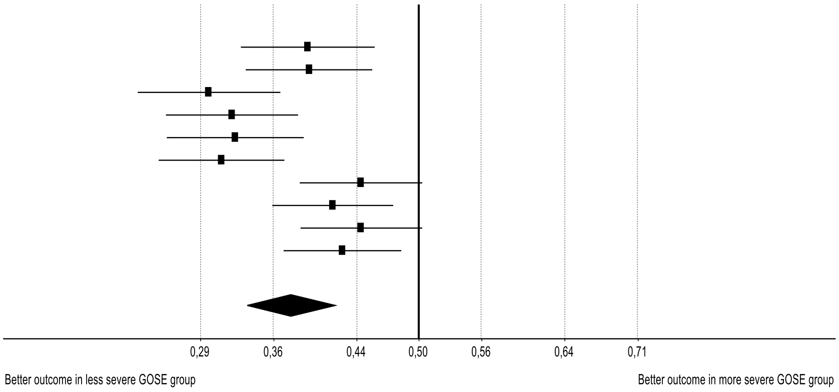

Analysis	MW	95,00%-CI	N1/N2	P-Value
**G2131-3X**				
SF36MCS-X	0,3931	(0,3285 to 0,4576)	281 / 128	0,0012
SF12MCS-X	0,3946	(0,3338 to 0,4554)	311 / 145	0,0007
SF36PCS-X	0,2981	(0,2296 to 0,3666)	281 / 128	0,0000
SF12PCS-X	0,3204	(0,2571 to 0,3836)	311 / 145	0,0000
QOLIBRI-X	0,3234	(0,2578 to 0,3890)	293 / 132	0,0000
QOLI-OS-X	0,3103	(0,2499 to 0,3706)	324 / 157	0,0000
GAD7-X3	0,4442	(0,3856 to 0,5029)	298 / 123	0,0626
PHQ9-X3	0,4173	(0,3594 to 0,4752)	300 / 124	0,0051
PCL5-X3	0,4444	(0,3859 to 0,5029)	291 / 120	0,0627
RPQ-X3	0,4266	(0,3699 to 0,4832)	314 / 127	0,0111
**Wei-Lachin**				
COMBINED	0,3772	(0,3352 to 0,4193)	3004 / 1329	0,0000

GOSE/-Q 7-8 vs. GOSE/-Q 3-4 - Age < 65

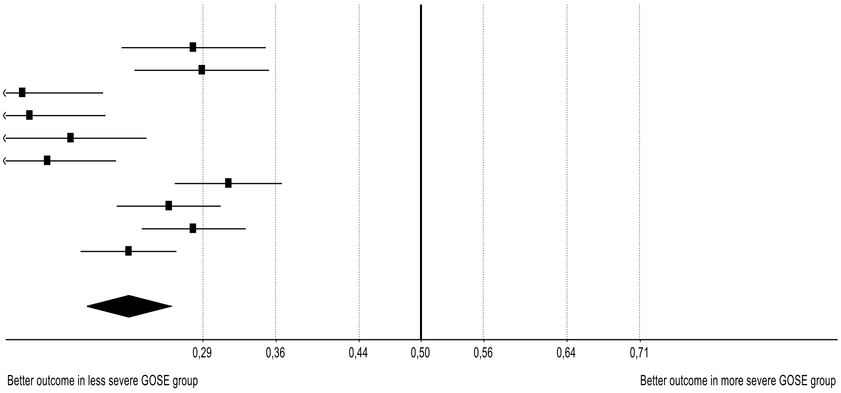

Analysis	MW	95,00%-CI	N1/N2	P-Value
**G1131-3X**				
SF36MCS-X	0,2811	(0,2116 to 0,3505)	361 / 128	0,0000
SF12MCS-X	0,2891	(0,2244 to 0,3539)	401 / 145	0,0000
SF36PCS-X	0,1163	(0,0387 to 0,1940)	361 / 128	0,0000
SF12PCS-X	0,1236	(0,0510 to 0,1963)	401 / 145	0,0000
QOLIBRI-X	0,1632	(0,0905 to 0,2359)	367 / 132	0,0000
QOLI-OS-X	0,1408	(0,0753 to 0,2063)	412 / 157	0,0000
GAD7-X3	0,3149	(0,2633 to 0,3664)	366 / 123	0,0000
PHQ9-X3	0,2575	(0,2075 to 0,3075)	366 / 124	0,0000
PCL5-X3	0,2812	(0,2310 to 0,3314)	369 / 120	0,0000
RPQ-X3	0,2185	(0,1722 to 0,2648)	392 / 127	0,0000
**Wei-Lachin**				
COMBINED	0,2186	(0,1783 to 0,2590)	3796 / 1329	0,0000

GOSE/-Q 7-8 vs. GOSE/-Q 5-6 – Age ≥ 65 Years

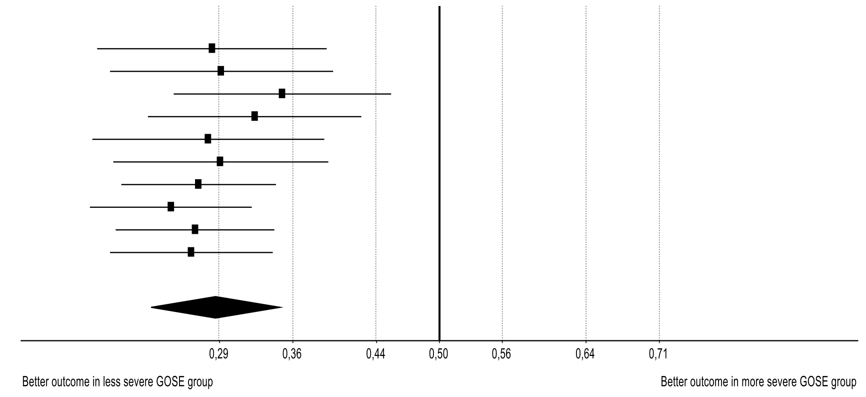

Analysis	MW	95,00%-CI	N1/N2	P-Value
**H1222-3X**				
SF36MCS-X	0,2832	(0,1737 to 0,3926)	169 / 48	0,0001
SF12MCS-X	0,2922	(0,1858 to 0,3986)	183 / 51	0,0001
SF36PCS-X	0,3504	(0,2465 to 0,4543)	169 / 48	0,0048
SF12PCS-X	0,3240	(0,2219 to 0,4260)	183 / 51	0,0007
QOLIBRI-X	0,2796	(0,1687 to 0,3905)	178 / 45	0,0001
QOLI-OS-X	0,2915	(0,1885 to 0,3945)	189 / 50	0,0001
GAD7-X3	0,2704	(0,1967 to 0,3441)	176 / 48	0,0000
PHQ9-X3	0,2439	(0,1669 to 0,3210)	173 / 47	0,0000
PCL5-X3	0,2670	(0,1914 to 0,3425)	175 / 46	0,0000
RPQ-X3	0,2633	(0,1854 to 0,3412)	176 / 48	0,0000
**Wei-Lachin**				
COMBINED	0,2865	(0,2250 to 0,3481)	1771 / 482	0,0000

GOSE/-Q 5-6 vs. GOSE/-Q 3-4 - Age ≥ 65 Years

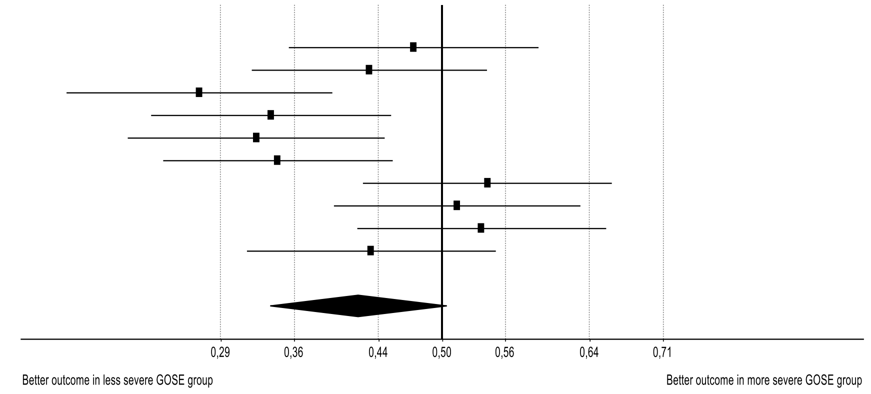

Analysis	MW	95,00%-CI	N1/N2	P-Value
**H2232-3X**				
SF36MCS-X	0,4732	(0,3546 to 0,5918)	48 / 42	0,6581
SF12MCS-X	0,4312	(0,3196 to 0,5428)	51 / 50	0,2268
SF36PCS-X	0,2698	(0,1439 to 0,3958)	48 / 42	0,0003
SF12PCS-X	0,3378	(0,2242 to 0,4515)	51 / 50	0,0052
QOLIBRI-X	0,3240	(0,2020 to 0,4461)	45 / 43	0,0047
QOLI-OS-X	0,3443	(0,2355 to 0,4531)	50 / 56	0,0050
GAD7-X3	0,5432	(0,4248 to 0,6616)	48 / 42	0,4750
PHQ9-X3	0,5147	(0,3977 to 0,6318)	47 / 44	0,8050
PCL5-X3	0,5378	(0,4199 to 0,6557)	46 / 44	0,5298
RPQ-X3	0,4330	(0,3149 to 0,5511)	48 / 42	0,2664
**Wei-Lachin**				
COMBINED	0,4209	(0,3374 to 0,5044)	482 / 455	0,0634

GOSE/-Q 7-8 vs. GOSE/-Q 3-4 - Age ≥ 65 Years

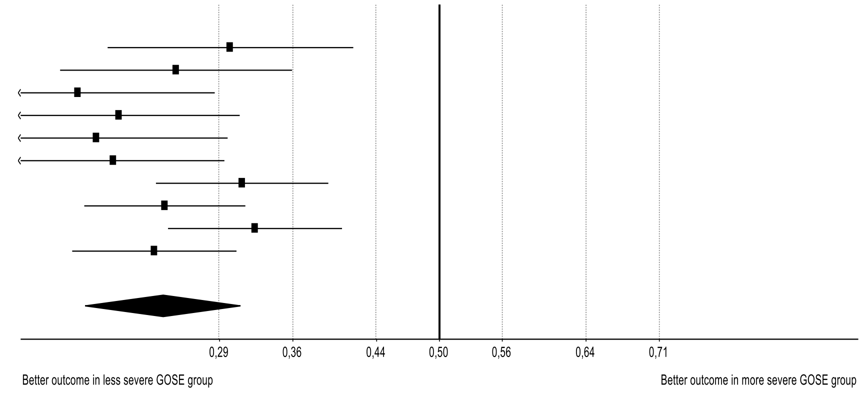

Analysis	MW	95,00%-CI	N1/N2	P-Value
**H1232-3X**				
SF36MCS-X	0,3004	(0,1831 to 0,4177)	169 / 42	0,0009
SF12MCS-X	0,2487	(0,1377 to 0,3597)	183 / 50	0,0000
SF36PCS-X	0,1550	(0,0243 to 0,2857)	169 / 42	0,0000
SF12PCS-X	0,1945	(0,0795 to 0,3096)	183 / 50	0,0000
QOLIBRI-X	0,1727	(0,0476 to 0,2978)	178 / 43	0,0000
QOLI-OS-X	0,1892	(0,0836 to 0,2949)	189 / 56	0,0000
GAD7-X3	0,3120	(0,2296 to 0,3944)	176 / 42	0,0000
PHQ9-X3	0,2381	(0,1615 to 0,3147)	173 / 44	0,0000
PCL5-X3	0,3243	(0,2411 to 0,4075)	175 / 44	0,0000
RPQ-X3	0,2281	(0,1498 to 0,3064)	176 / 42	0,0000
**Wei-Lachin**				
COMBINED	0,2363	(0,1623 to 0,3103)	1771 / 455	0,0000

6 months after TBI (completers)

GOSE/-Q 7-8 vs. GOSE/-Q 5-6 - Age < 65

Analysis	MW	95,00%-CI	N1/N2	P-Value
**G1121-6X**				
SF36MCS-X	0,3279	(0,2808 to 0,3749)	424 / 277	0,0000
SF12MCS-X	0,3440	(0,3001 to 0,3878)	462 / 316	0,0000
SF36PCS-X	0,2352	(0,1856 to 0,2848)	424 / 277	0,0000
SF12PCS-X	0,2240	(0,1771 to 0,2708)	462 / 316	0,0000
QOLIBRI-X	0,2785	(0,2319 to 0,3251)	428 / 294	0,0000
QOLI-OS-X	0,2755	(0,2319 to 0,3191)	476 / 327	0,0000
GAD7-X6	0,3554	(0,3145 to 0,3963)	428 / 295	0,0000
PHQ9-X6	0,2830	(0,2413 to 0,3247)	430 / 297	0,0000
PCL5-X6	0,3127	(0,2672 to 0,3582)	428 / 289	0,0000
RPQ-X6	0,2183	(0,1793 to 0,2572)	459 / 305	0,0000
**Wei-Lachin**				
COMBINED	0,2854	(0,2556 to 0,3153)	4421 / 2993	0,0000

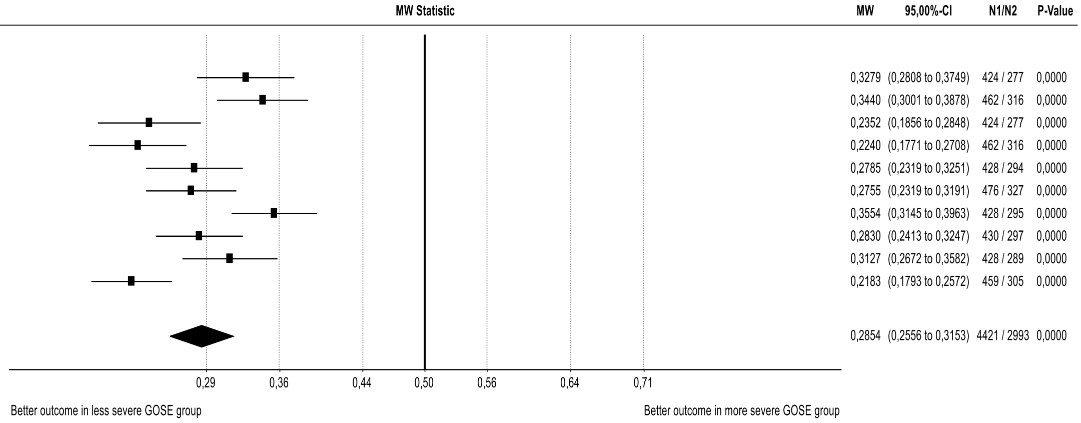

GOSE/-Q 5-6 vs. GOSE/-Q 3-4 -Age < 65

Analysis	MW	95,00%-CI	N1/N2	P-Value
**G2131-6X**				
SF36MCS-X	0,4290	(0,3455 to 0,5124)	277 / 65	0,0952
SF12MCS-X	0,4199	(0,3409 to 0,4989)	316 / 75	0,0469
SF36PCS-X	0,3307	(0,2395 to 0,4219)	277 / 65	0,0003
SF12PCS-X	0,3317	(0,2468 to 0,4167)	316 / 75	0,0001
QOLIBRI-X	0,3378	(0,2496 to 0,4260)	294 / 66	0,0003
QOLI-OS-X	0,3782	(0,3024 to 0,4539)	327 / 86	0,0016
GAD7-X6	0,4571	(0,3831 to 0,5311)	295 / 62	0,2557
PHQ9-X6	0,4434	(0,3696 to 0,5172)	297 / 63	0,1328
PCL5-X6	0,5018	(0,4240 to 0,5797)	289 / 62	0,9630
RPQ-X6	0,4680	(0,3936 to 0,5423)	305 / 67	0,3984
**Wei-Lachin**				
COMBINED	0,4098	(0,3529 to 0,4667)	2993 / 686	0,0019

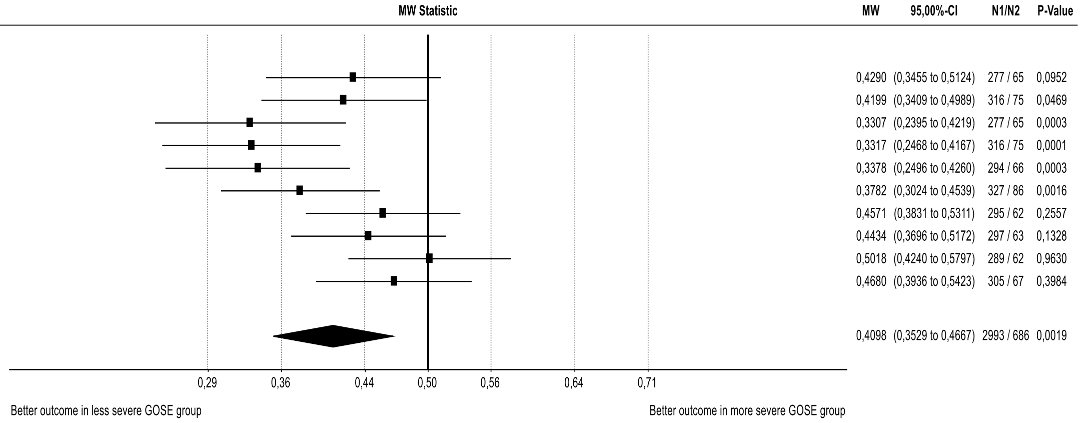

GOSE/-Q 7-8 vs. GOSE/-Q 3-4 -Age < 65

Analysis	MW	95,00%-CI	N1/N2	P-Value
**G1131-6X**				
SF36MCS-X	0,2878	(0,1929 to 0,3827)	424 / 65	0,0000
SF12MCS-X	0,2976	(0,2094 to 0,3858)	462 / 75	0,0000
SF36PCS-X	0,1400	(0,0342 to 0,2458)	424 / 65	0,0000
SF12PCS-X	0,1368	(0,0375 to 0,2362)	462 / 75	0,0000
QOLIBRI-X	0,1574	(0,0556 to 0,2592)	428 / 66	0,0000
QOLI-OS-X	0,1969	(0,1117 to 0,2822)	476 / 86	0,0000
GAD7-X6	0,2965	(0,2346 to 0,3584)	428 / 62	0,0000
PHQ9-X6	0,2337	(0,1776 to 0,2899)	430 / 63	0,0000
PCL5-X6	0,3087	(0,2460 to 0,3714)	428 / 62	0,0000
RPQ-X6	0,2258	(0,1694 to 0,2821)	459 / 67	0,0000
**Wei-Lachin**				
COMBINED	0,2281	(0,1731 to 0,2832)	4421 / 686	0,0000

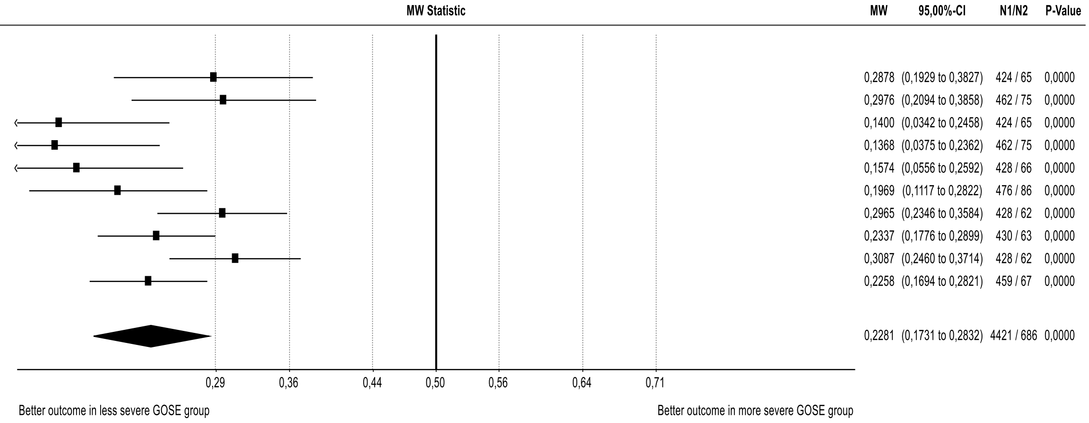

GOSE/-Q 7-8 vs. GOSE/-Q 5-6 - Age ≥ 65 Years

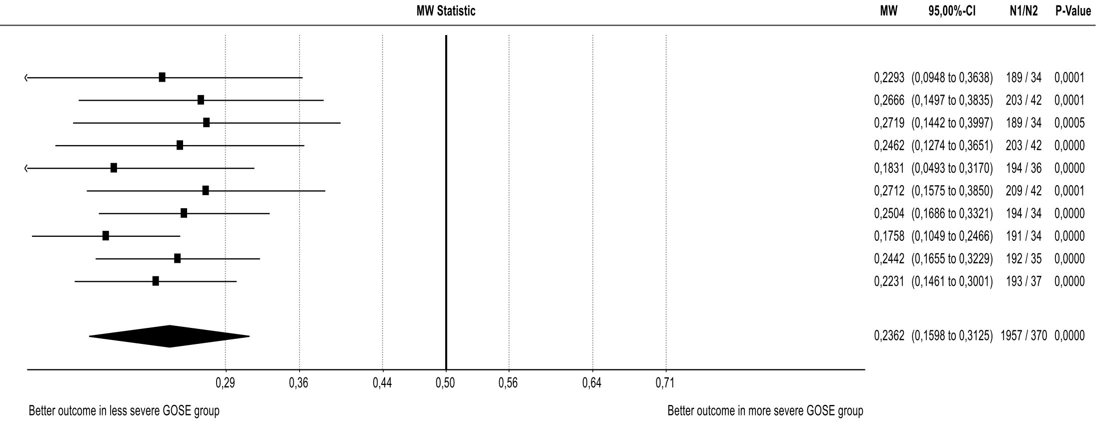

Analysis	MW	95,00%-CI	N1/N2	P-Value
**H1222-6X**				
SF36MCS-X	0,2293	(0,0948 to 0,3638)	189 / 34	0,0001
SF12MCS-X	0,2666	(0,1497 to 0,3835)	203 / 42	0,0001
SF36PCS-X	0,2719	(0,1442 to 0,3997)	189 / 34	0,0005
SF12PCS-X	0,2462	(0,1274 to 0,3651)	203 / 42	0,0000
QOLIBRI-X	0,1831	(0,0493 to 0,3170)	194 / 36	0,0000
QOLI-OS-X	0,2712	(0,1575 to 0,3850)	209 / 42	0,0001
GAD7-X6	0,2504	(0,1686 to 0,3321)	194 / 34	0,0000
PHQ9-X6	0,1758	(0,1049 to 0,2466)	191 / 34	0,0000
PCL5-X6	0,2442	(0,1655 to 0,3229)	192 / 35	0,0000
RPQ-X6	0,2231	(0,1461 to 0,3001)	193 / 37	0,0000
**Wei-Lachin**				
COMBINED	0,2362	(0,1598 to 0,3125)	1957 / 370	0,0000

GOSE/-Q 5-6 vs. GOSE/-Q 3-4 - Age ≥ 65 Years

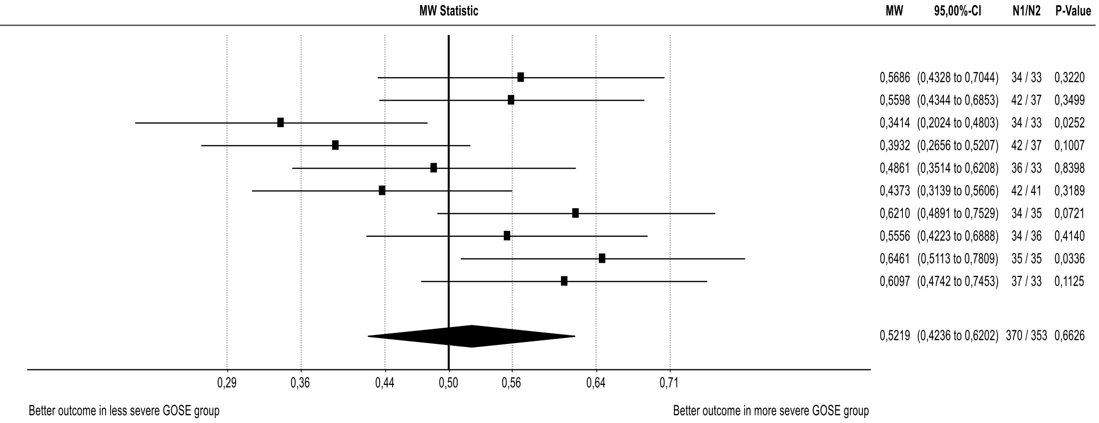

Analysis	MW	95,00%-CI	N1/N2	P-Value
**H2232-6X**				
SF36MCS-X	0,5686	(0,4328 to 0,7044)	34 / 33	0,3220
SF12MCS-X	0,5598	(0,4344 to 0,6853)	42 / 37	0,3499
SF36PCS-X	0,3414	(0,2024 to 0,4803)	34 / 33	0,0252
SF12PCS-X	0,3932	(0,2656 to 0,5207)	42 / 37	0,1007
QOLIBRI-X	0,4861	(0,3514 to 0,6208)	36 / 33	0,8398
QOLI-OS-X	0,4373	(0,3139 to 0,5606)	42 / 41	0,3189
GAD7-X6	0,6210	(0,4891 to 0,7529)	34 / 35	0,0721
PHQ9-X6	0,5556	(0,4223 to 0,6888)	34 / 36	0,4140
PCL5-X6	0,6461	(0,5113 to 0,7809)	35 / 35	0,0336
RPQ-X6	0,6097	(0,4742 to 0,7453)	37 / 33	0,1125
**Wei-Lachin**				
COMBINED	0,5219	(0,4236 to 0,6202)	370 / 353	0,6626

GOSE/-Q 7-8 vs. GOSE/-Q 3-4 - Age ≥ 65 Years

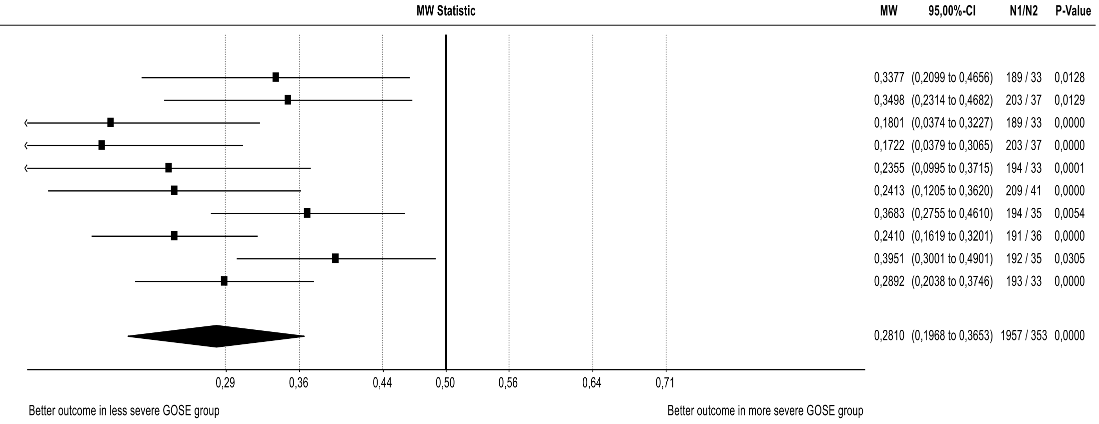

Analysis	MW	95,00%-CI	N1/N2	P-Value
**H1232-6X**				
SF36MCS-X	0,3377	(0,2099 to 0,4656)	189 / 33	0,0128
SF12MCS-X	0,3498	(0,2314 to 0,4682)	203 / 37	0,0129
SF36PCS-X	0,1801	(0,0374 to 0,3227)	189 / 33	0,0000
SF12PCS-X	0,1722	(0,0379 to 0,3065)	203 / 37	0,0000
QOLIBRI-X	0,2355	(0,0995 to 0,3715)	194 / 33	0,0001
QOLI-OS-X	0,2413	(0,1205 to 0,3620)	209 / 41	0,0000
GAD7-X6	0,3683	(0,2755 to 0,4610)	194 / 35	0,0054
PHQ9-X6	0,2410	(0,1619 to 0,3201)	191 / 36	0,0000
PCL5-X6	0,3951	(0,3001 to 0,4901)	192 / 35	0,0305
RPQ-X6	0,2892	(0,2038 to 0,3746)	193 / 33	0,0000
**Wei-Lachin**				
COMBINED	0,2810	(0,1968 to 0,3653)	1957 / 353	0,0000

12 months after TBI (completers)

GOSE/-Q 7-8 vs. GOSE/-Q 5-6 -Age < 65

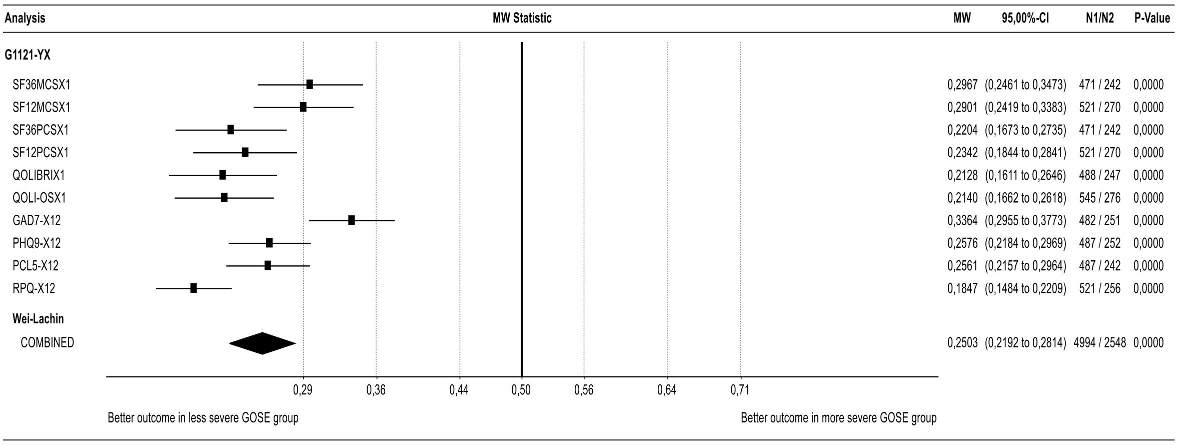

GOSE/-Q 5-6 vs. GOSE/-Q 3-4 -Age < 65

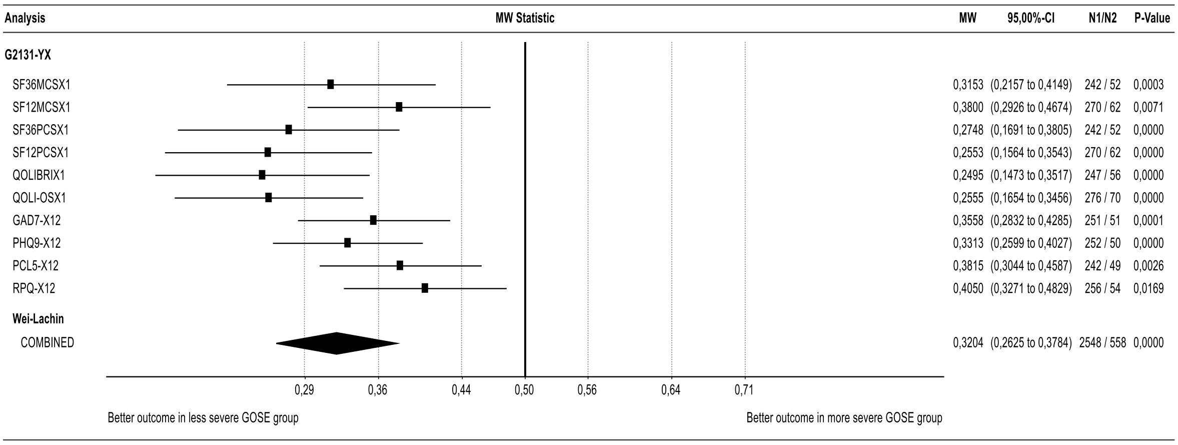

GOSE/-Q 7-8 vs. GOSE/-Q 3-4 -Age < 65

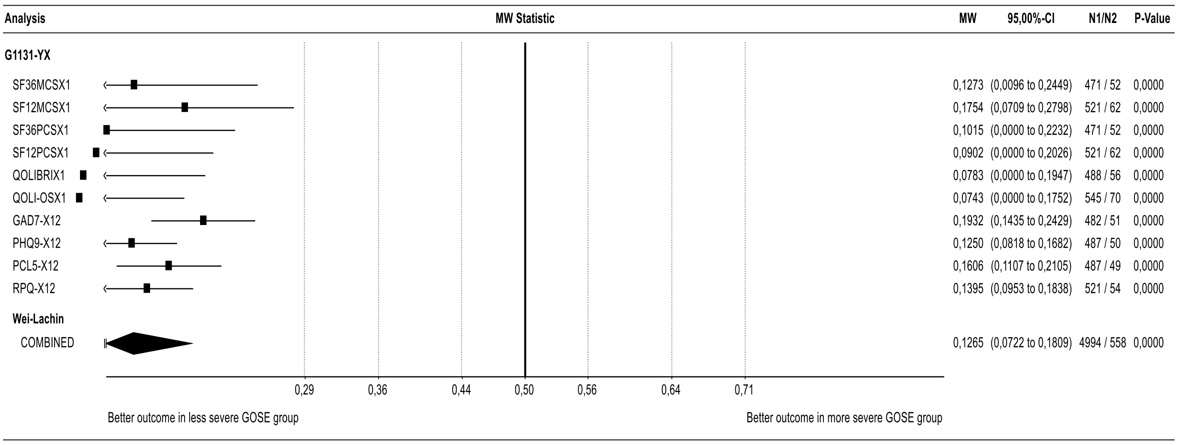

GOSE/-Q 7-8 vs. GOSE/-Q 3-4 - Age ≥ 65 Years

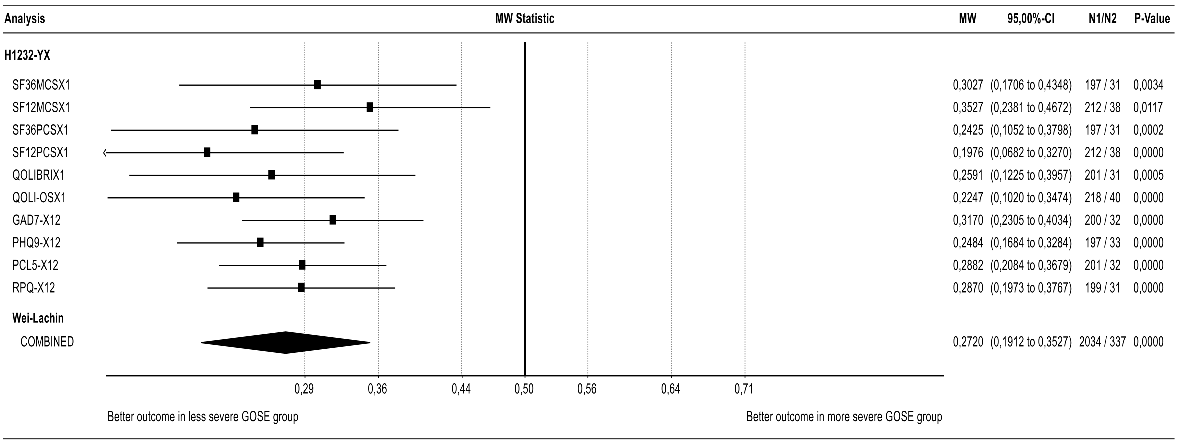

ISS

3 months after TBI (data as available)

GOSE/-Q 7-8 vs. GOSE/-Q 5-6 -ISS < 10

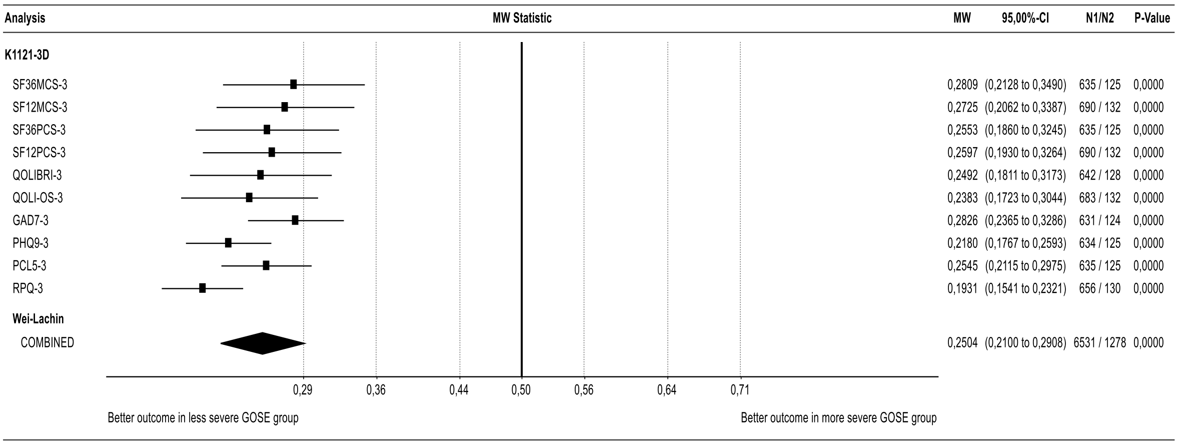

GOSE/-Q 5-6 vs. GOSE/-Q 3-4 -ISS < 10

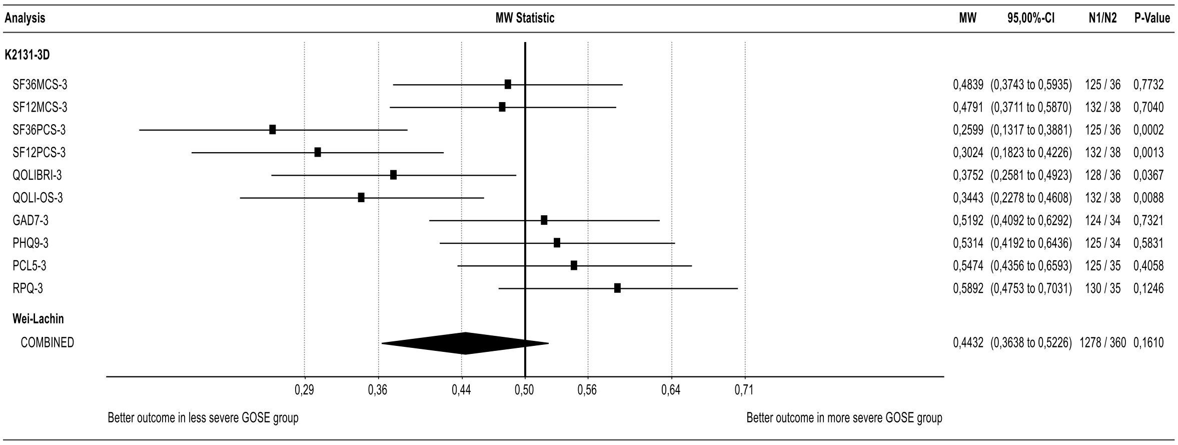

GOSE/-Q 7-8 vs. GOSE/-Q 3-4 -ISS < 10

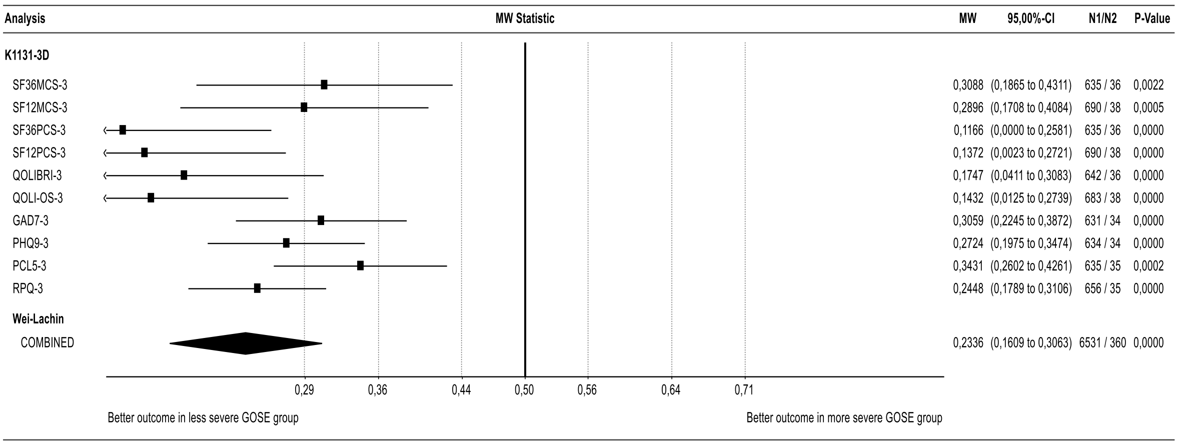

GOSE/-Q 7-8 vs. GOSE/-Q 5-6 – ISS ≥ 10

Analysis	MW	95,00%-CI	N1/N2	P-Value
**L1222-3D**				
SF36MCS-3	0,3288	(0,2892 to 0,3684)	589 / 396	0,0000
SF12MCS-3	0,3415	(0,3042 to 0,3788)	641 / 442	0,0000
SF36PCS-3	0,2674	(0,2267 to 0,3081)	589 / 396	0,0000
SF12PCS-3	0,2756	(0,2371 to 0,3141)	641 / 442	0,0000
QOLIBRI-3	0,2991	(0,2602 to 0,3380)	598 / 414	0,0000
QOLI-OS-3	0,2811	(0,2439 to 0,3183)	652 / 448	0,0000
GAD7-3	0,3323	(0,2984 to 0,3661)	586 / 415	0,0000
PHQ9-3	0,3010	(0,2665 to 0,3354)	588 / 414	0,0000
PCL5-3	0,3047	(0,2696 to 0,3398)	587 / 410	0,0000
RPQ-3	0,2539	(0,2207 to 0,2871)	606 / 427	0,0000
**Wei-Lachin**				
COMBINED	0,2985	(0,2729 to 0,3242)	6077 / 4204	0,0000

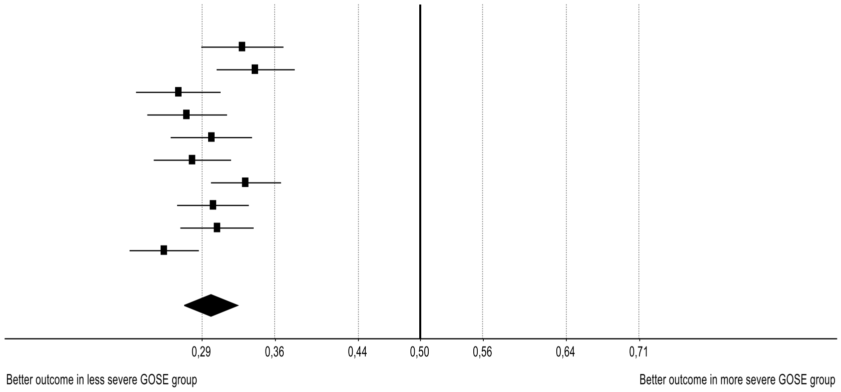

GOSE/-Q 5-6 vs. GOSE/-Q 3-4 - ISS ≥ 10

Analysis	MW	95,00%-CI	N1/N2	P-Value
**L2232-3D**				
SF36MCS-3	0,4350	(0,3892 to 0,4807)	396 / 267	0,0054
SF12MCS-3	0,4046	(0,3615 to 0,4476)	442 / 314	0,0000
SF36PCS-3	0,2807	(0,2314 to 0,3300)	396 / 267	0,0000
SF12PCS-3	0,3005	(0,2554 to 0,3456)	442 / 314	0,0000
QOLIBRI-3	0,3162	(0,2688 to 0,3636)	414 / 275	0,0000
QOLI-OS-3	0,2933	(0,2509 to 0,3356)	448 / 354	0,0000
GAD7-3	0,4479	(0,4045 to 0,4914)	415 / 273	0,0188
PHQ9-3	0,4097	(0,3667 to 0,4526)	414 / 276	0,0000
PCL5-3	0,4802	(0,4359 to 0,5244)	410 / 264	0,3792
RPQ-3	0,4255	(0,3828 to 0,4681)	427 / 279	0,0006
**Wei-Lachin**				
COMBINED	0,3793	(0,3476 to 0,4111)	4204 / 2883	0,0000

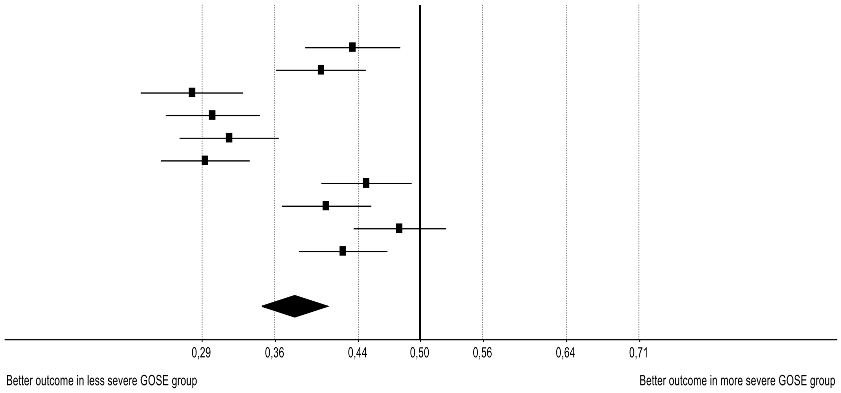

GOSE/-Q 7-8 vs. GOSE/-Q 3-4 - ISS ≥ 10

Analysis	MW	95,00%-CI	N1/N2	P-Value
**L1232-3D**				
SF36MCS-3	0,2734	(0,2245 to 0,3224)	589 / 267	0,0000
SF12MCS-3	0,2593	(0,2137 to 0,3048)	641 / 314	0,0000
SF36PCS-3	0,1266	(0,0727 to 0,1804)	589 / 267	0,0000
SF12PCS-3	0,1364	(0,0871 to 0,1858)	641 / 314	0,0000
QOLIBRI-3	0,1643	(0,1133 to 0,2153)	598 / 275	0,0000
QOLI-OS-3	0,1377	(0,0931 to 0,1824)	652 / 354	0,0000
GAD7-3	0,2929	(0,2560 to 0,3298)	586 / 273	0,0000
PHQ9-3	0,2291	(0,1928 to 0,2655)	588 / 276	0,0000
PCL5-3	0,2895	(0,2514 to 0,3275)	587 / 264	0,0000
RPQ-3	0,2008	(0,1656 to 0,2359)	606 / 279	0,0000
**Wei-Lachin**				
COMBINED	0,2110	(0,1815 to 0,2405)	6077 / 2883	0,0000

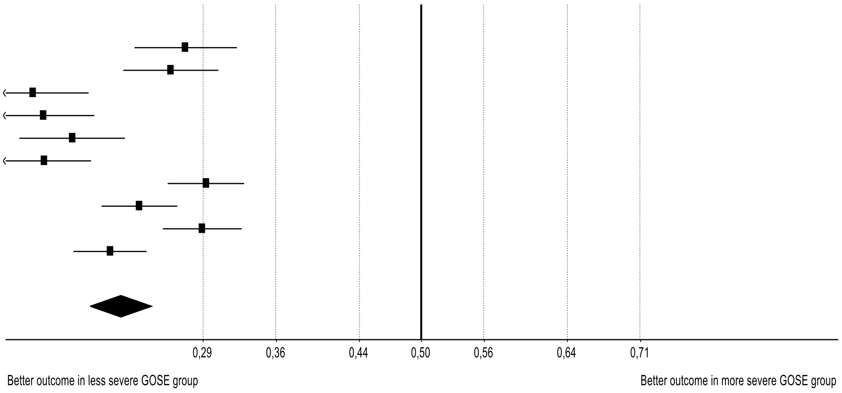

6 months after TBI (data as available)

GOSE/-Q 7-8 vs. GOSE/-Q 5-6 -ISS < 10

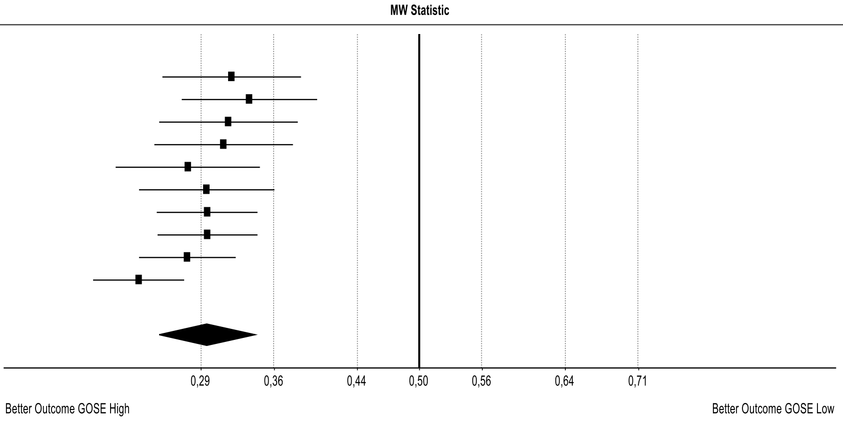

Analysis	MW	95,00%-CI	N1/N2	P-Value
**K1121-6D**				
SF36MCS-6	0,3193	(0,2526 to 0,3861)	574 / 123	0,0000
SF12MCS-6	0,3365	(0,2714 to 0,4015)	597 / 125	0,0000
SF36PCS-6	0,3162	(0,2495 to 0,3828)	572 / 123	0,0000
SF12PCS-6	0,3118	(0,2454 to 0,3782)	597 / 125	0,0000
QOLIBRI-6	0,2776	(0,2084 to 0,3467)	576 / 121	0,0000
QOLI-OS-6	0,2955	(0,2304 to 0,3606)	593 / 127	0,0000
GAD7-6	0,2960	(0,2475 to 0,3445)	567 / 121	0,0000
PHQ9-6	0,2965	(0,2484 to 0,3446)	568 / 122	0,0000
PCL5-6	0,2769	(0,2307 to 0,3231)	576 / 121	0,0000
RPQ-6	0,2302	(0,1865 to 0,2738)	587 / 126	0,0000
**Wei-Lachin**				
COMBINED	0,2956	(0,2496 to 0,3417)	5807 / 1234	0,0000

GOSE/-Q 5-6 vs. GOSE/-Q 3-4 -ISS < 10

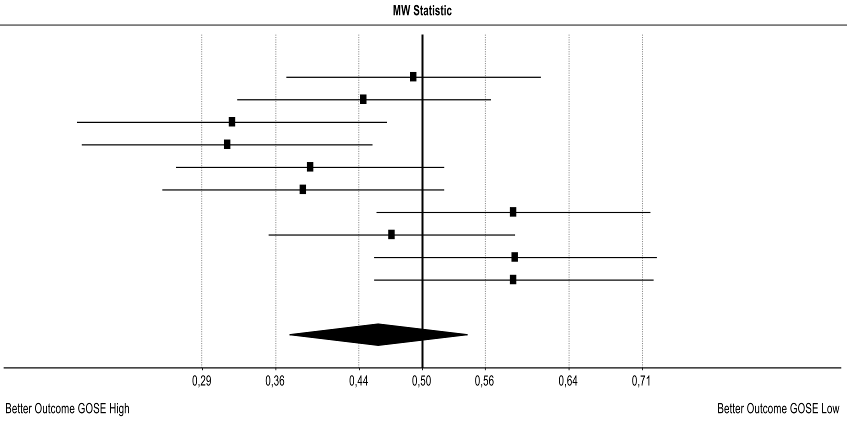

Analysis	MW	95,00%-CI	N1/N2	P-Value
**K2131-6D**				
SF36MCS-6	0,4919	(0,3706 to 0,6132)	123 / 24	0,8955
SF12MCS-6	0,4443	(0,3231 to 0,5655)	125 / 27	0,3678
SF36PCS-6	0,3188	(0,1707 to 0,4668)	123 / 24	0,0164
SF12PCS-6	0,3141	(0,1752 to 0,4529)	125 / 27	0,0087
QOLIBRI-6	0,3934	(0,2653 to 0,5215)	121 / 25	0,1029
QOLI-OS-6	0,3865	(0,2517 to 0,5212)	127 / 25	0,0987
GAD7-6	0,5873	(0,4562 to 0,7184)	121 / 24	0,1919
PHQ9-6	0,4713	(0,3537 to 0,5890)	122 / 24	0,6327
PCL5-6	0,5890	(0,4541 to 0,7240)	121 / 22	0,1960
RPQ-6	0,5875	(0,4542 to 0,7208)	126 / 24	0,1984
**Wei-Lachin**				
COMBINED	0,4584	(0,3736 to 0,5432)	1234 / 246	0,3361

GOSE/-Q 7-8 vs. GOSE/-Q 5-6 - ISS ≥ 10

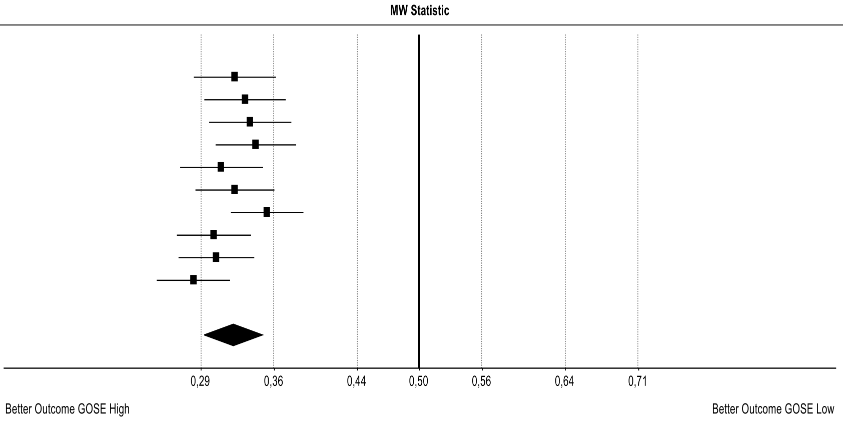

Analysis	MW	95,00%-CI	N1/N2	P-Value
**L1222-6D**				
SF36MCS-6	0,3226	(0,2829 to 0,3623)	527 / 403	0,0000
SF12MCS-6	0,3325	(0,2936 to 0,3714)	558 / 413	0,0000
SF36PCS-6	0,3372	(0,2977 to 0,3766)	527 / 403	0,0000
SF12PCS-6	0,3425	(0,3038 to 0,3813)	558 / 413	0,0000
QOLIBRI-6	0,3098	(0,2701 to 0,3494)	540 / 400	0,0000
QOLI-OS-6	0,3227	(0,2846 to 0,3609)	567 / 418	0,0000
GAD7-6	0,3536	(0,3184 to 0,3887)	536 / 402	0,0000
PHQ9-6	0,3022	(0,2666 to 0,3379)	535 / 404	0,0000
PCL5-6	0,3047	(0,2684 to 0,3410)	537 / 400	0,0000
RPQ-6	0,2828	(0,2479 to 0,3178)	553 / 413	0,0000
**Wei-Lachin**				
COMBINED	0,3211	(0,2933 to 0,3488)	5438 / 4069	0,0000

GOSE/-Q 5-6 vs. GOSE/-Q 3-4 - ISS ≥ 10

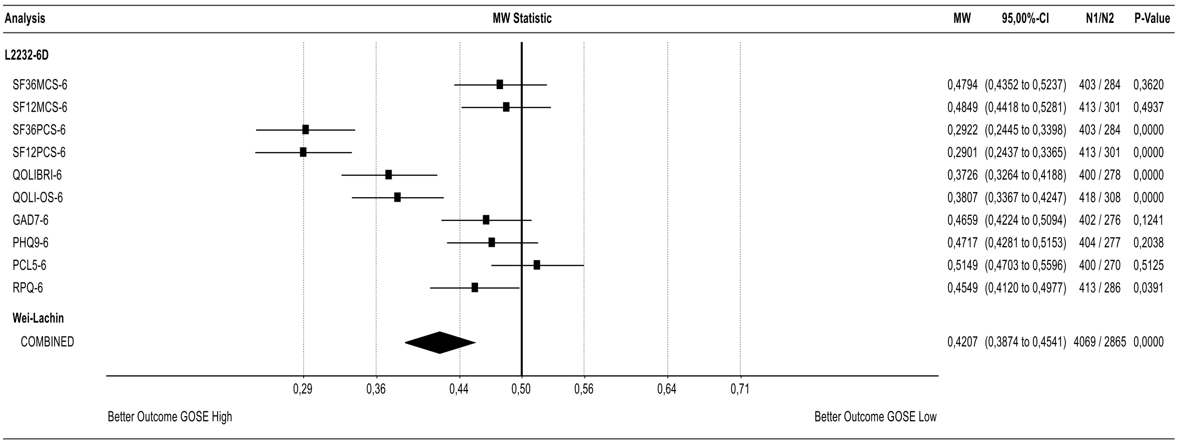

GOSE/-Q 7-8 vs. GOSE/-Q 3-4 - ISS ≥ 10

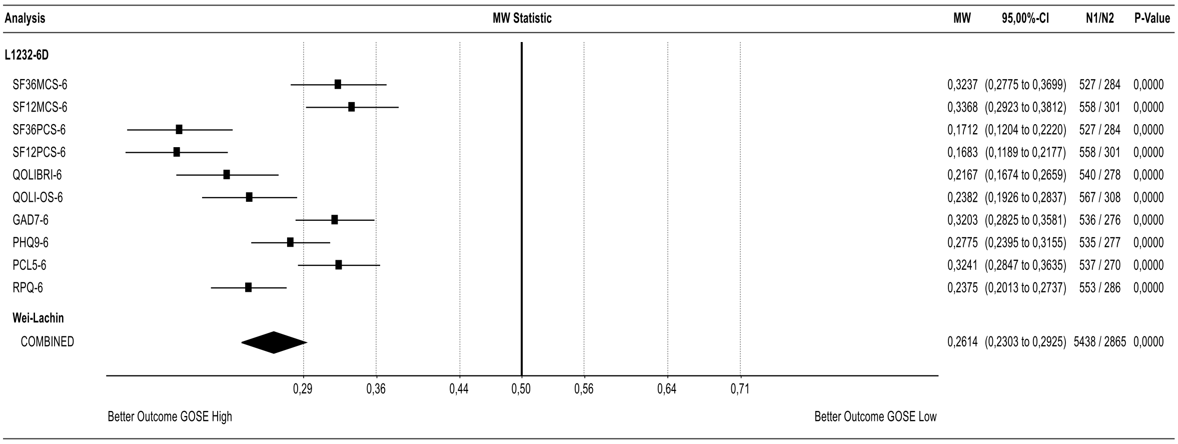

<u>12 months after TBI (data as available)</u>

GOSE/-Q 7-8 vs. GOSE/-Q 5-6 -ISS < 10

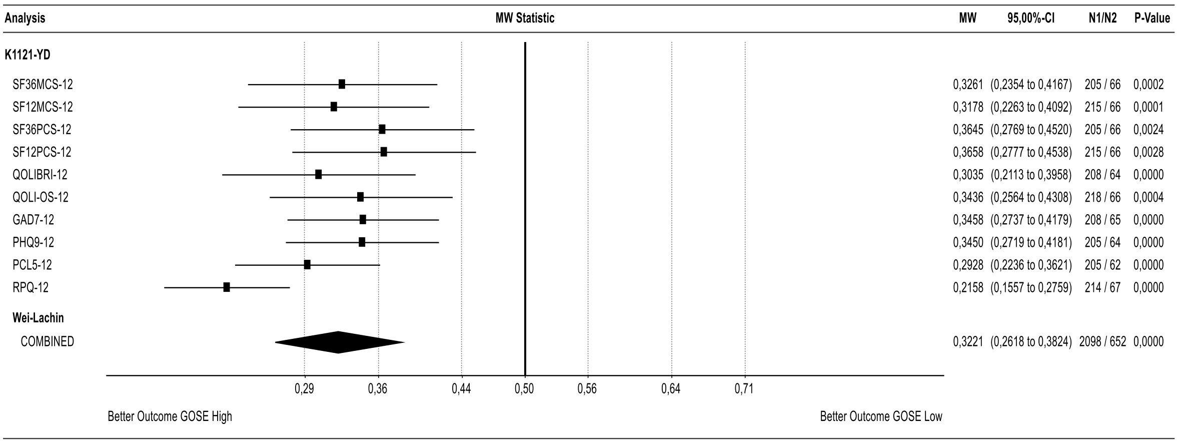

GOSE/-Q 5-6 vs. GOSE/-Q 3-4 -ISS < 10

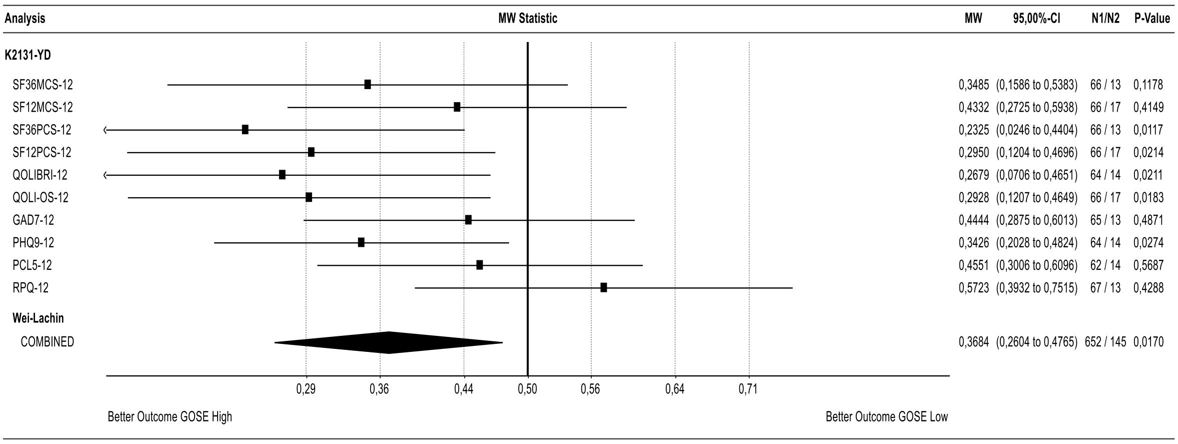

GOSE/-Q 7-8 vs. GOSE/-Q 5-6 - ISS ≥ 10

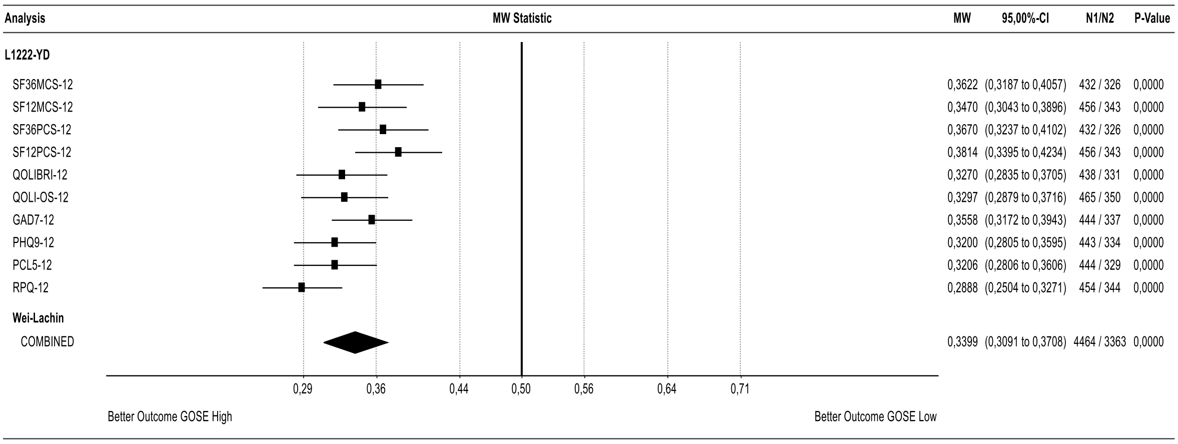

GOSE/-Q 5-6 vs. GOSE/-Q 3-4 - ISS ≥ 10

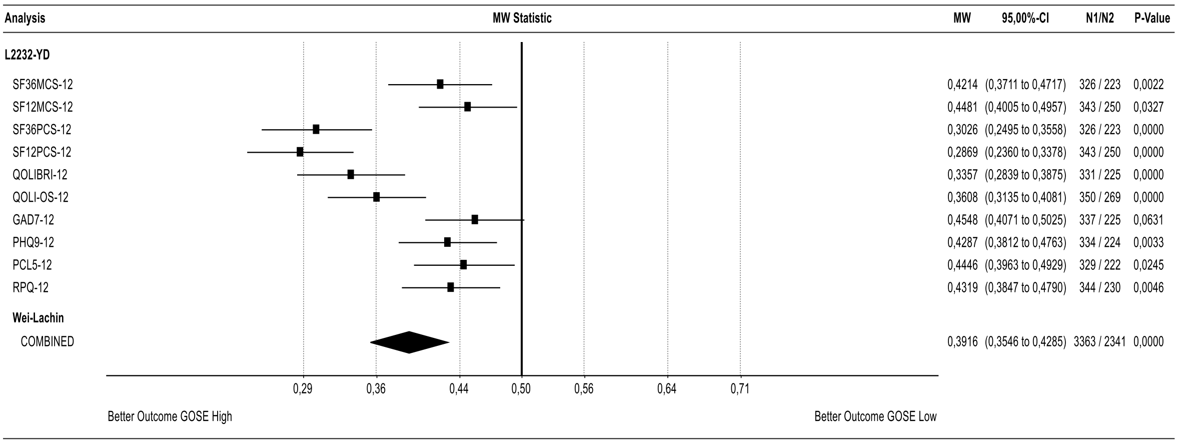

GOSE/-Q 7-8 vs. GOSE/-Q 3-4 - ISS ≥ 10

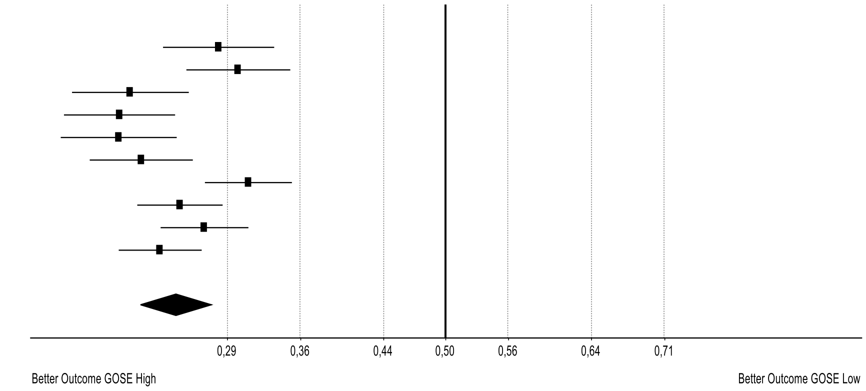

Analysis	MW	95,00%-CI	N1/N2	P-Value
**L1232-YD**				
SF36MCS-12	0,2818	(0,2285 to 0,3350)	432 / 223	0,0000
SF12MCS-12	0,3004	(0,2506 to 0,3502)	456 / 250	0,0000
SF36PCS-12	0,1965	(0,1404 to 0,2526)	432 / 223	0,0000
SF12PCS-12	0,1867	(0,1333 to 0,2401)	456 / 250	0,0000
QOLIBRI-12	0,1854	(0,1297 to 0,2410)	438 / 225	0,0000
QOLI-OS-12	0,2073	(0,1579 to 0,2566)	465 / 269	0,0000
GAD7-12	0,3101	(0,2681 to 0,3521)	444 / 225	0,0000
PHQ9-12	0,2441	(0,2031 to 0,2852)	443 / 224	0,0000
PCL5-12	0,2680	(0,2255 to 0,3106)	444 / 222	0,0000
RPQ-12	0,2251	(0,1852 to 0,2651)	454 / 230	0,0000
**Wei-Lachin**				
COMBINED	0,2405	(0,2066 to 0,2744)	4464 / 2341	0,0000

<u>3 months after TBI (completers)</u>

GOSE/-Q 7-8 vs. GOSE/-Q 5-6 -ISS < 10

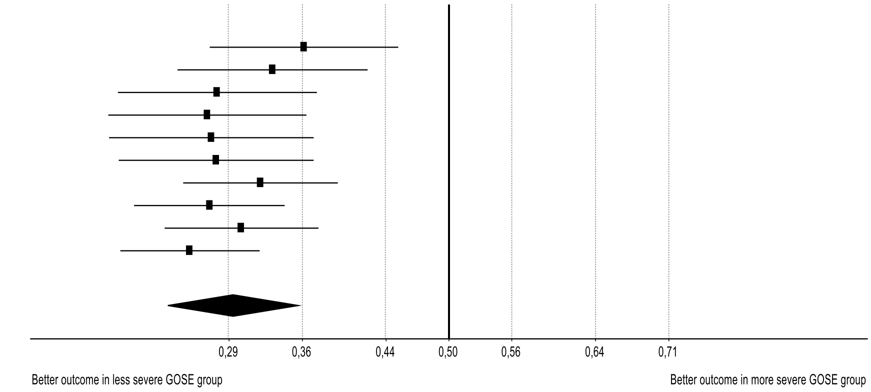

Analysis	MW	95,00%-CI	N1/N2	P-Value
**K1121-3X**				
SF36MCS-X	0,3619	(0,2718 to 0,4521)	170 / 65	0,0027
SF12MCS-X	0,3319	(0,2408 to 0,4229)	184 / 65	0,0003
SF36PCS-X	0,2792	(0,1840 to 0,3743)	170 / 65	0,0000
SF12PCS-X	0,2696	(0,1750 to 0,3643)	184 / 65	0,0000
QOLIBRI-X	0,2733	(0,1756 to 0,3709)	176 / 62	0,0000
QOLI-OS-X	0,2782	(0,1851 to 0,3713)	189 / 65	0,0000
GAD7-X3	0,3203	(0,2462 to 0,3944)	173 / 63	0,0000
PHQ9-X3	0,2717	(0,1999 to 0,3435)	173 / 63	0,0000
PCL5-X3	0,3021	(0,2288 to 0,3754)	169 / 61	0,0000
RPQ-X3	0,2530	(0,1863 to 0,3198)	182 / 66	0,0000
**Wei-Lachin**				
COMBINED	0,2941	(0,2320 to 0,3563)	1770 / 640	0,0000

GOSE/-Q 7-8 vs. GOSE/-Q 5-6 – ISS ≥ 10

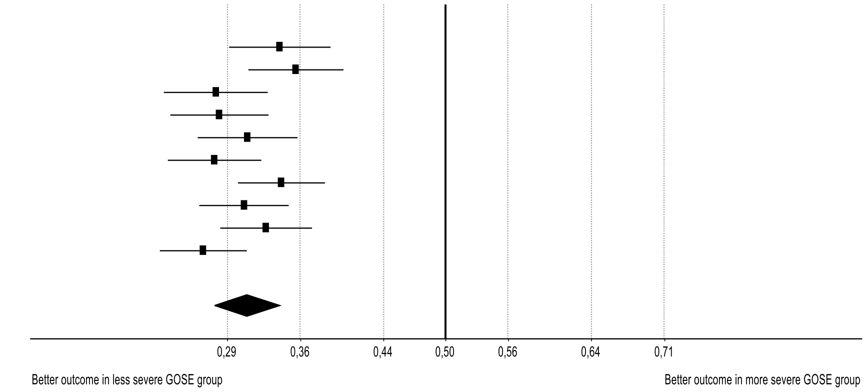

Analysis	MW	95,00%-CI	N1/N2	P-Value
**L1222-3X**				
SF36MCS-X	0,3405	(0,2917 to 0,3892)	356 / 262	0,0000
SF12MCS-X	0,3558	(0,3100 to 0,4016)	396 / 294	0,0000
SF36PCS-X	0,2791	(0,2291 to 0,3291)	356 / 262	0,0000
SF12PCS-X	0,2821	(0,2348 to 0,3293)	396 / 294	0,0000
QOLIBRI-X	0,3096	(0,2618 to 0,3575)	365 / 275	0,0000
QOLI-OS-X	0,2776	(0,2325 to 0,3228)	408 / 306	0,0000
GAD7-X3	0,3421	(0,3000 to 0,3842)	365 / 281	0,0000
PHQ9-X3	0,3062	(0,2630 to 0,3493)	362 / 282	0,0000
PCL5-X3	0,3274	(0,2834 to 0,3714)	371 / 274	0,0000
RPQ-X3	0,2671	(0,2254 to 0,3088)	382 / 294	0,0000
**Wei-Lachin**				
COMBINED	0,3088	(0,2774 to 0,3401)	3757 / 2824	0,0000

GOSE/-Q 5-6 vs. GOSE/-Q 3-4 - ISS ≥ 10

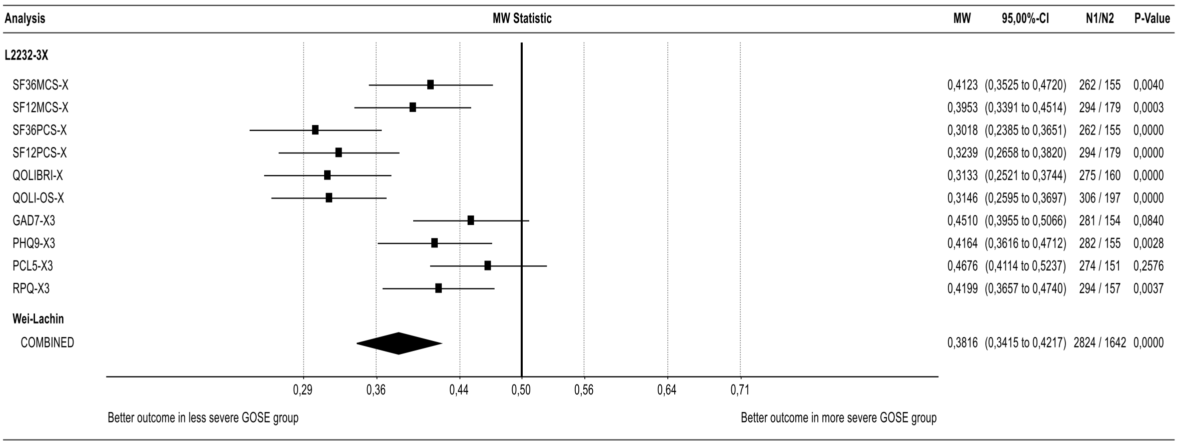

GOSE/-Q 7-8 vs. GOSE/-Q 3-4 - ISS ≥ 10

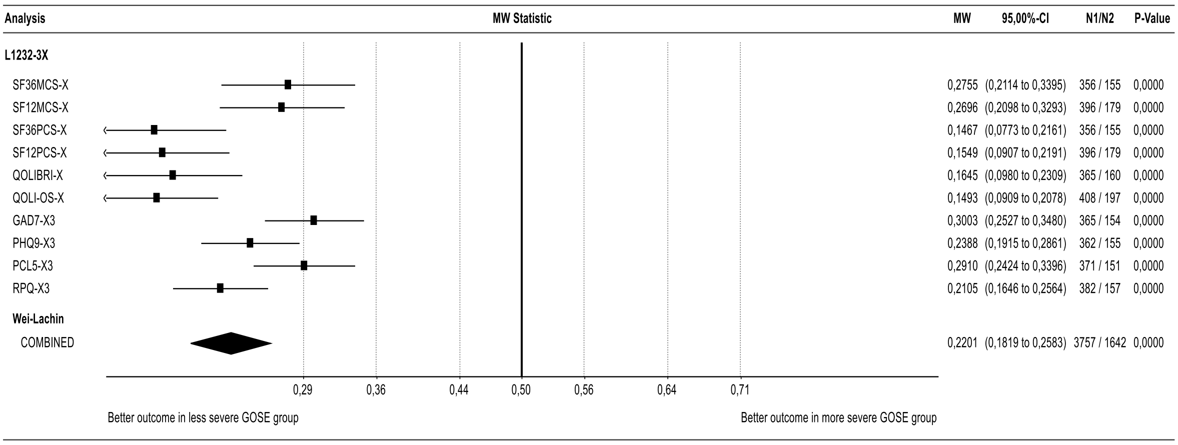

<u>6 months after TBI (completers)</u>

GOSE/-Q 7-8 vs. GOSE/-Q 5-6 -ISS < 10

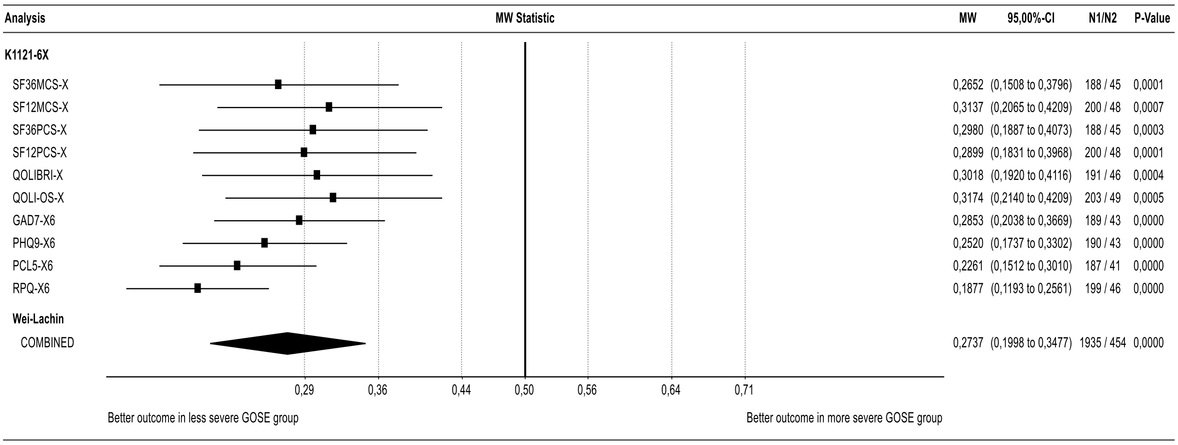

GOSE/-Q 7-8 vs. GOSE/-Q 5-6 - ISS ≥ 10

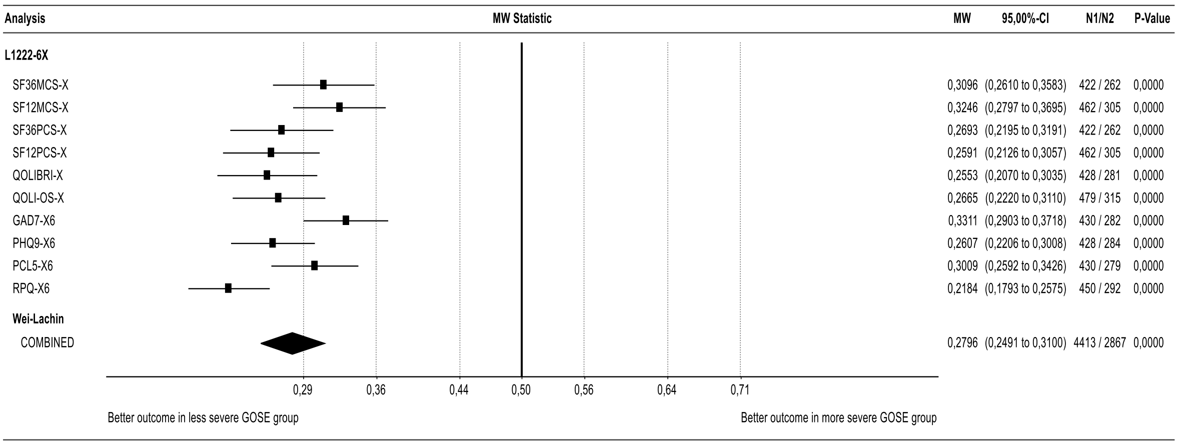

GOSE/-Q 5-6 vs. GOSE/-Q 3-4 - ISS ≥ 10

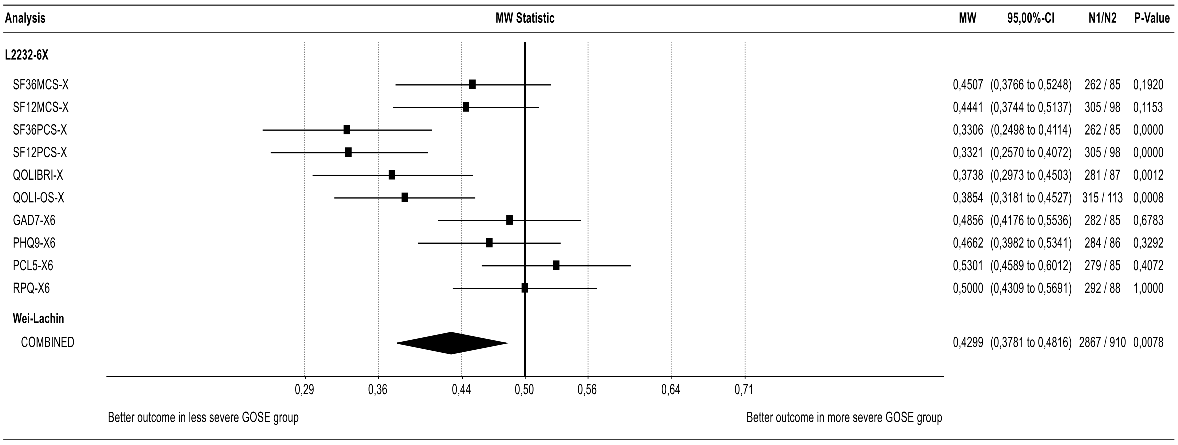

GOSE/-Q 7-8 vs. GOSE/-Q 3-4 - ISS ≥ 10

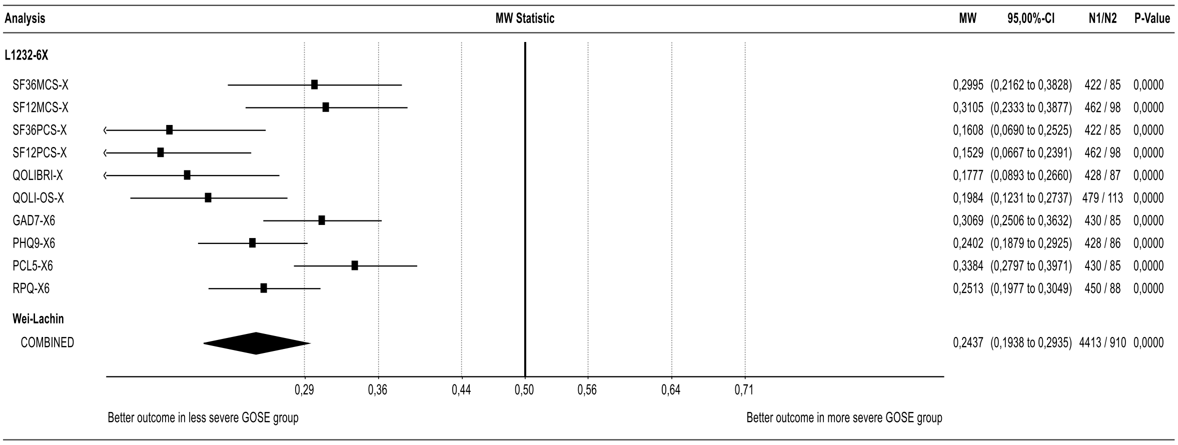

12 months after TBI (completers)

GOSE/-Q 7-8 vs. GOSE/-Q 5-6 -ISS < 10

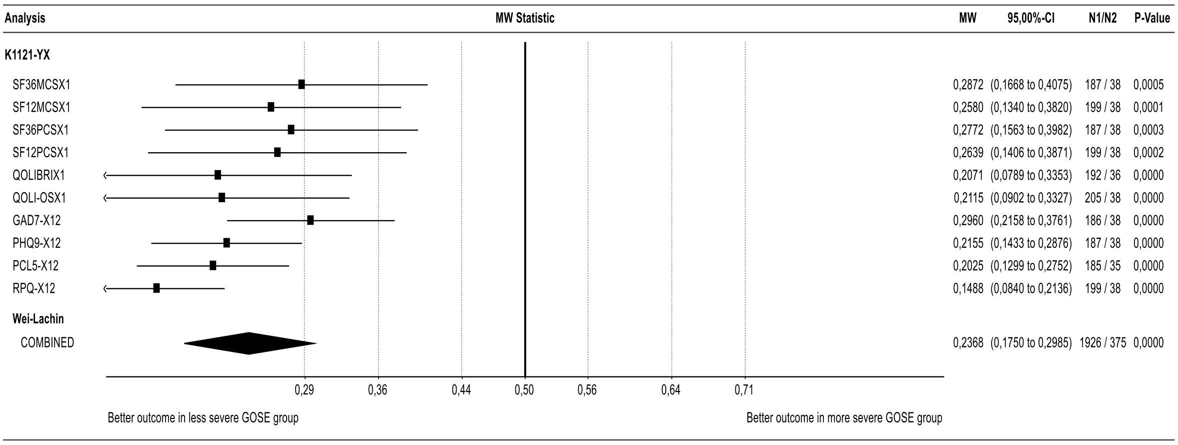

GOSE/-Q 7-8 vs. GOSE/-Q 5-6 - ISS ≥ 10

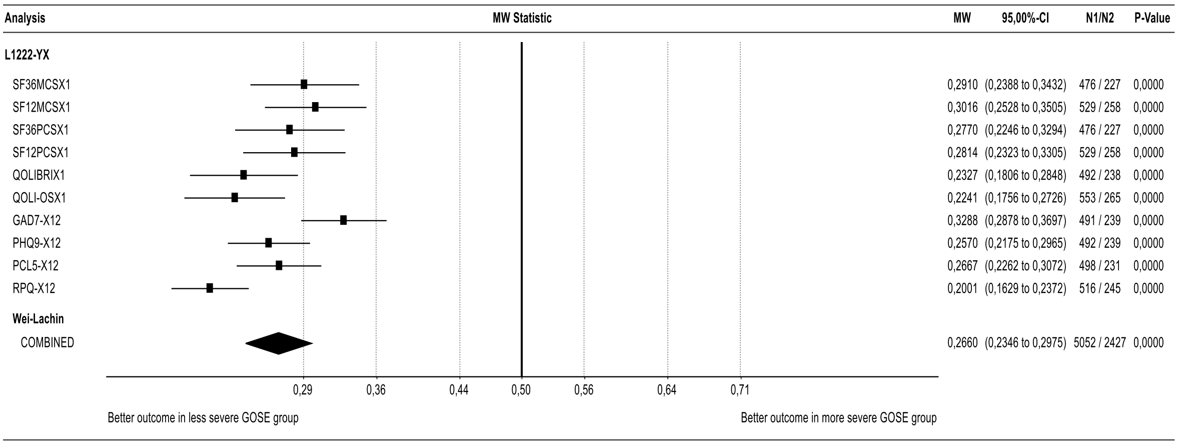

GOSE/-Q 5-6 vs. GOSE/-Q 3-4 - ISS ≥ 10

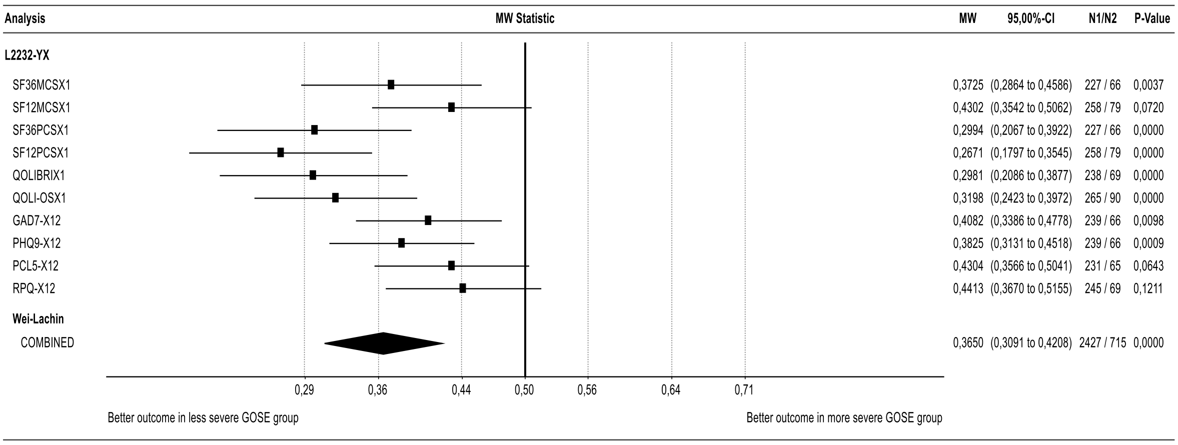

GOSE/-Q 7-8 vs. GOSE/-Q 3-4 - ISS ≥ 10

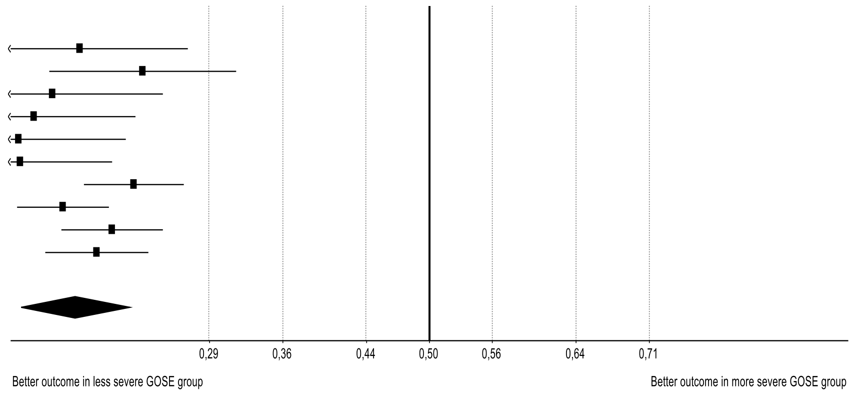

Analysis	MW	95,00%-CI	N1/N2	P-Value
**L1232-YX**				
SF36MCSX1	0,1669	(0,0645 to 0,2693)	476 / 66	0,0000
SF12MCSX1	0,2269	(0,1376 to 0,3161)	529 / 79	0,0000
SF36PCSX1	0,1407	(0,0360 to 0,2454)	476 / 66	0,0000
SF12PCSX1	0,1225	(0,0253 to 0,2198)	529 / 79	0,0000
QOLIBRIX1	0,1077	(0,0048 to 0,2106)	492 / 69	0,0000
QOLI-OSX1	0,1097	(0,0219 to 0,1975)	553 / 90	0,0000
GAD7-X12	0,2181	(0,1702 to 0,2660)	491 / 66	0,0000
PHQ9-X12	0,1502	(0,1062 to 0,1942)	492 / 66	0,0000
PCL5-X12	0,1974	(0,1488 to 0,2459)	498 / 65	0,0000
RPQ-X12	0,1829	(0,1338 to 0,2320)	516 / 69	0,0000
**Wei-Lachin**				
COMBINED	0,1623	(0,1102 to 0,2144)	5052 / 715	0,0000

Education

3 months after TBI (data as available)

GOSE/-Q 7-8 vs. GOSE/-Q 5-6 -Edu ≤ Primary

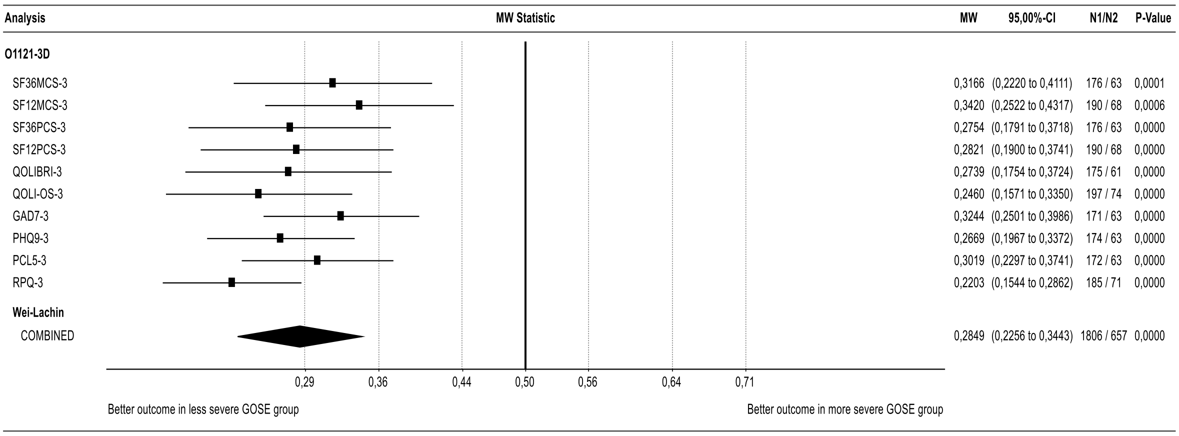

GOSE/-Q 5-6 vs. GOSE/-Q 3-4 -Edu ≤ Primary

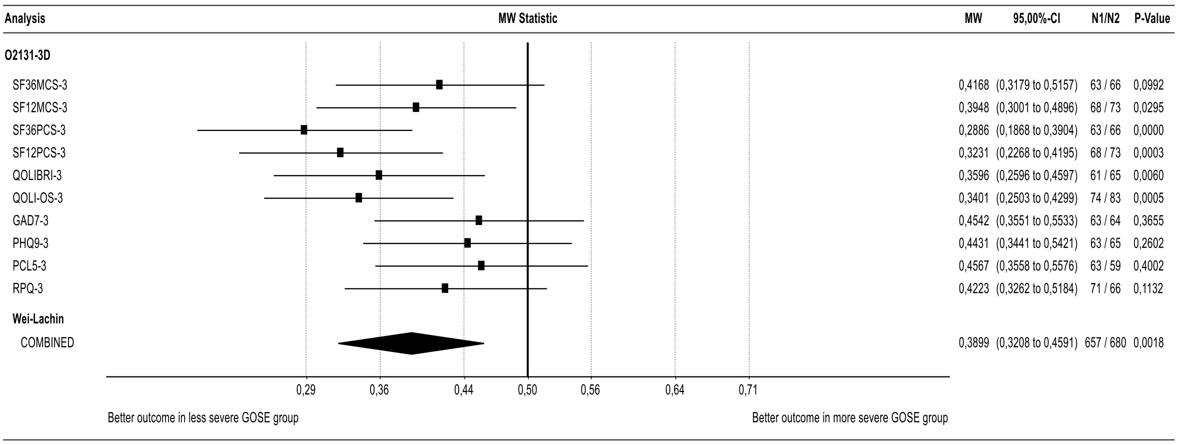

GOSE/-Q 7-8 vs. GOSE/-Q 3-4 -Edu ≤ Primary

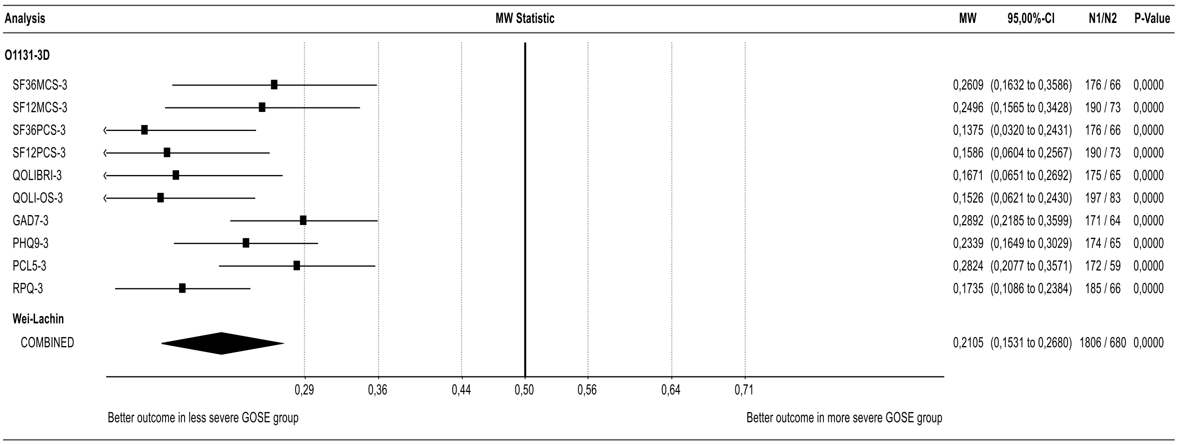

GOSE/-Q 7-8 vs. GOSE/-Q 5-6 – Edu > Primary

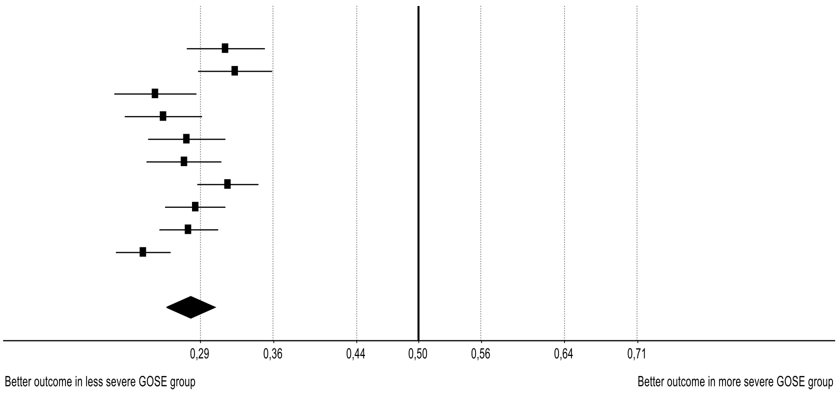

Analysis	MW	95,00%-CI	N1/N2	P-Value
**P1222-3D**				
SF36MCS-3	0,3144	(0,2766 to 0,3521)	952 / 417	0,0000
SF12MCS-3	0,3238	(0,2881 to 0,3594)	1024 / 461	0,0000
SF36PCS-3	0,2469	(0,2075 to 0,2862)	952 / 417	0,0000
SF12PCS-3	0,2542	(0,2170 to 0,2915)	1024 / 461	0,0000
QOLIBRI-3	0,2771	(0,2399 to 0,3143)	966 / 438	0,0000
QOLI-OS-3	0,2743	(0,2385 to 0,3101)	1019 / 460	0,0000
GAD7-3	0,3164	(0,2870 to 0,3458)	951 / 434	0,0000
PHQ9-3	0,2851	(0,2558 to 0,3144)	951 / 434	0,0000
PCL5-3	0,2787	(0,2503 to 0,3071)	952 / 431	0,0000
RPQ-3	0,2352	(0,2086 to 0,2618)	974 / 440	0,0000
**Wei-Lachin**				
COMBINED	0,2806	(0,2575 to 0,3037)	9765 / 4393	0,0000

GOSE/-Q 5-6 vs. GOSE/-Q 3-4 - Edu > Primary

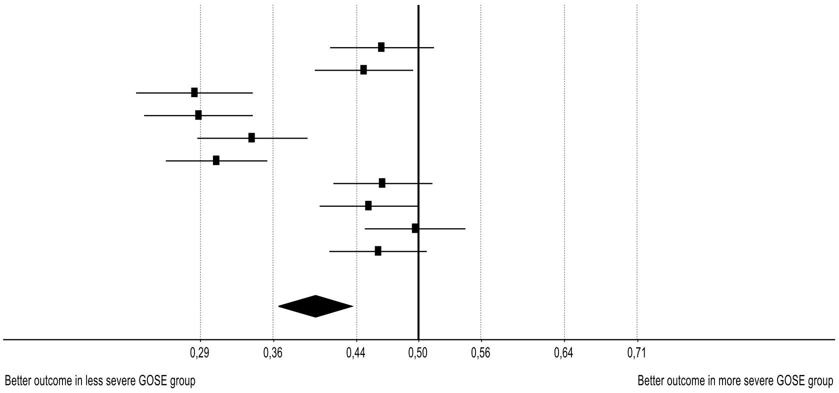

Analysis	MW	95,00%-CI	N1/N2	P-Value
**P2232-3D**				
SF36MCS-3	0,4647	(0,4147 to 0,5148)	417 / 195	0,1672
SF12MCS-3	0,4477	(0,4004 to 0,4949)	461 / 227	0,0300
SF36PCS-3	0,2844	(0,2281 to 0,3407)	417 / 195	0,0000
SF12PCS-3	0,2884	(0,2363 to 0,3405)	461 / 227	0,0000
QOLIBRI-3	0,3400	(0,2870 to 0,3930)	438 / 202	0,0000
QOLI-OS-3	0,3056	(0,2571 to 0,3541)	460 / 253	0,0000
GAD7-3	0,4655	(0,4179 to 0,5132)	434 / 198	0,1566
PHQ9-3	0,4523	(0,4051 to 0,4995)	434 / 200	0,0478
PCL5-3	0,4967	(0,4483 to 0,5451)	431 / 196	0,8943
RPQ-3	0,4611	(0,4144 to 0,5078)	440 / 205	0,1027
**Wei-Lachin**				
COMBINED	0,4007	(0,3654 to 0,4359)	4393 / 2098	0,0000

GOSE/-Q 7-8 vs. GOSE/-Q 3-4 - Edu > Primary

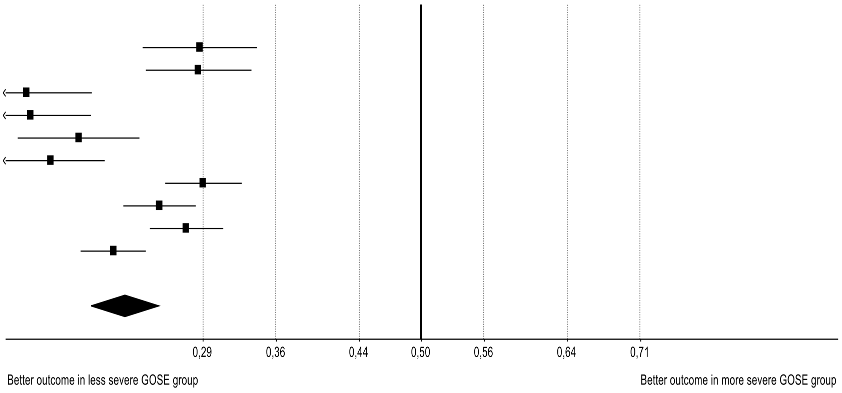

Analysis	MW	95,00%-CI	N1/N2	P-Value
**P1232-3D**				
SF36MCS-3	0,2872	(0,2321 to 0,3424)	952 / 195	0,0000
SF12MCS-3	0,2857	(0,2348 to 0,3367)	1024 / 227	0,0000
SF36PCS-3	0,1207	(0,0580 to 0,1834)	952 / 195	0,0000
SF12PCS-3	0,1247	(0,0668 to 0,1826)	1024 / 227	0,0000
QOLIBRI-3	0,1706	(0,1123 to 0,2290)	966 / 202	0,0000
QOLI-OS-3	0,1434	(0,0913 to 0,1955)	1019 / 253	0,0000
GAD7-3	0,2904	(0,2537 to 0,3271)	951 / 198	0,0000
PHQ9-3	0,2486	(0,2138 to 0,2834)	951 / 200	0,0000
PCL5-3	0,2742	(0,2387 to 0,3096)	952 / 196	0,0000
RPQ-3	0,2039	(0,1724 to 0,2355)	974 / 205	0,0000
**Wei-Lachin**				
COMBINED	0,2150	(0,1827 to 0,2472)	9765 / 2098	0,0000

6 months after TBI (data as available)

GOSE/-Q 7-8 vs. GOSE/-Q 5-6 -Edu ≤ Primary

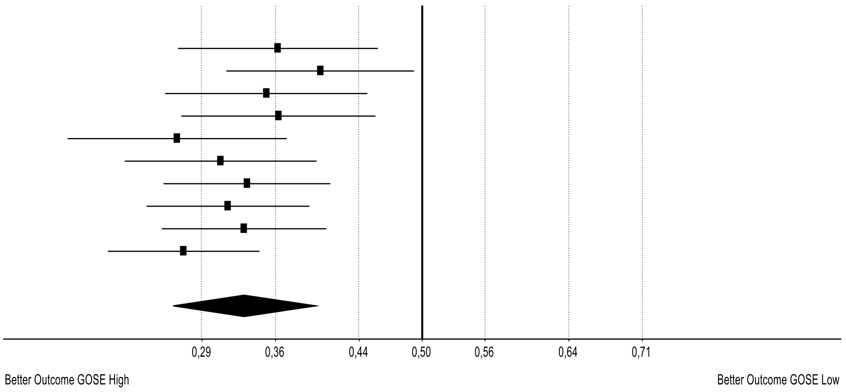

Analysis	MW	95,00%-CI	N1/N2	P-Value
**O1121-6D**				
SF36MCS-6	0,3627	(0,2674 to 0,4581)	154 / 58	0,0048
SF12MCS-6	0,4033	(0,3137 to 0,4930)	162 / 62	0,0345
SF36PCS-6	0,3518	(0,2553 to 0,4482)	154 / 58	0,0026
SF12PCS-6	0,3632	(0,2705 to 0,4558)	162 / 62	0,0038
QOLIBRI-6	0,2667	(0,1622 to 0,3711)	155 / 55	0,0000
QOLI-OS-6	0,3078	(0,2163 to 0,3994)	169 / 66	0,0000
GAD7-6	0,3331	(0,2534 to 0,4128)	156 / 54	0,0000
PHQ9-6	0,3148	(0,2370 to 0,3925)	157 / 56	0,0000
PCL5-6	0,3303	(0,2517 to 0,4089)	156 / 59	0,0000
RPQ-6	0,2728	(0,2006 to 0,3450)	167 / 66	0,0000
**Wei-Lachin**				
COMBINED	0,3306	(0,2627 to 0,3986)	1592 / 596	0,0000

GOSE/-Q 5-6 vs. GOSE/-Q 3-4 -Edu ≤ Primary

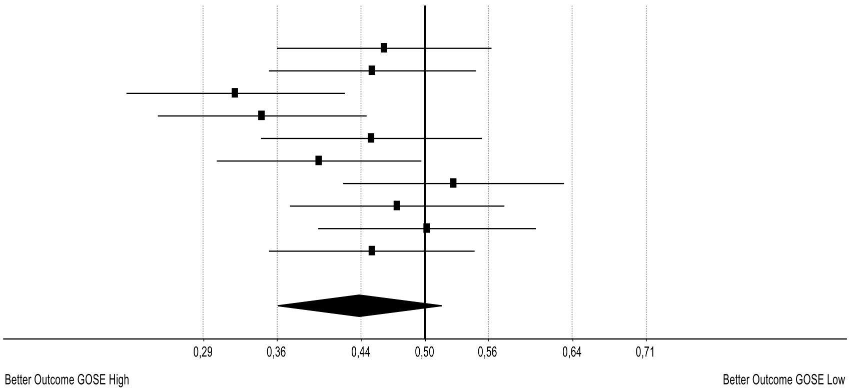

Analysis	MW	95,00%-CI	N1/N2	P-Value
**O2131-6D**				
SF36MCS-6	0,4621	(0,3604 to 0,5638)	58 / 63	0,4649
SF12MCS-6	0,4507	(0,3525 to 0,5488)	62 / 68	0,3245
SF36PCS-6	0,3207	(0,2175 to 0,4240)	58 / 63	0,0007
SF12PCS-6	0,3459	(0,2468 to 0,4451)	62 / 68	0,0023
QOLIBRI-6	0,4495	(0,3452 to 0,5539)	55 / 60	0,3435
QOLI-OS-6	0,3999	(0,3032 to 0,4966)	66 / 69	0,0424
GAD7-6	0,5276	(0,4229 to 0,6324)	54 / 61	0,6053
PHQ9-6	0,4742	(0,3725 to 0,5759)	56 / 61	0,6196
PCL5-6	0,5026	(0,3995 to 0,6057)	59 / 58	0,9601
RPQ-6	0,4500	(0,3524 to 0,5477)	66 / 64	0,3162
**Wei-Lachin**				
COMBINED	0,4383	(0,3609 to 0,5158)	596 / 635	0,1188

GOSE/-Q 7-8 vs. GOSE/-Q 3-4 -Edu ≤ Primary

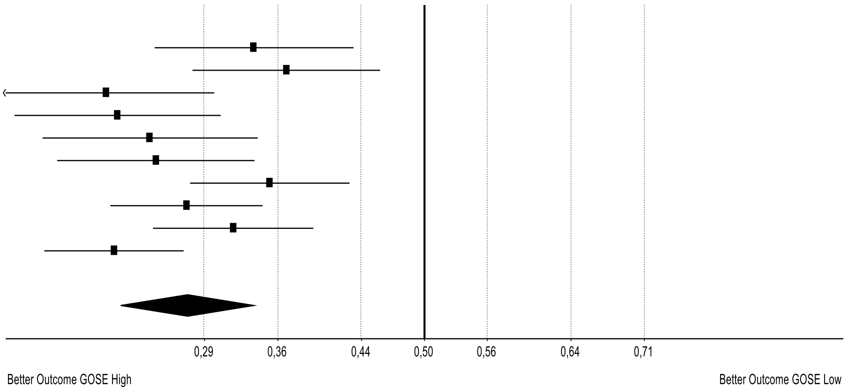

Analysis	MW	95,00%-CI	N1/N2	P-Value
**O1131-6D**				
SF36MCS-6	0,3375	(0,2426 to 0,4324)	154 / 63	0,0008
SF12MCS-6	0,3685	(0,2789 to 0,4580)	162 / 68	0,0040
SF36PCS-6	0,1962	(0,0930 to 0,2995)	154 / 63	0,0000
SF12PCS-6	0,2073	(0,1091 to 0,3056)	162 / 68	0,0000
QOLIBRI-6	0,2384	(0,1359 to 0,3409)	155 / 60	0,0000
QOLI-OS-6	0,2440	(0,1500 to 0,3380)	169 / 69	0,0000
GAD7-6	0,3527	(0,2762 to 0,4292)	156 / 61	0,0002
PHQ9-6	0,2731	(0,2003 to 0,3458)	157 / 61	0,0000
PCL5-6	0,3179	(0,2415 to 0,3943)	156 / 58	0,0000
RPQ-6	0,2039	(0,1373 to 0,2706)	167 / 64	0,0000
**Wei-Lachin**				
COMBINED	0,2739	(0,2106 to 0,3373)	1592 / 635	0,0000

GOSE/-Q 7-8 vs. GOSE/-Q 5-6 - Edu > Primary

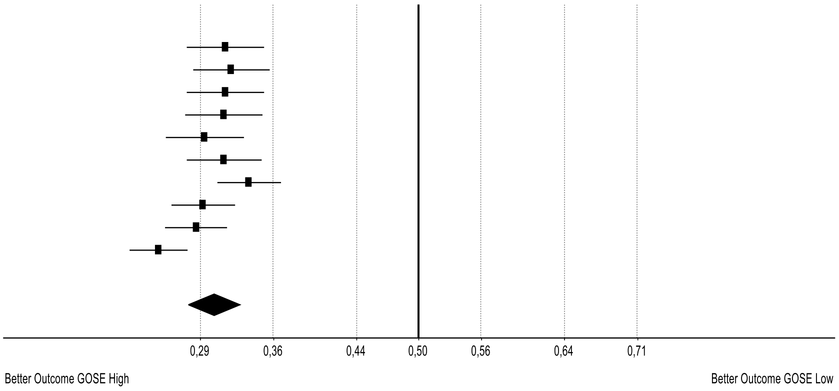

Analysis	MW	95,00%-CI	N1/N2	P-Value
**P1222-6D**				
SF36MCS-6	0,3140	(0,2766 to 0,3514)	853 / 428	0,0000
SF12MCS-6	0,3199	(0,2831 to 0,3567)	892 / 436	0,0000
SF36PCS-6	0,3140	(0,2766 to 0,3514)	851 / 428	0,0000
SF12PCS-6	0,3126	(0,2756 to 0,3497)	892 / 436	0,0000
QOLIBRI-6	0,2941	(0,2566 to 0,3317)	868 / 425	0,0000
QOLI-OS-6	0,3128	(0,2768 to 0,3489)	886 / 437	0,0000
GAD7-6	0,3369	(0,3061 to 0,3677)	856 / 429	0,0000
PHQ9-6	0,2926	(0,2620 to 0,3231)	854 / 431	0,0000
PCL5-6	0,2859	(0,2562 to 0,3157)	863 / 423	0,0000
RPQ-6	0,2496	(0,2216 to 0,2777)	875 / 431	0,0000
**Wei-Lachin**				
COMBINED	0,3032	(0,2784 to 0,3281)	8690 / 4304	0,0000

GOSE/-Q 5-6 vs. GOSE/-Q 3-4 - Edu > Primary

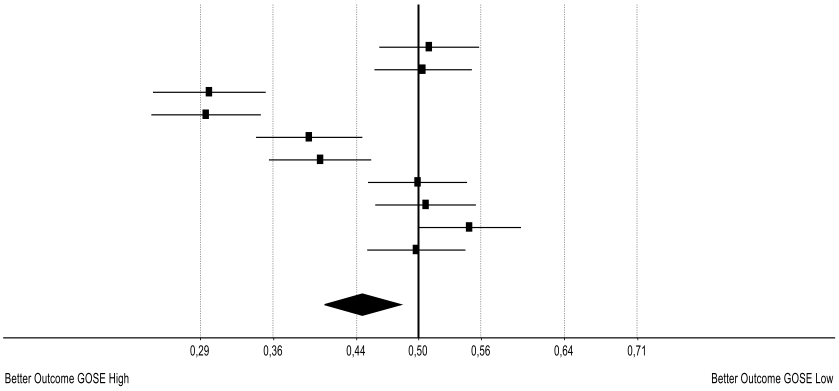

Analysis	MW	95,00%-CI	N1/N2	P-Value
**P2232-6D**				
SF36MCS-6	0,5100	(0,4623 to 0,5577)	428 / 208	0,6818
SF12MCS-6	0,5041	(0,4574 to 0,5509)	436 / 220	0,8624
SF36PCS-6	0,2983	(0,2442 to 0,3525)	428 / 208	0,0000
SF12PCS-6	0,2955	(0,2428 to 0,3483)	436 / 220	0,0000
QOLIBRI-6	0,3944	(0,3432 to 0,4456)	425 / 204	0,0001
QOLI-OS-6	0,4053	(0,3562 to 0,4543)	437 / 222	0,0002
GAD7-6	0,4989	(0,4512 to 0,5466)	429 / 201	0,9629
PHQ9-6	0,5067	(0,4584 to 0,5549)	431 / 201	0,7868
PCL5-6	0,5491	(0,4996 to 0,5986)	423 / 199	0,0519
RPQ-6	0,4976	(0,4503 to 0,5449)	431 / 208	0,9198
**Wei-Lachin**				
COMBINED	0,4460	(0,4093 to 0,4827)	4304 / 2091	0,0039

GOSE/-Q 7-8 vs. GOSE/-Q 3-4 - Edu > Primary

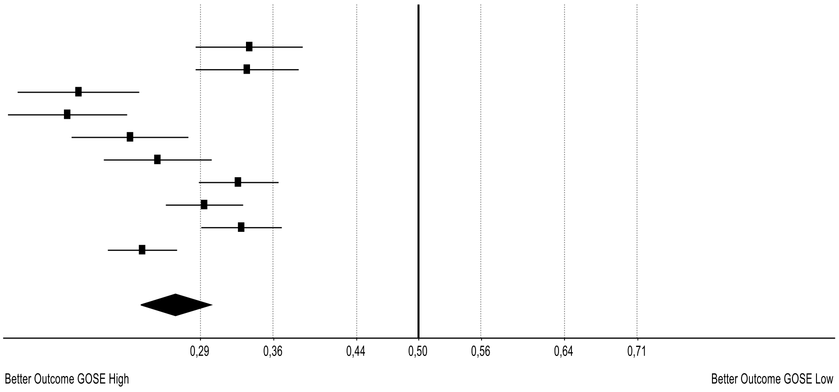

Analysis	MW	95,00%-CI	N1/N2	P-Value
**P1232-6D**				
SF36MCS-6	0,3370	(0,2857 to 0,3884)	853 / 208	0,0000
SF12MCS-6	0,3350	(0,2852 to 0,3848)	892 / 220	0,0000
SF36PCS-6	0,1731	(0,1146 to 0,2315)	851 / 208	0,0000
SF12PCS-6	0,1625	(0,1052 to 0,2197)	892 / 220	0,0000
QOLIBRI-6	0,2224	(0,1665 to 0,2784)	868 / 204	0,0000
QOLI-OS-6	0,2491	(0,1974 to 0,3009)	886 / 222	0,0000
GAD7-6	0,3267	(0,2885 to 0,3649)	856 / 201	0,0000
PHQ9-6	0,2937	(0,2565 to 0,3310)	854 / 201	0,0000
PCL5-6	0,3296	(0,2910 to 0,3682)	863 / 199	0,0000
RPQ-6	0,2341	(0,2008 to 0,2675)	875 / 208	0,0000
**Wei-Lachin**				
COMBINED	0,2663	(0,2327 to 0,2999)	8690 / 2091	0,0000

12 months after TBI (data as available)

GOSE/-Q 7-8 vs. GOSE/-Q 5-6 -Edu ≤ Primary

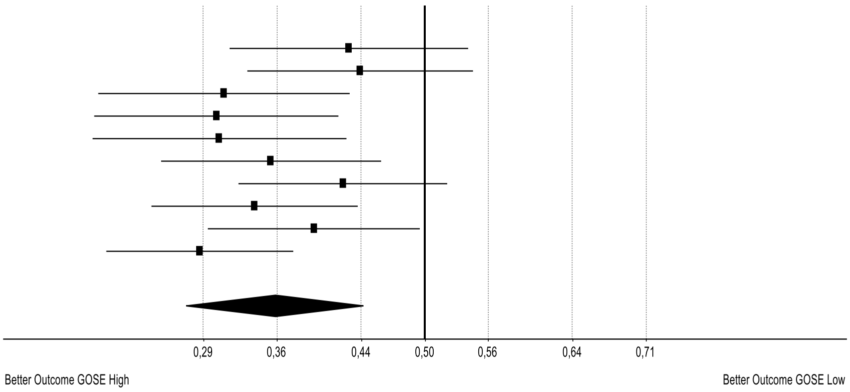

Analysis	MW	95,00%-CI	N1/N2	P-Value
**O1121-YD**				
SF36MCS-12	0,4283	(0,3153 to 0,5414)	95 / 39	0,2140
SF12MCS-12	0,4387	(0,3318 to 0,5456)	102 / 42	0,2613
SF36PCS-12	0,3097	(0,1907 to 0,4287)	95 / 39	0,0017
SF12PCS-12	0,3028	(0,1870 to 0,4185)	102 / 42	0,0008
QOLIBRI-12	0,3054	(0,1849 to 0,4259)	95 / 40	0,0015
QOLI-OS-12	0,3543	(0,2498 to 0,4588)	111 / 47	0,0063
GAD7-12	0,4225	(0,3233 to 0,5217)	96 / 41	0,1259
PHQ9-12	0,3388	(0,2412 to 0,4364)	96 / 40	0,0012
PCL5-12	0,3951	(0,2947 to 0,4954)	96 / 40	0,0403
RPQ-12	0,2870	(0,1982 to 0,3758)	102 / 46	0,0000
**Wei-Lachin**				
COMBINED	0,3583	(0,2742 to 0,4423)	990 / 416	0,0009

GOSE/-Q 5-6 vs. GOSE/-Q 3-4 -Edu ≤ Primary

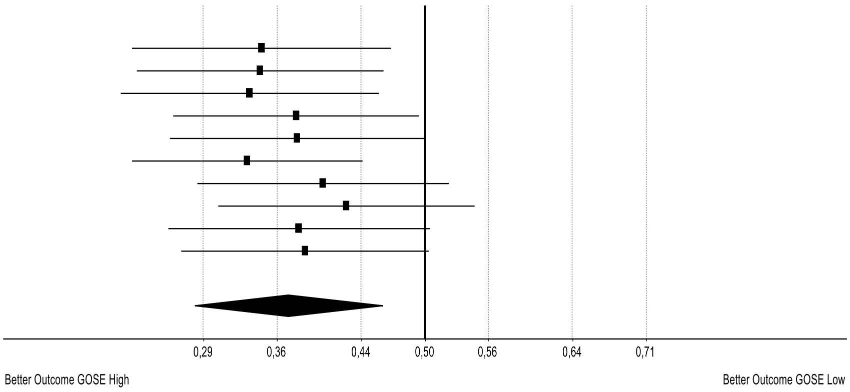

Analysis	MW	95,00%-CI	N1/N2	P-Value
**O2131-YD**				
SF36MCS-12	0,3453	(0,2229 to 0,4677)	39 / 45	0,0132
SF12MCS-12	0,3443	(0,2275 to 0,4610)	42 / 50	0,0089
SF36PCS-12	0,3342	(0,2118 to 0,4566)	39 / 45	0,0079
SF12PCS-12	0,3783	(0,2618 to 0,4948)	42 / 50	0,0407
QOLIBRI-12	0,3791	(0,2583 to 0,4999)	40 / 46	0,0498
QOLI-OS-12	0,3321	(0,2228 to 0,4414)	47 / 56	0,0026
GAD7-12	0,4041	(0,2848 to 0,5233)	41 / 45	0,1149
PHQ9-12	0,4261	(0,3045 to 0,5477)	40 / 45	0,2336
PCL5-12	0,3810	(0,2567 to 0,5052)	40 / 44	0,0605
RPQ-12	0,3867	(0,2694 to 0,5041)	46 / 45	0,0585
**Wei-Lachin**				
COMBINED	0,3711	(0,2822 to 0,4601)	416 / 471	0,0045

GOSE/-Q 7-8 vs. GOSE/-Q 3-4 -Edu ≤ Primary

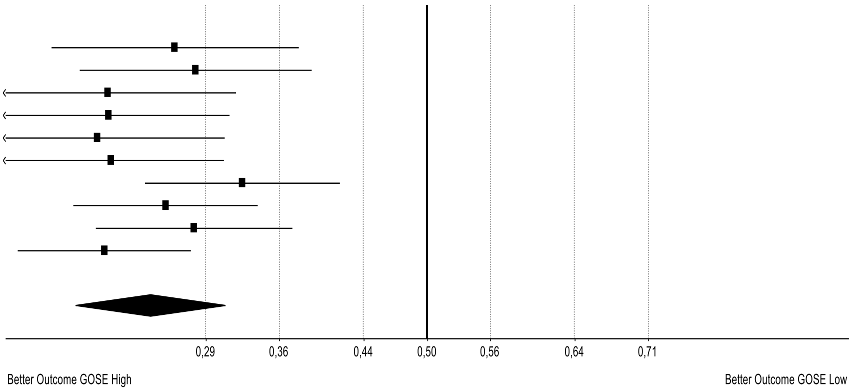

Analysis	MW	95,00%-CI	N1/N2	P-Value
**O1131-YD**				
SF36MCS-12	0,2612	(0,1437 to 0,3787)	95 / 45	0,0001
SF12MCS-12	0,2808	(0,1704 to 0,3911)	102 / 50	0,0001
SF36PCS-12	0,1974	(0,0762 to 0,3186)	95 / 45	0,0000
SF12PCS-12	0,1981	(0,0832 to 0,3131)	102 / 50	0,0000
QOLIBRI-12	0,1876	(0,0671 to 0,3082)	95 / 46	0,0000
QOLI-OS-12	0,2008	(0,0941 to 0,3074)	111 / 56	0,0000
GAD7-12	0,3251	(0,2327 to 0,4176)	96 / 45	0,0002
PHQ9-12	0,2521	(0,1647 to 0,3394)	96 / 45	0,0000
PCL5-12	0,2794	(0,1860 to 0,3727)	96 / 44	0,0000
RPQ-12	0,1942	(0,1120 to 0,2765)	102 / 45	0,0000
**Wei-Lachin**				
COMBINED	0,2377	(0,1668 to 0,3086)	990 / 471	0,0000

GOSE/-Q 7-8 vs. GOSE/-Q 5-6 - Edu > Primary

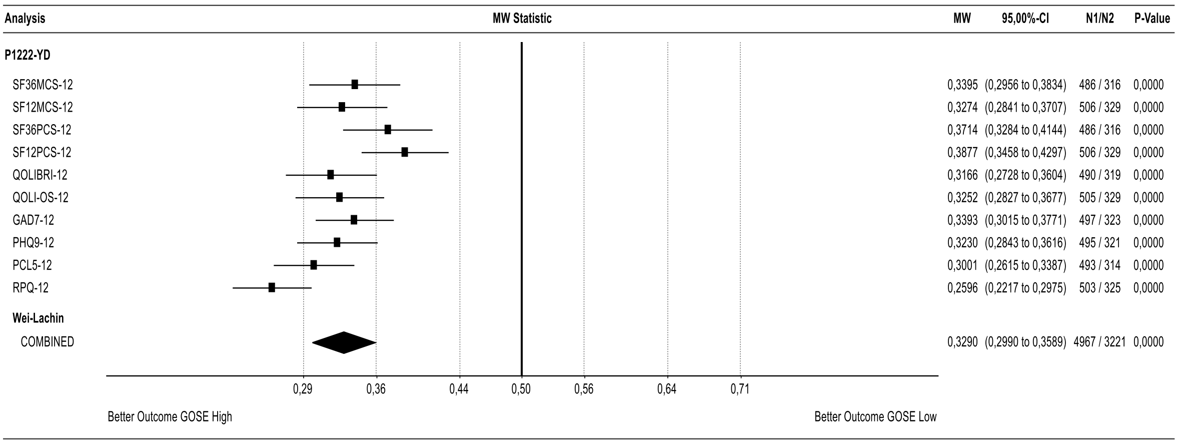

GOSE/-Q 5-6 vs. GOSE/-Q 3-4 - Edu > Primary

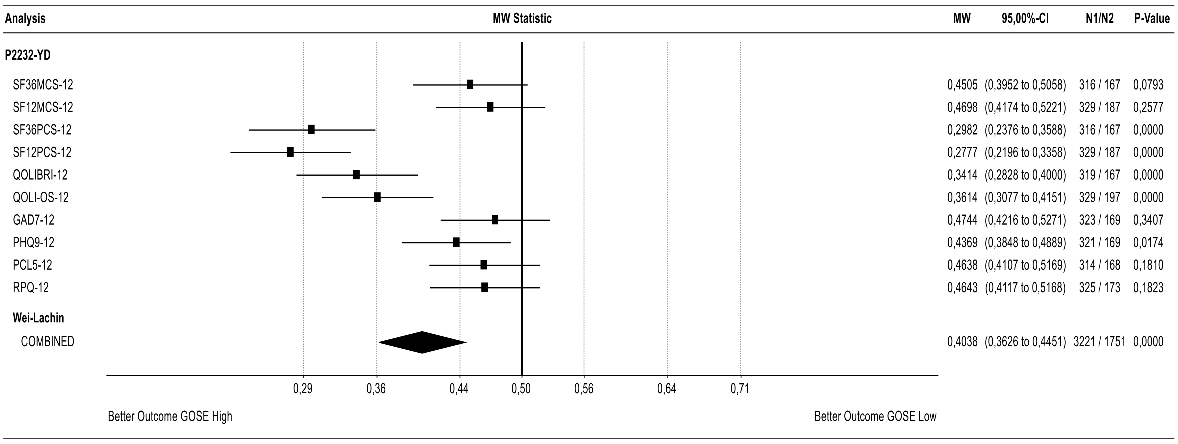

GOSE/-Q 7-8 vs. GOSE/-Q 3-4 - Edu > Primary

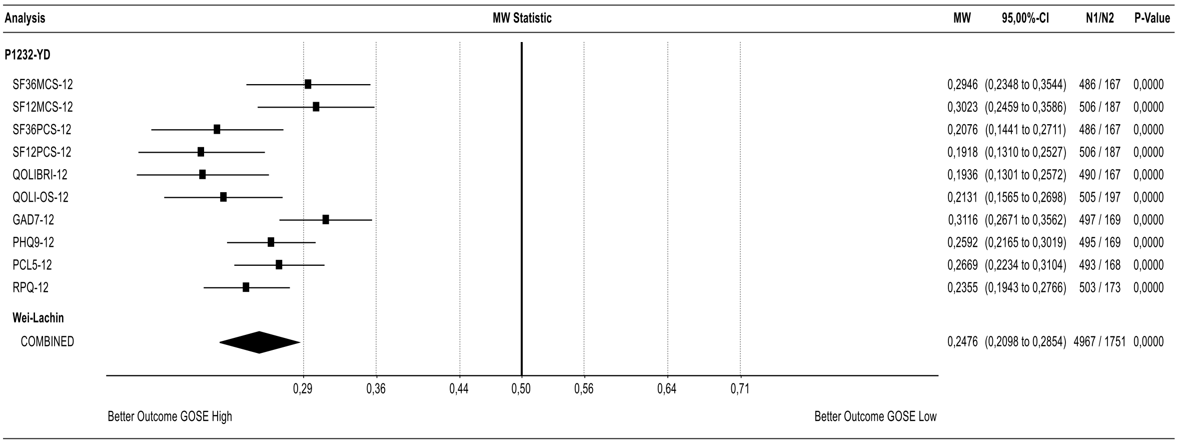

3 months after TBI (completers)

GOSE/-Q 7-8 vs. GOSE/-Q 5-6 -Edu ≤ Primary

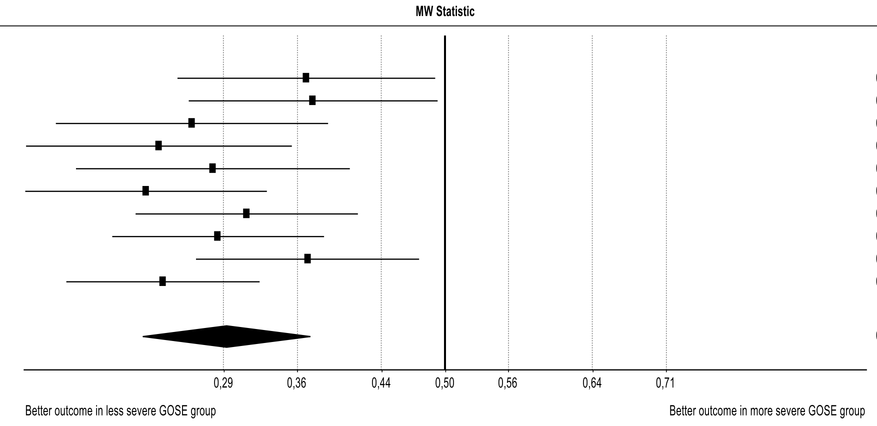

Analysis	MW	95,00%-CI	N1/N2	P-Value
**O1121-3X**				
SF36MCS-X	0,3685	(0,2461 to 0,4909)	75 / 36	0,0353
SF12MCS-X	0,3750	(0,2570 to 0,4929)	85 / 39	0,0378
SF36PCS-X	0,2602	(0,1310 to 0,3893)	75 / 36	0,0003
SF12PCS-X	0,2290	(0,1028 to 0,3551)	85 / 39	0,0000
QOLIBRI-X	0,2801	(0,1502 to 0,4101)	77 / 35	0,0009
QOLI-OS-X	0,2166	(0,1017 to 0,3315)	95 / 45	0,0000
GAD7-X3	0,3123	(0,2067 to 0,4178)	77 / 34	0,0005
PHQ9-X3	0,2848	(0,1842 to 0,3854)	79 / 36	0,0000
PCL5-X3	0,3699	(0,2641 to 0,4758)	77 / 36	0,0160
RPQ-X3	0,2326	(0,1407 to 0,3244)	88 / 45	0,0000
**Wei-Lachin**				
COMBINED	0,2929	(0,2132 to 0,3726)	813 / 381	0,0000

GOSE/-Q 5-6 vs. GOSE/-Q 3-4 -Edu ≤ Primary

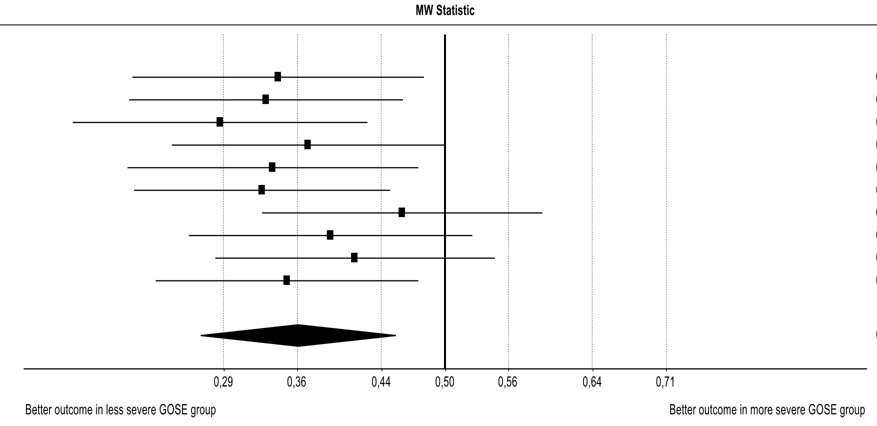

Analysis	MW	95,00%-CI	N1/N2	P-Value
**O2131-3X**				
SF36MCS-X	0,3418	(0,2032 to 0,4803)	36 / 33	0,0252
SF12MCS-X	0,3306	(0,2007 to 0,4606)	39 / 38	0,0106
SF36PCS-X	0,2870	(0,1471 to 0,4269)	36 / 33	0,0029
SF12PCS-X	0,3701	(0,2410 to 0,4992)	39 / 38	0,0486
QOLIBRI-X	0,3368	(0,1988 to 0,4748)	35 / 33	0,0204
QOLI-OS-X	0,3266	(0,2053 to 0,4479)	45 / 43	0,0051
GAD7-X3	0,4594	(0,3265 to 0,5924)	34 / 33	0,5499
PHQ9-X3	0,3914	(0,2571 to 0,5258)	36 / 33	0,1132
PCL5-X3	0,4145	(0,2819 to 0,5471)	36 / 32	0,2064
RPQ-X3	0,3505	(0,2260 to 0,4749)	45 / 35	0,0185
**Wei-Lachin**				
COMBINED	0,3609	(0,2682 to 0,4535)	381 / 351	0,0032

GOSE/-Q 7-8 vs. GOSE/-Q 3-4 -Edu ≤ Primary

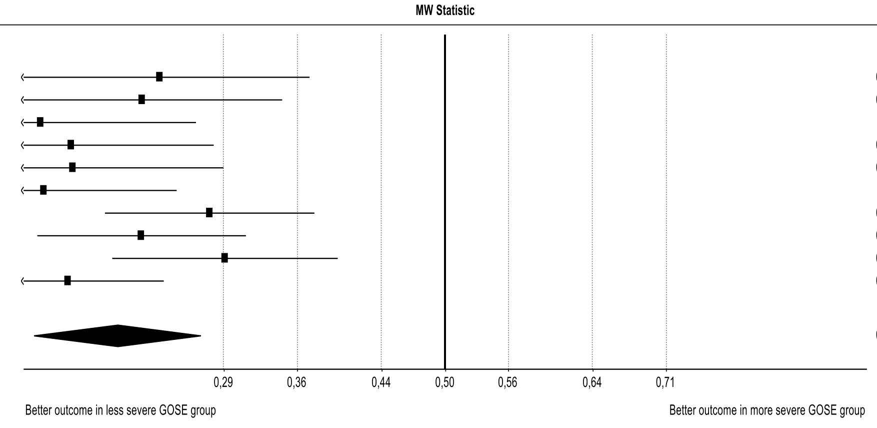

Analysis	MW	95,00%-CI	N1/N2	P-Value
**O1131-3X**				
SF36MCS-X	0,2297	(0,0880 to 0,3714)	75 / 33	0,0002
SF12MCS-X	0,2130	(0,0805 to 0,3455)	85 / 38	0,0000
SF36PCS-X	0,1162	(0,0000 to 0,2642)	75 / 33	0,0000
SF12PCS-X	0,1455	(0,0102 to 0,2808)	85 / 38	0,0000
QOLIBRI-X	0,1470	(0,0040 to 0,2900)	77 / 33	0,0000
QOLI-OS-X	0,1195	(0,0000 to 0,2453)	95 / 43	0,0000
GAD7-X3	0,2769	(0,1773 to 0,3764)	77 / 33	0,0000
PHQ9-X3	0,2121	(0,1133 to 0,3109)	79 / 33	0,0000
PCL5-X3	0,2916	(0,1848 to 0,3984)	77 / 32	0,0001
RPQ-X3	0,1425	(0,0515 to 0,2336)	88 / 35	0,0000
**Wei-Lachin**				
COMBINED	0,1894	(0,1104 to 0,2684)	813 / 351	0,0000

GOSE/-Q 7-8 vs. GOSE/-Q 5-6 – Edu > Primary

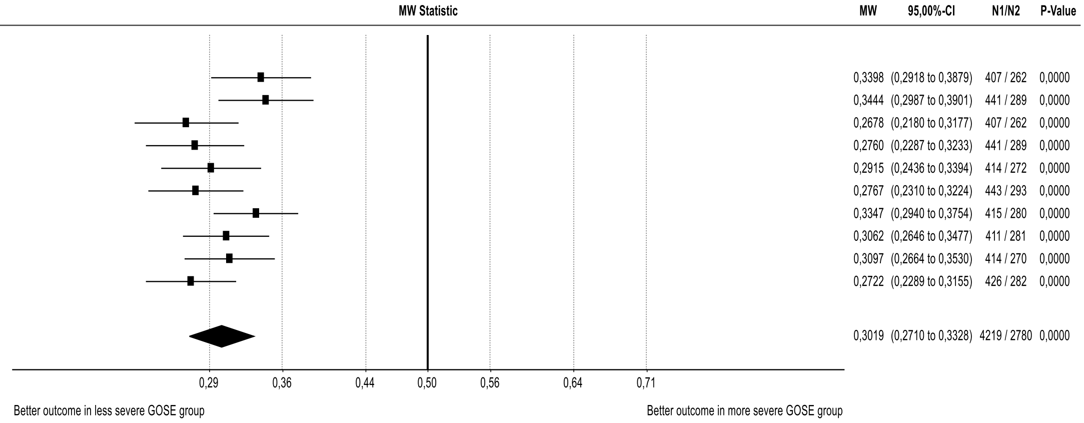

Analysis	MW	95,00%-CI	N1/N2	P-Value
**P1222-3X**				
SF36MCS-X	0,3398	(0,2918 to 0,3879)	407 / 262	0,0000
SF12MCS-X	0,3444	(0,2987 to 0,3901)	441 / 289	0,0000
SF36PCS-X	0,2678	(0,2180 to 0,3177)	407 / 262	0,0000
SF12PCS-X	0,2760	(0,2287 to 0,3233)	441 / 289	0,0000
QOLIBRI-X	0,2915	(0,2436 to 0,3394)	414 / 272	0,0000
QOLI-OS-X	0,2767	(0,2310 to 0,3224)	443 / 293	0,0000
GAD7-X3	0,3347	(0,2940 to 0,3754)	415 / 280	0,0000
PHQ9-X3	0,3062	(0,2646 to 0,3477)	411 / 281	0,0000
PCL5-X3	0,3097	(0,2664 to 0,3530)	414 / 270	0,0000
RPQ-X3	0,2722	(0,2289 to 0,3155)	426 / 282	0,0000
**Wei-Lachin**				
COMBINED	0,3019	(0,2710 to 0,3328)	4219 / 2780	0,0000

GOSE/-Q 5-6 vs. GOSE/-Q 3-4 - Edu > Primary

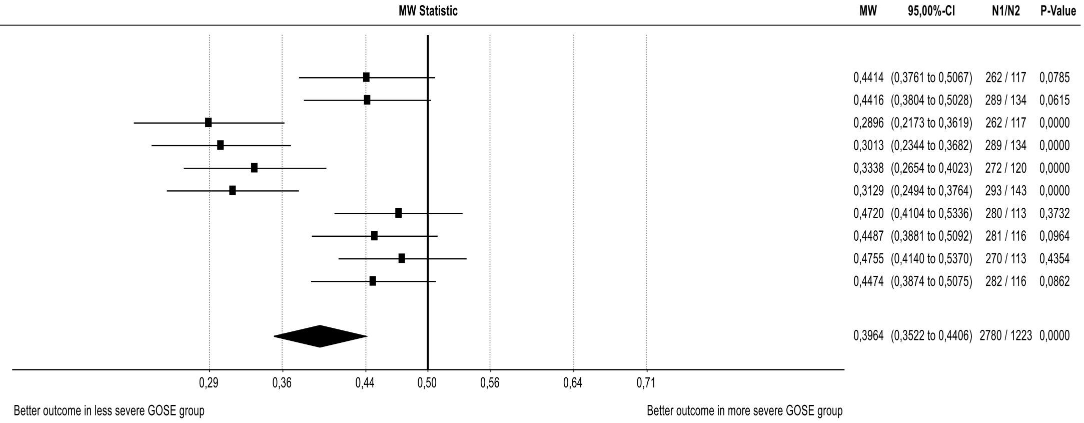

Analysis	MW	95,00%-CI	N1/N2	P-Value
**P2232-3X**				
SF36MCS-X	0,4414	(0,3761 to 0,5067)	262 / 117	0,0785
SF12MCS-X	0,4416	(0,3804 to 0,5028)	289 / 134	0,0615
SF36PCS-X	0,2896	(0,2173 to 0,3619)	262 / 117	0,0000
SF12PCS-X	0,3013	(0,2344 to 0,3682)	289 / 134	0,0000
QOLIBRI-X	0,3338	(0,2654 to 0,4023)	272 / 120	0,0000
QOLI-OS-X	0,3129	(0,2494 to 0,3764)	293 / 143	0,0000
GAD7-X3	0,4720	(0,4104 to 0,5336)	280 / 113	0,3732
PHQ9-X3	0,4487	(0,3881 to 0,5092)	281 / 116	0,0964
PCL5-X3	0,4755	(0,4140 to 0,5370)	270 / 113	0,4354
RPQ-X3	0,4474	(0,3874 to 0,5075)	282 / 116	0,0862
**Wei-Lachin**				
COMBINED	0,3964	(0,3522 to 0,4406)	2780 / 1223	0,0000

GOSE/-Q 7-8 vs. GOSE/-Q 3-4 - Edu > Primary

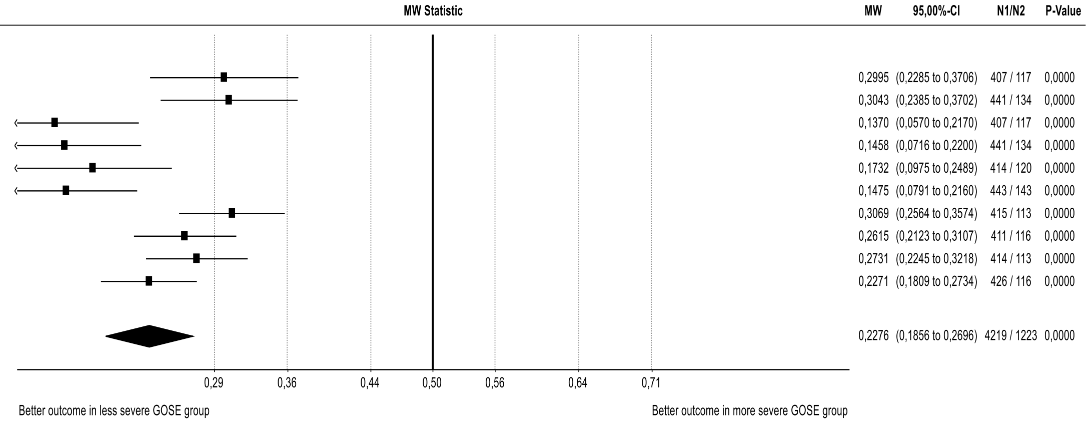

Analysis	MW	95,00%-CI	N1/N2	P-Value
**P1232-3X**				
SF36MCS-X	0,2995	(0,2285 to 0,3706)	407 / 117	0,0000
SF12MCS-X	0,3043	(0,2385 to 0,3702)	441 / 134	0,0000
SF36PCS-X	0,1370	(0,0570 to 0,2170)	407 / 117	0,0000
SF12PCS-X	0,1458	(0,0716 to 0,2200)	441 / 134	0,0000
QOLIBRI-X	0,1732	(0,0975 to 0,2489)	414 / 120	0,0000
QOLI-OS-X	0,1475	(0,0791 to 0,2160)	443 / 143	0,0000
GAD7-X3	0,3069	(0,2564 to 0,3574)	415 / 113	0,0000
PHQ9-X3	0,2615	(0,2123 to 0,3107)	411 / 116	0,0000
PCL5-X3	0,2731	(0,2245 to 0,3218)	414 / 113	0,0000
RPQ-X3	0,2271	(0,1809 to 0,2734)	426 / 116	0,0000
**Wei-Lachin**				
COMBINED	0,2276	(0,1856 to 0,2696)	4219 / 1223	0,0000

6 months after TBI (completers)

GOSE/-Q 7-8 vs. GOSE/-Q 5-6 -Edu ≤ Primary

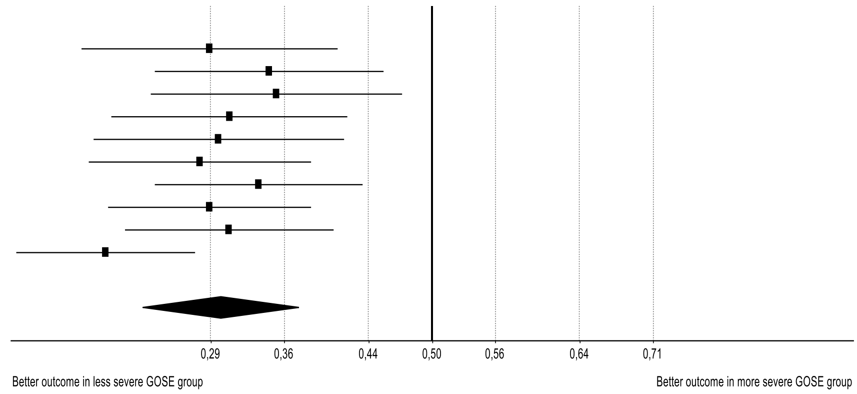

Analysis	MW	95,00%-CI	N1/N2	P-Value
**O1121-6X**				
SF36MCS-X	0,2894	(0,1680 to 0,4108)	82 / 39	0,0007
SF12MCS-X	0,3459	(0,2372 to 0,4545)	90 / 47	0,0054
SF36PCS-X	0,3524	(0,2332 to 0,4716)	82 / 39	0,0152
SF12PCS-X	0,3082	(0,1962 to 0,4201)	90 / 47	0,0008
QOLIBRI-X	0,2978	(0,1793 to 0,4164)	83 / 41	0,0008
QOLI-OS-X	0,2800	(0,1743 to 0,3856)	103 / 52	0,0000
GAD7-X6	0,3357	(0,2372 to 0,4342)	83 / 40	0,0011
PHQ9-X6	0,2893	(0,1931 to 0,3854)	86 / 41	0,0000
PCL5-X6	0,3076	(0,2088 to 0,4064)	84 / 40	0,0001
RPQ-X6	0,1906	(0,1058 to 0,2754)	98 / 48	0,0000
**Wei-Lachin**				
COMBINED	0,2997	(0,2257 to 0,3737)	881 / 434	0,0000

GOSE/-Q 7-8 vs. GOSE/-Q 5-6 - Edu > Primary

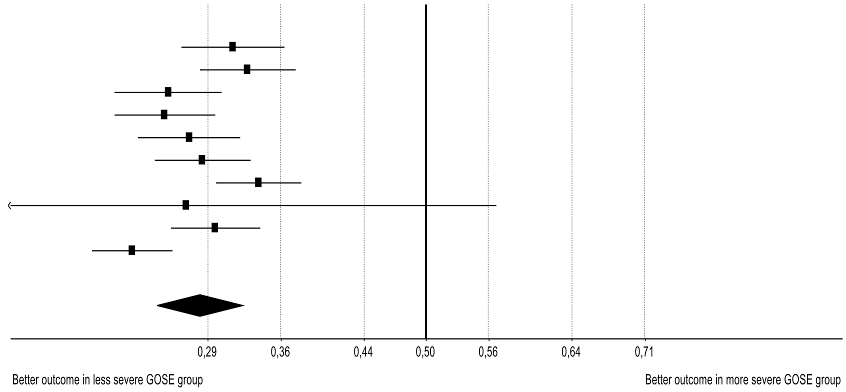

Analysis	MW	95,00%-CI	N1/N2	P-Value
**P1222-6X**				
SF36MCS-X	0,3138	(0,2642 to 0,3634)	473 / 245	0,0000
SF12MCS-X	0,3282	(0,2821 to 0,3743)	507 / 280	0,0000
SF36PCS-X	0,2518	(0,2003 to 0,3033)	473 / 245	0,0000
SF12PCS-X	0,2484	(0,2000 to 0,2968)	507 / 280	0,0000
QOLIBRI-X	0,2720	(0,2229 to 0,3212)	475 / 260	0,0000
QOLI-OS-X	0,2849	(0,2388 to 0,3310)	509 / 282	0,0000
GAD7-X6	0,3390	(0,2982 to 0,3798)	480 / 260	0,0000
PHQ9-X6	0,2691	(0,0000 to 0,5676)	476 / 263	0,1296
PCL5-X6	0,2974	(0,2543 to 0,3405)	474 / 255	0,0000
RPQ-X6	0,2175	(0,1788 to 0,2561)	491 / 263	0,0000
**Wei-Lachin**				
COMBINED	0,2822	(0,2410 to 0,3234)	4865 / 2633	0,0000

GOSE/-Q 5-6 vs. GOSE/-Q 3-4 - Edu > Primary

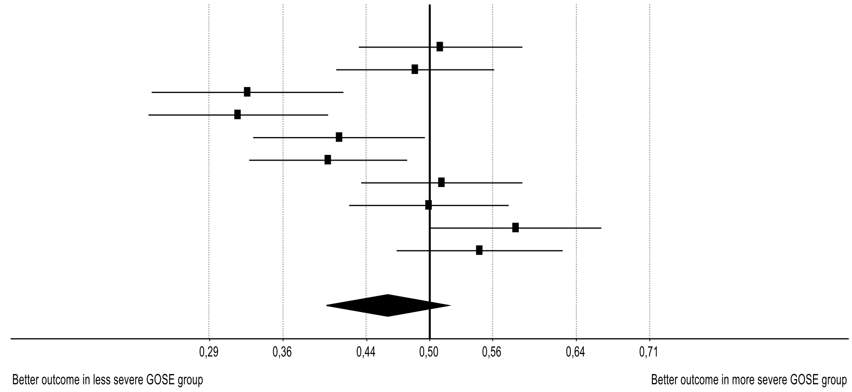

Analysis	MW	95,00%-CI	N1/N2	P-Value
**P2232-6X**				
SF36MCS-X	0,5106	(0,4325 to 0,5887)	245 / 66	0,7900
SF12MCS-X	0,4866	(0,4113 to 0,5619)	280 / 75	0,7272
SF36PCS-X	0,3263	(0,2348 to 0,4179)	245 / 66	0,0002
SF12PCS-X	0,3174	(0,2316 to 0,4033)	280 / 75	0,0000
QOLIBRI-X	0,4139	(0,3318 to 0,4960)	260 / 68	0,0397
QOLI-OS-X	0,4035	(0,3281 to 0,4790)	282 / 85	0,0122
GAD7-X6	0,5120	(0,4350 to 0,5890)	260 / 66	0,7594
PHQ9-X6	0,4998	(0,4237 to 0,5760)	263 / 68	0,9966
PCL5-X6	0,5827	(0,5010 to 0,6644)	255 / 67	0,0473
RPQ-X6	0,5480	(0,4687 to 0,6274)	263 / 68	0,2354
**Wei-Lachin**				
COMBINED	0,4601	(0,4023 to 0,5179)	2633 / 704	0,1760

GOSE/-Q 7-8 vs. GOSE/-Q 3-4 - Edu > Primary

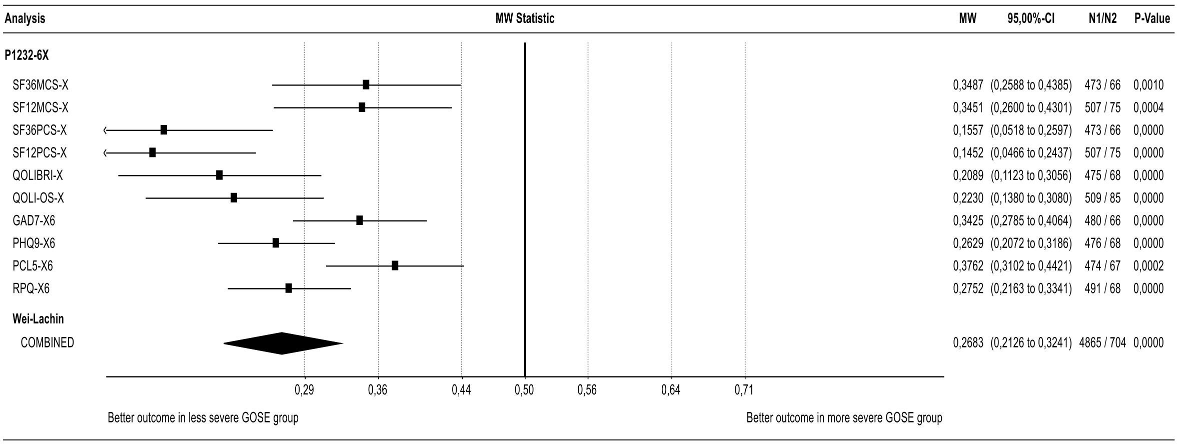

12 months after TBI (completers)

GOSE/-Q 7-8 vs. GOSE/-Q 5-6 -Edu ≤ Primary

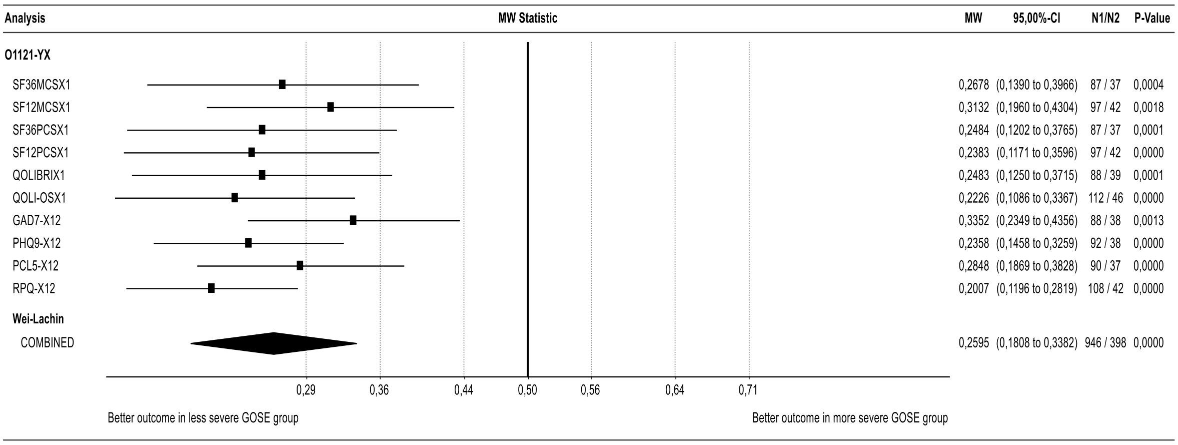

GOSE/-Q 7-8 vs. GOSE/-Q 5-6 - Edu > Primary

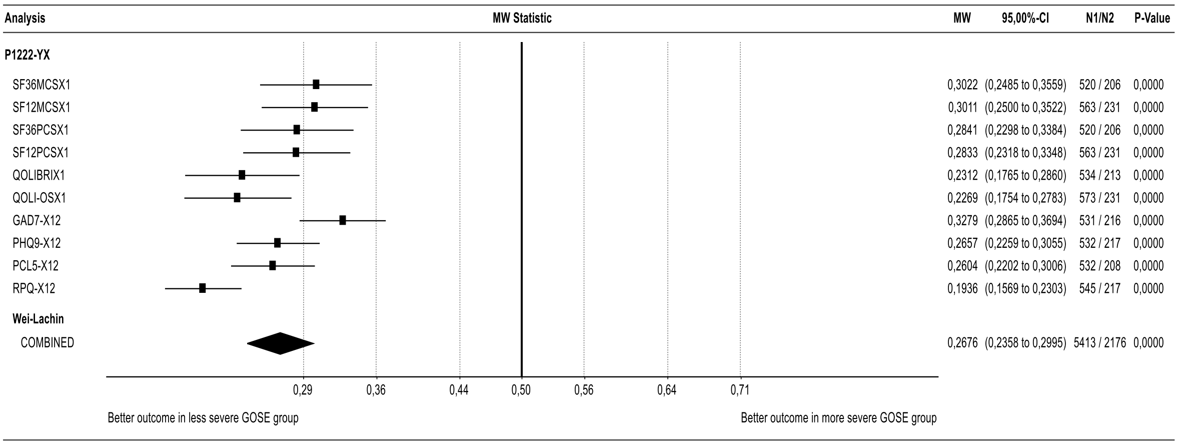

GOSE/-Q 5-6 vs. GOSE/-Q 3-4 - Edu > Primary

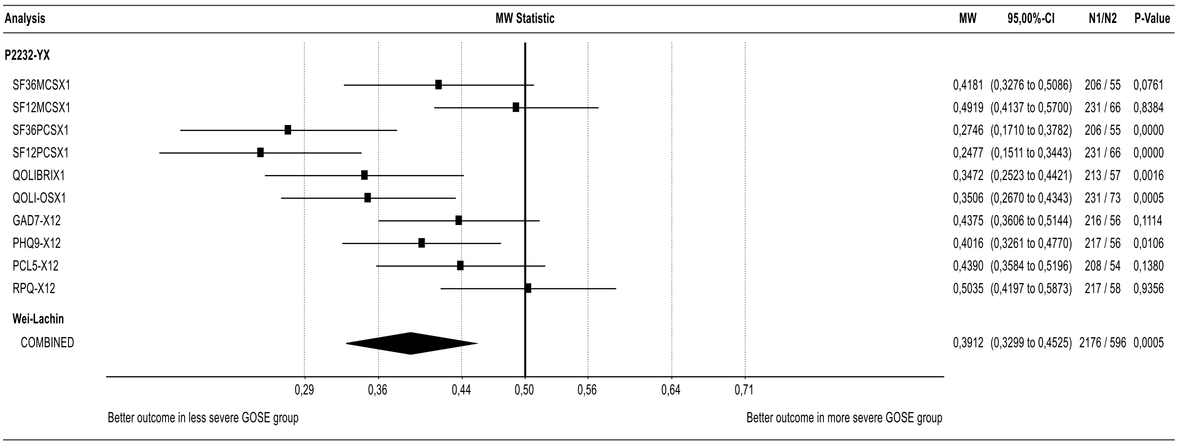

GOSE/-Q 7-8 vs. GOSE/-Q 3-4 - Edu > Primary

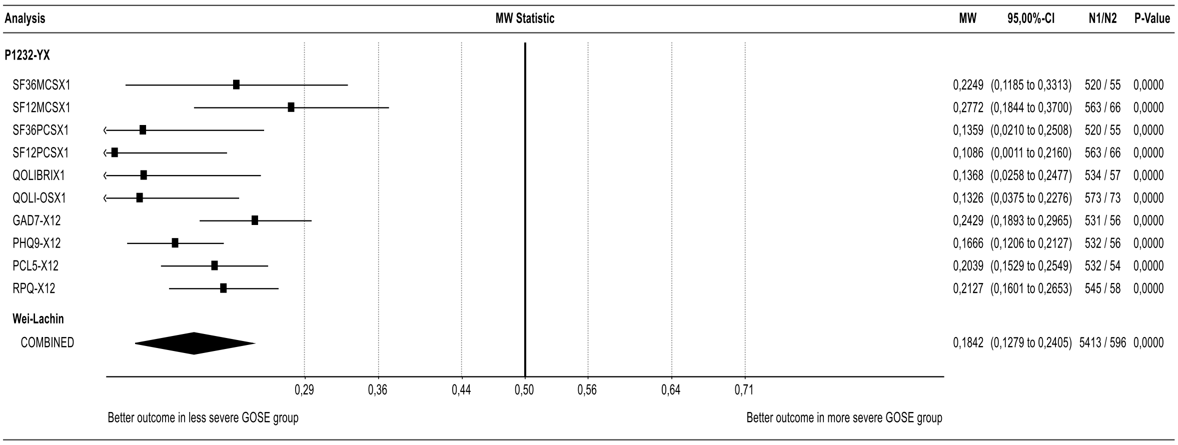

Clinical care pathways

3 months after TBI (data as available)

GOSE/-Q 7-8 vs. GOSE/-Q 5-6 - ER

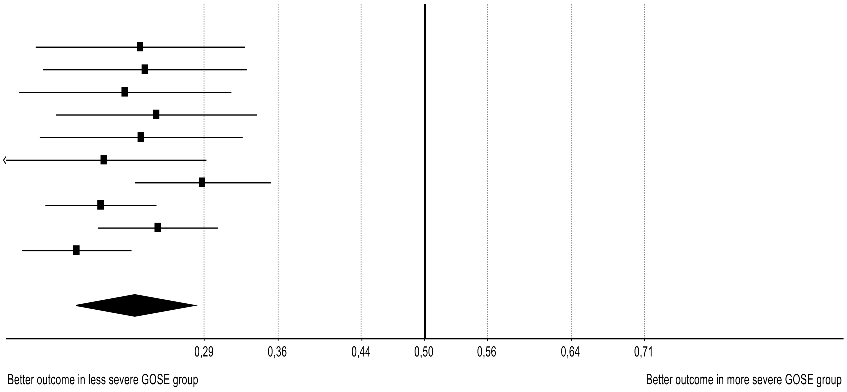

Analysis	MW	95,00%-CI	N1/N2	P-Value
**S1121-3D**				
SF36MCS-3	0,2289	(0,1286 to 0,3292)	395 / 62	0,0000
SF12MCS-3	0,2331	(0,1358 to 0,3303)	420 / 65	0,0000
SF36PCS-3	0,2144	(0,1128 to 0,3161)	395 / 62	0,0000
SF12PCS-3	0,2442	(0,1481 to 0,3404)	420 / 65	0,0000
QOLIBRI-3	0,2298	(0,1330 to 0,3267)	396 / 64	0,0000
QOLI-OS-3	0,1939	(0,0959 to 0,2918)	414 / 63	0,0000
GAD7-3	0,2883	(0,2231 to 0,3535)	389 / 62	0,0000
PHQ9-3	0,1913	(0,1380 to 0,2446)	391 / 63	0,0000
PCL5-3	0,2454	(0,1880 to 0,3027)	396 / 63	0,0000
RPQ-3	0,1679	(0,1158 to 0,2201)	402 / 64	0,0000
**Wei-Lachin**				
COMBINED	0,2237	(0,1671 to 0,2803)	4018 / 633	0,0000

GOSE/-Q 7-8 vs. GOSE/-Q 5-6- Admission

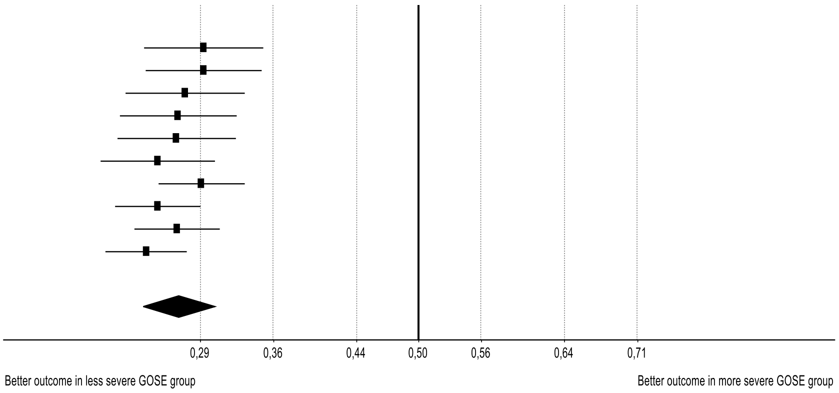

Analysis	MW	95,00%-CI	N1/N2	P-Value
**T1222-3D**				
SF36MCS-3	0,2934	(0,2359 to 0,3509)	552 / 180	0,0000
SF12MCS-3	0,2932	(0,2377 to 0,3486)	599 / 193	0,0000
SF36PCS-3	0,2754	(0,2178 to 0,3331)	552 / 180	0,0000
SF12PCS-3	0,2686	(0,2126 to 0,3246)	599 / 193	0,0000
QOLIBRI-3	0,2671	(0,2102 to 0,3240)	560 / 185	0,0000
QOLI-OS-3	0,2492	(0,1944 to 0,3040)	599 / 194	0,0000
GAD7-3	0,2911	(0,2495 to 0,3327)	548 / 183	0,0000
PHQ9-3	0,2491	(0,2084 to 0,2898)	551 / 183	0,0000
PCL5-3	0,2677	(0,2266 to 0,3088)	547 / 184	0,0000
RPQ-3	0,2380	(0,1988 to 0,2771)	571 / 190	0,0000
**Wei-Lachin**				
COMBINED	0,2693	(0,2349 to 0,3037)	5678 / 1865	0,0000

GOSE/-Q 5-6 vs. GOSE/-Q 3-4 - Admission

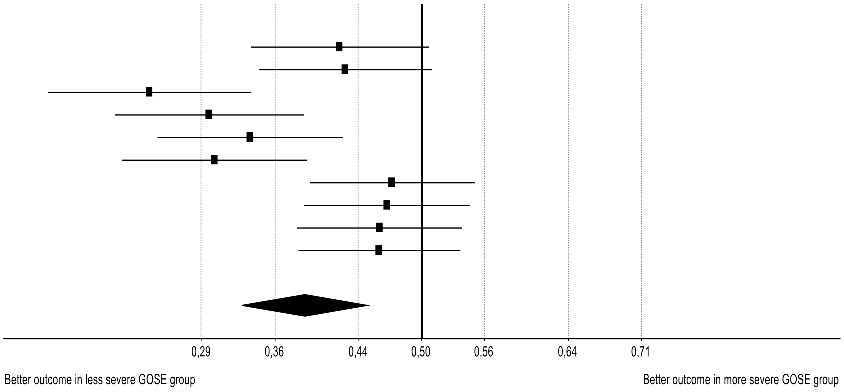

Analysis	MW	95,00%-CI	N1/N2	P-Value
**T2232-3D**				
SF36MCS-3	0,4221	(0,3372 to 0,5070)	180 / 67	0,0721
SF12MCS-3	0,4276	(0,3450 to 0,5102)	193 / 70	0,0858
SF36PCS-3	0,2401	(0,1431 to 0,3371)	180 / 67	0,0000
SF12PCS-3	0,2976	(0,2073 to 0,3879)	193 / 70	0,0000
QOLIBRI-3	0,3365	(0,2482 to 0,4249)	185 / 69	0,0003
QOLI-OS-3	0,3029	(0,2143 to 0,3914)	194 / 70	0,0000
GAD7-3	0,4720	(0,3931 to 0,5509)	183 / 67	0,4864
PHQ9-3	0,4674	(0,3883 to 0,5465)	183 / 68	0,4193
PCL5-3	0,4600	(0,3811 to 0,5390)	184 / 67	0,3209
RPQ-3	0,4598	(0,3826 to 0,5371)	190 / 69	0,3080
**Wei-Lachin**				
COMBINED	0,3886	(0,3290 to 0,4482)	1865 / 684	0,0002

GOSE/-Q 7-8 vs. GOSE/-Q 3-4 - Admission

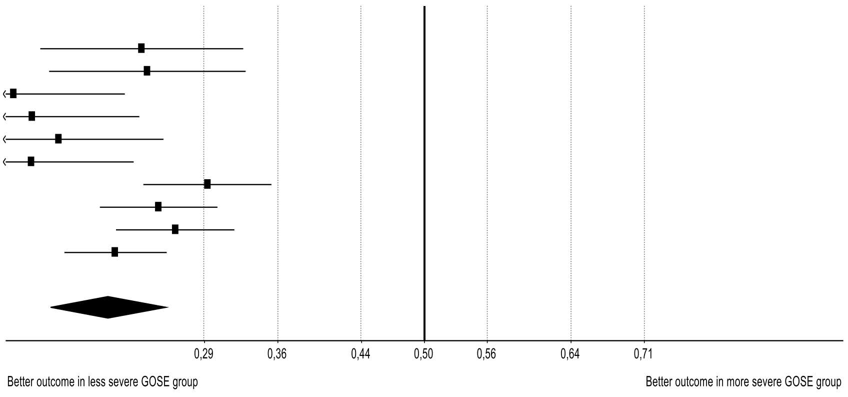

Analysis	MW	95,00%-CI	N1/N2	P-Value
**T1232-3D**				
SF36MCS-3	0,2304	(0,1333 to 0,3275)	552 / 67	0,0000
SF12MCS-3	0,2360	(0,1421 to 0,3298)	599 / 70	0,0000
SF36PCS-3	0,1084	(0,0022 to 0,2145)	552 / 67	0,0000
SF12PCS-3	0,1258	(0,0237 to 0,2279)	599 / 70	0,0000
QOLIBRI-3	0,1514	(0,0517 to 0,2511)	560 / 69	0,0000
QOLI-OS-3	0,1247	(0,0264 to 0,2229)	599 / 70	0,0000
GAD7-3	0,2931	(0,2318 to 0,3544)	548 / 67	0,0000
PHQ9-3	0,2464	(0,1904 to 0,3024)	551 / 68	0,0000
PCL5-3	0,2626	(0,2060 to 0,3192)	547 / 67	0,0000
RPQ-3	0,2053	(0,1566 to 0,2541)	571 / 69	0,0000
**Wei-Lachin**				
COMBINED	0,1984	(0,1436 to 0,2532)	5678 / 684	0,0000

GOSE/-Q 7-8 vs. GOSE/-Q 5-6- ICU

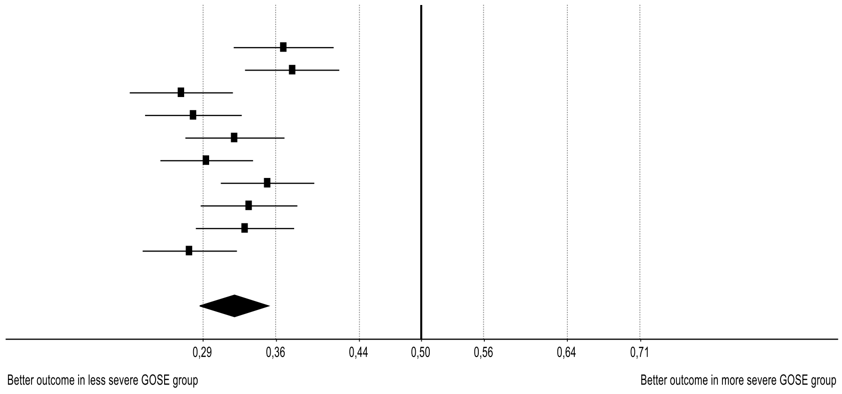

Analysis	MW	95,00%-CI	N1/N2	P-Value
**U1323-3D**				
SF36MCS-3	0,3676	(0,3195 to 0,4158)	285 / 283	0,0000
SF12MCS-3	0,3758	(0,3306 to 0,4209)	320 / 322	0,0000
SF36PCS-3	0,2689	(0,2193 to 0,3184)	285 / 283	0,0000
SF12PCS-3	0,2807	(0,2343 to 0,3270)	320 / 322	0,0000
QOLIBRI-3	0,3206	(0,2731 to 0,3681)	291 / 297	0,0000
QOLI-OS-3	0,2936	(0,2488 to 0,3385)	329 / 329	0,0000
GAD7-3	0,3523	(0,3073 to 0,3973)	288 / 298	0,0000
PHQ9-3	0,3341	(0,2878 to 0,3805)	287 / 297	0,0000
PCL5-3	0,3306	(0,2832 to 0,3780)	286 / 292	0,0000
RPQ-3	0,2773	(0,2319 to 0,3226)	296 / 307	0,0000
**Wei-Lachin**				
COMBINED	0,3202	(0,2872 to 0,3531)	2987 / 3030	0,0000

GOSE/-Q 5-6 vs. GOSE/-Q 3-4 - ICU

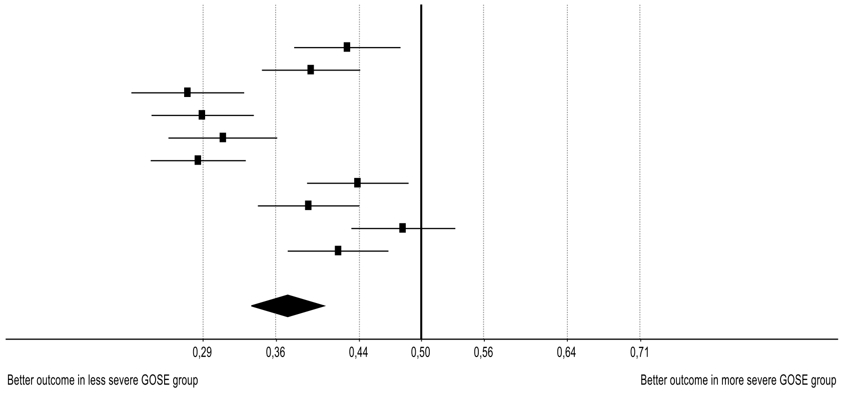

Analysis	MW	95,00%-CI	N1/N2	P-Value
**U2333-3D**				
SF36MCS-3	0,4289	(0,3778 to 0,4799)	283 / 229	0,0063
SF12MCS-3	0,3938	(0,3463 to 0,4413)	322 / 274	0,0000
SF36PCS-3	0,2753	(0,2212 to 0,3293)	283 / 229	0,0000
SF12PCS-3	0,2895	(0,2403 to 0,3388)	322 / 274	0,0000
QOLIBRI-3	0,3092	(0,2569 to 0,3616)	297 / 234	0,0000
QOLI-OS-3	0,2855	(0,2396 to 0,3314)	329 / 313	0,0000
GAD7-3	0,4386	(0,3898 to 0,4874)	298 / 232	0,0137
PHQ9-3	0,3916	(0,3430 to 0,4402)	297 / 234	0,0000
PCL5-3	0,4825	(0,4327 to 0,5323)	292 / 225	0,4915
RPQ-3	0,4201	(0,3718 to 0,4684)	307 / 237	0,0012
**Wei-Lachin**				
COMBINED	0,3715	(0,3364 to 0,4066)	3030 / 2481	0,0000

GOSE/-Q 7-8 vs. GOSE/-Q 3-4 - ICU

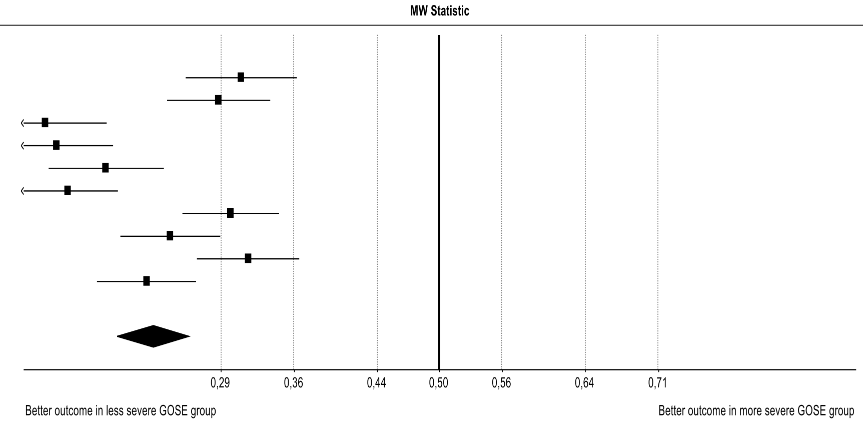

Analysis	MW	95,00%-CI	N1/N2	P-Value
**U1333-3D**				
SF36MCS-3	0,3096	(0,2561 to 0,3631)	285 / 229	0,0000
SF12MCS-3	0,2876	(0,2381 to 0,3371)	320 / 274	0,0000
SF36PCS-3	0,1212	(0,0623 to 0,1801)	285 / 229	0,0000
SF12PCS-3	0,1325	(0,0789 to 0,1861)	320 / 274	0,0000
QOLIBRI-3	0,1797	(0,1241 to 0,2353)	291 / 234	0,0000
QOLI-OS-3	0,1433	(0,0953 to 0,1913)	329 / 313	0,0000
GAD7-3	0,2994	(0,2529 to 0,3459)	288 / 232	0,0000
PHQ9-3	0,2413	(0,1932 to 0,2894)	287 / 234	0,0000
PCL5-3	0,3162	(0,2668 to 0,3656)	286 / 225	0,0000
RPQ-3	0,2185	(0,1711 to 0,2658)	296 / 237	0,0000
**Wei-Lachin**				
COMBINED	0,2249	(0,1906 to 0,2592)	2987 / 2481	0,0000

6 months after TBI (data as available)

GOSE/-Q 7-8 vs. GOSE/-Q 5-6 - ER

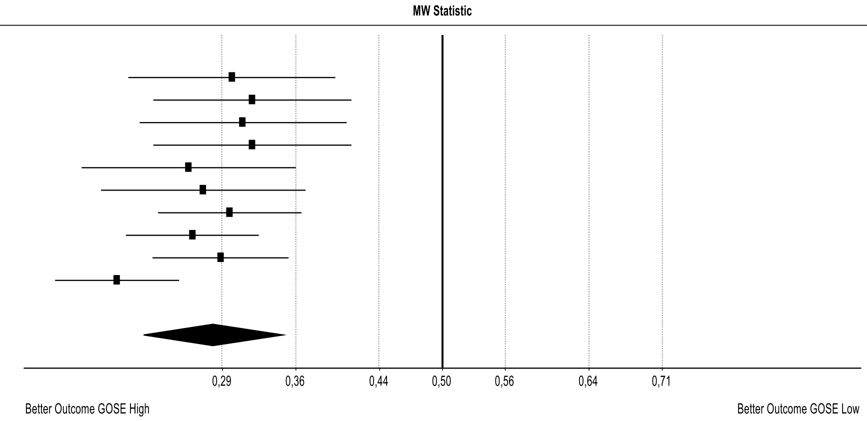

Analysis	MW	95,00%-CI	N1/N2	P-Value
**S1121-6D**				
SF36MCS-6	0,2994	(0,2006 to 0,3983)	347 / 56	0,0001
SF12MCS-6	0,3188	(0,2244 to 0,4132)	363 / 59	0,0002
SF36PCS-6	0,3099	(0,2112 to 0,4086)	346 / 56	0,0002
SF12PCS-6	0,3186	(0,2239 to 0,4134)	363 / 59	0,0002
QOLIBRI-6	0,2582	(0,1559 to 0,3606)	350 / 56	0,0000
QOLI-OS-6	0,2720	(0,1744 to 0,3697)	357 / 58	0,0000
GAD7-6	0,2972	(0,2286 to 0,3658)	340 / 57	0,0000
PHQ9-6	0,2617	(0,1982 to 0,3252)	341 / 57	0,0000
PCL5-6	0,2886	(0,2236 to 0,3536)	349 / 56	0,0000
RPQ-6	0,1896	(0,1301 to 0,2492)	354 / 59	0,0000
**Wei-Lachin**				
COMBINED	0,2814	(0,2151 to 0,3478)	3510 / 573	0,0000

GOSE/-Q 7-8 vs. GOSE/-Q 5-6- Admission

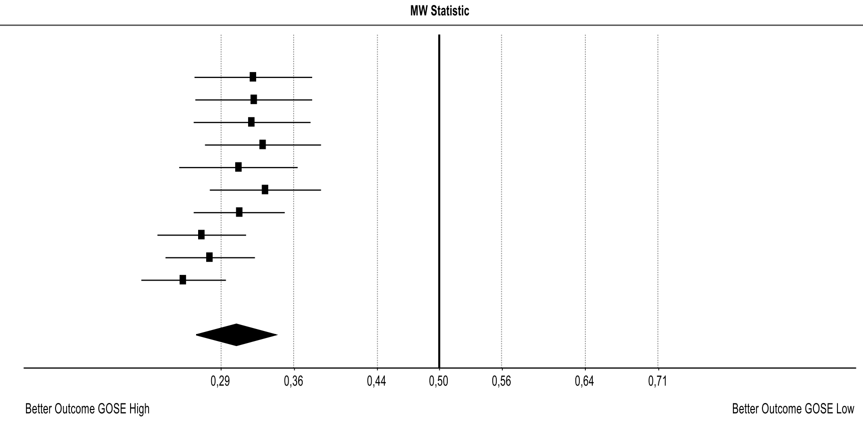

Analysis	MW	95,00%-CI	N1/N2	P-Value
**T1222-6D**				
SF36MCS-6	0,3213	(0,2649 to 0,3778)	503 / 178	0,0000
SF12MCS-6	0,3217	(0,2656 to 0,3778)	523 / 179	0,0000
SF36PCS-6	0,3199	(0,2636 to 0,3761)	502 / 178	0,0000
SF12PCS-6	0,3302	(0,2745 to 0,3859)	523 / 179	0,0000
QOLIBRI-6	0,3069	(0,2502 to 0,3637)	509 / 174	0,0000
QOLI-OS-6	0,3330	(0,2796 to 0,3864)	530 / 183	0,0000
GAD7-6	0,3077	(0,2639 to 0,3515)	505 / 174	0,0000
PHQ9-6	0,2718	(0,2291 to 0,3145)	506 / 175	0,0000
PCL5-6	0,2795	(0,2366 to 0,3224)	503 / 176	0,0000
RPQ-6	0,2539	(0,2132 to 0,2946)	521 / 180	0,0000
**Wei-Lachin**				
COMBINED	0,3046	(0,2665 to 0,3427)	5125 / 1776	0,0000

GOSE/-Q 5-6 vs. GOSE/-Q 3-4 - Admission

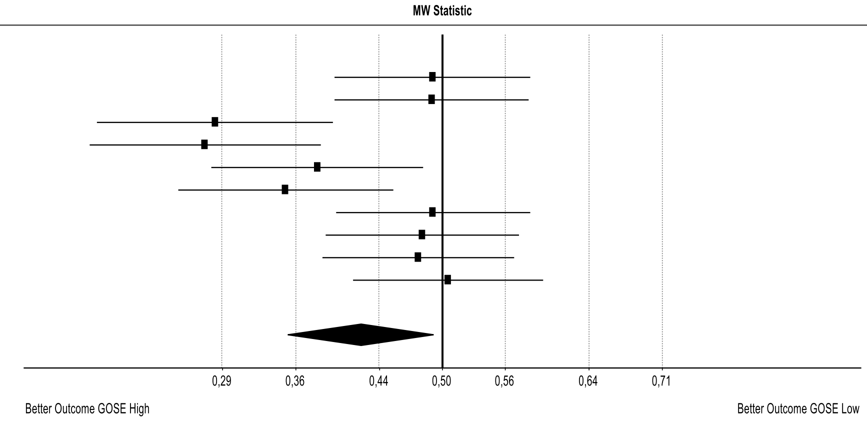

Analysis	MW	95,00%-CI	N1/N2	P-Value
**T2232-6D**				
SF36MCS-6	0,4908	(0,3970 to 0,5846)	178 / 46	0,8472
SF12MCS-6	0,4902	(0,3975 to 0,5828)	179 / 48	0,8352
SF36PCS-6	0,2833	(0,1707 to 0,3958)	178 / 46	0,0002
SF12PCS-6	0,2737	(0,1634 to 0,3839)	179 / 48	0,0001
QOLIBRI-6	0,3809	(0,2797 to 0,4820)	174 / 46	0,0210
QOLI-OS-6	0,3507	(0,2482 to 0,4532)	183 / 47	0,0043
GAD7-6	0,4914	(0,3988 to 0,5841)	174 / 45	0,8563
PHQ9-6	0,4812	(0,3889 to 0,5735)	175 / 45	0,6899
PCL5-6	0,4776	(0,3860 to 0,5692)	176 / 43	0,6319
RPQ-6	0,5057	(0,4148 to 0,5966)	180 / 47	0,9016
**Wei-Lachin**				
COMBINED	0,4225	(0,3528 to 0,4923)	1776 / 461	0,0294

GOSE/-Q 7-8 vs. GOSE/-Q 3-4 - Admission

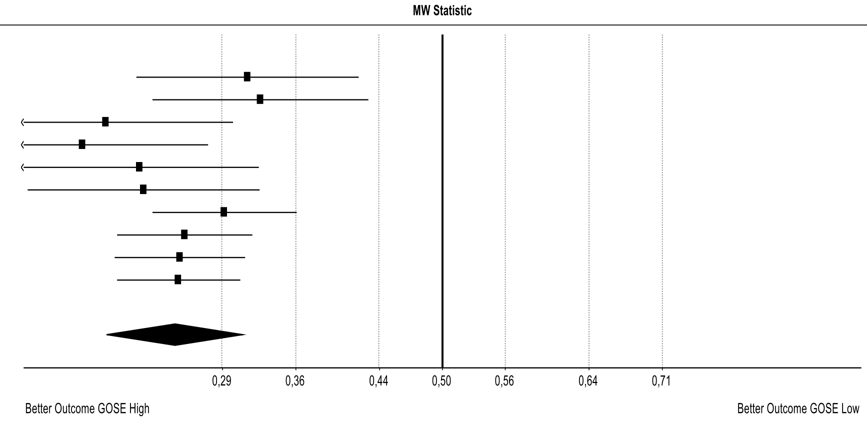

Analysis	MW	95,00%-CI	N1/N2	P-Value
**T1232-6D**				
SF36MCS-6	0,3142	(0,2080 to 0,4205)	503 / 46	0,0006
SF12MCS-6	0,3267	(0,2234 to 0,4300)	523 / 48	0,0010
SF36PCS-6	0,1790	(0,0576 to 0,3005)	502 / 46	0,0000
SF12PCS-6	0,1562	(0,0358 to 0,2765)	523 / 48	0,0000
QOLIBRI-6	0,2115	(0,0980 to 0,3250)	509 / 46	0,0000
QOLI-OS-6	0,2149	(0,1039 to 0,3260)	530 / 47	0,0000
GAD7-6	0,2921	(0,2233 to 0,3608)	505 / 45	0,0000
PHQ9-6	0,2543	(0,1897 to 0,3189)	506 / 45	0,0000
PCL5-6	0,2497	(0,1871 to 0,3123)	503 / 43	0,0000
RPQ-6	0,2484	(0,1896 to 0,3071)	521 / 47	0,0000
**Wei-Lachin**				
COMBINED	0,2447	(0,1799 to 0,3095)	5125 / 461	0,0000

GOSE/-Q 7-8 vs. GOSE/-Q 5-6- ICU

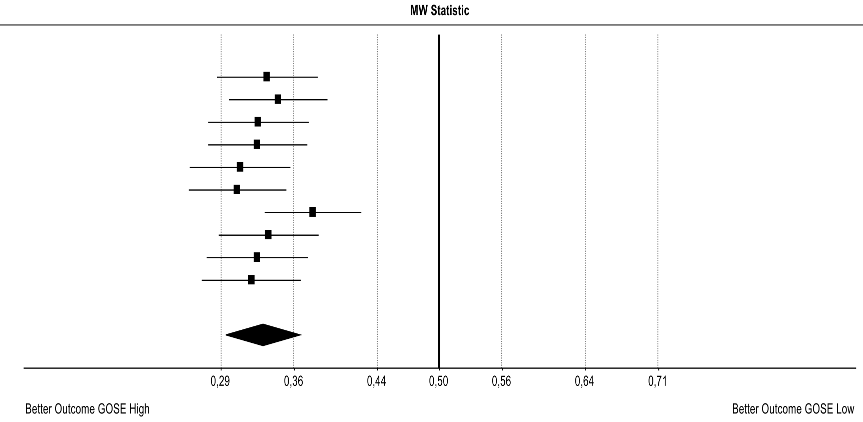

Analysis	MW	95,00%-CI	N1/N2	P-Value
**U1323-6D**				
SF36MCS-6	0,3346	(0,2863 to 0,3830)	257 / 298	0,0000
SF12MCS-6	0,3448	(0,2975 to 0,3921)	275 / 306	0,0000
SF36PCS-6	0,3258	(0,2774 to 0,3742)	257 / 298	0,0000
SF12PCS-6	0,3253	(0,2778 to 0,3728)	275 / 306	0,0000
QOLIBRI-6	0,3087	(0,2603 to 0,3570)	263 / 296	0,0000
QOLI-OS-6	0,3060	(0,2593 to 0,3528)	279 / 310	0,0000
GAD7-6	0,3783	(0,3320 to 0,4246)	264 / 298	0,0000
PHQ9-6	0,3359	(0,2880 to 0,3837)	262 / 300	0,0000
PCL5-6	0,3251	(0,2764 to 0,3739)	267 / 295	0,0000
RPQ-6	0,3195	(0,2719 to 0,3670)	271 / 306	0,0000
**Wei-Lachin**				
COMBINED	0,3304	(0,2949 to 0,3659)	2670 / 3013	0,0000

GOSE/-Q 5-6 vs. GOSE/-Q 3-4 - ICU

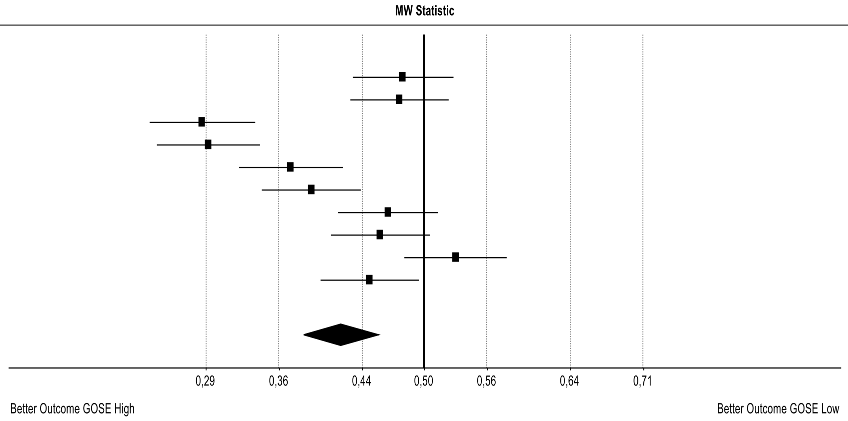

Analysis	MW	95,00%-CI	N1/N2	P-Value
**U2333-6D**				
SF36MCS-6	0,4793	(0,4311 to 0,5275)	298 / 258	0,3997
SF12MCS-6	0,4758	(0,4286 to 0,5230)	306 / 273	0,3145
SF36PCS-6	0,2866	(0,2357 to 0,3376)	298 / 258	0,0000
SF12PCS-6	0,2926	(0,2431 to 0,3421)	306 / 273	0,0000
QOLIBRI-6	0,3718	(0,3220 to 0,4216)	296 / 252	0,0000
QOLI-OS-6	0,3912	(0,3439 to 0,4386)	310 / 279	0,0000
GAD7-6	0,4651	(0,4173 to 0,5129)	298 / 250	0,1528
PHQ9-6	0,4577	(0,4099 to 0,5055)	300 / 251	0,0831
PCL5-6	0,5299	(0,4809 to 0,5790)	295 / 244	0,2316
RPQ-6	0,4472	(0,3998 to 0,4946)	306 / 256	0,0291
**Wei-Lachin**				
COMBINED	0,4197	(0,3835 to 0,4560)	3013 / 2594	0,0000

GOSE/-Q 7-8 vs. GOSE/-Q 3-4 - ICU

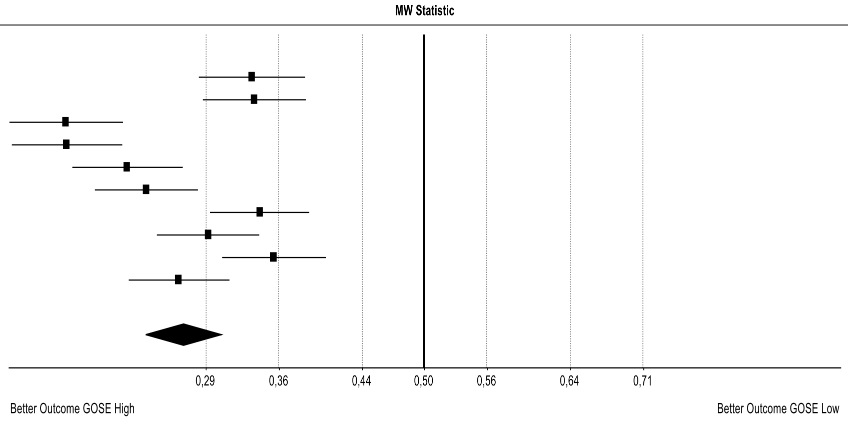

Analysis	MW	95,00%-CI	N1/N2	P-Value
**U1333-6D**				
SF36MCS-6	0,3343	(0,2832 to 0,3853)	257 / 258	0,0000
SF12MCS-6	0,3367	(0,2873 to 0,3862)	275 / 273	0,0000
SF36PCS-6	0,1557	(0,1011 to 0,2104)	257 / 258	0,0000
SF12PCS-6	0,1564	(0,1032 to 0,2096)	275 / 273	0,0000
QOLIBRI-6	0,2146	(0,1614 to 0,2678)	263 / 252	0,0000
QOLI-OS-6	0,2329	(0,1832 to 0,2826)	279 / 279	0,0000
GAD7-6	0,3418	(0,2943 to 0,3893)	264 / 250	0,0000
PHQ9-6	0,2921	(0,2430 to 0,3413)	262 / 251	0,0000
PCL5-6	0,3554	(0,3055 to 0,4054)	267 / 244	0,0000
RPQ-6	0,2641	(0,2154 to 0,3128)	271 / 256	0,0000
**Wei-Lachin**				
COMBINED	0,2684	(0,2323 to 0,3046)	2670 / 2594	0,0000

<u>12 months after TBI (data as available)</u>

GOSE/-Q 7-8 vs. GOSE/-Q 5-6- Admission

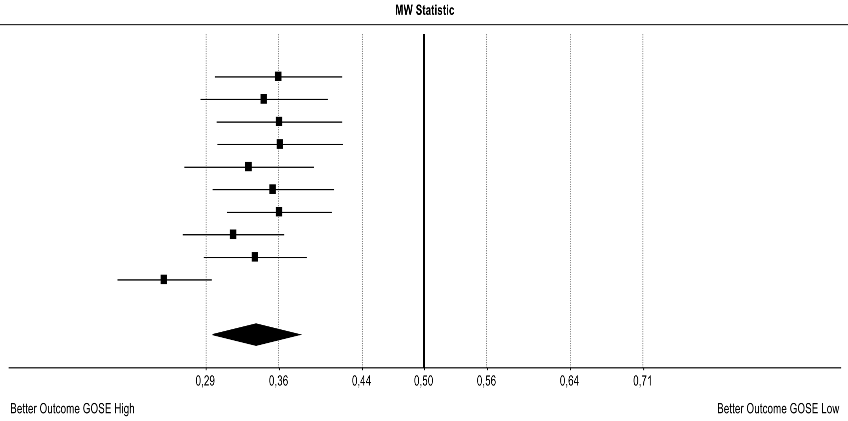

Analysis	MW	95,00%-CI	N1/N2	P-Value
**T1222-YD**				
SF36MCS-12	0,3599	(0,2988 to 0,4210)	423 / 143	0,0000
SF12MCS-12	0,3461	(0,2847 to 0,4074)	440 / 144	0,0000
SF36PCS-12	0,3604	(0,2999 to 0,4208)	423 / 143	0,0000
SF12PCS-12	0,3615	(0,3012 to 0,4219)	440 / 144	0,0000
QOLIBRI-12	0,3313	(0,2689 to 0,3937)	427 / 139	0,0000
QOLI-OS-12	0,3546	(0,2960 to 0,4133)	447 / 147	0,0000
GAD7-12	0,3605	(0,3104 to 0,4107)	428 / 142	0,0000
PHQ9-12	0,3166	(0,2678 to 0,3653)	426 / 142	0,0000
PCL5-12	0,3374	(0,2876 to 0,3873)	426 / 139	0,0000
RPQ-12	0,2500	(0,2047 to 0,2952)	441 / 146	0,0000
**Wei-Lachin**				
COMBINED	0,3378	(0,2960 to 0,3797)	4321 / 1429	0,0000

GOSE/-Q 5-6 vs. GOSE/-Q 3-4 - Admission

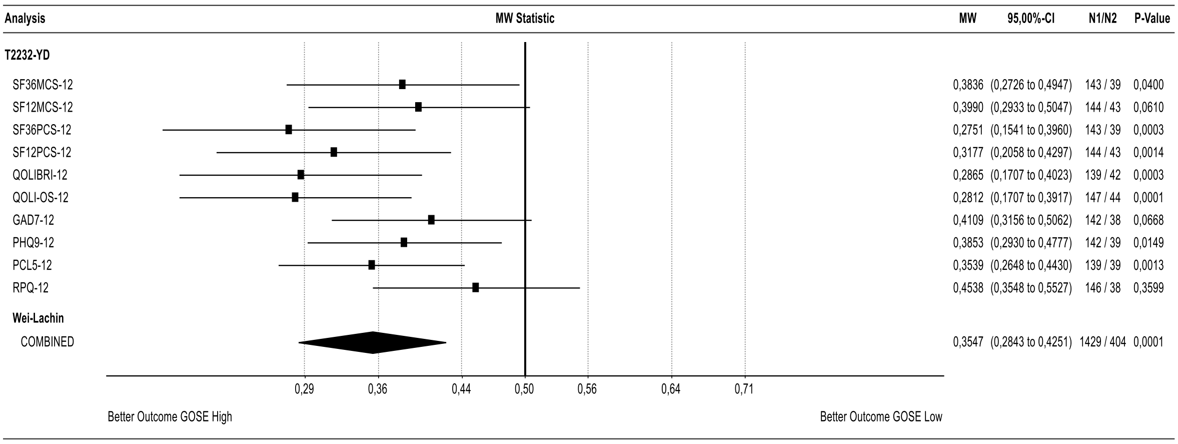

GOSE/-Q 7-8 vs. GOSE/-Q 3-4 - Admission

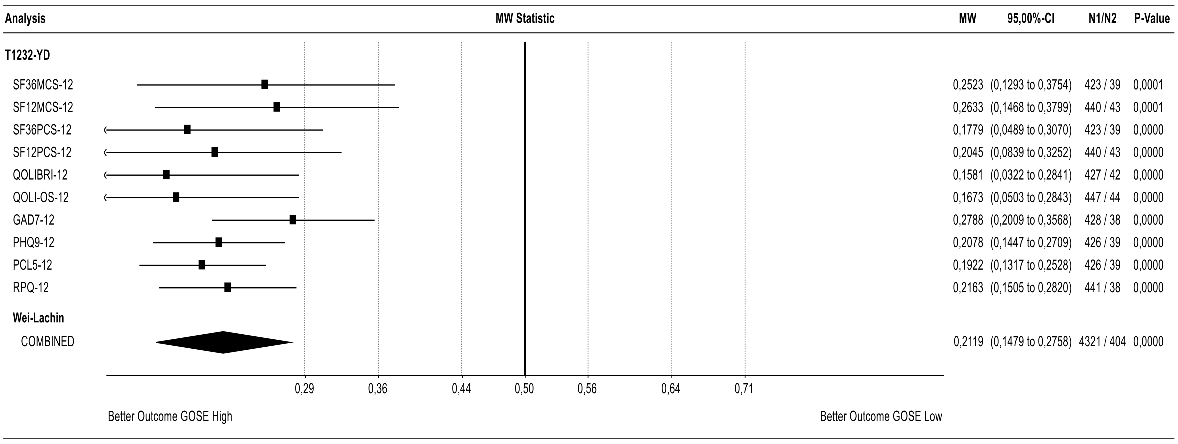

GOSE/-Q 7-8 vs. GOSE/-Q 5-6- ICU

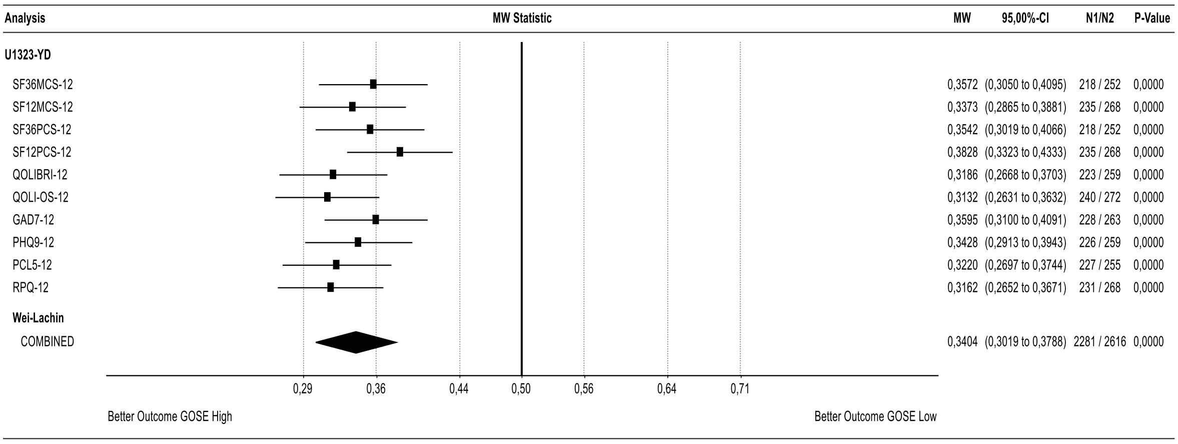

GOSE/-Q 5-6 vs. GOSE/-Q 3-4 - ICU

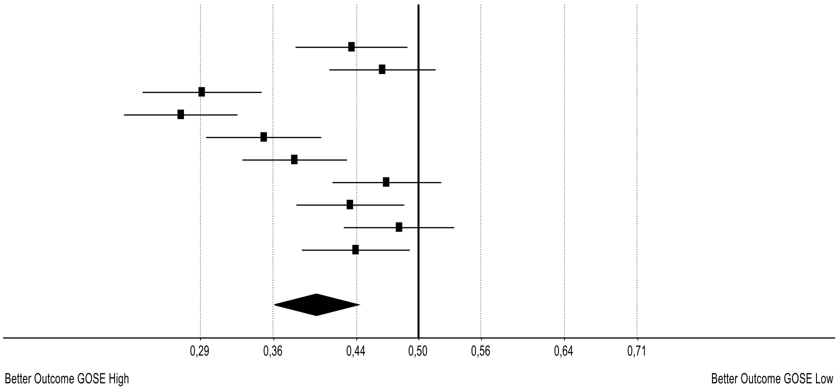

Analysis	MW	95,00%-CI	N1/N2	P-Value
**U2333-YD**				
SF36MCS-12	0,4354	(0,3813 to 0,4894)	252 / 201	0,0191
SF12MCS-12	0,4654	(0,4142 to 0,5165)	268 / 228	0,1840
SF36PCS-12	0,2915	(0,2344 to 0,3486)	252 / 201	0,0000
SF12PCS-12	0,2712	(0,2168 to 0,3255)	268 / 228	0,0000
QOLIBRI-12	0,3511	(0,2958 to 0,4063)	259 / 201	0,0000
QOLI-OS-12	0,3804	(0,3301 to 0,4308)	272 / 246	0,0000
GAD7-12	0,4693	(0,4173 to 0,5213)	263 / 203	0,2474
PHQ9-12	0,4343	(0,3823 to 0,4863)	259 / 202	0,0133
PCL5-12	0,4813	(0,4283 to 0,5344)	255 / 200	0,4909
RPQ-12	0,4395	(0,3879 to 0,4912)	268 / 208	0,0217
**Wei-Lachin**				
COMBINED	0,4019	(0,3617 to 0,4421)	2616 / 2118	0,0000

GOSE/-Q 7-8 vs. GOSE/-Q 3-4 - ICU

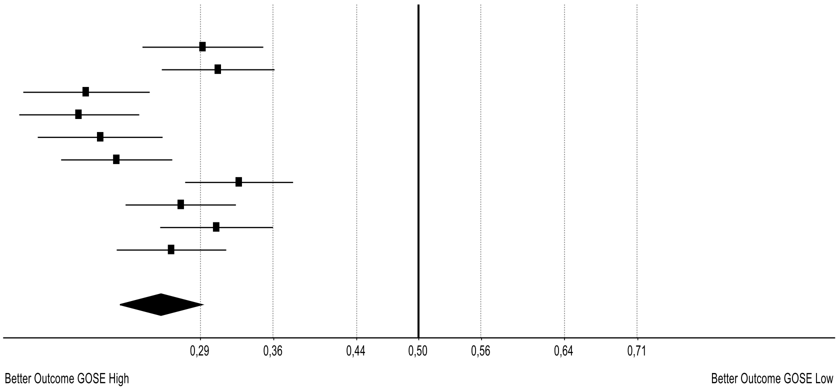

Analysis	MW	95,00%-CI	N1/N2	P-Value
**U1333-YD**				
SF36MCS-12	0,2923	(0,2343 to 0,3502)	218 / 201	0,0000
SF12MCS-12	0,3069	(0,2526 to 0,3613)	235 / 228	0,0000
SF36PCS-12	0,1805	(0,1195 to 0,2415)	218 / 201	0,0000
SF12PCS-12	0,1734	(0,1158 to 0,2309)	235 / 228	0,0000
QOLIBRI-12	0,1938	(0,1340 to 0,2536)	223 / 201	0,0000
QOLI-OS-12	0,2096	(0,1564 to 0,2628)	240 / 246	0,0000
GAD7-12	0,3274	(0,2756 to 0,3792)	228 / 203	0,0000
PHQ9-12	0,2714	(0,2183 to 0,3245)	226 / 202	0,0000
PCL5-12	0,3055	(0,2511 to 0,3598)	227 / 200	0,0000
RPQ-12	0,2622	(0,2095 to 0,3148)	231 / 208	0,0000
**Wei-Lachin**				
COMBINED	0,2523	(0,2126 to 0,2920)	2281 / 2118	0,0000

<u>3 months after TBI (completers)</u>

GOSE/-Q 7-8 vs. GOSE/-Q 5-6- Admission

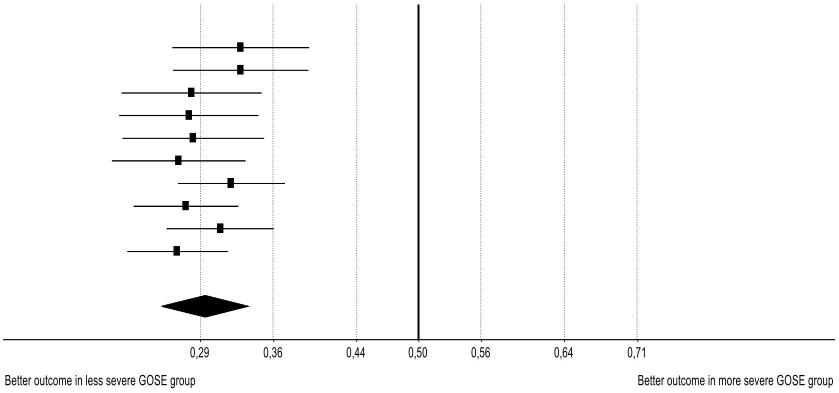

Analysis	MW	95,00%-CI	N1/N2	P-Value
**T1222-3X**				
SF36MCS-X	0,3290	(0,2630 to 0,3949)	350 / 130	0,0000
SF12MCS-X	0,3287	(0,2635 to 0,3939)	377 / 132	0,0000
SF36PCS-X	0,2814	(0,2139 to 0,3489)	350 / 130	0,0000
SF12PCS-X	0,2791	(0,2121 to 0,3462)	377 / 132	0,0000
QOLIBRI-X	0,2832	(0,2150 to 0,3513)	357 / 126	0,0000
QOLI-OS-X	0,2694	(0,2049 to 0,3338)	387 / 135	0,0000
GAD7-X3	0,3197	(0,2682 to 0,3712)	355 / 128	0,0000
PHQ9-X3	0,2763	(0,2256 to 0,3269)	355 / 129	0,0000
PCL5-X3	0,3091	(0,2573 to 0,3608)	353 / 127	0,0000
RPQ-X3	0,2679	(0,2195 to 0,3162)	375 / 136	0,0000
**Wei-Lachin**				
COMBINED	0,2944	(0,2530 to 0,3358)	3636 / 1305	0,0000

GOSE/-Q 5-6 vs. GOSE/-Q 3-4 - Admission

Analysis	MW	95,00%-CI	N1/N2	P-Value
**T2232-3X**				
SF36MCS-X	0,3248	(0,1950 to 0,4546)	130 / 31	0,0082
SF12MCS-X	0,3207	(0,1945 to 0,4470)	132 / 33	0,0054
SF36PCS-X	0,2507	(0,1122 to 0,3892)	130 / 31	0,0004
SF12PCS-X	0,3025	(0,1741 to 0,4308)	132 / 33	0,0025
QOLIBRI-X	0,2275	(0,0947 to 0,3603)	126 / 33	0,0001
QOLI-OS-X	0,1962	(0,0660 to 0,3264)	135 / 33	0,0000
GAD7-X3	0,3852	(0,2810 to 0,4895)	128 / 29	0,0310
PHQ9-X3	0,3645	(0,2617 to 0,4672)	129 / 30	0,0097
PCL5-X3	0,3677	(0,2661 to 0,4693)	127 / 30	0,0107
RPQ-X3	0,3570	(0,2582 to 0,4558)	136 / 29	0,0046
**Wei-Lachin**				
COMBINED	0,3097	(0,2373 to 0,3821)	1305 / 312	0,0000

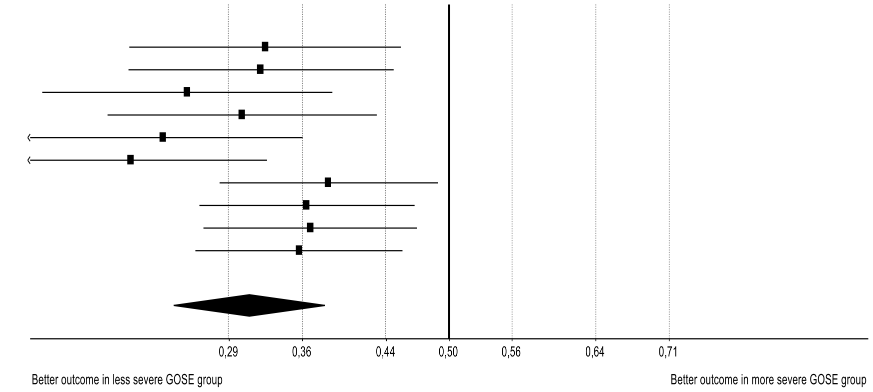

GOSE/-Q 7-8 vs. GOSE/-Q 3-4 - Admission

Analysis	MW	95,00%-CI	N1/N2	P-Value
**T1232-3X**				
SF36MCS-X	0,1870	(0,0418 to 0,3322)	350 / 31	0,0000
SF12MCS-X	0,1794	(0,0395 to 0,3194)	377 / 33	0,0000
SF36PCS-X	0,1283	(0,0000 to 0,2795)	350 / 31	0,0000
SF12PCS-X	0,1479	(0,0037 to 0,2920)	377 / 33	0,0000
QOLIBRI-X	0,0798	(0,0000 to 0,2279)	357 / 33	0,0000
QOLI-OS-X	0,0635	(0,0000 to 0,2042)	387 / 33	0,0000
GAD7-X3	0,2310	(0,1515 to 0,3105)	355 / 29	0,0000
PHQ9-X3	0,1904	(0,1180 to 0,2628)	355 / 30	0,0000
PCL5-X3	0,2145	(0,1419 to 0,2872)	353 / 30	0,0000
RPQ-X3	0,1590	(0,0989 to 0,2191)	375 / 29	0,0000
**Wei-Lachin**				
COMBINED	0,1581	(0,0931 to 0,2231)	3636 / 312	0,0000

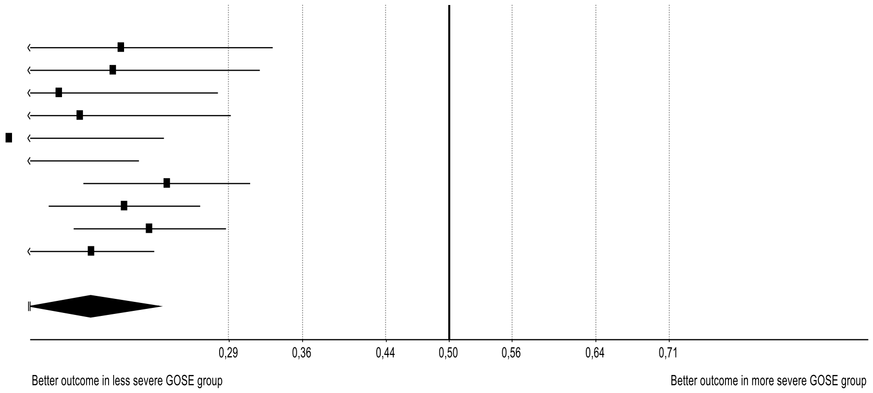

GOSE/-Q 7-8 vs. GOSE/-Q 5-6- ICU

Analysis	MW	95,00%-CI	N1/N2	P-Value
**U1323-3X**				
SF36MCS-X	0,3669	(0,3087 to 0,4252)	180 / 199	0,0000
SF12MCS-X	0,3666	(0,3123 to 0,4209)	207 / 230	0,0000
SF36PCS-X	0,2710	(0,2113 to 0,3306)	180 / 199	0,0000
SF12PCS-X	0,2813	(0,2259 to 0,3366)	207 / 230	0,0000
QOLIBRI-X	0,3242	(0,2674 to 0,3811)	188 / 212	0,0000
QOLI-OS-X	0,2841	(0,2307 to 0,3374)	214 / 239	0,0000
GAD7-X3	0,3590	(0,3042 to 0,4139)	187 / 218	0,0000
PHQ9-X3	0,3294	(0,2725 to 0,3863)	184 / 218	0,0000
PCL5-X3	0,3403	(0,2829 to 0,3977)	191 / 210	0,0000
RPQ-X3	0,2800	(0,2245 to 0,3355)	193 / 226	0,0000
**Wei-Lachin**				
COMBINED	0,3203	(0,2810 to 0,3596)	1931 / 2181	0,0000

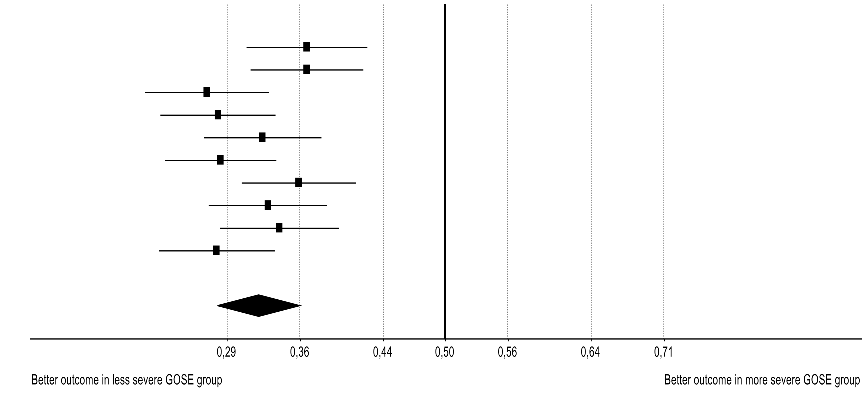

GOSE/-Q 5-6 vs. GOSE/-Q 3-4 - ICU

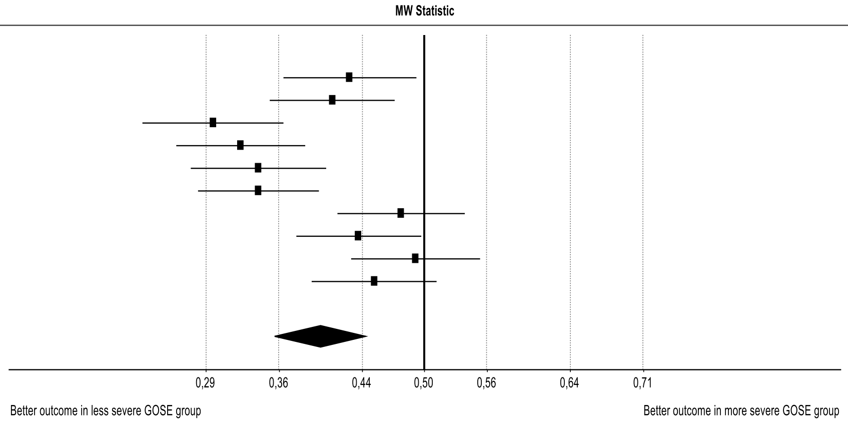

Analysis	MW	95,00%-CI	N1/N2	P-Value
**U2333-3X**				
SF36MCS-X	0,4283	(0,3643 to 0,4924)	199 / 139	0,0284
SF12MCS-X	0,4114	(0,3516 to 0,4711)	230 / 162	0,0037
SF36PCS-X	0,2969	(0,2292 to 0,3646)	199 / 139	0,0000
SF12PCS-X	0,3233	(0,2615 to 0,3851)	230 / 162	0,0000
QOLIBRI-X	0,3405	(0,2755 to 0,4055)	212 / 142	0,0000
QOLI-OS-X	0,3402	(0,2822 to 0,3982)	239 / 180	0,0000
GAD7-X3	0,4775	(0,4166 to 0,5384)	218 / 136	0,4685
PHQ9-X3	0,4368	(0,3766 to 0,4971)	218 / 138	0,0399
PCL5-X3	0,4913	(0,4295 to 0,5531)	210 / 134	0,7825
RPQ-X3	0,4516	(0,3919 to 0,5113)	226 / 140	0,1124
**Wei-Lachin**				
COMBINED	0,3998	(0,3562 to 0,4434)	2181 / 1472	0,0000

GOSE/-Q 7-8 vs. GOSE/-Q 3-4 - ICU

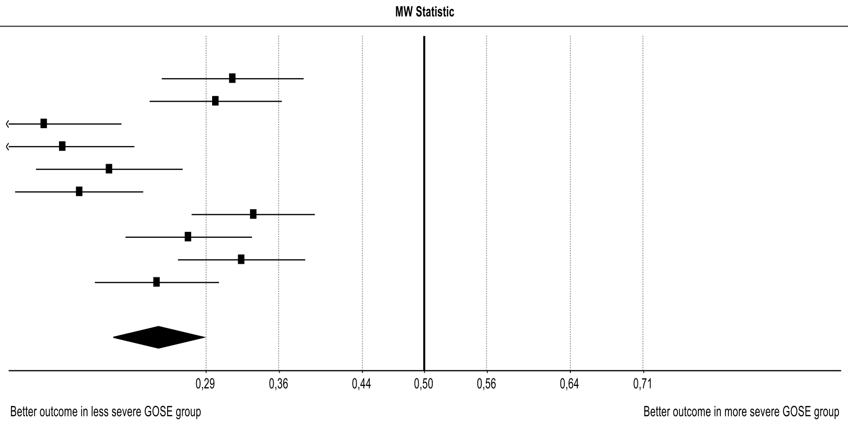

Analysis	MW	95,00%-CI	N1/N2	P-Value
**U1333-3X**				
SF36MCS-X	0,3159	(0,2478 to 0,3841)	180 / 139	0,0000
SF12MCS-X	0,2995	(0,2358 to 0,3631)	207 / 162	0,0000
SF36PCS-X	0,1344	(0,0599 to 0,2090)	180 / 139	0,0000
SF12PCS-X	0,1525	(0,0840 to 0,2209)	207 / 162	0,0000
QOLIBRI-X	0,1972	(0,1269 to 0,2676)	188 / 142	0,0000
QOLI-OS-X	0,1684	(0,1067 to 0,2301)	214 / 180	0,0000
GAD7-X3	0,3356	(0,2762 to 0,3949)	187 / 136	0,0000
PHQ9-X3	0,2734	(0,2125 to 0,3342)	184 / 138	0,0000
PCL5-X3	0,3243	(0,2630 to 0,3855)	191 / 134	0,0000
RPQ-X3	0,2428	(0,1832 to 0,3023)	193 / 140	0,0000
**Wei-Lachin**				
COMBINED	0,2444	(0,2009 to 0,2879)	1931 / 1472	0,0000

<u>6 months after TBI (completers)</u>

GOSE/-Q 7-8 vs. GOSE/-Q 5-6- Admission

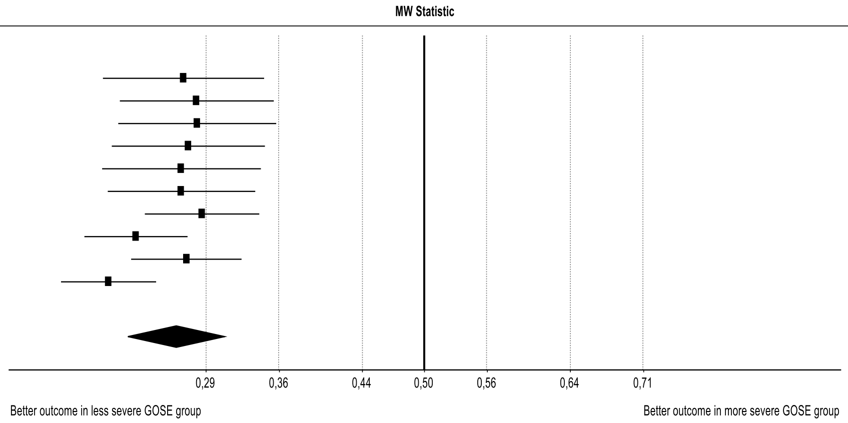

Analysis	MW	95,00%-CI	N1/N2	P-Value
**T1222-6X**				
SF36MCS-X	0,2685	(0,1910 to 0,3460)	387 / 100	0,0000
SF12MCS-X	0,2811	(0,2071 to 0,3550)	409 / 108	0,0000
SF36PCS-X	0,2814	(0,2055 to 0,3574)	387 / 100	0,0000
SF12PCS-X	0,2733	(0,1996 to 0,3470)	409 / 108	0,0000
QOLIBRI-X	0,2664	(0,1902 to 0,3427)	389 / 102	0,0000
QOLI-OS-X	0,2664	(0,1958 to 0,3370)	418 / 113	0,0000
GAD7-X6	0,2864	(0,2316 to 0,3412)	389 / 100	0,0000
PHQ9-X6	0,2231	(0,1735 to 0,2726)	390 / 101	0,0000
PCL5-X6	0,2713	(0,2184 to 0,3242)	388 / 100	0,0000
RPQ-X6	0,1963	(0,1505 to 0,2420)	412 / 106	0,0000
**Wei-Lachin**				
COMBINED	0,2614	(0,2147 to 0,3082)	3978 / 1038	0,0000

GOSE/-Q 7-8 vs. GOSE/-Q 5-6- ICU

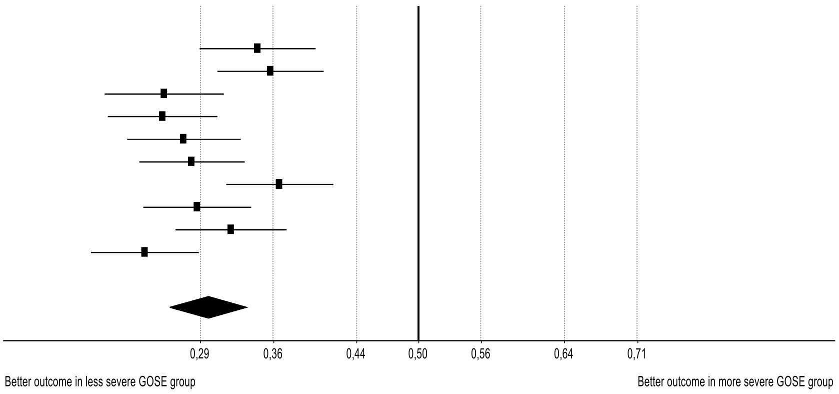

Analysis	MW	95,00%-CI	N1/N2	P-Value
**U1323-6X**				
SF36MCS-X	0,3451	(0,2895 to 0,4007)	226 / 211	0,0000
SF12MCS-X	0,3577	(0,3066 to 0,4089)	256 / 250	0,0000
SF36PCS-X	0,2555	(0,1981 to 0,3129)	226 / 211	0,0000
SF12PCS-X	0,2537	(0,2007 to 0,3067)	256 / 250	0,0000
QOLIBRI-X	0,2739	(0,2193 to 0,3286)	233 / 228	0,0000
QOLI-OS-X	0,2818	(0,2311 to 0,3325)	267 / 256	0,0000
GAD7-X6	0,3664	(0,3151 to 0,4177)	233 / 229	0,0000
PHQ9-X6	0,2872	(0,2353 to 0,3390)	231 / 230	0,0000
PCL5-X6	0,3194	(0,2662 to 0,3727)	232 / 224	0,0000
RPQ-X6	0,2367	(0,1847 to 0,2887)	240 / 236	0,0000
**Wei-Lachin**				
COMBINED	0,2978	(0,2609 to 0,3346)	2400 / 2325	0,0000

GOSE/-Q 5-6 vs. GOSE/-Q 3-4 - ICU

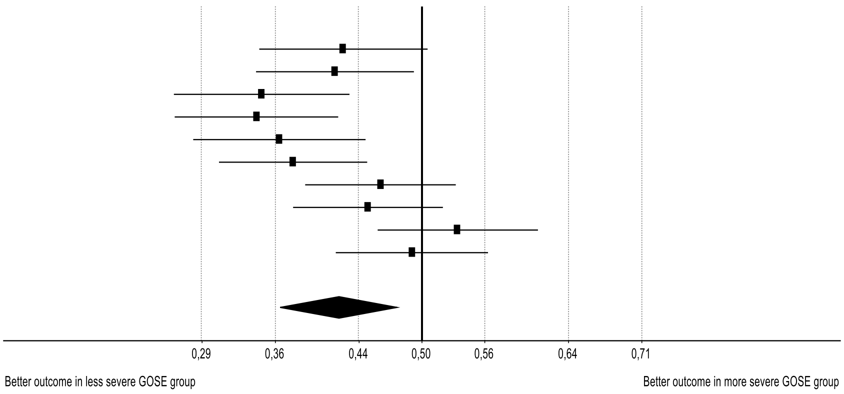

Analysis	MW	95,00%-CI	N1/N2	P-Value
**U2333-6X**				
SF36MCS-X	0,4252	(0,3450 to 0,5054)	211 / 77	0,0676
SF12MCS-X	0,4171	(0,3419 to 0,4924)	250 / 89	0,0309
SF36PCS-X	0,3474	(0,2634 to 0,4315)	211 / 77	0,0004
SF12PCS-X	0,3424	(0,2640 to 0,4207)	250 / 89	0,0001
QOLIBRI-X	0,3642	(0,2817 to 0,4467)	228 / 77	0,0013
QOLI-OS-X	0,3773	(0,3063 to 0,4483)	256 / 105	0,0007
GAD7-X6	0,4608	(0,3890 to 0,5326)	229 / 76	0,2845
PHQ9-X6	0,4488	(0,3771 to 0,5204)	230 / 78	0,1611
PCL5-X6	0,5345	(0,4583 to 0,6108)	224 / 77	0,3748
RPQ-X6	0,4908	(0,4178 to 0,5638)	236 / 80	0,8040
**Wei-Lachin**				
COMBINED	0,4209	(0,3649 to 0,4768)	2325 / 825	0,0056

GOSE/-Q 7-8 vs. GOSE/-Q 3-4 - ICU

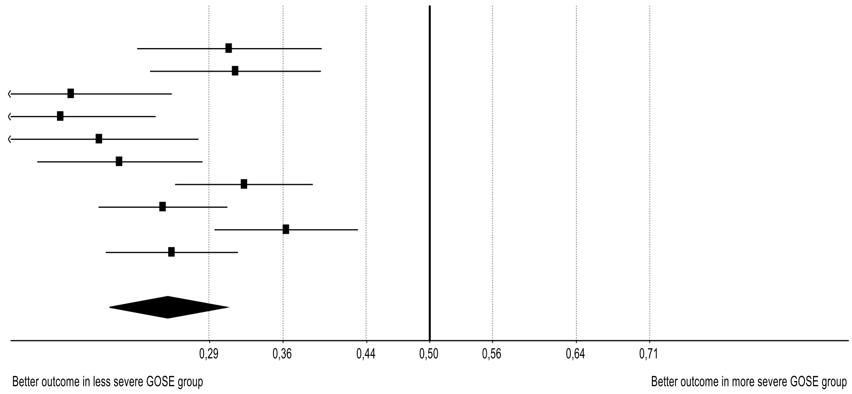

Analysis	MW	95,00%-CI	N1/N2	P-Value
**U1333-6X**				
SF36MCS-X	0,3090	(0,2211 to 0,3970)	226 / 77	0,0000
SF12MCS-X	0,3148	(0,2331 to 0,3964)	256 / 89	0,0000
SF36PCS-X	0,1577	(0,0610 to 0,2544)	226 / 77	0,0000
SF12PCS-X	0,1480	(0,0571 to 0,2389)	256 / 89	0,0000
QOLIBRI-X	0,1853	(0,0912 to 0,2794)	233 / 77	0,0000
QOLI-OS-X	0,2044	(0,1257 to 0,2831)	267 / 105	0,0000
GAD7-X6	0,3231	(0,2575 to 0,3888)	233 / 76	0,0000
PHQ9-X6	0,2458	(0,1839 to 0,3076)	231 / 78	0,0000
PCL5-X6	0,3636	(0,2952 to 0,4319)	232 / 77	0,0001
RPQ-X6	0,2542	(0,1910 to 0,3174)	240 / 80	0,0000
**Wei-Lachin**				
COMBINED	0,2506	(0,1949 to 0,3062)	2400 / 825	0,0000

12 months after TBI (completers)

GOSE/-Q 7-8 vs. GOSE/-Q 5-6- Admission

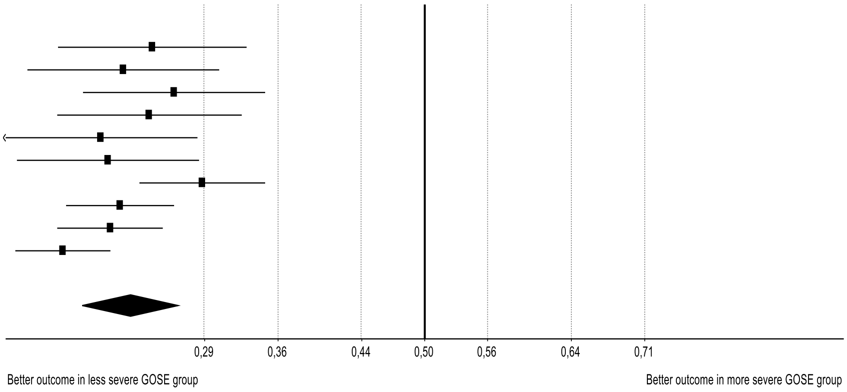

Analysis	MW	95,00%-CI	N1/N2	P-Value
**T1222-YX**				
SF36MCSX1	0,2405	(0,1503 to 0,3307)	401 / 76	0,0000
SF12MCSX1	0,2129	(0,1212 to 0,3045)	426 / 77	0,0000
SF36PCSX1	0,2613	(0,1742 to 0,3483)	401 / 76	0,0000
SF12PCSX1	0,2376	(0,1493 to 0,3258)	426 / 77	0,0000
QOLIBRIX1	0,1908	(0,0984 to 0,2831)	406 / 74	0,0000
QOLI-OSX1	0,1982	(0,1113 to 0,2851)	441 / 77	0,0000
GAD7-X12	0,2880	(0,2277 to 0,3483)	402 / 75	0,0000
PHQ9-X12	0,2097	(0,1581 to 0,2614)	404 / 75	0,0000
PCL5-X12	0,2001	(0,1498 to 0,2504)	405 / 72	0,0000
RPQ-X12	0,1549	(0,1096 to 0,2002)	429 / 77	0,0000
**Wei-Lachin**				
COMBINED	0,2194	(0,1736 to 0,2651)	4141 / 756	0,0000

GOSE/-Q 7-8 vs. GOSE/-Q 5-6- ICU

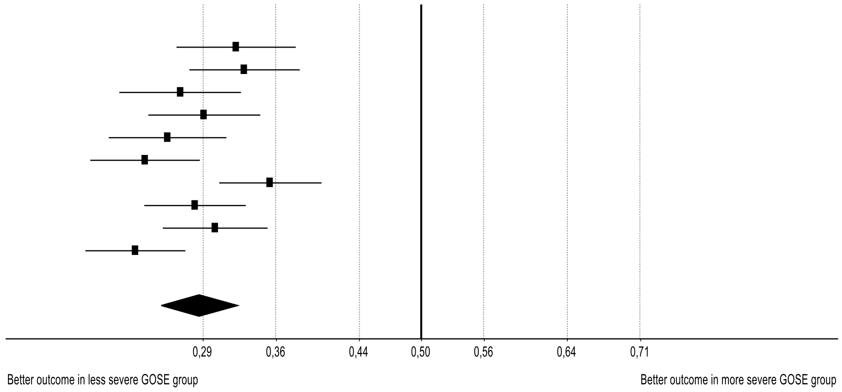

Analysis	MW	95,00%-CI	N1/N2	P-Value
**U1323-YX**				
SF36MCSX1	0,3219	(0,2649 to 0,3790)	267 / 193	0,0000
SF12MCSX1	0,3299	(0,2769 to 0,3829)	307 / 224	0,0000
SF36PCSX1	0,2681	(0,2094 to 0,3268)	267 / 193	0,0000
SF12PCSX1	0,2911	(0,2372 to 0,3450)	307 / 224	0,0000
QOLIBRIX1	0,2559	(0,1992 to 0,3126)	283 / 203	0,0000
QOLI-OSX1	0,2344	(0,1817 to 0,2872)	322 / 231	0,0000
GAD7-X12	0,3547	(0,3053 to 0,4040)	280 / 205	0,0000
PHQ9-X12	0,2824	(0,2333 to 0,3314)	280 / 206	0,0000
PCL5-X12	0,3016	(0,2512 to 0,3521)	283 / 198	0,0000
RPQ-X12	0,2253	(0,1773 to 0,2733)	291 / 210	0,0000
**Wei-Lachin**				
COMBINED	0,2865	(0,2495 to 0,3235)	2887 / 2087	0,0000

GOSE/-Q 5-6 vs. GOSE/-Q 3-4 - ICU

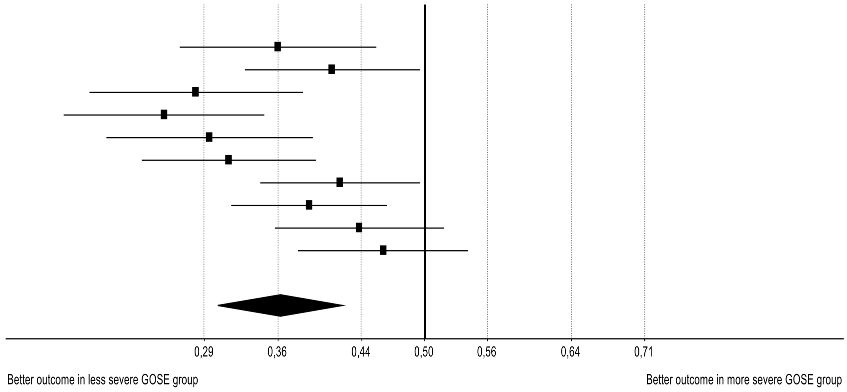

Analysis	MW	95,00%-CI	N1/N2	P-Value
**U2333-YX**				
SF36MCSX1	0,3602	(0,2664 to 0,4541)	193 / 56	0,0035
SF12MCSX1	0,4123	(0,3292 to 0,4955)	224 / 67	0,0389
SF36PCSX1	0,2823	(0,1804 to 0,3842)	193 / 56	0,0000
SF12PCSX1	0,2517	(0,1559 to 0,3475)	224 / 67	0,0000
QOLIBRIX1	0,2951	(0,1967 to 0,3936)	203 / 57	0,0000
QOLI-OSX1	0,3133	(0,2301 to 0,3966)	231 / 79	0,0000
GAD7-X12	0,4195	(0,3433 to 0,4956)	205 / 55	0,0382
PHQ9-X12	0,3900	(0,3155 to 0,4645)	206 / 55	0,0038
PCL5-X12	0,4383	(0,3576 to 0,5190)	198 / 54	0,1343
RPQ-X12	0,4609	(0,3797 to 0,5420)	210 / 58	0,3447
**Wei-Lachin**				
COMBINED	0,3624	(0,3024 to 0,4223)	2087 / 604	0,0000

GOSE/-Q 7-8 vs. GOSE/-Q 3-4 - ICU

Analysis	MW	95,00%-CI	N1/N2	P-Value
**U1333-YX**				
SF36MCSX1	0,1836	(0,0743 to 0,2928)	267 / 56	0,0000
SF12MCSX1	0,2345	(0,1384 to 0,3306)	307 / 67	0,0000
SF36PCSX1	0,1186	(0,0025 to 0,2347)	267 / 56	0,0000
SF12PCSX1	0,1087	(0,0018 to 0,2157)	307 / 67	0,0000
QOLIBRIX1	0,1244	(0,0129 to 0,2358)	283 / 57	0,0000
QOLI-OSX1	0,1152	(0,0215 to 0,2088)	322 / 79	0,0000
GAD7-X12	0,2565	(0,1980 to 0,3150)	280 / 55	0,0000
PHQ9-X12	0,1717	(0,1181 to 0,2254)	280 / 55	0,0000
PCL5-X12	0,2331	(0,1721 to 0,2941)	283 / 54	0,0000
RPQ-X12	0,2056	(0,1466 to 0,2646)	291 / 58	0,0000
**Wei-Lachin**				
COMBINED	0,1752	(0,1190 to 0,2314)	2887 / 604	0,0000

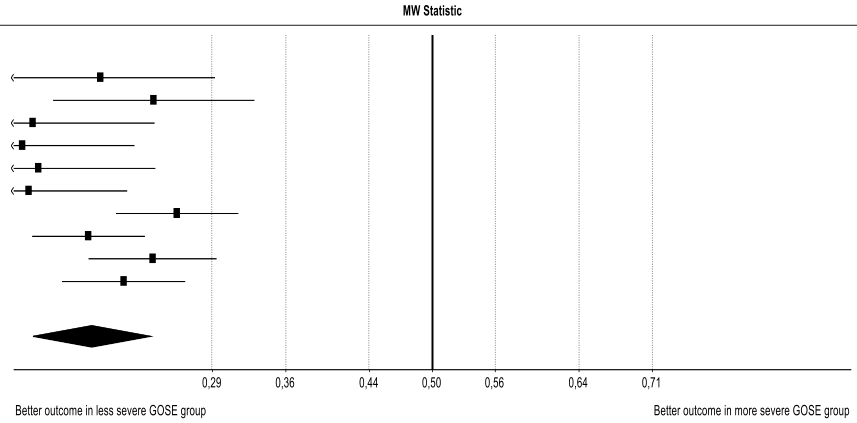

Premorbid psychiatric disturbances

3 months after TBI (data as available)

GOSE/-Q 7-8 vs. GOSE/-Q 5-6 - Absent

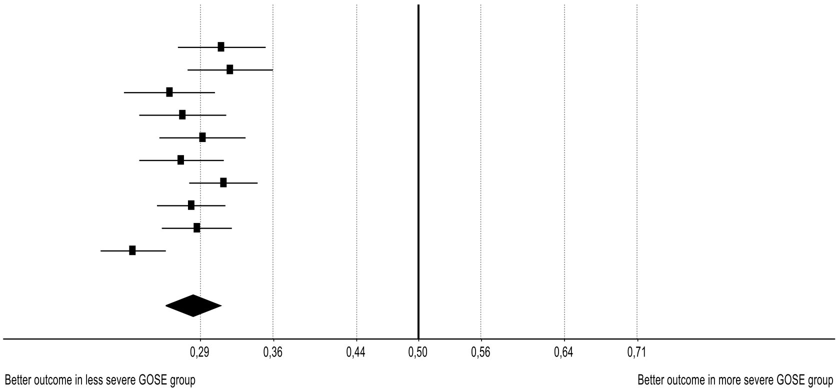

Analysis	MW	95,00%-CI	N1/N2	P-Value
**X1121-3D**				
SF36MCS-3	0,3106	(0,2681 to 0,3531)	751 / 333	0,0000
SF12MCS-3	0,3188	(0,2780 to 0,3597)	796 / 356	0,0000
SF36PCS-3	0,2604	(0,2168 to 0,3040)	751 / 333	0,0000
SF12PCS-3	0,2730	(0,2312 to 0,3148)	796 / 356	0,0000
QOLIBRI-3	0,2921	(0,2504 to 0,3337)	756 / 344	0,0000
QOLI-OS-3	0,2717	(0,2311 to 0,3123)	798 / 359	0,0000
GAD7-3	0,3124	(0,2793 to 0,3454)	748 / 343	0,0000
PHQ9-3	0,2815	(0,2485 to 0,3145)	750 / 344	0,0000
PCL5-3	0,2867	(0,2532 to 0,3201)	752 / 339	0,0000
RPQ-3	0,2254	(0,1941 to 0,2567)	766 / 349	0,0000
**Wei-Lachin**				
COMBINED	0,2833	(0,2568 to 0,3097)	7664 / 3456	0,0000

GOSE/-Q 5-6 vs. GOSE/-Q 3-4 - Absent

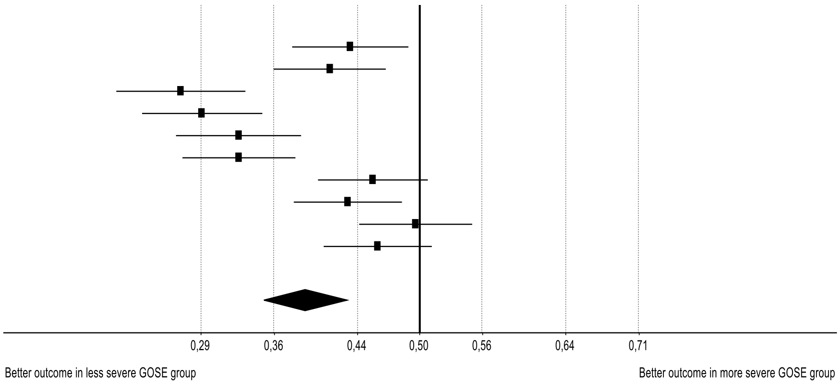

Analysis	MW	95,00%-CI	N1/N2	P-Value
**X2131-3D**				
SF36MCS-3	0,4332	(0,3774 to 0,4890)	333 / 165	0,0189
SF12MCS-3	0,4137	(0,3600 to 0,4674)	356 / 184	0,0016
SF36PCS-3	0,2707	(0,2087 to 0,3327)	333 / 165	0,0000
SF12PCS-3	0,2910	(0,2333 to 0,3487)	356 / 184	0,0000
QOLIBRI-3	0,3262	(0,2665 to 0,3858)	344 / 161	0,0000
QOLI-OS-3	0,3265	(0,2727 to 0,3804)	359 / 199	0,0000
GAD7-3	0,4549	(0,4023 to 0,5074)	343 / 163	0,0924
PHQ9-3	0,4308	(0,3788 to 0,4828)	344 / 165	0,0091
PCL5-3	0,4962	(0,4423 to 0,5502)	339 / 158	0,8913
RPQ-3	0,4597	(0,4077 to 0,5117)	349 / 167	0,1285
**Wei-Lachin**				
COMBINED	0,3903	(0,3504 to 0,4301)	3456 / 1711	0,0000

GOSE/-Q 7-8 vs. GOSE/-Q 3-4 - Absent

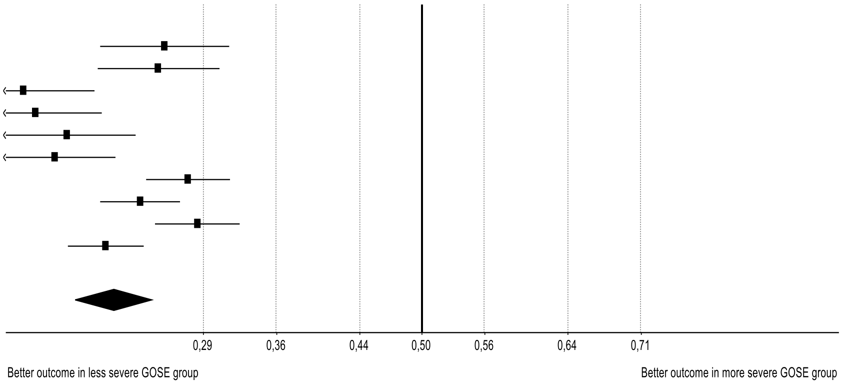

Analysis	MW	95,00%-CI	N1/N2	P-Value
**X1131-3D**				
SF36MCS-3	0,2529	(0,1910 to 0,3148)	751 / 165	0,0000
SF12MCS-3	0,2469	(0,1883 to 0,3056)	796 / 184	0,0000
SF36PCS-3	0,1171	(0,0487 to 0,1855)	751 / 165	0,0000
SF12PCS-3	0,1288	(0,0648 to 0,1928)	796 / 184	0,0000
QOLIBRI-3	0,1590	(0,0933 to 0,2248)	756 / 161	0,0000
QOLI-OS-3	0,1475	(0,0894 to 0,2056)	798 / 199	0,0000
GAD7-3	0,2753	(0,2350 to 0,3156)	748 / 163	0,0000
PHQ9-3	0,2295	(0,1912 to 0,2677)	750 / 165	0,0000
PCL5-3	0,2844	(0,2438 to 0,3251)	752 / 158	0,0000
RPQ-3	0,1964	(0,1602 to 0,2325)	766 / 167	0,0000
**Wei-Lachin**				
COMBINED	0,2038	(0,1673 to 0,2403)	7664 / 1711	0,0000

GOSE/-Q 7-8 vs. GOSE/-Q 5-6- Present

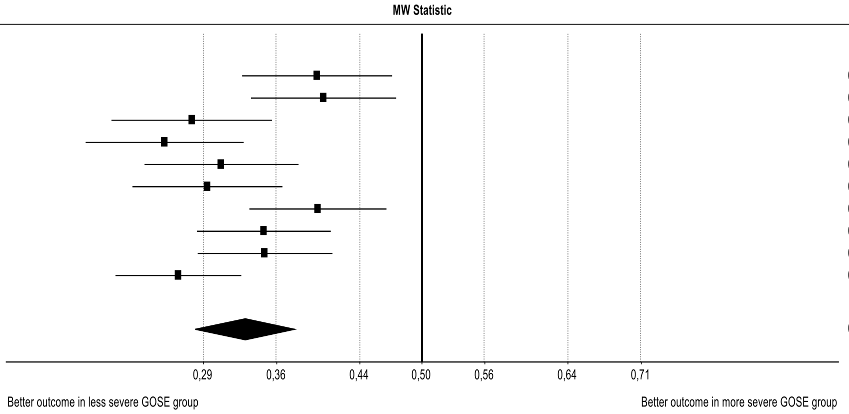

Analysis	MW	95,00%-CI	N1/N2	P-Value
**Y1222-3D**				
SF36MCS-3	0,3990	(0,3270 to 0,4710)	192 / 104	0,0060
SF12MCS-3	0,4055	(0,3361 to 0,4749)	199 / 112	0,0076
SF36PCS-3	0,2789	(0,2020 to 0,3559)	192 / 104	0,0000
SF12PCS-3	0,2531	(0,1772 to 0,3290)	199 / 112	0,0000
QOLIBRI-3	0,3074	(0,2335 to 0,3813)	194 / 108	0,0000
QOLI-OS-3	0,2941	(0,2221 to 0,3661)	196 / 113	0,0000
GAD7-3	0,4001	(0,3342 to 0,4660)	190 / 104	0,0030
PHQ9-3	0,3483	(0,2840 to 0,4125)	192 / 105	0,0000
PCL5-3	0,3493	(0,2845 to 0,4140)	193 / 105	0,0000
RPQ-3	0,2661	(0,2059 to 0,3263)	194 / 107	0,0000
**Wei-Lachin**				
COMBINED	0,3302	(0,2827 to 0,3776)	1941 / 1074	0,0000

GOSE/-Q 5-6 vs. GOSE/-Q 3-4 - Present

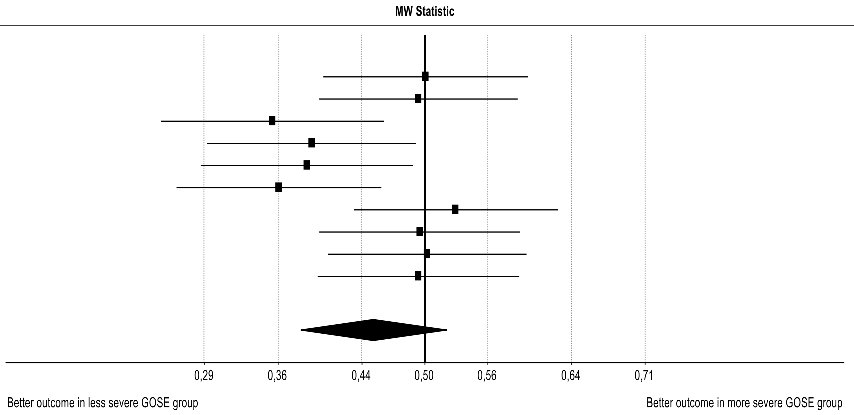

Analysis	MW	95,00%-CI	N1/N2	P-Value
**Y2232-3D**				
SF36MCS-3	0,5010	(0,4034 to 0,5986)	104 / 49	0,9843
SF12MCS-3	0,4943	(0,3995 to 0,5890)	112 / 53	0,9057
SF36PCS-3	0,3550	(0,2488 to 0,4612)	104 / 49	0,0075
SF12PCS-3	0,3924	(0,2927 to 0,4921)	112 / 53	0,0343
QOLIBRI-3	0,3877	(0,2869 to 0,4886)	108 / 52	0,0291
QOLI-OS-3	0,3612	(0,2634 to 0,4589)	113 / 58	0,0054
GAD7-3	0,5299	(0,4327 to 0,6271)	104 / 51	0,5467
PHQ9-3	0,4955	(0,3996 to 0,5914)	105 / 52	0,9269
PCL5-3	0,5026	(0,4078 to 0,5974)	105 / 53	0,9570
RPQ-3	0,4939	(0,3978 to 0,5900)	107 / 49	0,9009
**Wei-Lachin**				
COMBINED	0,4513	(0,3818 to 0,5209)	1074 / 519	0,1705

GOSE/-Q 7-8 vs. GOSE/-Q 3-4 - Present

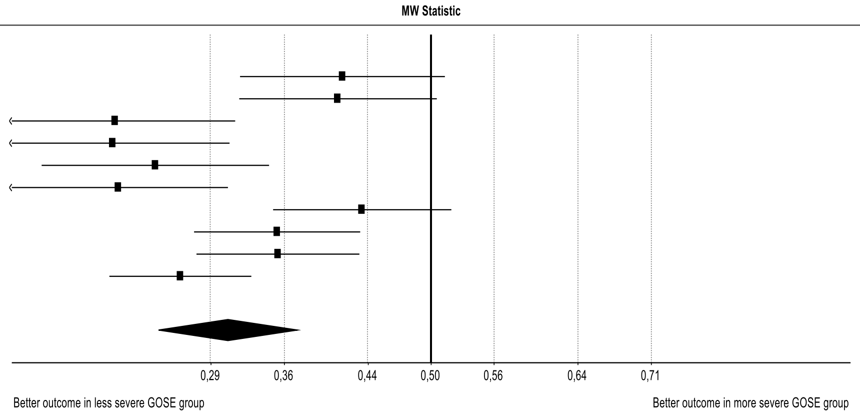

Analysis	MW	95,00%-CI	N1/N2	P-Value
**Y1232-3D**				
SF36MCS-3	0,4160	(0,3181 to 0,5138)	192 / 49	0,0924
SF12MCS-3	0,4115	(0,3171 to 0,5059)	199 / 53	0,0661
SF36PCS-3	0,1989	(0,0839 to 0,3138)	192 / 49	0,0000
SF12PCS-3	0,1965	(0,0851 to 0,3079)	199 / 53	0,0000
QOLIBRI-3	0,2370	(0,1286 to 0,3454)	194 / 52	0,0000
QOLI-OS-3	0,2021	(0,0978 to 0,3064)	196 / 58	0,0000
GAD7-3	0,4346	(0,3496 to 0,5195)	190 / 51	0,1313
PHQ9-3	0,3537	(0,2744 to 0,4329)	192 / 52	0,0003
PCL5-3	0,3542	(0,2765 to 0,4320)	193 / 53	0,0002
RPQ-3	0,2609	(0,1931 to 0,3288)	194 / 49	0,0000
**Wei-Lachin**				
COMBINED	0,3065	(0,2407 to 0,3724)	1941 / 519	0,0000

6 months after TBI (data as available)

GOSE/-Q 7-8 vs. GOSE/-Q 5-6 - Absent

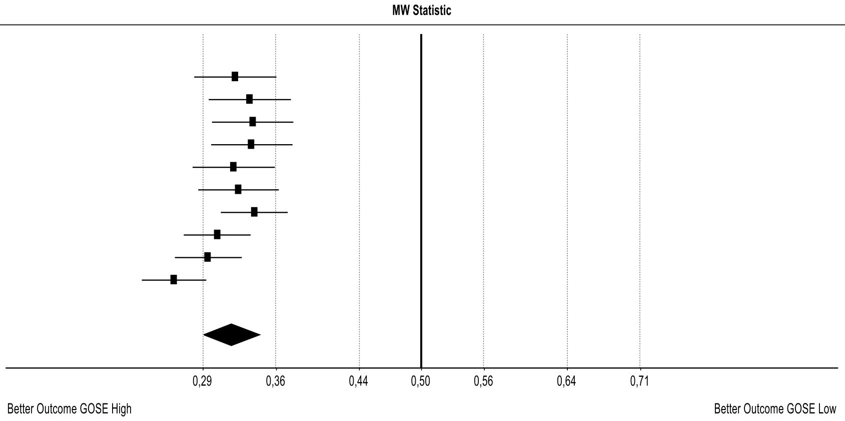

Analysis	MW	95,00%-CI	N1/N2	P-Value
**X1121-6D**				
SF36MCS-6	0,3212	(0,2815 to 0,3609)	812 / 372	0,0000
SF12MCS-6	0,3350	(0,2957 to 0,3742)	826 / 374	0,0000
SF36PCS-6	0,3379	(0,2987 to 0,3771)	811 / 372	0,0000
SF12PCS-6	0,3368	(0,2977 to 0,3760)	826 / 374	0,0000
QOLIBRI-6	0,3192	(0,2797 to 0,3587)	817 / 370	0,0000
QOLI-OS-6	0,3242	(0,2857 to 0,3626)	833 / 377	0,0000
GAD7-6	0,3395	(0,3072 to 0,3718)	814 / 370	0,0000
PHQ9-6	0,3038	(0,2716 to 0,3360)	816 / 372	0,0000
PCL5-6	0,2948	(0,2627 to 0,3270)	820 / 372	0,0000
RPQ-6	0,2619	(0,2309 to 0,2929)	821 / 375	0,0000
**Wei-Lachin**				
COMBINED	0,3174	(0,2906 to 0,3443)	8196 / 3728	0,0000

GOSE/-Q 5-6 vs. GOSE/-Q 3-4 – Absent

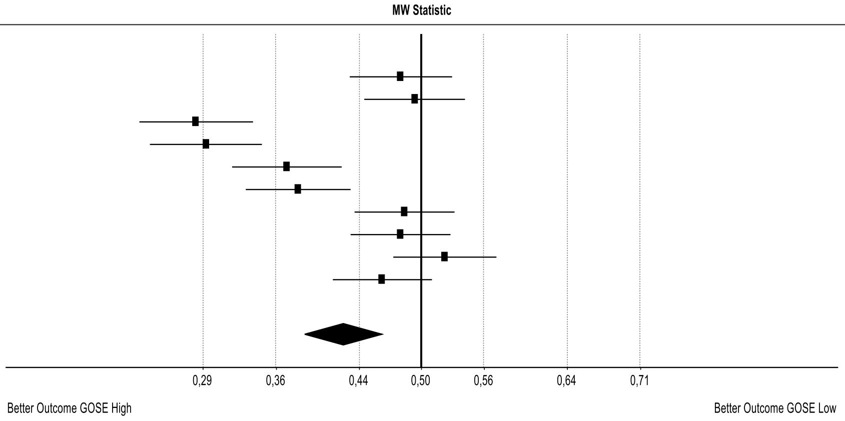

Analysis	MW	95,00%-CI	N1/N2	P-Value
**X2131-6D**				
SF36MCS-6	0,4801	(0,4309 to 0,5292)	372 / 214	0,4269
SF12MCS-6	0,4935	(0,4449 to 0,5420)	374 / 216	0,7921
SF36PCS-6	0,2834	(0,2289 to 0,3378)	372 / 214	0,0000
SF12PCS-6	0,2929	(0,2391 to 0,3467)	374 / 216	0,0000
QOLIBRI-6	0,3705	(0,3180 to 0,4230)	370 / 206	0,0000
QOLI-OS-6	0,3815	(0,3310 to 0,4319)	377 / 220	0,0000
GAD7-6	0,4835	(0,4356 to 0,5314)	370 / 210	0,4999
PHQ9-6	0,4796	(0,4315 to 0,5277)	372 / 209	0,4061
PCL5-6	0,5223	(0,4727 to 0,5720)	372 / 203	0,3773
RPQ-6	0,4624	(0,4149 to 0,5099)	375 / 214	0,1208
**Wei-Lachin**				
COMBINED	0,4250	(0,3876 to 0,4623)	3728 / 2122	0,0001

GOSE/-Q 7-8 vs. GOSE/-Q 3-4 - Absent

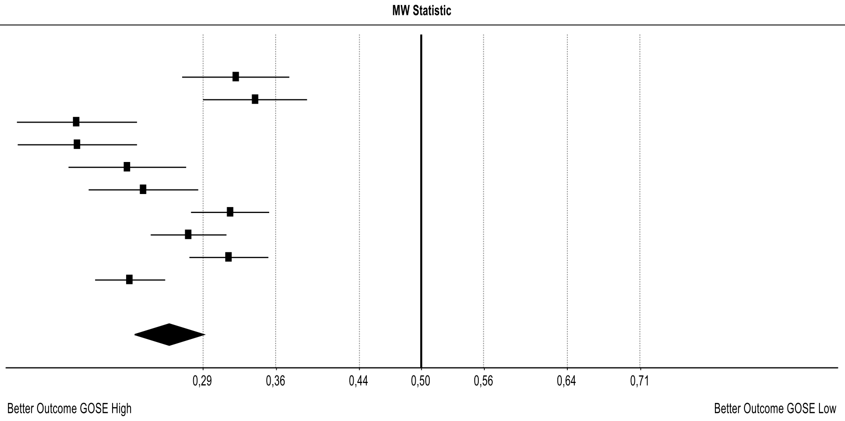

Analysis	MW	95,00%-CI	N1/N2	P-Value
**X1131-6D**				
SF36MCS-6	0,3215	(0,2699 to 0,3732)	812 / 214	0,0000
SF12MCS-6	0,3401	(0,2899 to 0,3903)	826 / 216	0,0000
SF36PCS-6	0,1686	(0,1109 to 0,2264)	811 / 214	0,0000
SF12PCS-6	0,1695	(0,1120 to 0,2270)	826 / 216	0,0000
QOLIBRI-6	0,2173	(0,1610 to 0,2735)	817 / 206	0,0000
QOLI-OS-6	0,2332	(0,1805 to 0,2858)	833 / 220	0,0000
GAD7-6	0,3162	(0,2787 to 0,3538)	814 / 210	0,0000
PHQ9-6	0,2761	(0,2396 to 0,3126)	816 / 209	0,0000
PCL5-6	0,3150	(0,2767 to 0,3532)	820 / 203	0,0000
RPQ-6	0,2198	(0,1861 to 0,2535)	821 / 214	0,0000
**Wei-Lachin**				
COMBINED	0,2577	(0,2243 to 0,2912)	8196 / 2122	0,0000

GOSE/-Q 7-8 vs. GOSE/-Q 5-6- Present

Analysis	MW	95,00%-CI	N1/N2	P-Value
**Y1222-6D**				
SF36MCS-6	0,3648	(0,2968 to 0,4329)	203 / 124	0,0001
SF12MCS-6	0,3842	(0,3175 to 0,4509)	206 / 126	0,0007
SF36PCS-6	0,2879	(0,2169 to 0,3589)	203 / 124	0,0000
SF12PCS-6	0,2979	(0,2279 to 0,3679)	206 / 126	0,0000
QOLIBRI-6	0,3078	(0,2383 to 0,3772)	204 / 124	0,0000
QOLI-OS-6	0,3210	(0,2537 to 0,3883)	206 / 127	0,0000
GAD7-6	0,3850	(0,3234 to 0,4467)	205 / 127	0,0003
PHQ9-6	0,3396	(0,2784 to 0,4007)	202 / 127	0,0000
PCL5-6	0,2974	(0,2370 to 0,3578)	209 / 121	0,0000
RPQ-6	0,2634	(0,2049 to 0,3219)	209 / 127	0,0000
**Wei-Lachin**				
COMBINED	0,3249	(0,2770 to 0,3728)	2053 / 1253	0,0000

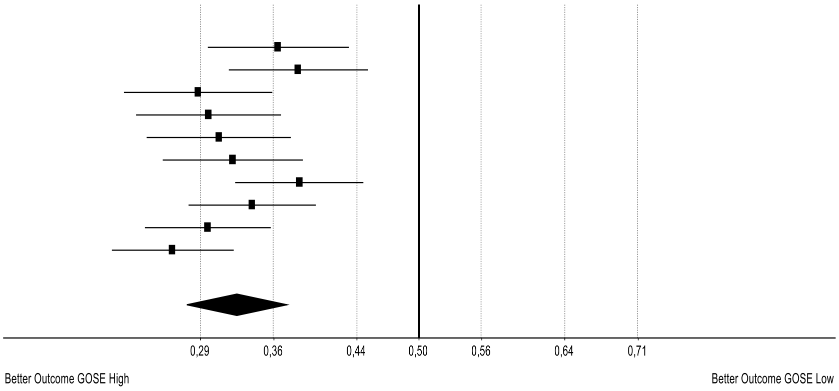

GOSE/-Q 5-6 vs. GOSE/-Q 3-4 - Present

Analysis	MW	95,00%-CI	N1/N2	P-Value
**Y2232-6D**				
SF36MCS-6	0,5473	(0,4608 to 0,6337)	124 / 61	0,2839
SF12MCS-6	0,5537	(0,4682 to 0,6392)	126 / 62	0,2183
SF36PCS-6	0,3787	(0,2849 to 0,4725)	124 / 61	0,0112
SF12PCS-6	0,3624	(0,2686 to 0,4562)	126 / 62	0,0040
QOLIBRI-6	0,4713	(0,3814 to 0,5612)	124 / 59	0,5316
QOLI-OS-6	0,4916	(0,4049 to 0,5784)	127 / 65	0,8502
GAD7-6	0,5071	(0,4176 to 0,5966)	127 / 57	0,8762
PHQ9-6	0,5214	(0,4315 to 0,6113)	127 / 58	0,6412
PCL5-6	0,5810	(0,4882 to 0,6738)	121 / 58	0,0872
RPQ-6	0,5654	(0,4748 to 0,6560)	127 / 58	0,1569
**Wei-Lachin**				
COMBINED	0,4980	(0,4295 to 0,5665)	1253 / 601	0,9542

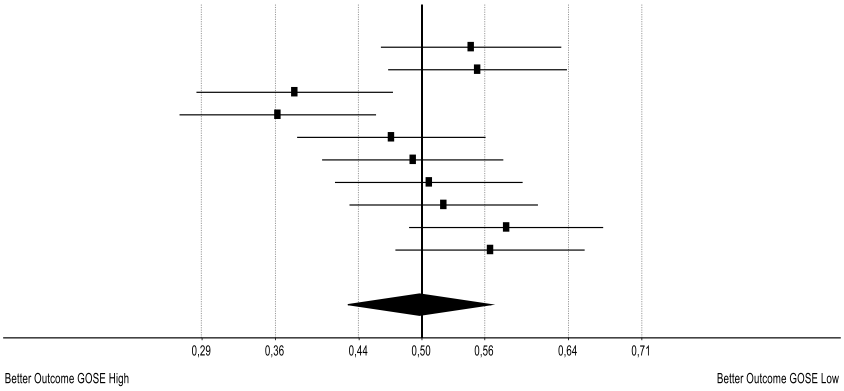

GOSE/-Q 7-8 vs. GOSE/-Q 3-4 - Present

Analysis	MW	95,00%-CI	N1/N2	P-Value
**Y1232-6D**				
SF36MCS-6	0,4243	(0,3366 to 0,5121)	203 / 61	0,0911
SF12MCS-6	0,4394	(0,3542 to 0,5247)	206 / 62	0,1639
SF36PCS-6	0,2062	(0,1023 to 0,3101)	203 / 61	0,0000
SF12PCS-6	0,2008	(0,0978 to 0,3038)	206 / 62	0,0000
QOLIBRI-6	0,2996	(0,2031 to 0,3961)	204 / 59	0,0000
QOLI-OS-6	0,3375	(0,2476 to 0,4273)	206 / 65	0,0004
GAD7-6	0,3909	(0,3134 to 0,4684)	205 / 57	0,0058
PHQ9-6	0,3570	(0,2821 to 0,4319)	202 / 58	0,0002
PCL5-6	0,3698	(0,2950 to 0,4446)	209 / 58	0,0006
RPQ-6	0,2922	(0,2258 to 0,3585)	209 / 58	0,0000
**Wei-Lachin**				
COMBINED	0,3318	(0,2677 to 0,3958)	2053 / 601	0,0000

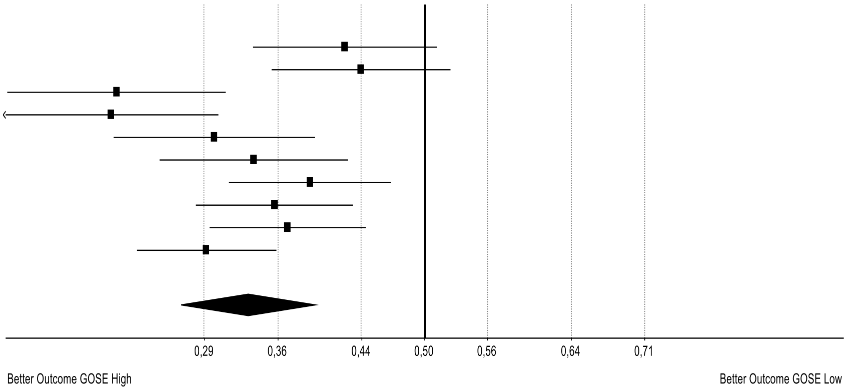

12 months after TBI (data as available)

GOSE/-Q 7-8 vs. GOSE/-Q 5-6 -Absent

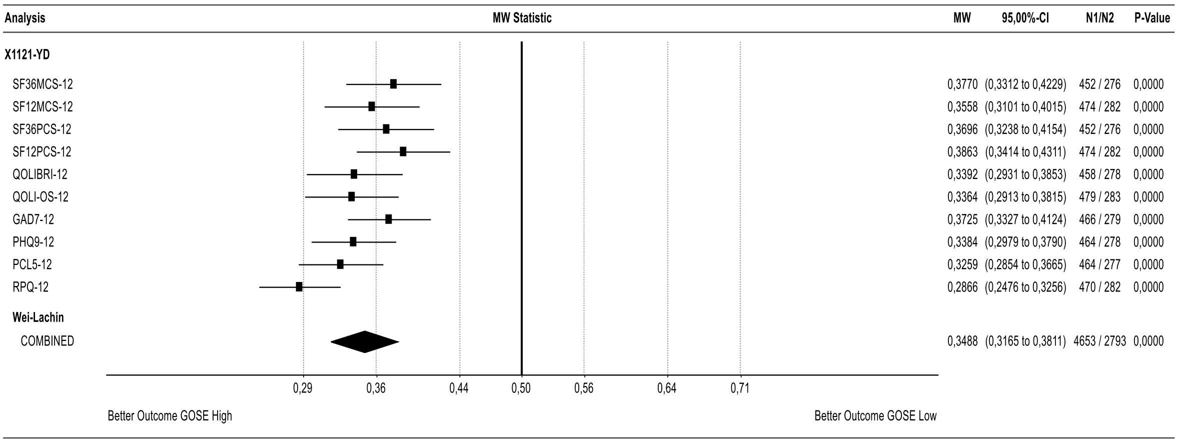

GOSE/-Q 5-6 vs. GOSE/-Q 3-4 - Absent

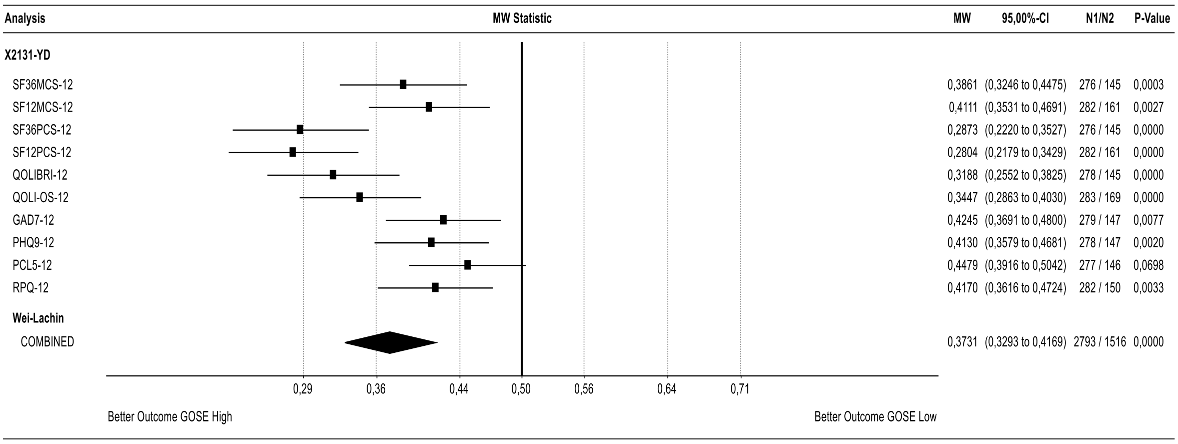

GOSE/-Q 7-8 vs. GOSE/-Q 3-4 - Absent

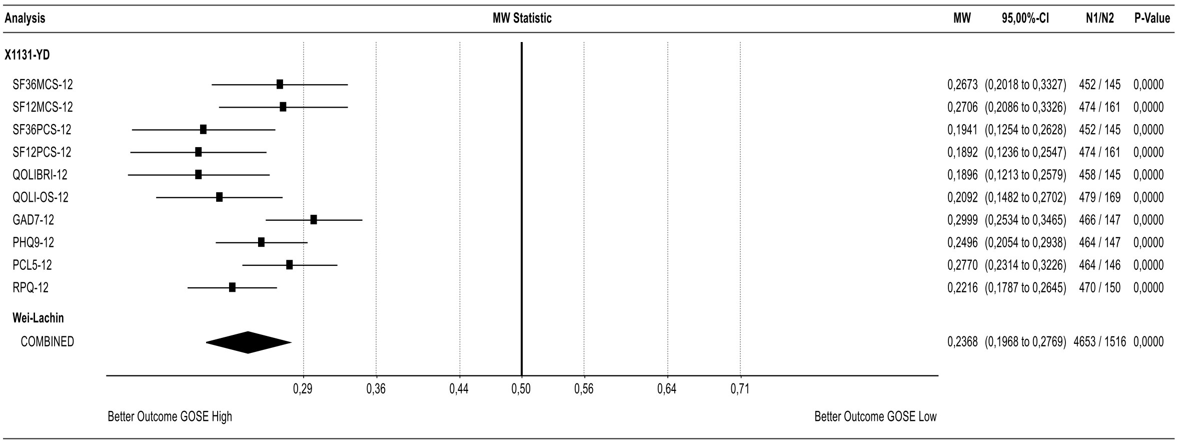

GOSE/-Q 7-8 vs. GOSE/-Q 5-6- Present

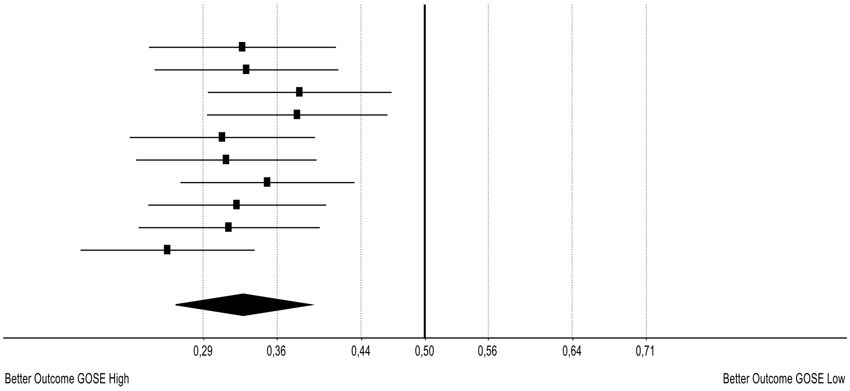

Analysis	MW	95,00%-CI	N1/N2	P-Value
**Y1222-YD**				
SF36MCS-12	0,3272	(0,2386 to 0,4158)	92 / 82	0,0001
SF12MCS-12	0,3310	(0,2440 to 0,4179)	93 / 86	0,0001
SF36PCS-12	0,3819	(0,2948 to 0,4689)	92 / 82	0,0078
SF12PCS-12	0,3792	(0,2937 to 0,4648)	93 / 86	0,0057
QOLIBRI-12	0,3080	(0,2200 to 0,3960)	93 / 83	0,0000
QOLI-OS-12	0,3122	(0,2266 to 0,3979)	95 / 86	0,0000
GAD7-12	0,3512	(0,2685 to 0,4339)	93 / 84	0,0004
PHQ9-12	0,3223	(0,2379 to 0,4067)	93 / 83	0,0000
PCL5-12	0,3146	(0,2285 to 0,4007)	92 / 80	0,0000
RPQ-12	0,2560	(0,1734 to 0,3387)	93 / 84	0,0000
**Wei-Lachin**				
COMBINED	0,3284	(0,2642 to 0,3925)	929 / 836	0,0000

GOSE/-Q 5-6 vs. GOSE/-Q 3-4 - Present

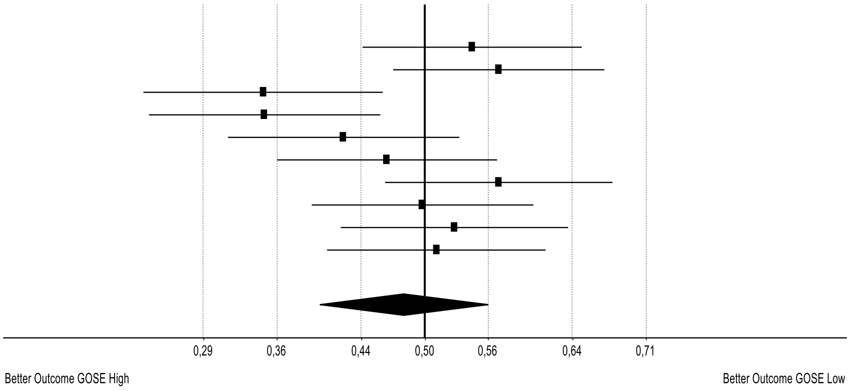

Analysis	MW	95,00%-CI	N1/N2	P-Value
**Y2232-YD**				
SF36MCS-12	0,5450	(0,4411 to 0,6488)	82 / 43	0,3961
SF12MCS-12	0,5704	(0,4701 to 0,6707)	86 / 46	0,1689
SF36PCS-12	0,3470	(0,2334 to 0,4606)	82 / 43	0,0083
SF12PCS-12	0,3481	(0,2385 to 0,4576)	86 / 46	0,0066
QOLIBRI-12	0,4232	(0,3138 to 0,5325)	83 / 44	0,1686
QOLI-OS-12	0,4644	(0,3603 to 0,5684)	86 / 47	0,5023
GAD7-12	0,5702	(0,4622 to 0,6782)	84 / 43	0,2029
PHQ9-12	0,4980	(0,3929 to 0,6031)	83 / 43	0,9708
PCL5-12	0,5280	(0,4202 to 0,6357)	80 / 42	0,6109
RPQ-12	0,5111	(0,4079 to 0,6143)	84 / 44	0,8332
**Wei-Lachin**				
COMBINED	0,4805	(0,4010 to 0,5600)	836 / 441	0,6312

GOSE/-Q 7-8 vs. GOSE/-Q 3-4 - Present

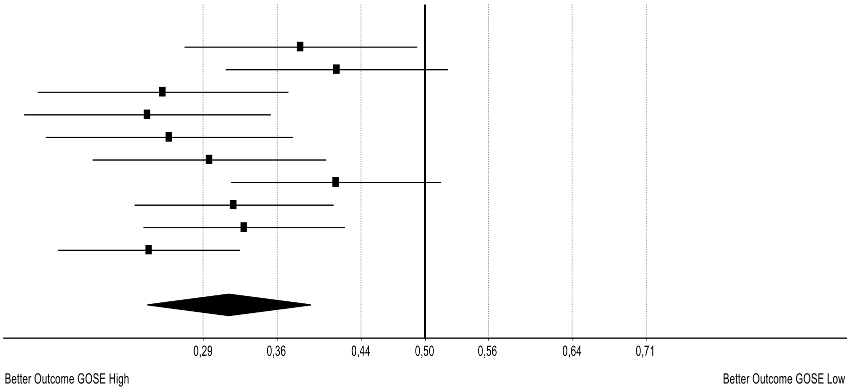

Analysis	MW	95,00%-CI	N1/N2	P-Value
**Y1232-YD**				
SF36MCS-12	0,3827	(0,2720 to 0,4934)	92 / 43	0,0378
SF12MCS-12	0,4168	(0,3114 to 0,5221)	93 / 46	0,1216
SF36PCS-12	0,2520	(0,1330 to 0,3710)	92 / 43	0,0000
SF12PCS-12	0,2371	(0,1202 to 0,3541)	93 / 46	0,0000
QOLIBRI-12	0,2581	(0,1409 to 0,3753)	93 / 44	0,0001
QOLI-OS-12	0,2961	(0,1854 to 0,4067)	95 / 47	0,0003
GAD7-12	0,4161	(0,3166 to 0,5156)	93 / 43	0,0984
PHQ9-12	0,3192	(0,2251 to 0,4133)	93 / 43	0,0002
PCL5-12	0,3285	(0,2330 to 0,4241)	92 / 42	0,0004
RPQ-12	0,2386	(0,1523 to 0,3250)	93 / 44	0,0000
**Wei-Lachin**				
COMBINED	0,3145	(0,2371 to 0,3920)	929 / 441	0,0000

3 months after TBI (completers)

GOSE/-Q 7-8 vs. GOSE/-Q 5-6 - Absent

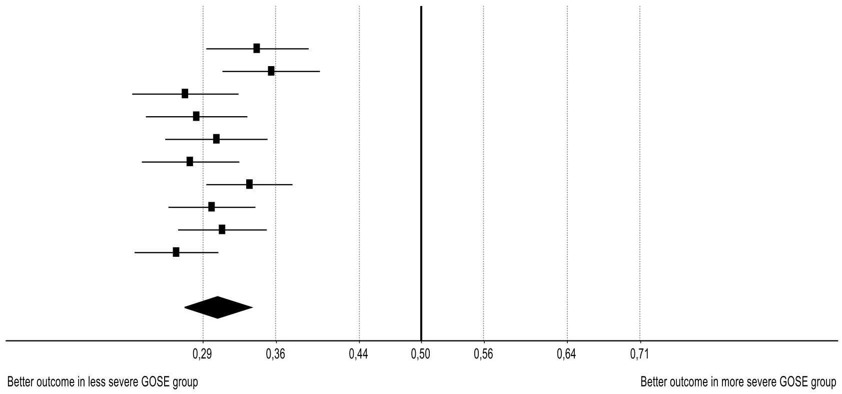

Analysis	MW	95,00%-CI	N1/N2	P-Value
**X1121-3X**				
SF36MCS-X	0,3423	(0,2931 to 0,3916)	408 / 248	0,0000
SF12MCS-X	0,3557	(0,3087 to 0,4027)	448 / 266	0,0000
SF36PCS-X	0,2730	(0,2221 to 0,3239)	408 / 248	0,0000
SF12PCS-X	0,2839	(0,2352 to 0,3327)	448 / 266	0,0000
QOLIBRI-X	0,3031	(0,2541 to 0,3521)	416 / 255	0,0000
QOLI-OS-X	0,2780	(0,2309 to 0,3252)	455 / 272	0,0000
GAD7-X3	0,3347	(0,2935 to 0,3760)	418 / 258	0,0000
PHQ9-X3	0,2988	(0,2569 to 0,3407)	417 / 260	0,0000
PCL5-X3	0,3084	(0,2658 to 0,3511)	419 / 255	0,0000
RPQ-X3	0,2645	(0,2240 to 0,3051)	431 / 267	0,0000
**Wei-Lachin**				
COMBINED	0,3043	(0,2721 to 0,3364)	4268 / 2595	0,0000

GOSE/-Q 5-6 vs. GOSE/-Q 3-4 - Absent

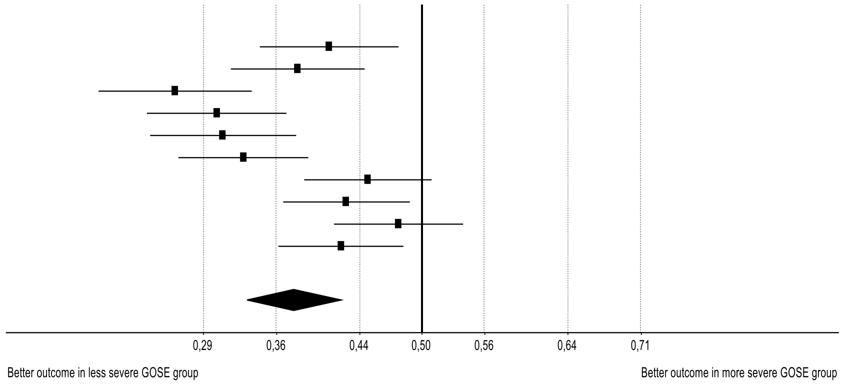

Analysis	MW	95,00%-CI	N1/N2	P-Value
**X2131-3X**				
SF36MCS-X	0,4109	(0,3441 to 0,4777)	248 / 117	0,0090
SF12MCS-X	0,3811	(0,3168 to 0,4453)	266 / 133	0,0003
SF36PCS-X	0,2628	(0,1891 to 0,3364)	248 / 117	0,0000
SF12PCS-X	0,3031	(0,2362 to 0,3701)	266 / 133	0,0000
QOLIBRI-X	0,3089	(0,2388 to 0,3791)	255 / 117	0,0000
QOLI-OS-X	0,3287	(0,2663 to 0,3911)	272 / 146	0,0000
GAD7-X3	0,4482	(0,3868 to 0,5096)	258 / 115	0,0984
PHQ9-X3	0,4275	(0,3666 to 0,4884)	260 / 116	0,0197
PCL5-X3	0,4775	(0,4152 to 0,5398)	255 / 113	0,4792
RPQ-X3	0,4224	(0,3623 to 0,4824)	267 / 117	0,0113
**Wei-Lachin**				
COMBINED	0,3771	(0,3316 to 0,4226)	2595 / 1224	0,0000

GOSE/-Q 7-8 vs. GOSE/-Q 3-4 - Absent

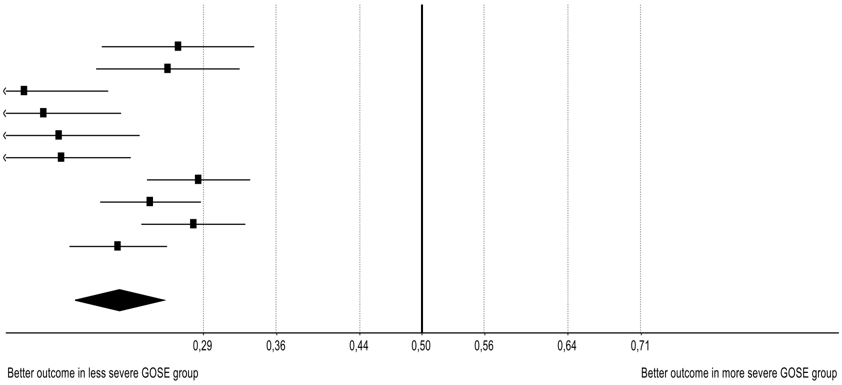

Analysis	MW	95,00%-CI	N1/N2	P-Value
**X1131-3X**				
SF36MCS-X	0,2660	(0,1928 to 0,3392)	408 / 117	0,0000
SF12MCS-X	0,2562	(0,1874 to 0,3251)	448 / 133	0,0000
SF36PCS-X	0,1179	(0,0367 to 0,1990)	408 / 117	0,0000
SF12PCS-X	0,1365	(0,0618 to 0,2112)	448 / 133	0,0000
QOLIBRI-X	0,1517	(0,0743 to 0,2292)	416 / 117	0,0000
QOLI-OS-X	0,1538	(0,0868 to 0,2208)	455 / 146	0,0000
GAD7-X3	0,2857	(0,2362 to 0,3352)	418 / 115	0,0000
PHQ9-X3	0,2392	(0,1907 to 0,2877)	417 / 116	0,0000
PCL5-X3	0,2805	(0,2307 to 0,3303)	419 / 113	0,0000
RPQ-X3	0,2084	(0,1615 to 0,2554)	431 / 117	0,0000
**Wei-Lachin**				
COMBINED	0,2096	(0,1670 to 0,2522)	4268 / 1224	0,0000

GOSE/-Q 7-8 vs. GOSE/-Q 5-6- Present

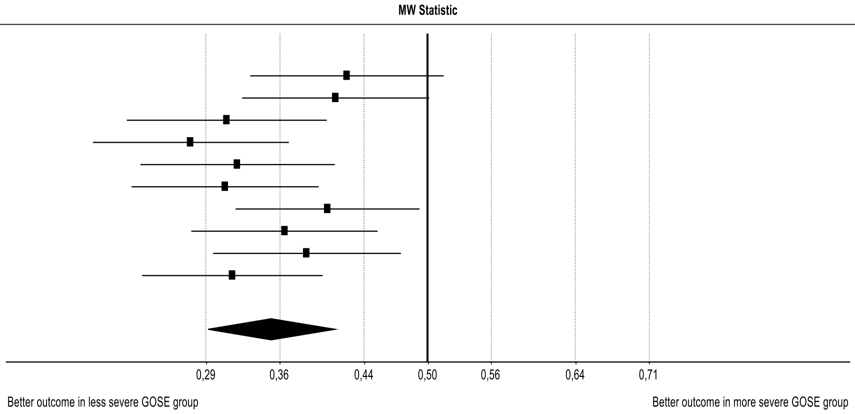

Analysis	MW	95,00%-CI	N1/N2	P-Value
**Y1222-3X**				
SF36MCS-X	0,4234	(0,3319 to 0,5149)	84 / 71	0,1007
SF12MCS-X	0,4132	(0,3246 to 0,5018)	87 / 78	0,0549
SF36PCS-X	0,3099	(0,2151 to 0,4047)	84 / 71	0,0001
SF12PCS-X	0,2756	(0,1829 to 0,3682)	87 / 78	0,0000
QOLIBRI-X	0,3199	(0,2277 to 0,4121)	87 / 74	0,0001
QOLI-OS-X	0,3084	(0,2197 to 0,3971)	90 / 80	0,0000
GAD7-X3	0,4051	(0,3181 to 0,4920)	87 / 75	0,0323
PHQ9-X3	0,3645	(0,2763 to 0,4528)	85 / 74	0,0026
PCL5-X3	0,3857	(0,2965 to 0,4750)	86 / 72	0,0121
RPQ-X3	0,3152	(0,2295 to 0,4009)	87 / 75	0,0000
**Wei-Lachin**				
COMBINED	0,3521	(0,2919 to 0,4123)	864 / 748	0,0000

GOSE/-Q 5-6 vs. GOSE/-Q 3-4 - Present

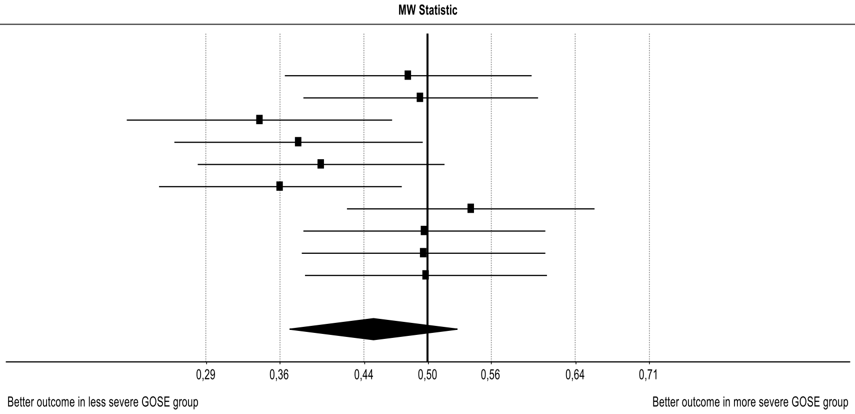

Analysis	MW	95,00%-CI	N1/N2	P-Value
**Y2232-3X**				
SF36MCS-X	0,4816	(0,3651 to 0,5982)	71 / 36	0,7572
SF12MCS-X	0,4934	(0,3826 to 0,6043)	78 / 39	0,9075
SF36PCS-X	0,3408	(0,2152 to 0,4663)	71 / 36	0,0129
SF12PCS-X	0,3777	(0,2598 to 0,4957)	78 / 39	0,0421
QOLIBRI-X	0,3988	(0,2819 to 0,5158)	74 / 38	0,0899
QOLI-OS-X	0,3603	(0,2454 to 0,4752)	80 / 42	0,0172
GAD7-X3	0,5410	(0,4237 to 0,6582)	75 / 35	0,4937
PHQ9-X3	0,4972	(0,3827 to 0,6117)	74 / 36	0,9616
PCL5-X3	0,4962	(0,3807 to 0,6118)	72 / 35	0,9490
RPQ-X3	0,4985	(0,3839 to 0,6131)	75 / 35	0,9792
**Wei-Lachin**				
COMBINED	0,4485	(0,3692 to 0,5279)	748 / 371	0,2039

GOSE/-Q 7-8 vs. GOSE/-Q 3-4 - Present

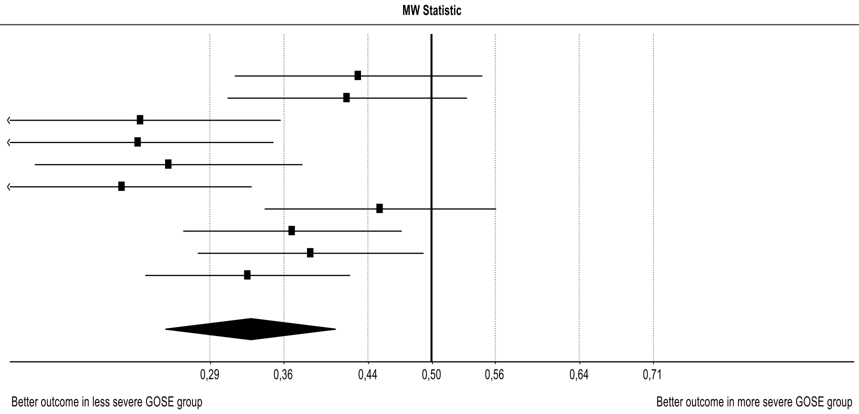

Analysis	MW	95,00%-CI	N1/N2	P-Value
**Y1232-3X**				
SF36MCS-X	0,4307	(0,3132 to 0,5483)	84 / 36	0,2480
SF12MCS-X	0,4201	(0,3065 to 0,5338)	87 / 39	0,1685
SF36PCS-X	0,2240	(0,0911 to 0,3569)	84 / 36	0,0000
SF12PCS-X	0,2218	(0,0932 to 0,3503)	87 / 39	0,0000
QOLIBRI-X	0,2509	(0,1242 to 0,3776)	87 / 38	0,0001
QOLI-OS-X	0,2067	(0,0839 to 0,3296)	90 / 42	0,0000
GAD7-X3	0,4512	(0,3416 to 0,5608)	87 / 35	0,3831
PHQ9-X3	0,3681	(0,2644 to 0,4719)	85 / 36	0,0127
PCL5-X3	0,3854	(0,2783 to 0,4925)	86 / 35	0,0359
RPQ-X3	0,3259	(0,2290 to 0,4229)	87 / 35	0,0004
**Wei-Lachin**				
COMBINED	0,3285	(0,2477 to 0,4093)	864 / 371	0,0000

6 months after TBI (completers)

GOSE/-Q 7-8 vs. GOSE/-Q 5-6 - Absent

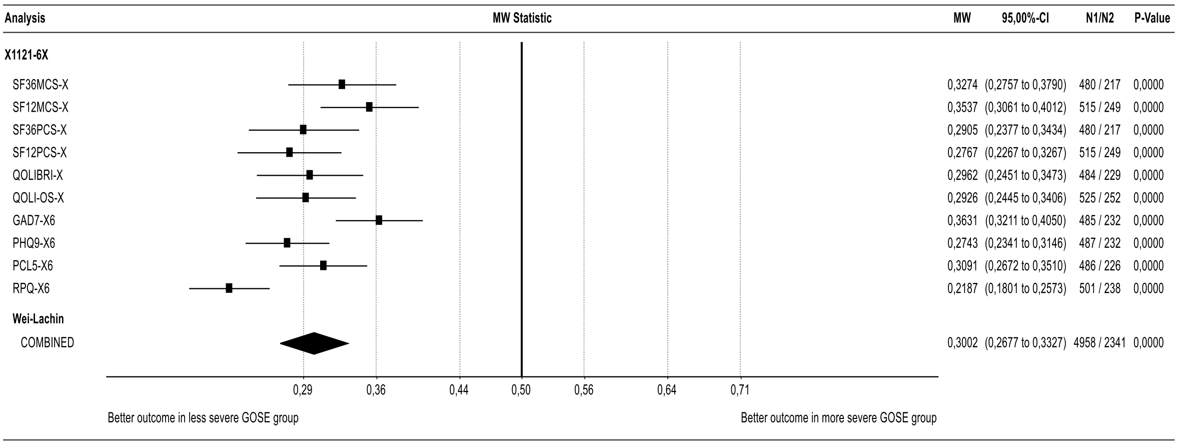

GOSE/-Q 5-6 vs. GOSE/-Q 3-4 – Absent

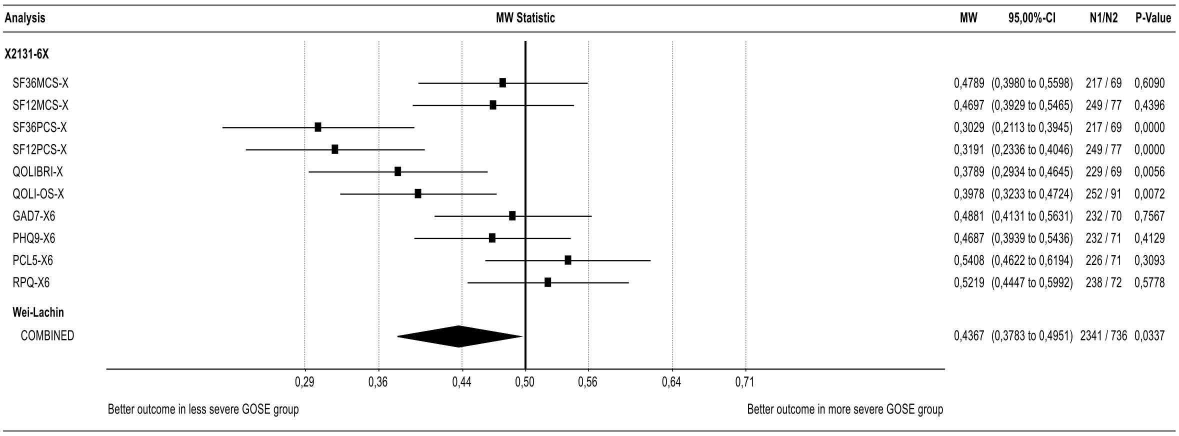

GOSE/-Q 7-8 vs. GOSE/-Q 3-4 - Absent

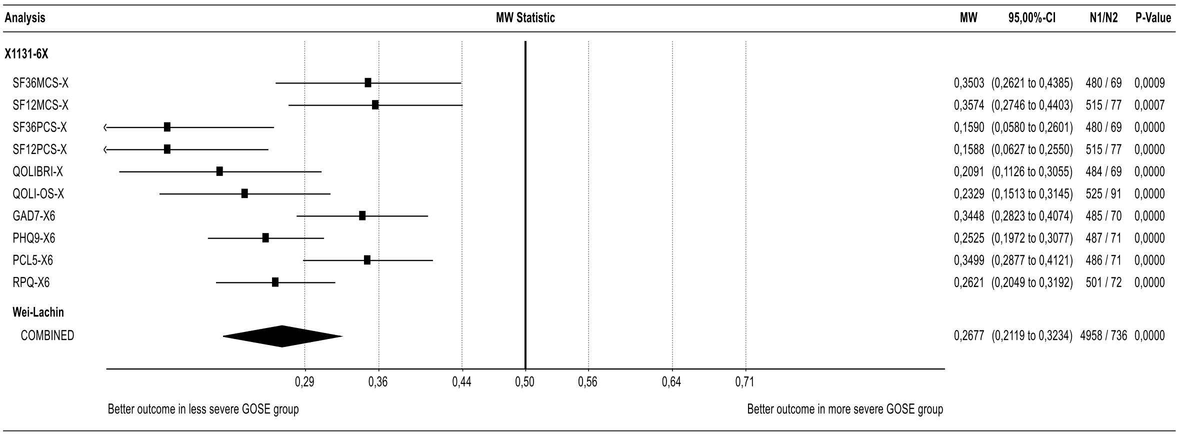

GOSE/-Q 7-8 vs. GOSE/-Q 5-6- Present

Analysis	MW	95,00%-CI	N1/N2	P-Value
**Y1222-6X**				
SF36MCS-X	0,2653	(0,1737 to 0,3569)	94 / 80	0,0000
SF12MCS-X	0,2926	(0,2059 to 0,3793)	98 / 88	0,0000
SF36PCS-X	0,2483	(0,1561 to 0,3404)	94 / 80	0,0000
SF12PCS-X	0,2472	(0,1587 to 0,3356)	98 / 88	0,0000
QOLIBRI-X	0,2419	(0,1532 to 0,3306)	95 / 87	0,0000
QOLI-OS-X	0,2690	(0,1843 to 0,3537)	101 / 90	0,0000
GAD7-X6	0,2634	(0,1809 to 0,3459)	96 / 85	0,0000
PHQ9-X6	0,2796	(0,1959 to 0,3633)	94 / 85	0,0000
PCL5-X6	0,2746	(0,1895 to 0,3596)	94 / 84	0,0000
RPQ-X6	0,2465	(0,1637 to 0,3294)	96 / 85	0,0000
**Wei-Lachin**				
COMBINED	0,2628	(0,2039 to 0,3217)	960 / 852	0,0000

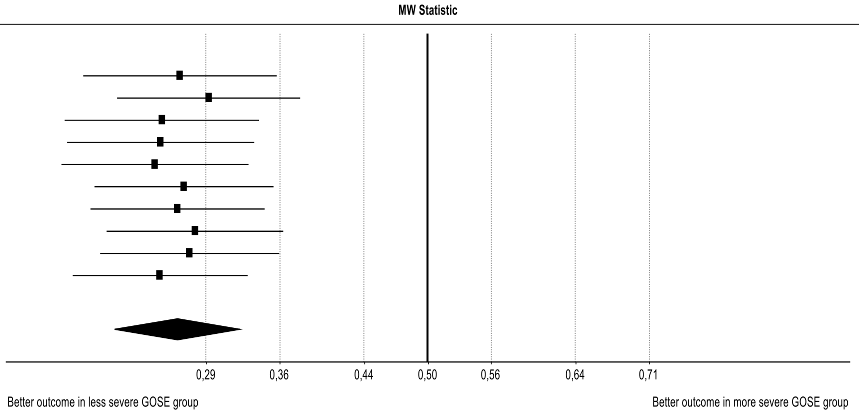

12 months after TBI (completers)

GOSE/-Q 7-8 vs. GOSE/-Q 5-6 -Absent

Analysis	MW	95,00%-CI	N1/N2	P-Value
**X1121-YX**				
SF36MCSX1	0,2951	(0,2383 to 0,3519)	525 / 186	0,0000
SF12MCSX1	0,2942	(0,2400 to 0,3485)	569 / 207	0,0000
SF36PCSX1	0,2976	(0,2414 to 0,3539)	525 / 186	0,0000
SF12PCSX1	0,3030	(0,2498 to 0,3562)	569 / 207	0,0000
QOLIBRIX1	0,2340	(0,1764 to 0,2916)	540 / 189	0,0000
QOLI-OSX1	0,2238	(0,1701 to 0,2775)	584 / 210	0,0000
GAD7-X12	0,3343	(0,2916 to 0,3770)	537 / 193	0,0000
PHQ9-X12	0,2557	(0,2156 to 0,2958)	539 / 193	0,0000
PCL5-X12	0,2577	(0,2167 to 0,2986)	540 / 187	0,0000
RPQ-X12	0,2016	(0,1639 to 0,2392)	557 / 195	0,0000
**Wei-Lachin**				
COMBINED	0,2697	(0,2369 to 0,3025)	5485 / 1953	0,0000

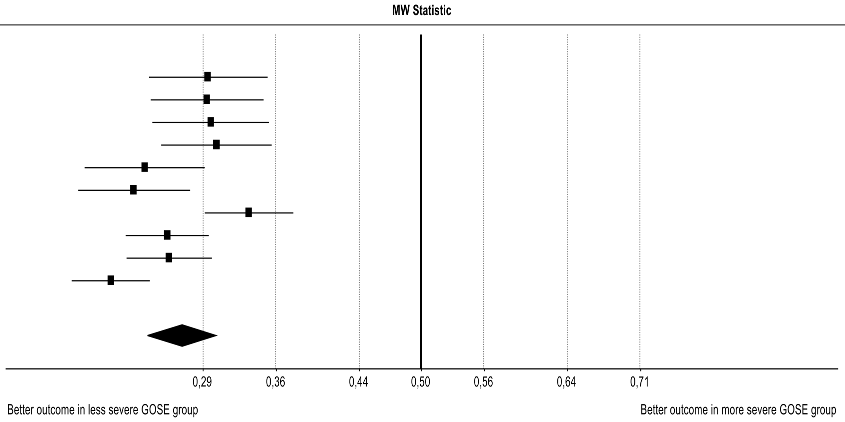

GOSE/-Q 5-6 vs. GOSE/-Q 3-4 - Absent

Analysis	MW	95,00%-CI	N1/N2	P-Value
**X2131-YX**				
SF36MCSX1	0,3769	(0,2826 to 0,4713)	186 / 55	0,0106
SF12MCSX1	0,4299	(0,3462 to 0,5137)	207 / 65	0,1010
SF36PCSX1	0,2573	(0,1526 to 0,3620)	186 / 55	0,0000
SF12PCSX1	0,2343	(0,1358 to 0,3329)	207 / 65	0,0000
QOLIBRIX1	0,2827	(0,1813 to 0,3842)	189 / 56	0,0000
QOLI-OSX1	0,3040	(0,2173 to 0,3907)	210 / 74	0,0000
GAD7-X12	0,4072	(0,3297 to 0,4846)	193 / 56	0,0188
PHQ9-X12	0,3954	(0,3180 to 0,4729)	193 / 56	0,0082
PCL5-X12	0,4541	(0,3722 to 0,5360)	187 / 56	0,2716
RPQ-X12	0,4491	(0,3669 to 0,5313)	195 / 58	0,2250
**Wei-Lachin**				
COMBINED	0,3591	(0,2963 to 0,4219)	1953 / 596	0,0000

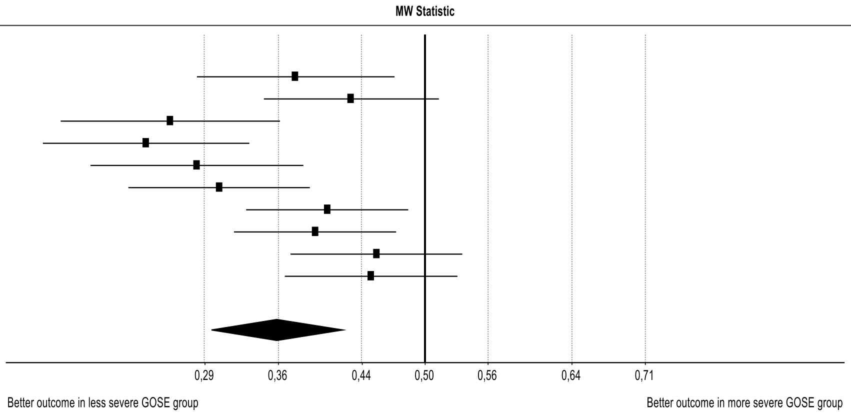

GOSE/-Q 7-8 vs. GOSE/-Q 3-4 - Absent

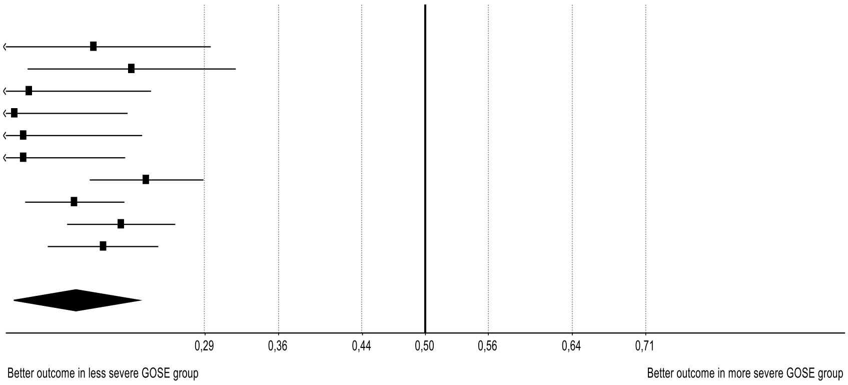

Analysis	MW	95,00%-CI	N1/N2	P-Value
**X1131-YX**				
SF36MCSX1	0,1846	(0,0735 to 0,2958)	525 / 55	0,0000
SF12MCSX1	0,2202	(0,1212 to 0,3193)	569 / 65	0,0000
SF36PCSX1	0,1228	(0,0072 to 0,2385)	525 / 55	0,0000
SF12PCSX1	0,1086	(0,0006 to 0,2166)	569 / 65	0,0000
QOLIBRIX1	0,1171	(0,0035 to 0,2307)	540 / 56	0,0000
QOLI-OSX1	0,1175	(0,0209 to 0,2141)	584 / 74	0,0000
GAD7-X12	0,2346	(0,1805 to 0,2886)	537 / 56	0,0000
PHQ9-X12	0,1661	(0,1192 to 0,2131)	539 / 56	0,0000
PCL5-X12	0,2104	(0,1591 to 0,2617)	540 / 56	0,0000
RPQ-X12	0,1931	(0,1405 to 0,2457)	557 / 58	0,0000
**Wei-Lachin**				
COMBINED	0,1675	(0,1081 to 0,2270)	5485 / 596	0,0000

GOSE/-Q 7-8 vs. GOSE/-Q 5-6- Present

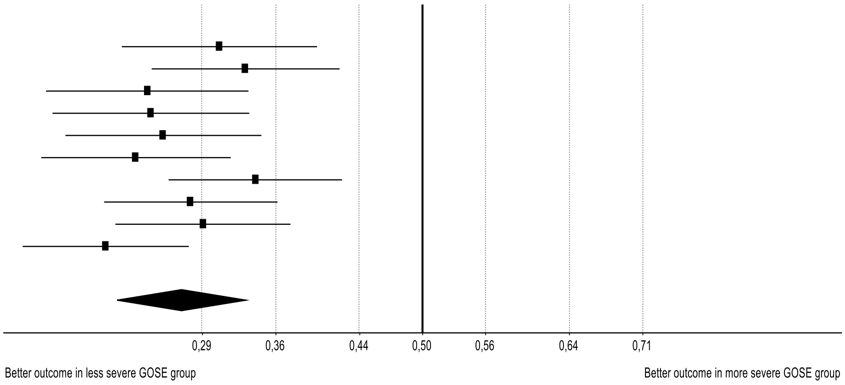

Analysis	MW	95,00%-CI	N1/N2	P-Value
**Y1222-YX**				
SF36MCSX1	0,3066	(0,2134 to 0,3998)	99 / 72	0,0000
SF12MCSX1	0,3315	(0,2418 to 0,4212)	106 / 76	0,0002
SF36PCSX1	0,2379	(0,1415 to 0,3342)	99 / 72	0,0000
SF12PCSX1	0,2411	(0,1472 to 0,3350)	106 / 76	0,0000
QOLIBRIX1	0,2530	(0,1594 to 0,3465)	105 / 73	0,0000
QOLI-OSX1	0,2269	(0,1364 to 0,3173)	111 / 77	0,0000
GAD7-X12	0,3409	(0,2582 to 0,4236)	105 / 73	0,0002
PHQ9-X12	0,2791	(0,1963 to 0,3619)	103 / 74	0,0000
PCL5-X12	0,2909	(0,2073 to 0,3745)	104 / 71	0,0000
RPQ-X12	0,1982	(0,1192 to 0,2772)	105 / 74	0,0000
**Wei-Lachin**				
COMBINED	0,2706	(0,2092 to 0,3321)	1043 / 738	0,0000

TBI severity

<u>3 months after TBI (data as available)</u>

GOSE/-Q 7-8 vs. GOSE/-Q 5-6 - Uncomplicated Mild TBI

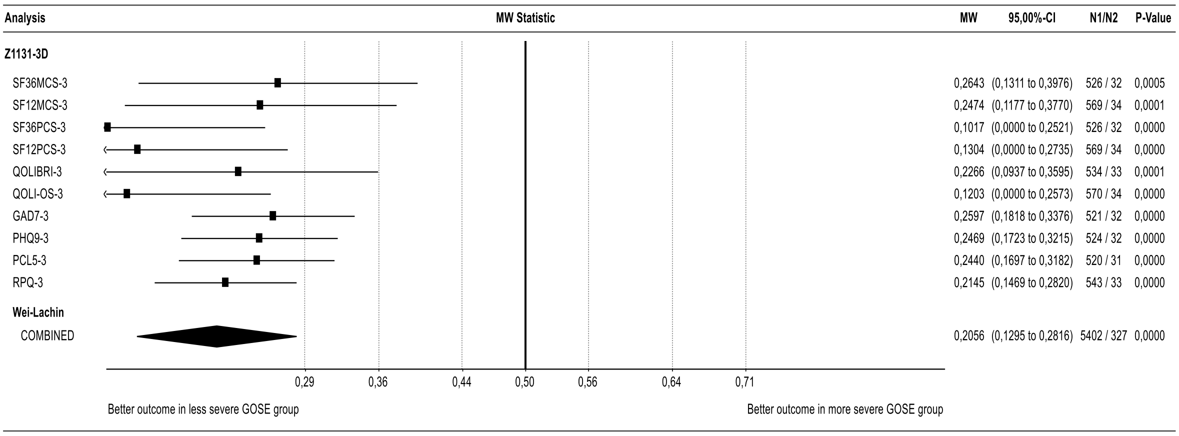

GOSE/-Q 5-6 vs. GOSE/-Q 3-4 - Uncomplicated Mild TBI

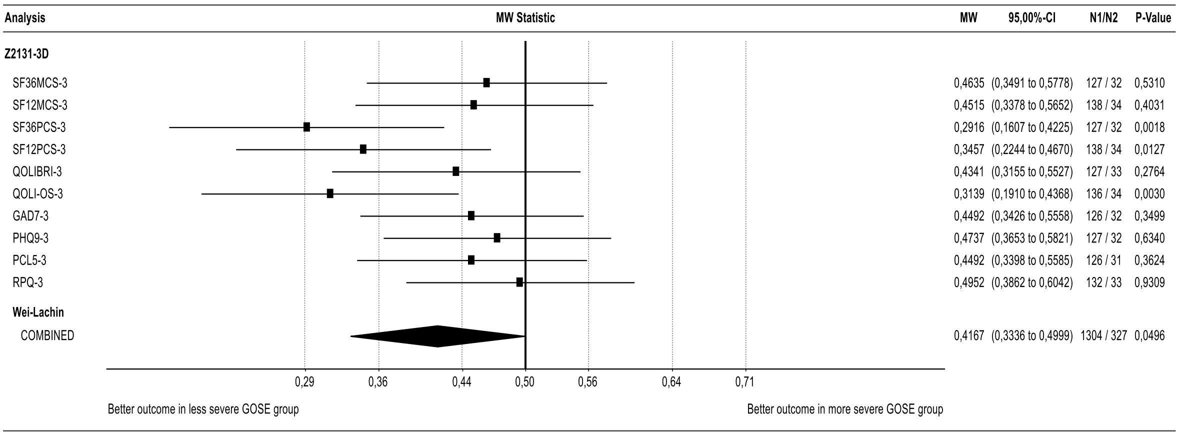

GOSE/-Q 7-8 vs. GOSE/-Q 3-4 - Uncomplicated Mild TBI

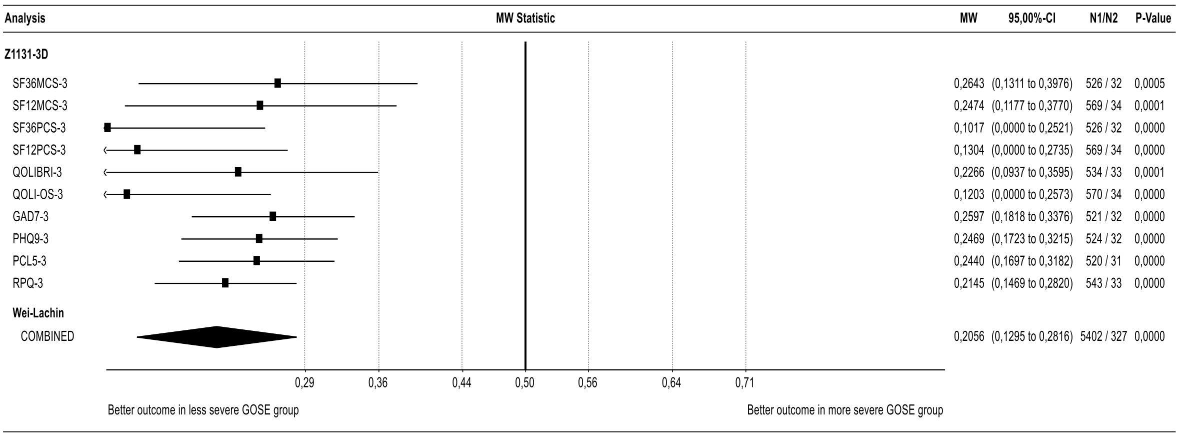

GOSE/-Q 7-8 vs. GOSE/-Q 5-6- Complicated Mild TBI

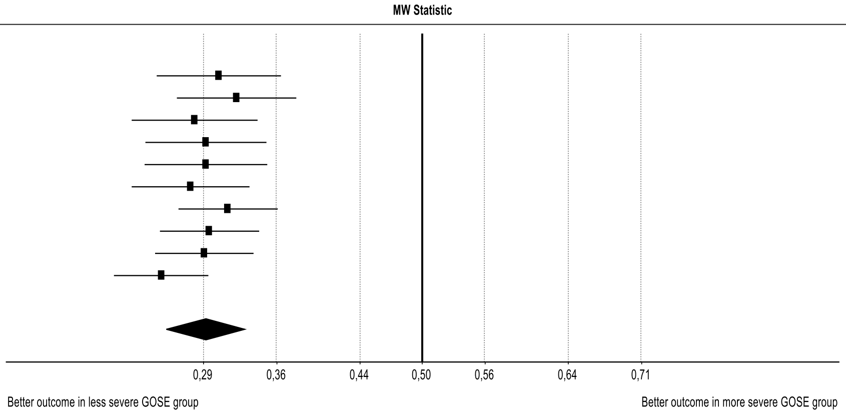

Analysis	MW	95,00%-CI	N1/N2	P-Value
**Z1222-3D**				
SF36MCS-3	0,3047	(0,2450 to 0,3644)	362 / 168	0,0000
SF12MCS-3	0,3220	(0,2648 to 0,3792)	386 / 181	0,0000
SF36PCS-3	0,2817	(0,2213 to 0,3421)	362 / 168	0,0000
SF12PCS-3	0,2925	(0,2347 to 0,3503)	386 / 181	0,0000
QOLIBRI-3	0,2926	(0,2338 to 0,3515)	361 / 173	0,0000
QOLI-OS-3	0,2777	(0,2209 to 0,3345)	382 / 184	0,0000
GAD7-3	0,3136	(0,2663 to 0,3610)	361 / 171	0,0000
PHQ9-3	0,2957	(0,2481 to 0,3433)	360 / 171	0,0000
PCL5-3	0,2908	(0,2433 to 0,3383)	361 / 171	0,0000
RPQ-3	0,2495	(0,2045 to 0,2945)	370 / 178	0,0000
**Wei-Lachin**				
COMBINED	0,2921	(0,2547 to 0,3295)	3691 / 1746	0,0000

GOSE/-Q 5-6 vs. GOSE/-Q 3-4 - Complicated Mild TBI

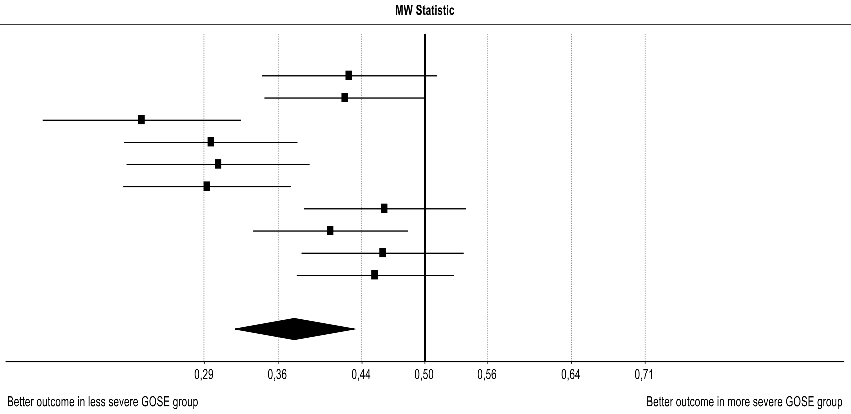

Analysis	MW	95,00%-CI	N1/N2	P-Value
**Z2232-3D**				
SF36MCS-3	0,4284	(0,3453 to 0,5116)	168 / 72	0,0915
SF12MCS-3	0,4243	(0,3475 to 0,5011)	181 / 86	0,0535
SF36PCS-3	0,2304	(0,1354 to 0,3253)	168 / 72	0,0000
SF12PCS-3	0,2962	(0,2132 to 0,3792)	181 / 86	0,0000
QOLIBRI-3	0,3032	(0,2157 to 0,3906)	173 / 75	0,0000
QOLI-OS-3	0,2926	(0,2128 to 0,3725)	184 / 91	0,0000
GAD7-3	0,4621	(0,3850 to 0,5393)	171 / 74	0,3359
PHQ9-3	0,4104	(0,3364 to 0,4843)	171 / 76	0,0174
PCL5-3	0,4600	(0,3830 to 0,5370)	171 / 72	0,3090
RPQ-3	0,4528	(0,3777 to 0,5279)	178 / 76	0,2182
**Wei-Lachin**				
COMBINED	0,3760	(0,3193 to 0,4328)	1746 / 780	0,0000

GOSE/-Q 7-8 vs. GOSE/-Q 3-4 - Complicated Mild TBI

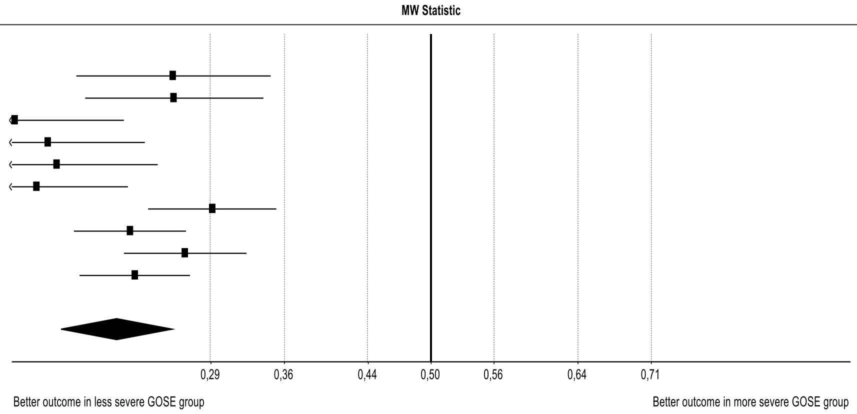

Analysis	MW	95,00%-CI	N1/N2	P-Value
**Z1232-3D**				
SF36MCS-3	0,2546	(0,1623 to 0,3470)	362 / 72	0,0000
SF12MCS-3	0,2553	(0,1706 to 0,3400)	386 / 86	0,0000
SF36PCS-3	0,1038	(0,0006 to 0,2071)	362 / 72	0,0000
SF12PCS-3	0,1352	(0,0432 to 0,2272)	386 / 86	0,0000
QOLIBRI-3	0,1437	(0,0474 to 0,2400)	361 / 75	0,0000
QOLI-OS-3	0,1239	(0,0368 to 0,2111)	382 / 91	0,0000
GAD7-3	0,2918	(0,2307 to 0,3530)	361 / 74	0,0000
PHQ9-3	0,2132	(0,1598 to 0,2667)	360 / 76	0,0000
PCL5-3	0,2658	(0,2074 to 0,3241)	361 / 72	0,0000
RPQ-3	0,2177	(0,1647 to 0,2707)	370 / 76	0,0000
**Wei-Lachin**				
COMBINED	0,2005	(0,1473 to 0,2537)	3691 / 780	0,0000

GOSE/-Q 7-8 vs. GOSE/-Q 5-6 - Moderate TBI

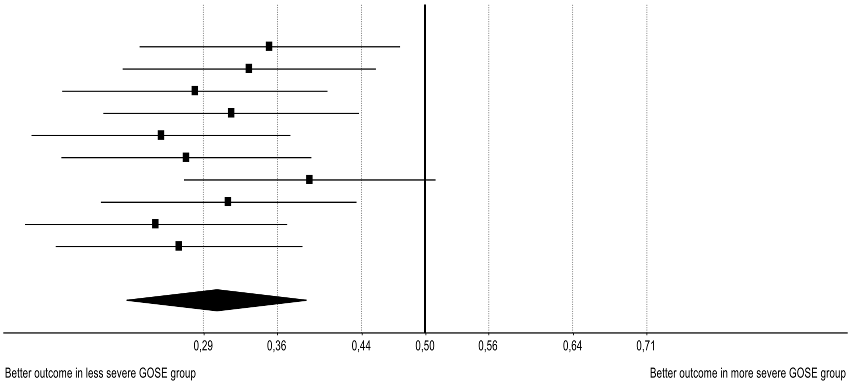

Analysis	MW	95,00%-CI	N1/N2	P-Value
**Z1323-3D**				
SF36MCS-3	0,3529	(0,2295 to 0,4763)	40 / 43	0,0195
SF12MCS-3	0,3333	(0,2133 to 0,4533)	45 / 45	0,0065
SF36PCS-3	0,2820	(0,1564 to 0,4076)	40 / 43	0,0007
SF12PCS-3	0,3163	(0,1955 to 0,4371)	45 / 45	0,0029
QOLIBRI-3	0,2499	(0,1271 to 0,3726)	41 / 45	0,0001
QOLI-OS-3	0,2741	(0,1555 to 0,3926)	46 / 47	0,0002
GAD7-3	0,3909	(0,2719 to 0,5099)	40 / 44	0,0723
PHQ9-3	0,3136	(0,1926 to 0,4347)	40 / 44	0,0025
PCL5-3	0,2451	(0,1210 to 0,3691)	40 / 43	0,0001
RPQ-3	0,2669	(0,1502 to 0,3836)	42 / 45	0,0001
**Wei-Lachin**				
COMBINED	0,3025	(0,2175 to 0,3875)	419 / 444	0,0000

GOSE/-Q 5-6 vs. GOSE/-Q 3-4 - Moderate TBI

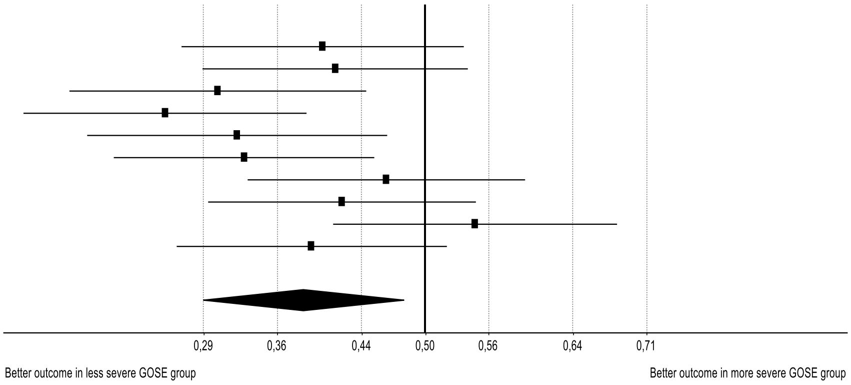

Analysis	MW	95,00%-CI	N1/N2	P-Value
**Z2333-3D**				
SF36MCS-3	0,4029	(0,2690 to 0,5367)	43 / 31	0,1550
SF12MCS-3	0,4150	(0,2894 to 0,5406)	45 / 37	0,1847
SF36PCS-3	0,3038	(0,1630 to 0,4446)	43 / 31	0,0063
SF12PCS-3	0,2538	(0,1198 to 0,3877)	45 / 37	0,0003
QOLIBRI-3	0,3218	(0,1799 to 0,4638)	45 / 29	0,0139
QOLI-OS-3	0,3285	(0,2054 to 0,4515)	47 / 40	0,0063
GAD7-3	0,4633	(0,3318 to 0,5947)	44 / 30	0,5838
PHQ9-3	0,4212	(0,2946 to 0,5478)	44 / 31	0,2223
PCL5-3	0,5477	(0,4132 to 0,6822)	43 / 29	0,4868
RPQ-3	0,3925	(0,2645 to 0,5204)	45 / 31	0,0996
**Wei-Lachin**				
COMBINED	0,3850	(0,2898 to 0,4803)	444 / 326	0,0180

GOSE/-Q 7-8 vs. GOSE/-Q 3-4 - Moderate TBI

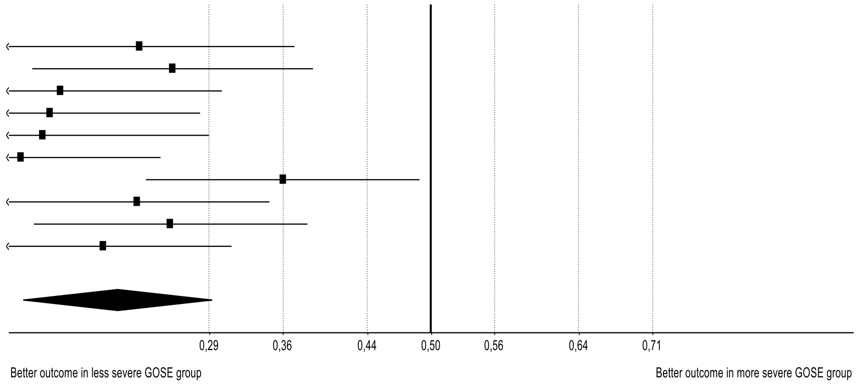

Analysis	MW	95,00%-CI	N1/N2	P-Value
**Z1333-3D**				
SF36MCS-3	0,2238	(0,0770 to 0,3705)	40 / 31	0,0002
SF12MCS-3	0,2556	(0,1228 to 0,3883)	45 / 37	0,0003
SF36PCS-3	0,1496	(0,0000 to 0,3023)	40 / 31	0,0000
SF12PCS-3	0,1390	(0,0000 to 0,2814)	45 / 37	0,0000
QOLIBRI-3	0,1325	(0,0000 to 0,2897)	41 / 29	0,0000
QOLI-OS-3	0,1122	(0,0000 to 0,2437)	46 / 40	0,0000
GAD7-3	0,3600	(0,2305 to 0,4895)	40 / 30	0,0341
PHQ9-3	0,2222	(0,0971 to 0,3473)	40 / 31	0,0000
PCL5-3	0,2534	(0,1241 to 0,3828)	40 / 29	0,0002
RPQ-3	0,1901	(0,0687 to 0,3115)	42 / 31	0,0000
**Wei-Lachin**				
COMBINED	0,2038	(0,1144 to 0,2933)	419 / 326	0,0000

GOSE/-Q 7-8 vs. GOSE/-Q 5-6- Severe TBI

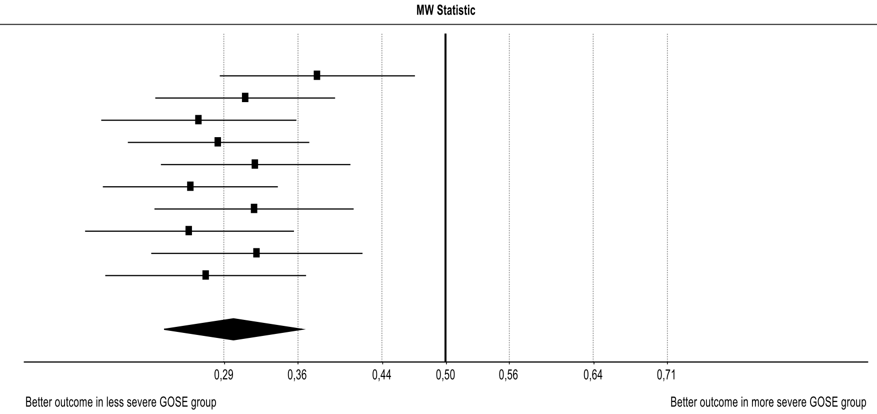

Analysis	MW	95,00%-CI	N1/N2	P-Value
**Z1424-3D**				
SF36MCS-3	0,3786	(0,2860 to 0,4712)	54 / 91	0,0102
SF12MCS-3	0,3103	(0,2249 to 0,3956)	63 / 107	0,0000
SF36PCS-3	0,2665	(0,1740 to 0,3590)	54 / 91	0,0000
SF12PCS-3	0,2849	(0,1992 to 0,3706)	63 / 107	0,0000
QOLIBRI-3	0,3200	(0,2304 to 0,4096)	56 / 99	0,0001
QOLI-OS-3	0,2583	(0,1754 to 0,3411)	67 / 109	0,0000
GAD7-3	0,3186	(0,2242 to 0,4129)	55 / 98	0,0002
PHQ9-3	0,2574	(0,1582 to 0,3566)	56 / 98	0,0000
PCL5-3	0,3210	(0,2209 to 0,4211)	56 / 99	0,0005
RPQ-3	0,2727	(0,1772 to 0,3681)	58 / 100	0,0000
**Wei-Lachin**				
COMBINED	0,2988	(0,2333 to 0,3643)	582 / 999	0,0000

GOSE/-Q 5-6 vs. GOSE/-Q 3-4 - Severe TBI

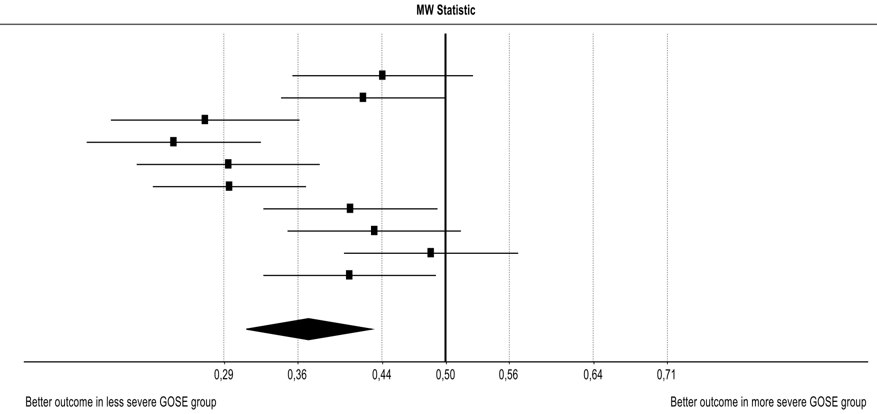

Analysis	MW	95,00%-CI	N1/N2	P-Value
**Z2434-3D**				
SF36MCS-3	0,4403	(0,3550 to 0,5257)	91 / 85	0,1705
SF12MCS-3	0,4221	(0,3438 to 0,5004)	107 / 103	0,0512
SF36PCS-3	0,2723	(0,1828 to 0,3619)	91 / 85	0,0000
SF12PCS-3	0,2426	(0,1599 to 0,3252)	107 / 103	0,0000
QOLIBRI-3	0,2943	(0,2077 to 0,3809)	99 / 87	0,0000
QOLI-OS-3	0,2950	(0,2224 to 0,3675)	109 / 130	0,0000
GAD7-3	0,4098	(0,3271 to 0,4926)	98 / 86	0,0327
PHQ9-3	0,4324	(0,3502 to 0,5146)	98 / 86	0,1071
PCL5-3	0,4861	(0,4035 to 0,5688)	99 / 86	0,7425
RPQ-3	0,4091	(0,3276 to 0,4905)	100 / 90	0,0286
**Wei-Lachin**				
COMBINED	0,3704	(0,3114 to 0,4295)	999 / 941	0,0000

GOSE/-Q 7-8 vs. GOSE/-Q 3-4 - Severe TBI

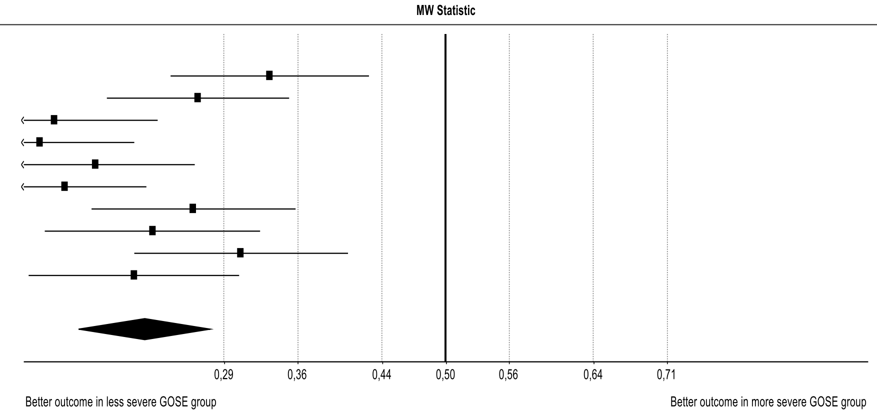

Analysis	MW	95,00%-CI	N1/N2	P-Value
**Z1434-3D**				
SF36MCS-3	0,3338	(0,2398 to 0,4277)	54 / 85	0,0005
SF12MCS-3	0,2653	(0,1790 to 0,3516)	63 / 103	0,0000
SF36PCS-3	0,1297	(0,0326 to 0,2269)	54 / 85	0,0000
SF12PCS-3	0,1158	(0,0268 to 0,2048)	63 / 103	0,0000
QOLIBRI-3	0,1684	(0,0745 to 0,2623)	56 / 87	0,0000
QOLI-OS-3	0,1395	(0,0626 to 0,2164)	67 / 130	0,0000
GAD7-3	0,2612	(0,1644 to 0,3580)	55 / 86	0,0000
PHQ9-3	0,2223	(0,1205 to 0,3241)	56 / 86	0,0000
PCL5-3	0,3063	(0,2048 to 0,4077)	56 / 86	0,0002
RPQ-3	0,2048	(0,1048 to 0,3047)	58 / 90	0,0000
**Wei-Lachin**				
COMBINED	0,2147	(0,1524 to 0,2770)	582 / 941	0,0000

6 months after TBI (data as available)

GOSE/-Q 7-8 vs. GOSE/-Q 5-6- Complicated Mild TBI

Analysis	MW	95,00%-CI	N1/N2	P-Value
**Z1222-6D**				
SF36MCS-6	0,3233	(0,2656 to 0,3811)	339 / 175	0,0000
SF12MCS-6	0,3405	(0,2833 to 0,3977)	349 / 175	0,0000
SF36PCS-6	0,3455	(0,2885 to 0,4025)	339 / 175	0,0000
SF12PCS-6	0,3529	(0,2963 to 0,4095)	349 / 175	0,0000
QOLIBRI-6	0,3157	(0,2572 to 0,3741)	341 / 169	0,0000
QOLI-OS-6	0,3213	(0,2656 to 0,3771)	350 / 179	0,0000
GAD7-6	0,3567	(0,3077 to 0,4056)	340 / 172	0,0000
PHQ9-6	0,3099	(0,2611 to 0,3588)	339 / 171	0,0000
PCL5-6	0,2820	(0,2340 to 0,3301)	342 / 172	0,0000
RPQ-6	0,2823	(0,2355 to 0,3292)	345 / 178	0,0000
**Wei-Lachin**				
COMBINED	0,3230	(0,2839 to 0,3622)	3433 / 1741	0,0000

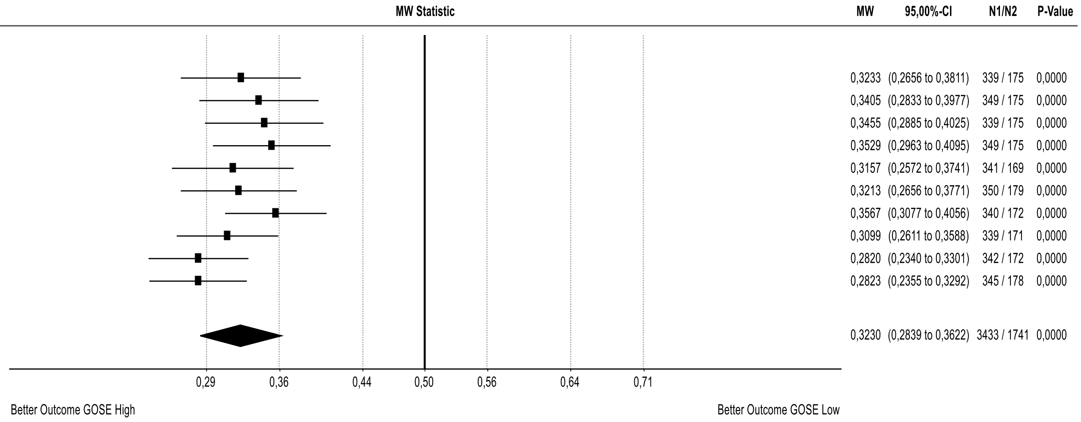

GOSE/-Q 5-6 vs. GOSE/-Q 3-4 - Complicated Mild TBI

Analysis	MW	95,00%-CI	N1/N2	P-Value
**Z2232-6D**				
SF36MCS-6	0,4617	(0,3841 to 0,5393)	175 / 82	0,3330
SF12MCS-6	0,4674	(0,3912 to 0,5437)	175 / 85	0,4025
SF36PCS-6	0,2422	(0,1532 to 0,3312)	175 / 82	0,0000
SF12PCS-6	0,2449	(0,1578 to 0,3320)	175 / 85	0,0000
QOLIBRI-6	0,3475	(0,2635 to 0,4315)	169 / 80	0,0004
QOLI-OS-6	0,3500	(0,2700 to 0,4301)	179 / 86	0,0002
GAD7-6	0,4497	(0,3750 to 0,5244)	172 / 78	0,1869
PHQ9-6	0,4245	(0,3507 to 0,4983)	171 / 79	0,0449
PCL5-6	0,5136	(0,4360 to 0,5913)	172 / 75	0,7307
RPQ-6	0,4407	(0,3677 to 0,5136)	178 / 82	0,1109
**Wei-Lachin**				
COMBINED	0,3942	(0,3354 to 0,4531)	1741 / 814	0,0004

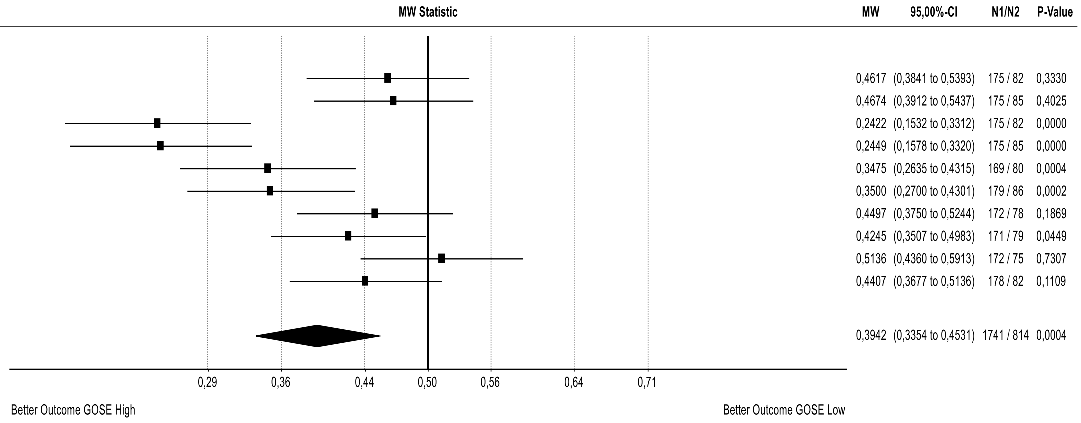

GOSE/-Q 7-8 vs. GOSE/-Q 3-4 - Complicated Mild TBI

Analysis	MW	95,00%-CI	N1/N2	P-Value
**Z1232-6D**				
SF36MCS-6	0,3080	(0,2253 to 0,3907)	339 / 82	0,0000
SF12MCS-6	0,3316	(0,2516 to 0,4115)	349 / 85	0,0000
SF36PCS-6	0,1606	(0,0674 to 0,2538)	339 / 82	0,0000
SF12PCS-6	0,1589	(0,0676 to 0,2501)	349 / 85	0,0000
QOLIBRI-6	0,2147	(0,1257 to 0,3037)	341 / 80	0,0000
QOLI-OS-6	0,2181	(0,1342 to 0,3021)	350 / 86	0,0000
GAD7-6	0,3077	(0,2479 to 0,3674)	340 / 78	0,0000
PHQ9-6	0,2445	(0,1883 to 0,3007)	339 / 79	0,0000
PCL5-6	0,2850	(0,2260 to 0,3440)	342 / 75	0,0000
RPQ-6	0,2403	(0,1869 to 0,2937)	345 / 82	0,0000
**Wei-Lachin**				
COMBINED	0,2469	(0,1931 to 0,3008)	3433 / 814	0,0000

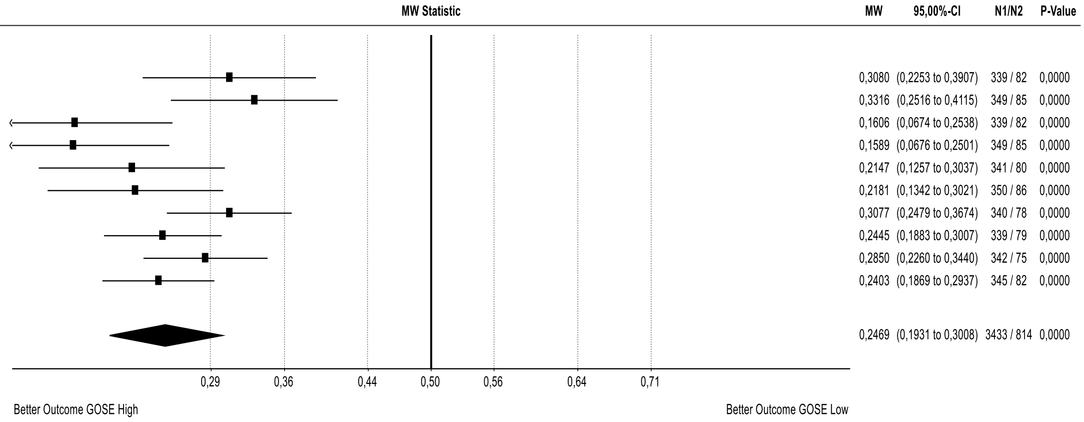

GOSE/-Q 7-8 vs. GOSE/-Q 5-6 - Moderate TBI

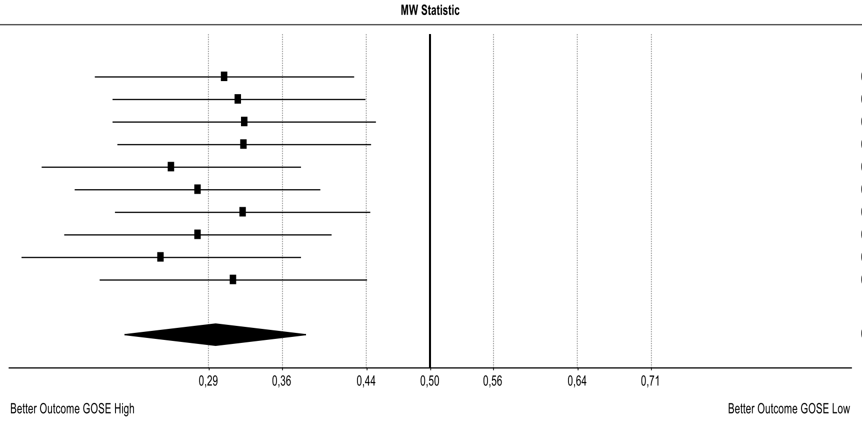

Analysis	MW	95,00%-CI	N1/N2	P-Value
**Z1323-6D**				
SF36MCS-6	0,3050	(0,1819 to 0,4281)	33 / 47	0,0019
SF12MCS-6	0,3185	(0,1986 to 0,4385)	37 / 49	0,0030
SF36PCS-6	0,3240	(0,1993 to 0,4486)	33 / 47	0,0057
SF12PCS-6	0,3238	(0,2037 to 0,4439)	37 / 49	0,0040
QOLIBRI-6	0,2547	(0,1319 to 0,3775)	33 / 47	0,0001
QOLI-OS-6	0,2797	(0,1631 to 0,3964)	39 / 50	0,0002
GAD7-6	0,3226	(0,2015 to 0,4438)	34 / 48	0,0041
PHQ9-6	0,2797	(0,1529 to 0,4065)	34 / 49	0,0007
PCL5-6	0,2450	(0,1126 to 0,3774)	34 / 47	0,0002
RPQ-6	0,3133	(0,1866 to 0,4401)	34 / 49	0,0039
**Wei-Lachin**				
COMBINED	0,2966	(0,2107 to 0,3826)	348 / 482	0,0000

GOSE/-Q 5-6 vs. GOSE/-Q 3-4 – Moderate TBI

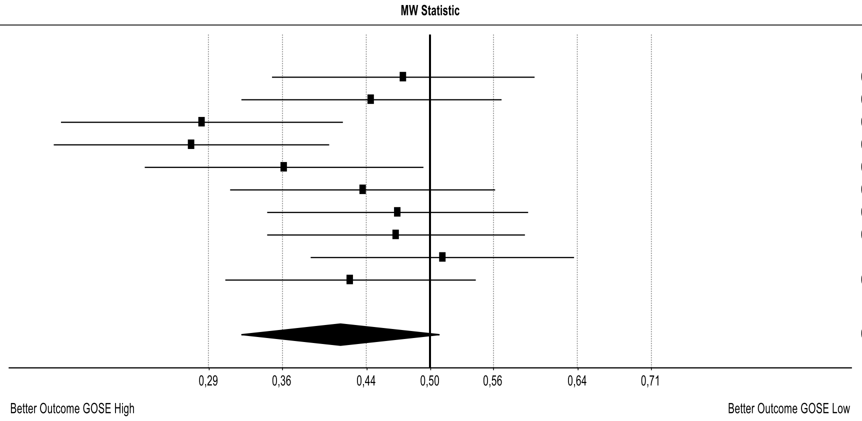

Analysis	MW	95,00%-CI	N1/N2	P-Value
**Z2333-6D**				
SF36MCS-6	0,4748	(0,3500 to 0,5996)	47 / 35	0,6919
SF12MCS-6	0,4446	(0,3213 to 0,5679)	49 / 37	0,3782
SF36PCS-6	0,2839	(0,1502 to 0,4176)	47 / 35	0,0015
SF12PCS-6	0,2739	(0,1432 to 0,4045)	49 / 37	0,0007
QOLIBRI-6	0,3617	(0,2295 to 0,4939)	47 / 33	0,0403
QOLI-OS-6	0,4362	(0,3104 to 0,5620)	50 / 34	0,3200
GAD7-6	0,4696	(0,3460 to 0,5933)	48 / 35	0,6304
PHQ9-6	0,4680	(0,3457 to 0,5902)	49 / 36	0,6075
PCL5-6	0,5119	(0,3868 to 0,6370)	47 / 34	0,8523
RPQ-6	0,4245	(0,3058 to 0,5433)	49 / 38	0,2129
**Wei-Lachin**				
COMBINED	0,4149	(0,3210 to 0,5088)	482 / 354	0,0756

GOSE/-Q 7-8 vs. GOSE/-Q 3-4 - Moderate TBI

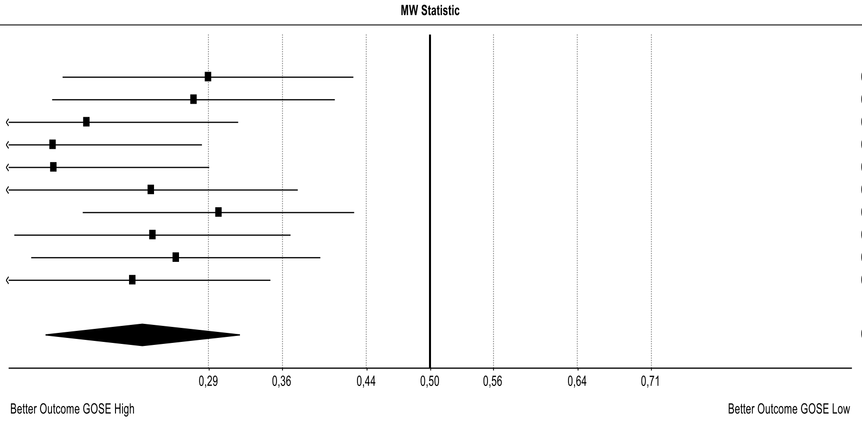

Analysis	MW	95,00%-CI	N1/N2	P-Value
**Z1333-6D**				
SF36MCS-6	0,2896	(0,1519 to 0,4274)	33 / 35	0,0028
SF12MCS-6	0,2761	(0,1420 to 0,4102)	37 / 37	0,0011
SF36PCS-6	0,1745	(0,0307 to 0,3182)	33 / 35	0,0000
SF12PCS-6	0,1424	(0,0008 to 0,2841)	37 / 37	0,0000
QOLIBRI-6	0,1428	(0,0000 to 0,2904)	33 / 33	0,0000
QOLI-OS-6	0,2357	(0,0966 to 0,3748)	39 / 34	0,0002
GAD7-6	0,2996	(0,1706 to 0,4285)	34 / 35	0,0023
PHQ9-6	0,2369	(0,1059 to 0,3679)	34 / 36	0,0001
PCL5-6	0,2591	(0,1219 to 0,3963)	34 / 34	0,0006
RPQ-6	0,2183	(0,0879 to 0,3487)	34 / 38	0,0000
**Wei-Lachin**				
COMBINED	0,2275	(0,1352 to 0,3198)	348 / 354	0,0000

GOSE/-Q 7-8 vs. GOSE/-Q 5-6- Severe TBI

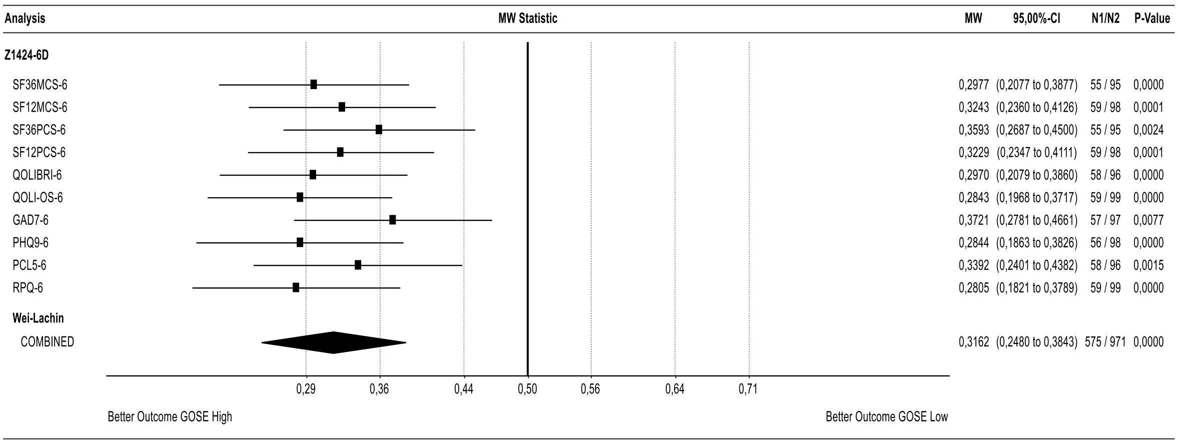

GOSE/-Q 5-6 vs. GOSE/-Q 3-4 - Severe TBI

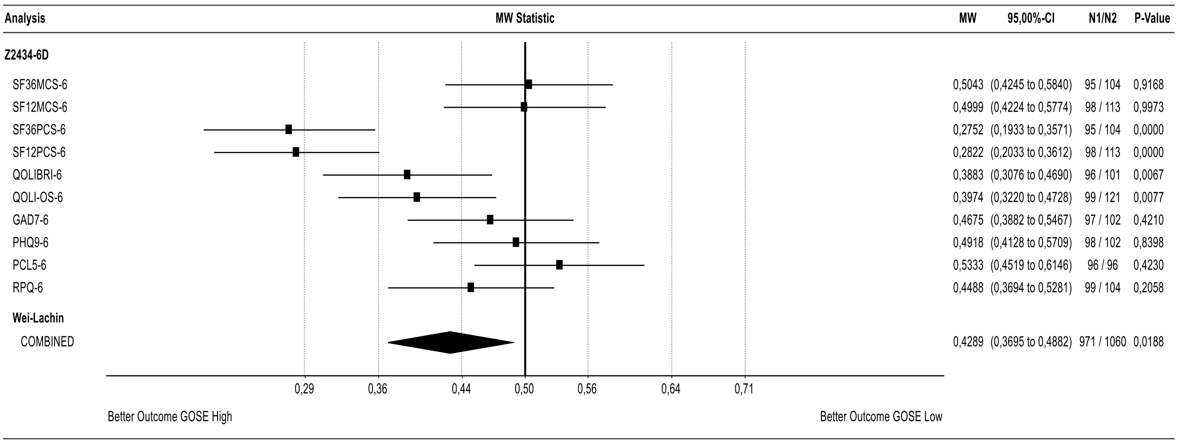

GOSE/-Q 7-8 vs. GOSE/-Q 3-4 - Severe TBI

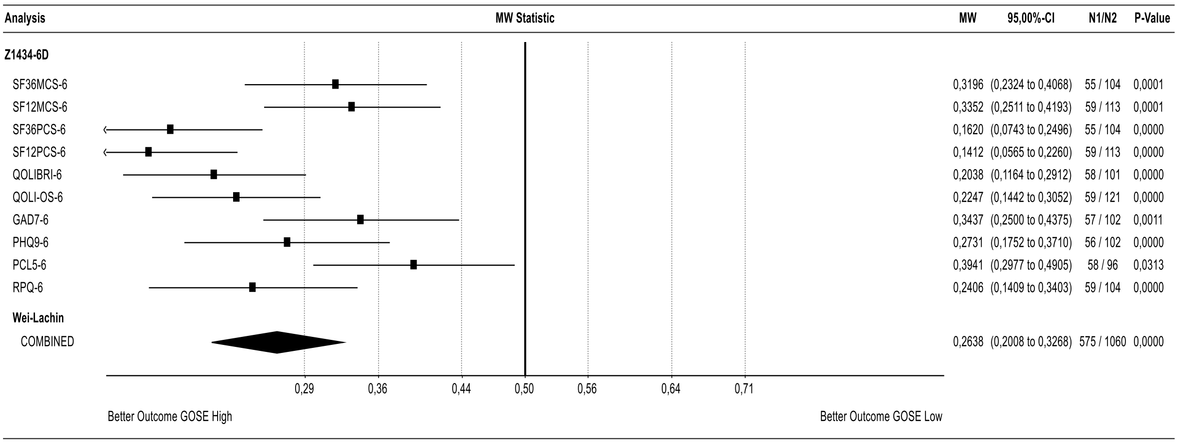

12 months after TBI (data as available)

GOSE/-Q 7-8 vs. GOSE/-Q 5-6- Complicated Mild TBI

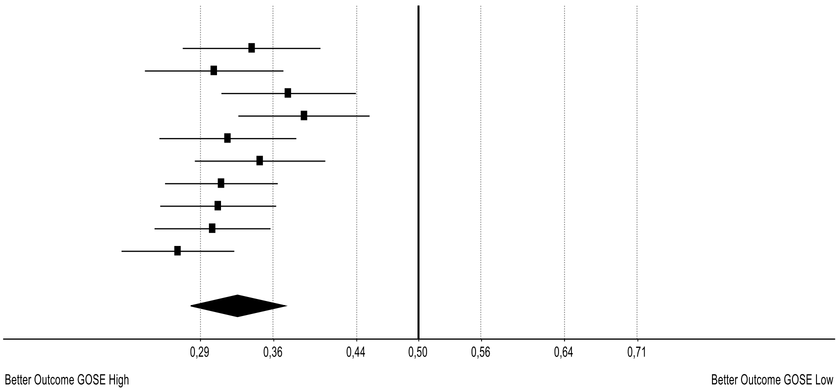

Analysis	MW	95,00%-CI	N1/N2	P-Value
**Z1222-YD**				
SF36MCS-12	0,3395	(0,2732 to 0,4058)	250 / 133	0,0000
SF12MCS-12	0,3033	(0,2370 to 0,3697)	263 / 138	0,0000
SF36PCS-12	0,3746	(0,3100 to 0,4393)	250 / 133	0,0001
SF12PCS-12	0,3897	(0,3267 to 0,4528)	263 / 138	0,0006
QOLIBRI-12	0,3163	(0,2503 to 0,3824)	253 / 135	0,0000
QOLI-OS-12	0,3475	(0,2847 to 0,4103)	267 / 142	0,0000
GAD7-12	0,3101	(0,2560 to 0,3642)	255 / 138	0,0000
PHQ9-12	0,3072	(0,2517 to 0,3628)	254 / 135	0,0000
PCL5-12	0,3016	(0,2458 to 0,3575)	253 / 135	0,0000
RPQ-12	0,2684	(0,2144 to 0,3224)	260 / 141	0,0000
**Wei-Lachin**				
COMBINED	0,3258	(0,2805 to 0,3711)	2568 / 1368	0,0000

GOSE/-Q 5-6 vs. GOSE/-Q 3-4 - Complicated Mild TBI

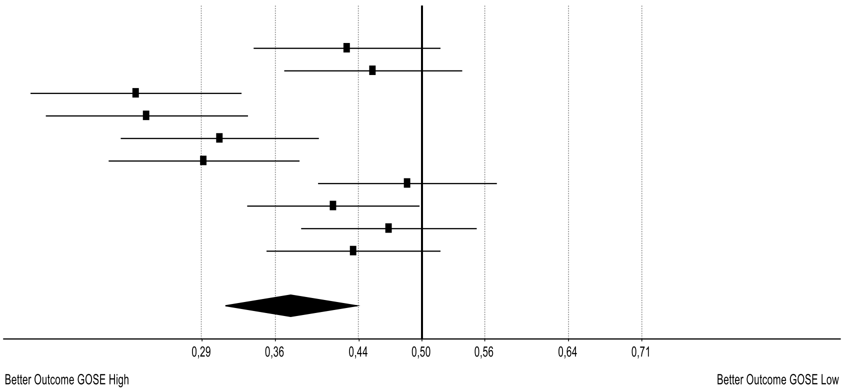

Analysis	MW	95,00%-CI	N1/N2	P-Value
**Z2232-YD**				
SF36MCS-12	0,4288	(0,3399 to 0,5177)	133 / 63	0,1165
SF12MCS-12	0,4535	(0,3685 to 0,5386)	138 / 68	0,2842
SF36PCS-12	0,2272	(0,1263 to 0,3281)	133 / 63	0,0000
SF12PCS-12	0,2374	(0,1408 to 0,3340)	138 / 68	0,0000
QOLIBRI-12	0,3073	(0,2125 to 0,4021)	135 / 63	0,0001
QOLI-OS-12	0,2923	(0,2010 to 0,3835)	142 / 69	0,0000
GAD7-12	0,4866	(0,4010 to 0,5722)	138 / 61	0,7585
PHQ9-12	0,4159	(0,3335 to 0,4983)	135 / 62	0,0455
PCL5-12	0,4690	(0,3852 to 0,5528)	135 / 62	0,4685
RPQ-12	0,4349	(0,3518 to 0,5180)	141 / 60	0,1249
**Wei-Lachin**				
COMBINED	0,3753	(0,3128 to 0,4378)	1368 / 639	0,0001

GOSE/-Q 7-8 vs. GOSE/-Q 3-4 - Complicated Mild TBI

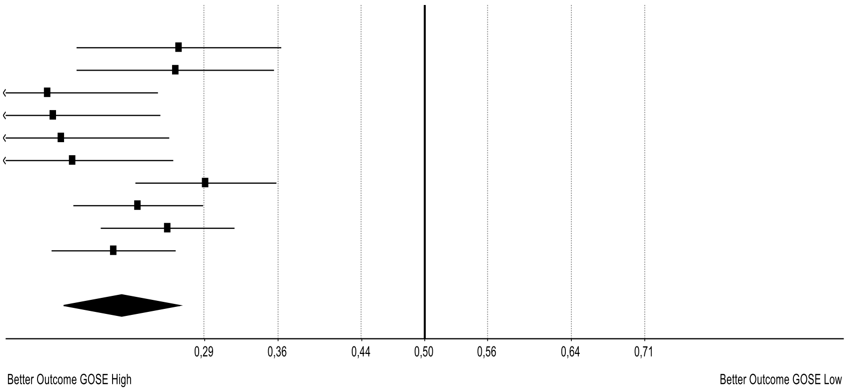

Analysis	MW	95,00%-CI	N1/N2	P-Value
**Z1232-YD**				
SF36MCS-12	0,2659	(0,1682 to 0,3637)	250 / 63	0,0000
SF12MCS-12	0,2625	(0,1682 to 0,3568)	263 / 68	0,0000
SF36PCS-12	0,1400	(0,0340 to 0,2460)	250 / 63	0,0000
SF12PCS-12	0,1461	(0,0443 to 0,2479)	263 / 68	0,0000
QOLIBRI-12	0,1534	(0,0500 to 0,2569)	253 / 63	0,0000
QOLI-OS-12	0,1641	(0,0675 to 0,2606)	267 / 69	0,0000
GAD7-12	0,2914	(0,2239 to 0,3590)	255 / 61	0,0000
PHQ9-12	0,2268	(0,1651 to 0,2885)	254 / 62	0,0000
PCL5-12	0,2550	(0,1915 to 0,3186)	253 / 62	0,0000
RPQ-12	0,2036	(0,1444 to 0,2628)	260 / 60	0,0000
**Wei-Lachin**				
COMBINED	0,2109	(0,1554 to 0,2664)	2568 / 639	0,0000

GOSE/-Q 7-8 vs. GOSE/-Q 5-6- Severe TBI

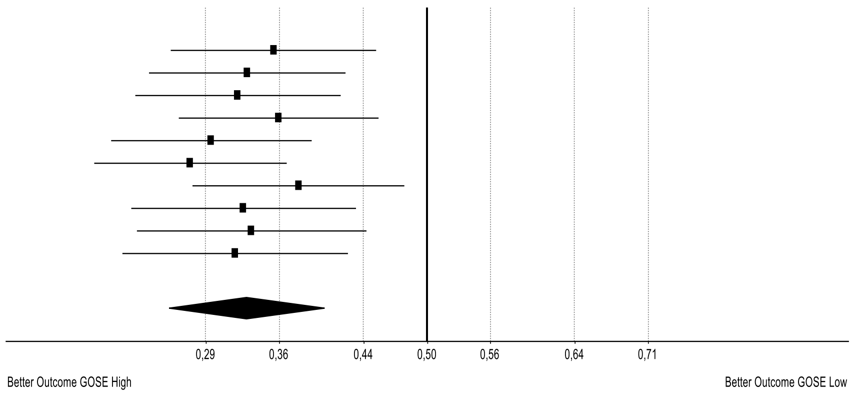

Analysis	MW	95,00%-CI	N1/N2	P-Value
**Z1424-YD**				
SF36MCS-12	0,3546	(0,2572 to 0,4520)	43 / 87	0,0034
SF12MCS-12	0,3297	(0,2363 to 0,4230)	47 / 94	0,0003
SF36PCS-12	0,3208	(0,2235 to 0,4181)	43 / 87	0,0003
SF12PCS-12	0,3591	(0,2643 to 0,4539)	47 / 94	0,0036
QOLIBRI-12	0,2953	(0,2001 to 0,3905)	45 / 90	0,0000
QOLI-OS-12	0,2754	(0,1842 to 0,3667)	48 / 95	0,0000
GAD7-12	0,3782	(0,2778 to 0,4785)	46 / 92	0,0174
PHQ9-12	0,3260	(0,2194 to 0,4325)	46 / 91	0,0014
PCL5-12	0,3338	(0,2251 to 0,4425)	45 / 89	0,0027
RPQ-12	0,3181	(0,2110 to 0,4253)	47 / 93	0,0009
**Wei-Lachin**				
COMBINED	0,3291	(0,2555 to 0,4027)	457 / 912	0,0000

GOSE/-Q 5-6 vs. GOSE/-Q 3-4 - Severe TBI

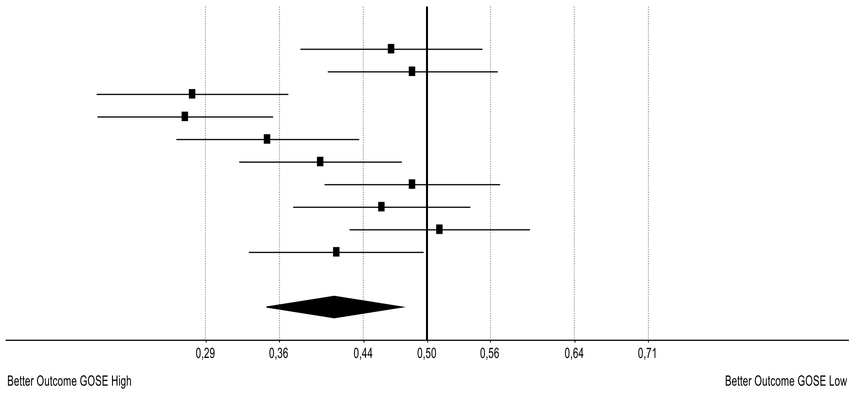

Analysis	MW	95,00%-CI	N1/N2	P-Value
**Z2434-YD**				
SF36MCS-12	0,4666	(0,3804 to 0,5529)	87 / 83	0,4482
SF12MCS-12	0,4865	(0,4057 to 0,5673)	94 / 100	0,7441
SF36PCS-12	0,2777	(0,1871 to 0,3682)	87 / 83	0,0000
SF12PCS-12	0,2708	(0,1875 to 0,3541)	94 / 100	0,0000
QOLIBRI-12	0,3489	(0,2621 to 0,4358)	90 / 84	0,0007
QOLI-OS-12	0,3994	(0,3222 to 0,4765)	95 / 115	0,0106
GAD7-12	0,4864	(0,4029 to 0,5699)	92 / 87	0,7503
PHQ9-12	0,4575	(0,3734 to 0,5416)	91 / 86	0,3221
PCL5-12	0,5120	(0,4265 to 0,5976)	89 / 84	0,7828
RPQ-12	0,4143	(0,3314 to 0,4973)	93 / 91	0,0430
**Wei-Lachin**				
COMBINED	0,4120	(0,3481 to 0,4760)	912 / 913	0,0070

GOSE/-Q 7-8 vs. GOSE/-Q 3-4 - Severe TBI

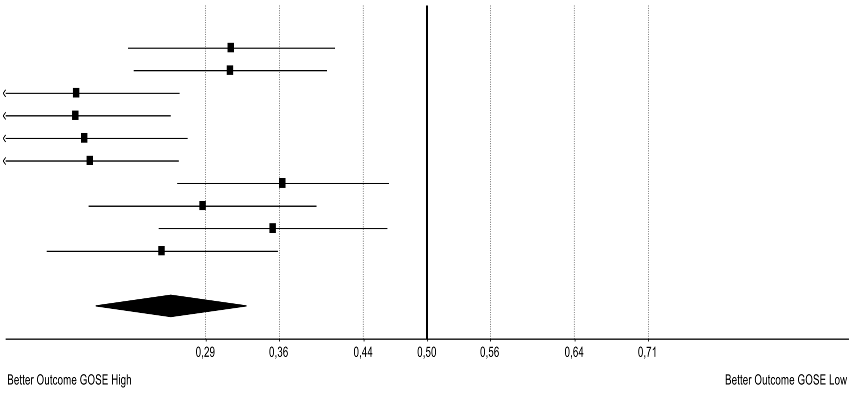

Analysis	MW	95,00%-CI	N1/N2	P-Value
**Z1434-YD**				
SF36MCS-12	0,3145	(0,2162 to 0,4129)	43 / 83	0,0002
SF12MCS-12	0,3135	(0,2220 to 0,4050)	47 / 100	0,0001
SF36PCS-12	0,1678	(0,0700 to 0,2657)	43 / 83	0,0000
SF12PCS-12	0,1672	(0,0770 to 0,2574)	47 / 100	0,0000
QOLIBRI-12	0,1755	(0,0780 to 0,2731)	45 / 84	0,0000
QOLI-OS-12	0,1803	(0,0960 to 0,2647)	48 / 115	0,0000
GAD7-12	0,3636	(0,2629 to 0,4643)	46 / 87	0,0079
PHQ9-12	0,2874	(0,1792 to 0,3956)	46 / 86	0,0001
PCL5-12	0,3540	(0,2457 to 0,4623)	45 / 84	0,0082
RPQ-12	0,2488	(0,1390 to 0,3585)	47 / 91	0,0000
**Wei-Lachin**				
COMBINED	0,2573	(0,1856 to 0,3289)	457 / 913	0,0000

3 months after TBI (completers)

GOSE/-Q 7-8 vs. GOSE/-Q 5-6- Complicated Mild TBI

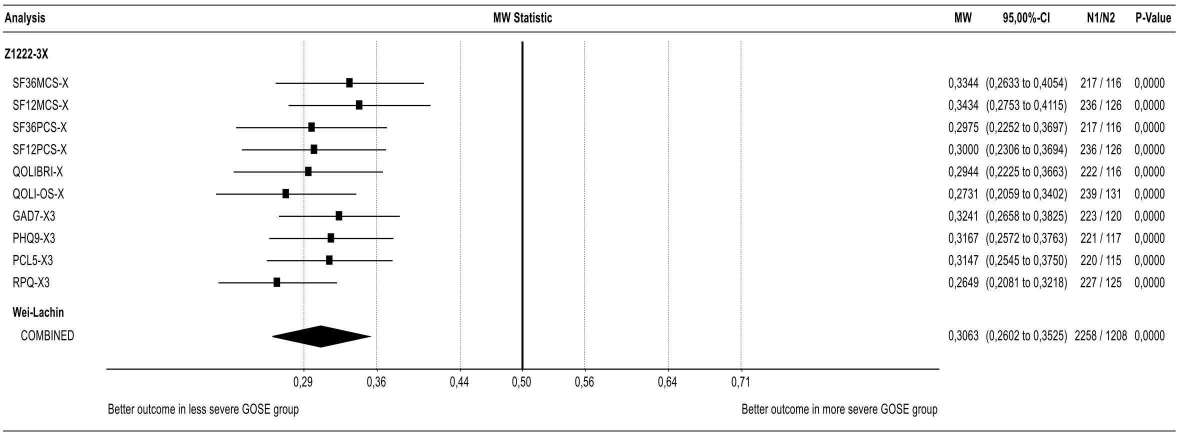

GOSE/-Q 5-6 vs. GOSE/-Q 3-4 - Complicated Mild TBI

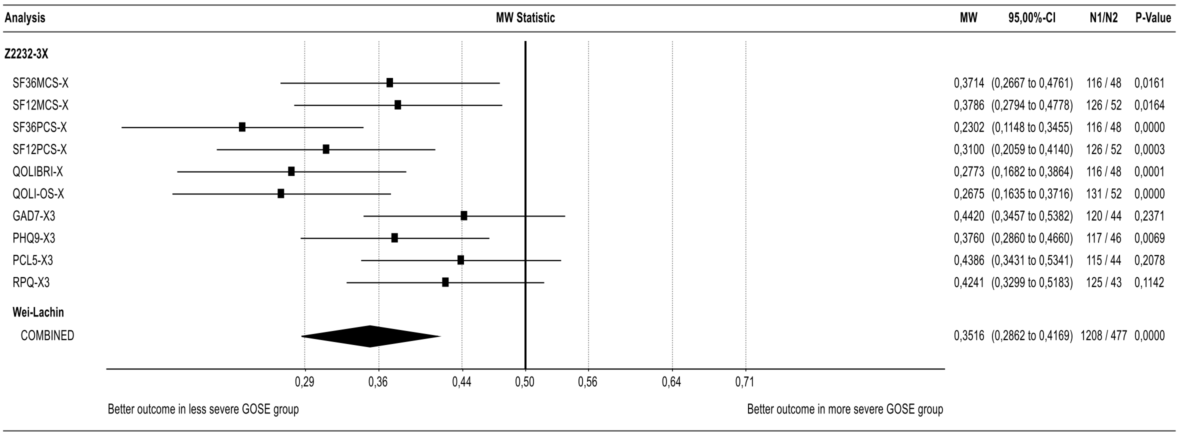

GOSE/-Q 7-8 vs. GOSE/-Q 3-4 - Complicated Mild TBI

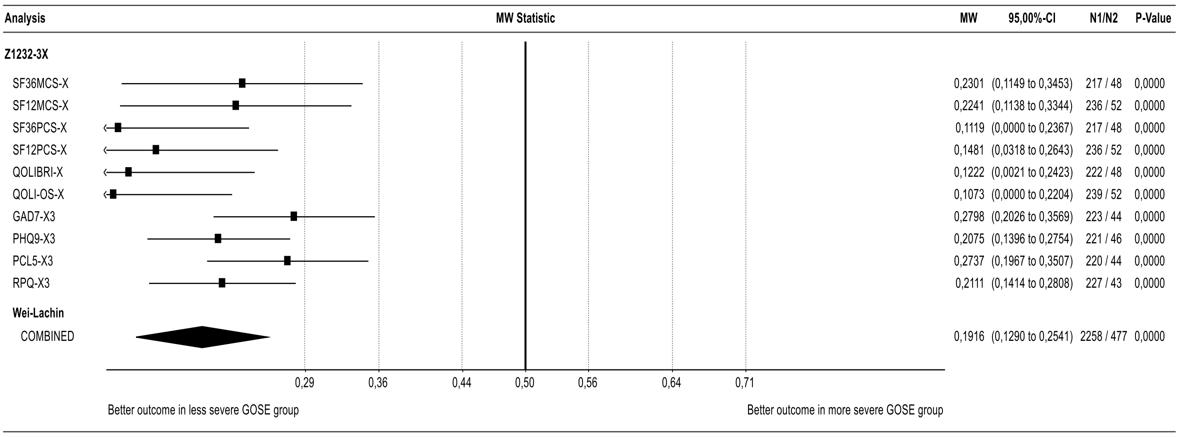

GOSE/-Q 7-8 vs. GOSE/-Q 5-6- Severe TBI

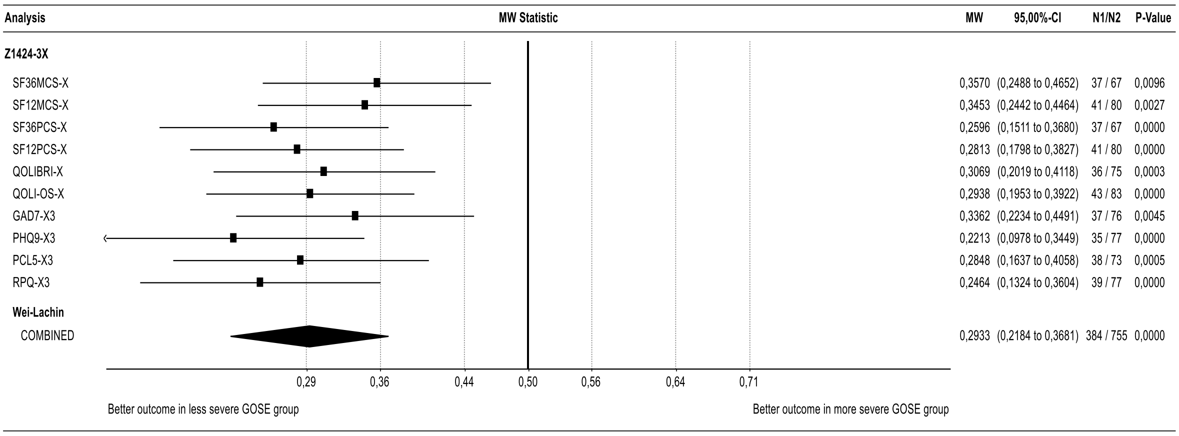

GOSE/-Q 5-6 vs. GOSE/-Q 3-4 - Severe TBI

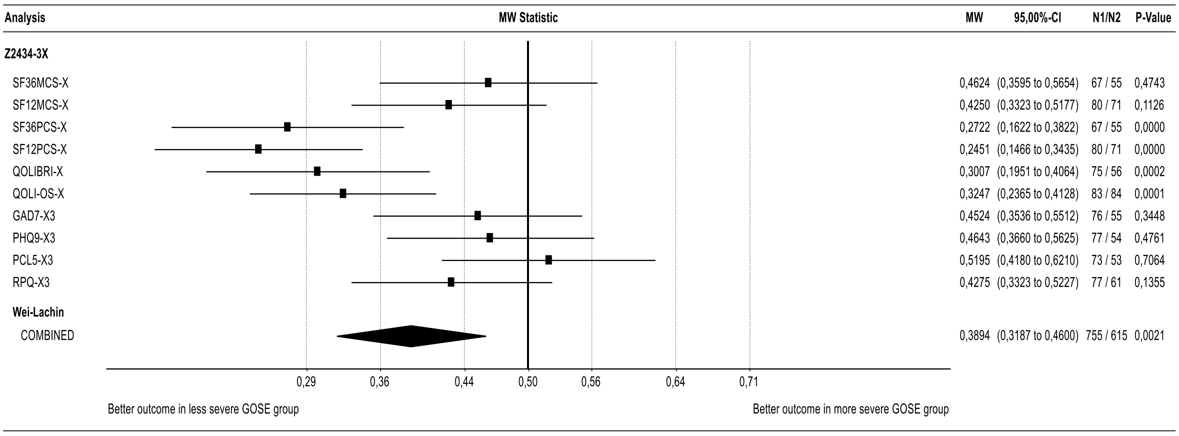

GOSE/-Q 7-8 vs. GOSE/-Q 3-4 - Severe TBI

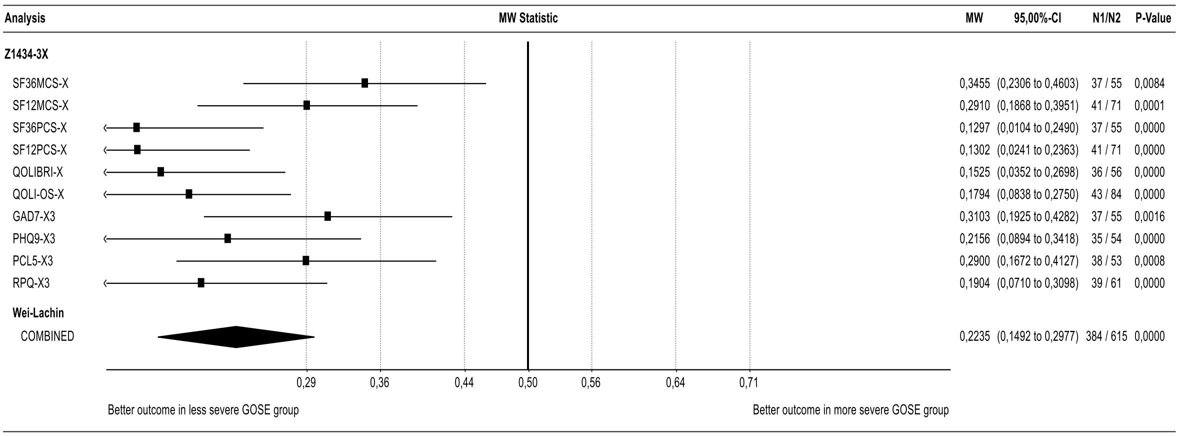

6 months after TBI (completers)

GOSE/-Q 7-8 vs. GOSE/-Q 5-6- Complicated Mild TBI

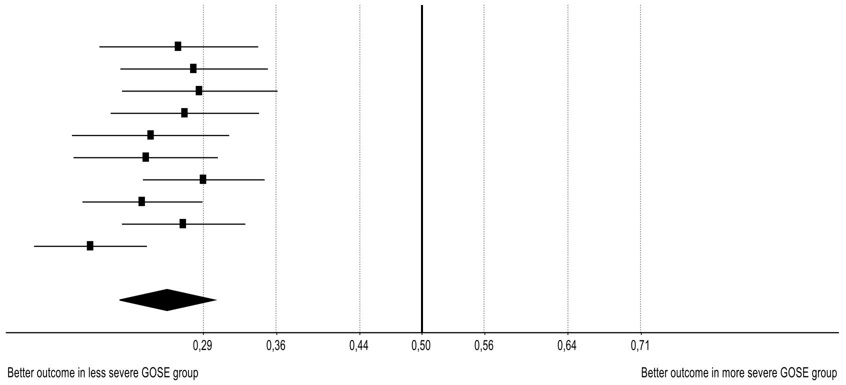

Analysis	MW	95,00%-CI	N1/N2	P-Value
**Z1222-6X**				
SF36MCS-X	0,2664	(0,1902 to 0,3426)	239 / 109	0,0000
SF12MCS-X	0,2812	(0,2103 to 0,3521)	255 / 123	0,0000
SF36PCS-X	0,2866	(0,2118 to 0,3615)	239 / 109	0,0000
SF12PCS-X	0,2720	(0,2009 to 0,3432)	255 / 123	0,0000
QOLIBRI-X	0,2394	(0,1639 to 0,3149)	240 / 112	0,0000
QOLI-OS-X	0,2349	(0,1655 to 0,3043)	258 / 127	0,0000
GAD7-X6	0,2903	(0,2317 to 0,3490)	244 / 111	0,0000
PHQ9-X6	0,2315	(0,1736 to 0,2894)	241 / 112	0,0000
PCL5-X6	0,2709	(0,2116 to 0,3303)	241 / 106	0,0000
RPQ-X6	0,1817	(0,1277 to 0,2357)	250 / 115	0,0000
**Wei-Lachin**				
COMBINED	0,2555	(0,2099 to 0,3012)	2462 / 1147	0,0000

GOSE/-Q 5-6 vs. GOSE/-Q 3-4 - Complicated Mild TBI

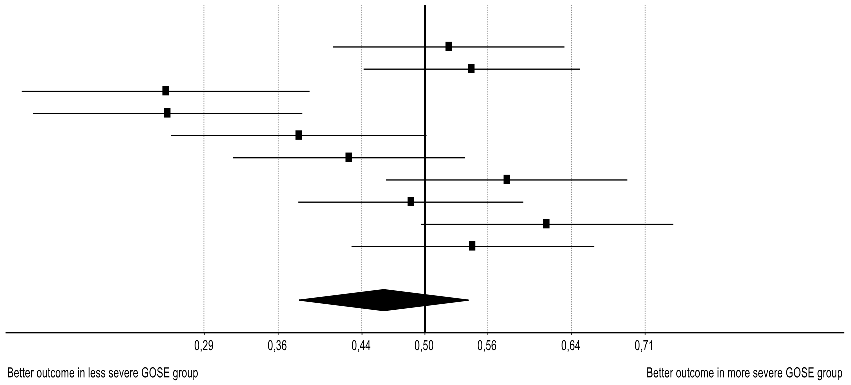

Analysis	MW	95,00%-CI	N1/N2	P-Value
**Z2232-6X**				
SF36MCS-X	0,5232	(0,4130 to 0,6334)	109 / 32	0,6796
SF12MCS-X	0,5449	(0,4417 to 0,6482)	123 / 36	0,3937
SF36PCS-X	0,2532	(0,1160 to 0,3903)	109 / 32	0,0004
SF12PCS-X	0,2550	(0,1262 to 0,3837)	123 / 36	0,0002
QOLIBRI-X	0,3800	(0,2579 to 0,5021)	112 / 33	0,0542
QOLI-OS-X	0,4278	(0,3170 to 0,5386)	127 / 36	0,2016
GAD7-X6	0,5785	(0,4636 to 0,6934)	111 / 32	0,1803
PHQ9-X6	0,4870	(0,3796 to 0,5944)	112 / 32	0,8128
PCL5-X6	0,6169	(0,4966 to 0,7372)	106 / 32	0,0569
RPQ-X6	0,5461	(0,4304 to 0,6618)	115 / 30	0,4350
**Wei-Lachin**				
COMBINED	0,4613	(0,3803 to 0,5423)	1147 / 331	0,3487

GOSE/-Q 7-8 vs. GOSE/-Q 3-4 - Complicated Mild TBI

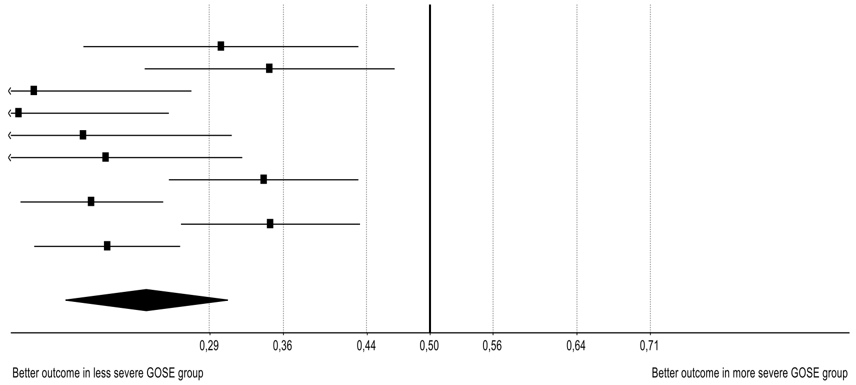

Analysis	MW	95,00%-CI	N1/N2	P-Value
**Z1232-6X**				
SF36MCS-X	0,3011	(0,1698 to 0,4323)	239 / 32	0,0030
SF12MCS-X	0,3471	(0,2278 to 0,4664)	255 / 36	0,0120
SF36PCS-X	0,1225	(0,0000 to 0,2727)	239 / 32	0,0000
SF12PCS-X	0,1077	(0,0000 to 0,2512)	255 / 36	0,0000
QOLIBRI-X	0,1696	(0,0278 to 0,3115)	240 / 33	0,0000
QOLI-OS-X	0,1914	(0,0612 to 0,3215)	258 / 36	0,0000
GAD7-X6	0,3416	(0,2514 to 0,4318)	244 / 32	0,0006
PHQ9-X6	0,1776	(0,1096 to 0,2456)	241 / 32	0,0000
PCL5-X6	0,3483	(0,2628 to 0,4338)	241 / 32	0,0005
RPQ-X6	0,1926	(0,1230 to 0,2622)	250 / 30	0,0000
**Wei-Lachin**				
COMBINED	0,2299	(0,1528 to 0,3071)	2462 / 331	0,0000

GOSE/-Q 7-8 vs. GOSE/-Q 5-6 - Moderate TBI

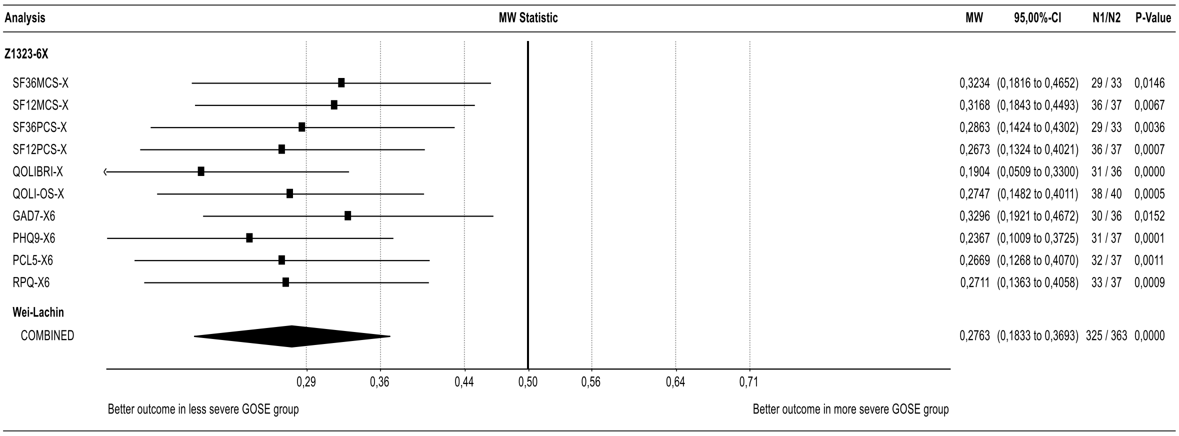

GOSE/-Q 7-8 vs. GOSE/-Q 5-6- Severe TBI

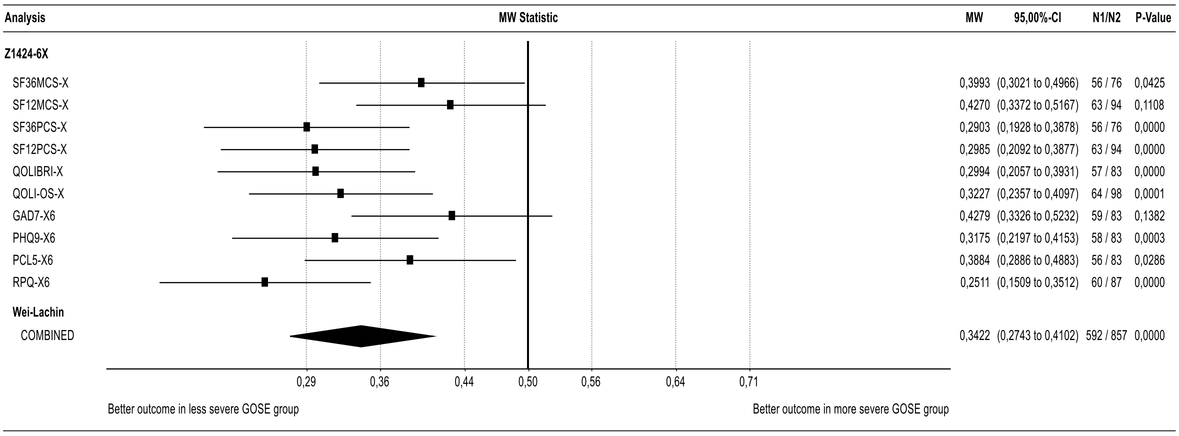

<u>12 months after TBI (completers)</u>

GOSE/-Q 7-8 vs. GOSE/-Q 5-6- Complicated Mild TBI

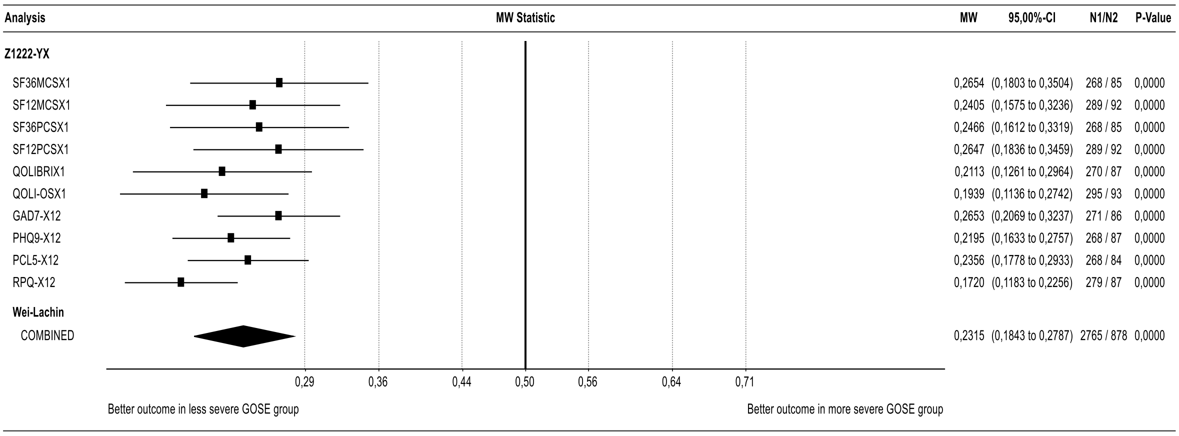

GOSE/-Q 7-8 vs. GOSE/-Q 5-6- Severe TBI

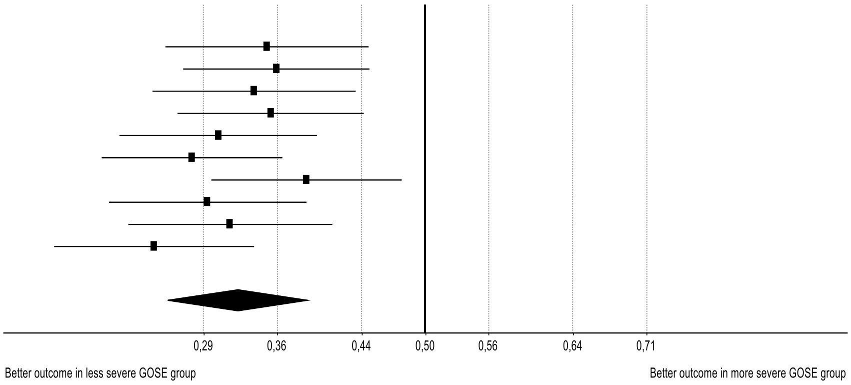

Analysis	MW	95,00%-CI	N1/N2	P-Value
**Z1424-YX**				
SF36MCSX1	0,3503	(0,2541 to 0,4464)	61 / 75	0,0023
SF12MCSX1	0,3592	(0,2711 to 0,4472)	72 / 90	0,0017
SF36PCSX1	0,3379	(0,2417 to 0,4341)	61 / 75	0,0010
SF12PCSX1	0,3537	(0,2656 to 0,4418)	72 / 90	0,0011
QOLIBRIX1	0,3041	(0,2105 to 0,3977)	64 / 80	0,0000
QOLI-OSX1	0,2790	(0,1937 to 0,3644)	77 / 96	0,0000
GAD7-X12	0,3877	(0,2976 to 0,4779)	65 / 81	0,0146
PHQ9-X12	0,2940	(0,2002 to 0,3879)	66 / 80	0,0000
PCL5-X12	0,3152	(0,2185 to 0,4120)	66 / 77	0,0002
RPQ-X12	0,2433	(0,1484 to 0,3382)	69 / 84	0,0000
**Wei-Lachin**				
COMBINED	0,3224	(0,2564 to 0,3885)	673 / 828	0,0000
