# Supplementary figures and images for: Sensitivity of outcome instruments in a priori selected patient groups after traumatic brain injury: Results from the CENTER-TBI study
Source: PLoS One. 2023 Apr 7;18(4):e0280796. doi: 10.1371/journal.pone.0280796 (PMC10081802; doi:10.1371/journal.pone.0280796)

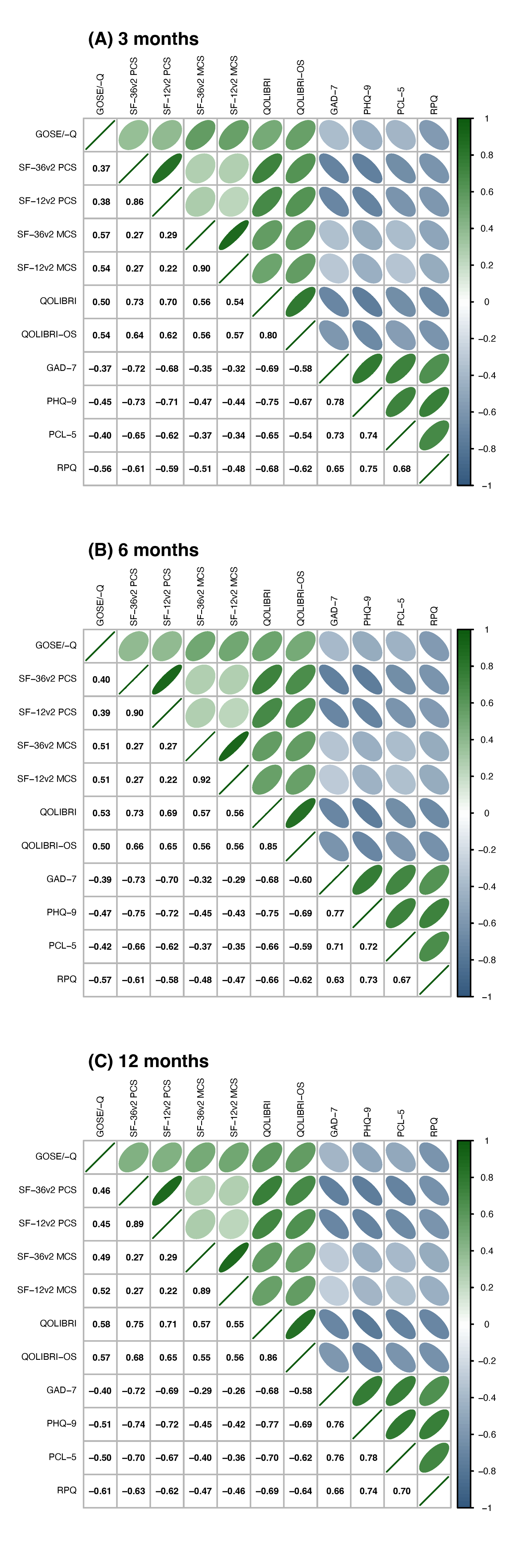

Supplement: S1 Fig — Graded blue ellipses indicate low (light blue) to high (dark blue) negative correlations, while graded green ellipses indicate low (light green) to high (dark green) positive correlations. GOSE/-Q = Combined information on recovery status using the Glasgow Outcome Scale–Extended and its questionnaire version; SF-36v2 = 36-item Short Form Health Survey–version 2; SF-12v2 = 12-Item Short Form Survey–version 2; PCS = Physical Component Summary Score, MCS = Mental Component Summary Score; QOLIBRI = Quality of Life after Traumatic Brain Injury; QOLIBRI-OS = Quality of Life after Traumatic Brain Injury–Overall Scale; GAD-7 = Generalized Anxiety Disorder-7; PHQ-9 = Patient Health Questionnaire-9; PCL-5 = Posttraumatic Stress Disorder Checklist for DSM-5; RPQ = Rivermead Post-Concussion Symptoms Questionnaire. (TIF) [file pone.0280796.s011.tif]
